# Homoleptic Fe(III) and Fe(IV) Complexes of a Dianionic C_3_-Symmetric Scorpionate

**DOI:** 10.1021/acs.inorgchem.3c00871

**Published:** 2023-06-27

**Authors:** Serhii Tretiakov, Martin Lutz, Charles James Titus, Frank de Groot, Joscha Nehrkorn, Thomas Lohmiller, Karsten Holldack, Alexander Schnegg, Maxime François Xavier Tarrago, Peng Zhang, Shengfa Ye, Dmitry Aleshin, Alexander Pavlov, Valentin Novikov, Marc-Etienne Moret

**Affiliations:** † Organic Chemistry & Catalysis, Institute for Sustainable and Circular Chemistry, Utrecht University, 3584 CG Utrecht, The Netherlands; ‡ Structural Biochemistry, Bijvoet Centre for Biomolecular Research, Faculty of Science, Utrecht University, 3584 CG Utrecht, The Netherlands; § Department of Physics, Stanford University, Stanford, California 94305, United States; ∥ Materials Chemistry & Catalysis, Debye Institute for Materials Science, Utrecht University, 3584 CG Utrecht, The Netherlands; ⊥ Max-Planck-Institute for Chemical Energy Conversion, EPR Research Group, 45470 Mülheim/Ruhr, Germany; # Department Spins in Energy Conversion and Quantum Information Science, Helmholtz Zentrum Berlin für Materialien und Energie GmbH, EPR4 Energy Joint Lab, 12489 Berlin, Germany; ∇ Department of Optics and Beamlines, Helmholtz Zentrum Berlin für Materialien und Energie GmbH, 12489 Berlin, Germany; ○ Department of Molecular Theory and Spectroscopy, Max Planck Institut für Kohlenforschung, 45470 Mülheim/Ruhr, Germany; ◆ State Key Laboratory of Catalysis, Dalian Institute of Chemical Physics, Chinese Academy of Sciences, Dalian 116023, P. R. China; ¶ University of Chinese Academy of Sciences, Beijing 10049, China; & A.N. Nesmeyanov Institute of Organoelement Compounds, Russian Academy of Sciences, Vavilova Street 28, Moscow 119991, Russia; ● Moscow Institute of Physics and Technology, Institutskiy per., 9, Dolgoprudny, Moscow 119991, Russia

## Abstract

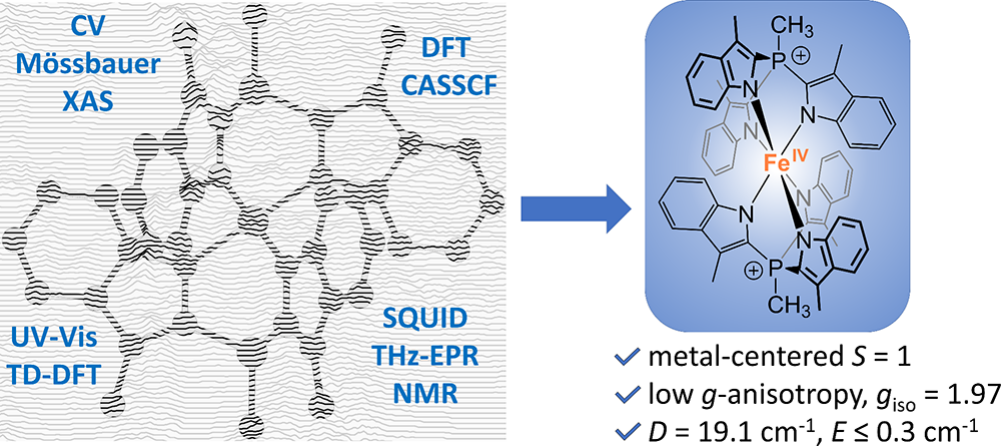

High-valent iron species have been implicated as key intermediates in catalytic oxidation reactions, both in biological and synthetic systems. Many heteroleptic Fe(IV) complexes have now been prepared and characterized, especially using strongly π-donating oxo, imido, or nitrido ligands. On the other hand, homoleptic examples are scarce. Herein, we investigate the redox chemistry of iron complexes of the dianonic tris-skatylmethylphosphonium (TSMP^2–^) scorpionate ligand. One-electron oxidation of the tetrahedral, bis-ligated [(TSMP)_2_Fe^II^]^2–^ leads to the octahedral [(TSMP)_2_Fe^III^]^−^. The latter undergoes thermal spin-cross-over both in the solid state and solution, which we characterize using superconducting quantum inference device (SQUID), Evans method, and paramagnetic nuclear magnetic resonance spectroscopy. Furthermore, [(TSMP)_2_Fe^III^]^−^ can be reversibly oxidized to the stable high-valent [(TSMP)_2_Fe^IV^]^0^ complex. We use a variety of electrochemical, spectroscopic, and computational techniques as well as SQUID magnetometry to establish a triplet (*S* = 1) ground state with a metal-centered oxidation and little spin delocalization on the ligand. The complex also has a fairly isotropic *g*-tensor (*g*_iso_ = 1.97) combined with a positive zero-field splitting (ZFS) parameter *D* (+19.1 cm^–1^) and very low rhombicity, in agreement with quantum chemical calculations. This thorough spectroscopic characterization contributes to a general understanding of octahedral Fe(IV) complexes.

## Abstract 2

The redox chemistry of bis-ligated homoleptic complexes of iron with a dianionic C_3_-symmetric tris-skatylmethylphosphonium (TSMP^2−^) scorpionate ligand is investigated. The pseudotetrahedral [(TSMP)_2_Fe^II^]^2−^ complex can undergo two consecutive one-electron oxidations to give locally octahedral Fe(III) and Fe(IV) compounds. The Fe(III) complex undergoes thermal spin-cross-over between the doublet and sextet states. The Fe(IV) compound has a triplet ground state. Its electronic structure is extensively analyzed by a combination of spectroscopic and computational methods.

## Introduction

**Chart 1:**
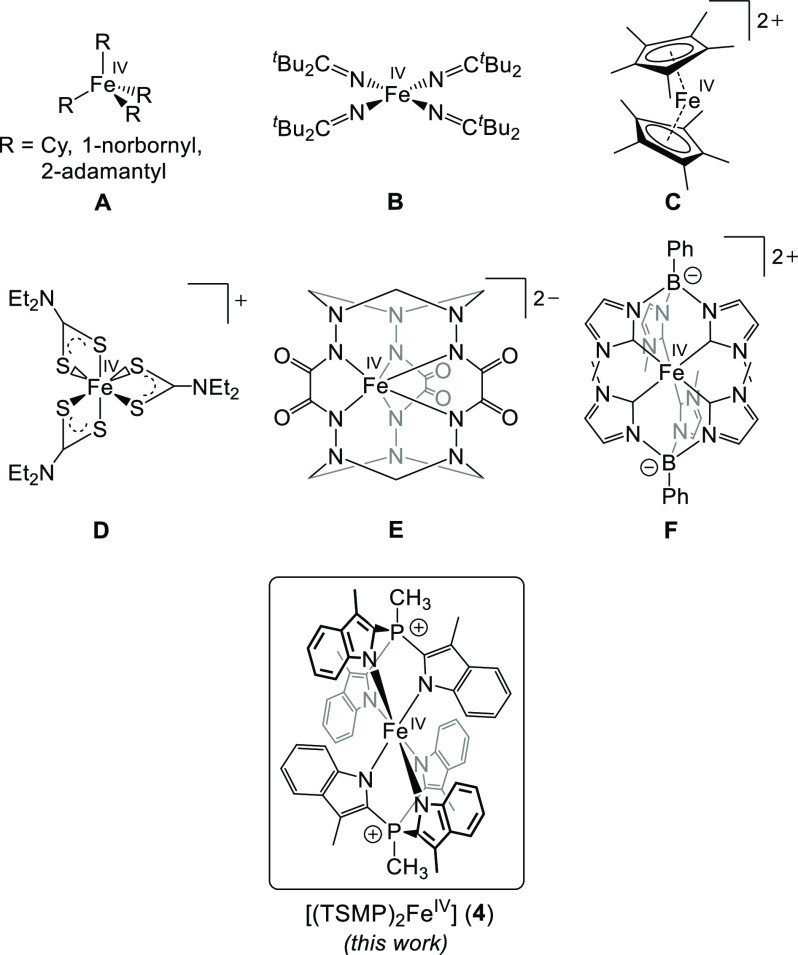
Reported Homoleptic Fe(IV) Complexes^20,21,30,22−29^

Fe(IV) compounds, both heme- and non-heme-based,^1−4^ are crucial intermediates in many biological transformations. As such, they provide inspiration for the development of small-molecule catalysts for green oxidation processes.^4−6^ Due to their generally highly-oxidizing nature, their isolation and spectroscopic characterization have been challenging. Perhaps, the most studied members of this family involve heteroleptic Fe(IV) complexes stabilized by strong π-donating ligands^7^ (oxo-,^3,5,8,9^ imido-,^10−12^ nitrido-,^13−17^ isocyanide,^18^ and ketimide^19^). In contrast, homoleptic Fe(IV) complexes are relatively scarce. Among these, a [FeF_4_] species was cryogenically trapped in neon or argon matrices^20,21^ and, according to DFT calculations, is expected to have a quintet ground state (*S* = 2). Due to high ionicity of the Fe–F bond, the existence of [FeF_4_] as a bulk material under normal conditions is thought unlikely. Next, a family of tetrahedral singlet (*S* = 0) Fe(IV) tetraalkyl complexes is known (**A** in Chart 1),^22−24^ which decompose at room temperature in a matter of days. Similar instability has been reported in a distorted square planar singlet complex **B**,^19,23^ an extremely rare example of a nontetrahedral FeX_4_ compound. The triplet (*S* = 1) dicationic decamethylferrocenium compound **C** was prepared by oxidation of decamethylferrocene in liquid sulfur dioxide^25^ and is sufficiently stable for spectroscopic characterization and X-ray crystal structure determination. The latter reveals that the Cp* rings can tilt with respect to one another depending on the counterion, namely, the tilt angle is 0° for the [Sb_2_F_11_]^−^ anion but 16.56° for SbF_6_^–^, which is caused by coordination of the anion to the Fe(IV) center. Therefore, these complexes can be considered contingently homoleptic. A stable triplet dithiocarbamate complex **D** was synthesized in 1972^26,27^ and is one of the first reported homoleptic Fe(IV) complexes. Its electronic structure was revisited in great detail more recently^28^ using a combination of spectroscopic and computational techniques, proving its identity as a true Fe(IV) compound. An indefinitely stable triplet hexahydrazide **E** that forms in water upon air oxidation^29^ was extensively characterized as having a triplet spin state and trigonal prismatic geometry. Finally, an NHC-based phenylborate complex **F** was synthesized recently,^30^ which has a triplet ground state and local *D*_3d_ symmetry at the metal center.

Due to the presence of weakly π-donating hard anionic N-donors, pyrrolide-based ligands have a potential for stabilizing high-valent metal centers.^31^ As a matter of fact, porphyrine and corrole-based coordination compounds are ubiquitous in nature and often support oxidized reactive intermediates.^32,33^ Nonetheless, the majority of these systems feature 4-fold planar coordination geometry around the metal, raising the question of what interesting electronic properties and reactivity alternative symmetries could provide. Some examples of such geometries include unconjugated dipyrrolylmethane^34−38^ and tris-pyrrolylethane^39^ metal complexes. However, in the case of these molecules, the absence of conjugation on the ligand leads to the relatively energetic aromatic π-orbitals which participate in molecular redox events.

We have previously reported a formally dianionic C_3_-symmetric tris-skatylmethylphosphonium (TSMP^2–^) ligand platform that is based on a π-extended pyrrole-based aromatic system, 3-methylindole (skatole).^40^ It also has a positively charged bridgehead phosphonium atom, which further lowers the energy of its highest occupied molecular orbital (HOMO). Herein, we show that these two aspects are sufficient to access a stable homoleptic, octahedral [(TSMP)_2_Fe^IV^] species (**4**, Chart 1) by one-electron oxidation of its isostructural Fe(III) analogue. We use a variety of electrochemical (cyclic voltammetry), spectroscopic (XAS, THz-EPR, ^57^Fe Mössbauer, paramagnetic NMR and optical spectroscopy), and computational techniques (DFT, TD-DFT, CASSCF/NEVPT2), supported by superconducting quantum inference device (SQUID) magnetometry, to establish a primarily metal-centered oxidation and provide a detailed picture of the electronic structure of **4**.

## Synthesis and Characterization

**Scheme 1:**
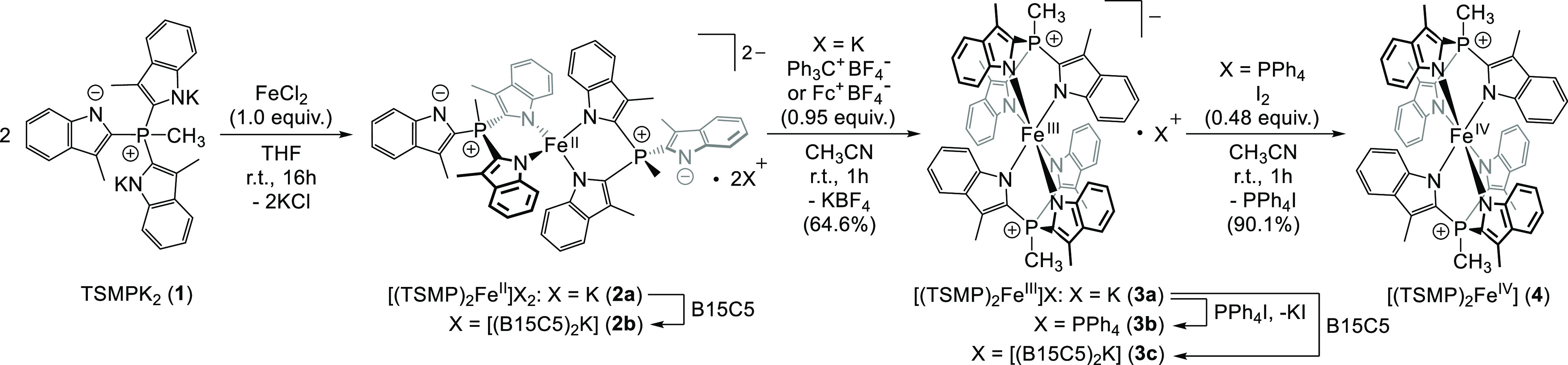
Synthesis of Metal Complexes **2a,b**, **3a–c**, and **4**

The high-spin Fe(II) complex [(TSMP)_2_Fe^II^]K_2_ (**2a**) and its crystallizable benzo-15-crown-5 (B15C5) adduct [(TSMP)_2_Fe^II^][(B15C5)_2_K]_2_ (**2b**) were synthesized as previously described^40^ using iron dichloride and two equivalents of the TSMPK_2_ salt (**1**) in THF (Scheme 1), followed by addition of benzo-15-crown-5 if needed. Complex **2a** is extremely air-sensitive and is immediately converted into the deep-blue Fe(III) compound [(TSMP)_2_Fe^III^]K (**3a**) upon exposure to even trace amounts of oxygen. Compound **3a** can also be prepared from **2a** by means of one-electron oxidation with ferrocenium or tritylium tetrafluoroborate in acetonitrile with 64.6% isolated yield (Scheme 1). In contrast, repeated attempts to synthesize **3a** directly from dipotassium salt **1** and Fe(III) chloride yielded complex mixtures of paramagnetic products with only small amounts of the target complex as indicated by ^1^H NMR spectroscopy, likely due to ligand oxidation and/or polymerization. The potassium cation in **3a** can be easily exchanged for a tetraphenylphosphonium by treatment with PPh_4_I in dichloromethane (DCM), yielding [(TSMP)_2_Fe^III^]PPh_4_ (**3b**). Furthermore, the potassium cation can also be complexed with two equivalents of B15C5 to form the [(TSMP)_2_Fe^III^][(B15C5)_2_K] adduct (**3c**).

**Figure 1:**
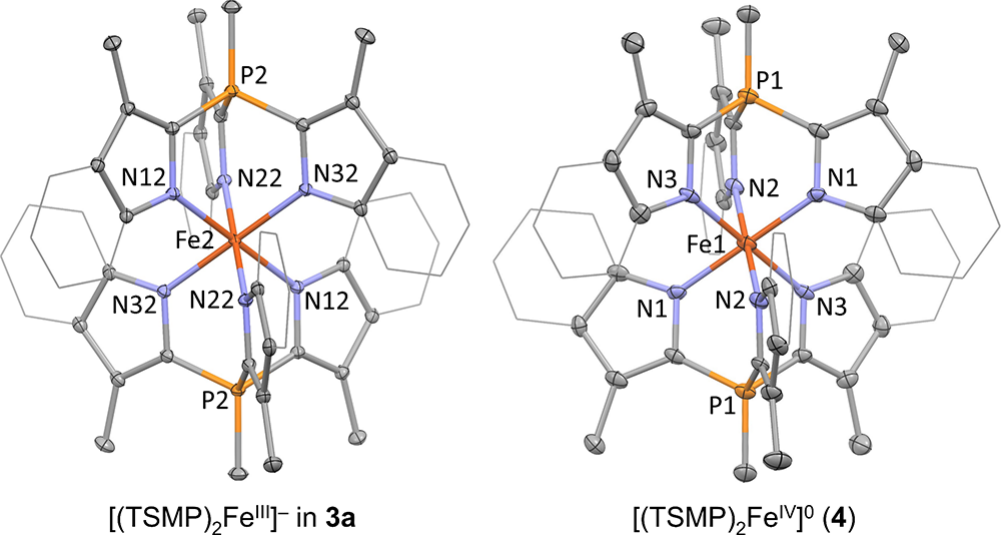
Molecular structure of complexes **3a** and **4** derived from single crystal X-ray diffraction. Displacement ellipsoids are drawn at 30% probability level. Fused benzene rings are shown in a wireframe style for clarity. Counterions, solvent molecules, and hydrogens are omitted for clarity. Selected bond distances (Å) and angles (degrees): **3a**: two molecular fragments in an asymmetric unit, fragment 1: Fe1–N11 1.9885(12), Fe1–N21 2.0019(12), Fe1–N31 1.9805(12), N11^∧^Fe1^∧^N21 91.11(5), N21^∧^Fe1^∧^N31 90.70(5), N31^∧^Fe1^∧^N11 90.73(5); fragment 2: Fe2–N12 1.9889(12), Fe2–N22 1.9885(12), Fe2–N32 1.9552(12), N12^∧^Fe2^∧^N22 91.56(5), N22^∧^Fe2^∧^N32 90.44(5), N32^∧^Fe2^∧^N12 91.16(5); **4**: molecule has C_*i*_ symmetry, Fe1–N1 1.966(5), Fe1–N2 1.975(6), Fe1–N3 1.966(6), N1^∧^Fe1^∧^N2 91.6(2), N2^∧^Fe1^∧^N3 91.0(2), N3^∧^Fe1^∧^N1 90.7(2).

The solid-state structure of **3a** was established by X-ray structure determination of single crystals grown from acetonitrile/ether. The compound crystallizes as one-dimensional coordination polymer with potassium atoms intercalated between aromatic rings of adjacent molecules of **3a** (Section S3). There are two independent iron centers in the structure, each located on a crystallographic inversion center. The first coordination sphere of the iron atom has approximate octahedral symmetry with N^∧^Fe^∧^N angles close to 90° and Fe–N bond lengths within 1.9552(12)–2.0020(12) Å (Figure 1) consistent with a low-spin (*S* = ^1^/_2_) state of the metal center.^41,42^ The solid-state structures of **3b** and **3c** are similar to that of **3a** (Section S3). Interestingly, the structure of **3c** also has two independent iron centers, each possessing exact inversion symmetry and an approximate octahedral environment, but with distinctly different spin states as can be seen from the two sets of Fe–N bond lengths. More specifically, the low-spin (*S* = ^1^/_2_) molecule features bonds within 1.970(2)–2.008(2) Å, while the high-spin (*S* = ^5^/_2_) unit has bonds within 2.123(3)–2.155(2) Å, with these ranges being typical for the assigned spin states.^41,42^ This discrepancy hints at the possibility of a spin cross-over, which is explored in more detail below.

^1^H NMR spectra of **3a** in acetonitrile-d_3_ solution within 233–348 K show only five paramagnetically shifted and broadened signals (Section S13.2), whereas six signals would be expected based on the *D*_3d_ symmetrical solution structure. The effective magnetic moment (μ_eff_) in solution measured by the Evans method in this temperature range varies from 3.50 to 4.84 μ_B_, indicating thermal spin cross-over (SCO) (vide infra). It suggests that the signal of the sixth proton, supposedly the closest one to the metal center, may be missing due to paramagnetic line broadening, which may be more pronounced at an increased μ_eff_ of the system. This hypothesis is confirmed by the ^1^H NMR spectrum of **3b** in DCM-d_2_ at 173 K, at which temperature most of the complex is in the low-spin *S* =  state as indicated by the fitted effective solution magnetic moment of 1.89 μ_B_ (vide infra). There, one can observe the sixth signal spanning over the range of >10 ppm (Figure S22).

Complex [(TSMP)_2_Fe^IV^] (**4**), for which the Fe(IV) oxidation state is demonstrated below, can be obtained as a bottle-green powder by one-electron oxidation of [(TSMP)_2_Fe^III^]PPh_4_ (**3b**) using elemental iodine in acetonitrile with 90.1% isolated yield. It is stable both in the solid state and solution for at least six months. Single crystals for X-ray structure determination were grown from pyridine/butyronitrile/ether and contain the electroneutral and roughly octahedral complex with Fe–N distances of 1.966(6)–1.975(5) Å (Figure 1). This is similar but still somewhat shorter on average than the distances in the isostructural anionic unit of **3a** (1.9552(12)–2.0020(12) Å). Solution ^1^H NMR spectra of [(TSMP)_2_Fe^IV^] (**4**) in DCM-d_2_ feature six paramagnetically shifted and broadened signals as expected for the *D*_3d_ symmetrical solution structure (vide infra). Very moderate solubility of **4** in all conventional NMR solvents precluded us from determining its effective solution magnetic moment by the Evans method. The results of magnetometry in the solid state are discussed below.

## Electrochemical Behavior

**Figure 2:**
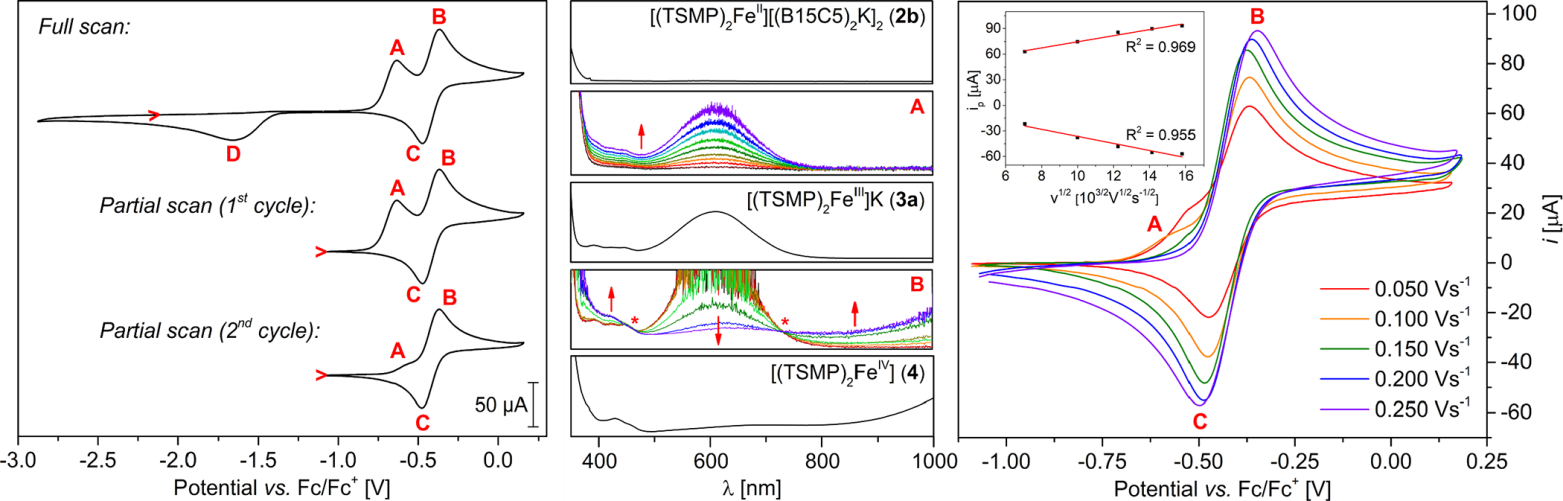
Cyclic voltammograms of compound **2b** (ca. 8 mM solution) in 0.1 M ^*n*^Bu_4_NPF_6_ acetonitrile electrolyte. Potentials are referenced with respect to the Fc/Fc^+^ redox couple. Left panel: overview scans at the rate of 100 mV/s; the full scan starts from an open-circuit potential of −2.12 V). Middle panel: UV–vis of independently synthesized compounds **2b**, **3a** (in MeCN), and **4** (in DCM) and time-dependent UV–vis spectra of controlled electrolysis at points **A** (−0.64 V) and **B** (−0.37 V) indicated on the CV on the left panel. Absorption in spectrum **B** was truncated due to detector saturation. The asterisks indicate isosbestic points that support a clean conversion between **3a** and **4**. Right panel: the quasi-reversible redox pair **A**–**C** centered at *E*_1/2_ = −0.41 V; the inset shows linear dependence of the peak current *vs* square root of the scan rate.

Cyclic voltammetry (CV) of [(TSMP)_2_Fe^II^][(B15C5)_2_K]_2_ (**2b**) reveals two couples of redox events: **A**/**D** and **B**/**C** (Figure 2, left panel). The full CV scan can be repeated at least a hundred times at rates within 50–250 mV/s with no visible changes, which demonstrates chemical reversibility of the redox cycle it represents. Controlled electrolysis at the points **A** and **B** of the CV using an optically transparent thin-layer electrochemical (OTTLE) cell^43^ (Figure 2, middle panel) allows the assignment of event **A** as the one-electron oxidation of [(TSMP)_2_Fe^II^]^2–^ (**2**) to [(TSMP)_2_Fe^III^]^−^ (**3**), whereas event **B** corresponds to the oxidation of [(TSMP)_2_Fe^III^]^−^ (**3**) to [(TSMP)_2_Fe^IV^] (**4**). Correspondingly, their reductive counterparts **D** and **C** can be assigned to the same respective processes but in reverse. Importantly, the independently measured CV of [(TSMP)_2_Fe^III^]K (**3a**) is very similar to that of **2b** (Section S5.2), which confirms our assignments.

The pair of redox events **A**/**D** is characterized by a large peak separation (Δ*E*_p_ of 1030 mV at 100 mV/s) and a large full width at half maximum (FWHM) of feature **D** (>500 mV). These are clear indications of an irreversible electron transfer, suggesting that a substantial reorganization energy for the [(TSMP)_2_Fe^II^]^2–^ (**2**)/[(TSMP)_2_Fe^III^]^−^ (**3**) redox couple arises from the different geometries of tetrahedral^40^**2** and octahedral **3**.

The pair of events **B**/**C** can be isolated by means of partial CV scans not involving feature **D** (Figure 2, left panel). During the first cycle, all [(TSMP)_2_Fe^II^]^2–^ (**2**) complex in the electrode diffusion layer is consumed to form [(TSMP)_2_Fe^III^]^−^ (**3**) (event **A**), followed by oxidation to [(TSMP)_2_Fe^IV^] (**4**; event **B**) with its subsequent reduction back to [(TSMP)_2_Fe^III^]^−^ (**3**) (event **C**). Since the potential scan window does not involve feature **D**, corresponding to regeneration of the initial [(TSMP)_2_Fe^II^]^2–^ (**2**) ions, by the start of the second cycle, the electrode diffusion layer is depleted of the latter, as indicated by the reduced intensity of feature **A**, as shown in Figure 2, left panel. The residual intensity is likely due to diffusion of ions of **2** from the outer pool of the [(TSMP)_2_Fe^II^][(B15C5)_2_K]_2_ (**2b**) complex. This interpretation is confirmed by varying the potential scanning rate (Figure 2, right panel): slower scans result in increased intensity of **A**, while the faster ones lead to its complete disappearance.

As for the pair of events **B**/**C** itself, it represents a redox process centered at *E*_1/2_ = −0.41 V with respect to the Fc/Fc^+^ couple, which lies within the typical range between −1.24 and – 0.03 V for other published Fe(III)/Fe(IV) couples.^11,19,29,44,45^ Its Δ*E*_p_ varies with potential scan rate from 110 mV at 50 mV/s to 152 mV at 250 mV/s, while the peak current changes linearly with the square root of the scan rate. These are diagnostic criteria for a quasi-reversible electron transfer,^46^ implying that the reorganization energy of a transition between [(TSMP)_2_Fe^III^]^−^ (**3**) and [(TSMP)_2_Fe^IV^] (**4**) states is rather small, consistently with the similar solid-state geometries of the complexes [(TSMP)_2_Fe^III^]K (**3a**) and [(TSMP)_2_Fe^IV^] (**4**) (Figure 1).

## SCO in the [(TSMP)_2_Fe^III^]^−^ Complex (**3**)

**Figure 3:**
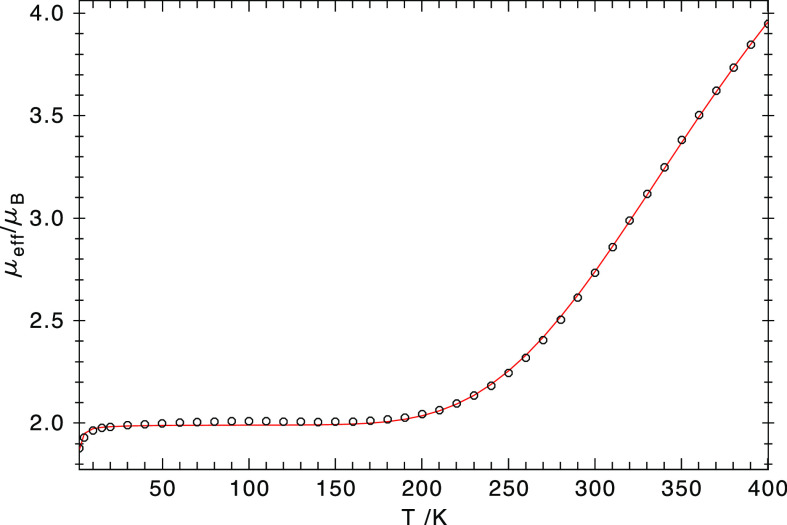
SQUID measurement of **3a** under an applied magnetic field of 1 T. The dots represent experimental data, and the solid red line the fit with the following parameters: *S* = ^1^/_2_, *g*_iso_ = 2.30, *zJ* = −6 cm^–1^; *S* = ^5^/_2_, *D* = *E* = 0, *g*_iso_ = 2.00, and TIP = 0.

To probe the electronic structure of [(TSMP)_2_Fe^III^]K (**3a**), we measured the effective magnetic moment (μ_eff_) of a powder sample over a temperature range of 2–400 K with a SQUID. As shown in Figure 3, from 10 to 180 K, μ_eff_ is nearly constant at 2.02 ± 0.01 μ_B_. This value is considerably higher than the spin-only value of 1.73 μ_B_ that is expected for *S* = ^1^/_2_ complexes, which reflects **3** having appreciable unquenched orbital angular momentum, typical of low-spin ferric complexes.^47^ Starting from 180 K, μ_eff_ increases with temperature and does not get saturated even at 400 K. These experimental findings show that over the course of the SQUID measurement, **3a** undergoes a thermally activated SCO from the *S* = ^1^/_2_ ground state to an *S* = ^5^/_2_ state. Of note, because the unsaturated μ_eff_ value at 400 K of 3.98 μ_B_ exceeds the spin-only value of the quartet *S* = ^3^/_2_ state (3.87 μ_B_), the second spin state cannot be ^3^/_2_ but is rather ^5^/_2_, anticipated for the distorted octahedral coordination geometry of **3**. The variation of μ_eff_ was fitted with the domain model of Sorai and Seki,^48^ and the satisfactory simulations yielded *T*_c_ = 450 ± 5 K and *n*Δ*H* = 15.4 ± 0.2 kJ/mol. Here, *T*_c_ represents the critical transition temperature of the SCO, Δ*H = H*_HS_ – *H*_LS_*—* the enthalpy difference between the high-state and low-spin state, and *n —* the number of molecules per domain. Below 10 K, the precipitous drop of μ_eff_ cannot be simply interpreted as field saturation but primarily arises from weak intermolecular interactions, for which a mean field model^49^ was invoked to fit the SQUID data and gave *zJ* = −6 ± 1 cm^–1^.

**Figure 4:**
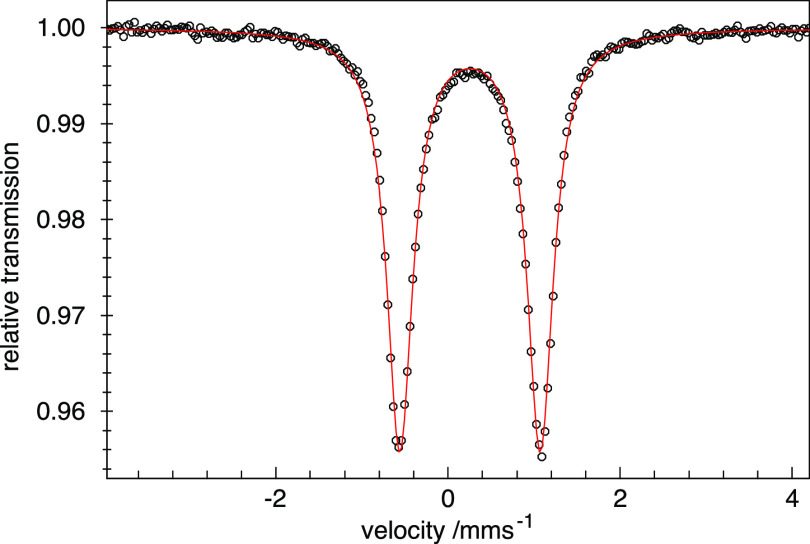
Zero-field Mössbauer spectrum of **3a** measured on a powder sample at 80 K. The fitted parameters are δ = 0.25 mm/s and |Δ*E*_Q_| = 1.63 mm/s.

The zero-field Mössbauer spectrum (Figure 4) of complex **3a** recorded at 80 K displays a well-resolved quadrupole doublet at isotope shift δ = 0.25 mm/s and quadrupole splitting |Δ*E*_Q_| = 1.63 mm/s. Both values are characteristic for low-spin ferric centers coordinated by six hard nitrogen donors. DFT calculations performed on both independent anions in the crystal structure of **3a** predict isomer shifts (δ = 0.29 and 0.26 mm/s) and quadrupole splittings (|Δ*E*_Q_| = 2.19 and 2.18 mm/s) in reasonable agreement with the experiment (see ESI Section S11.1 for details). Furthermore, no other iron species was identified in the spectrum, consistent with the high SCO critical temperature *T*_c_ of 450 K.

Interestingly, SQUID magnetometry of the benzo-15-crown-5 adduct [(TSMP)_2_Fe^III^][(B15C5)_2_K] (**3c**) reveals that at low temperatures, the system has a magnetic moment μ_eff_ of 3.61 μ_B_, in between the low- and high-spin states, similarly to 3.88 μ_B_ for the case of an intermediate-spin (*S* = ^3^/_2_). The system crosses over toward the high-spin state at only ca. 140 K (Section S8.2). However, an *S* = ^3^/_2_ electronic configuration is unlikely for a [(TSMP)_2_Fe^III^]^−^ (**3**) ion since it would imply significant distortion of the FeN_6_ environment, leading to strain in the TSMP scaffold, which is expected to be more energetic than the energy gap between the *S* = ^3^/_2_ and other spin states. Indeed, X-ray diffraction on a single crystal of **3c** grown from pyridine/*n*-hexane (Section S3) shows that at 100 K, it exists as a 1:1 mixture of the low-spin and high-spin components with two distinct sets of Fe–N bonds typical for the assigned spin states:^42,50,51^ 1.970(2)–2.008(2) and 2.123(3)–2.155(2) Å, respectively. Although rare for iron(III) coordination compounds, such behavior is not unprecedented and was observed for some tris(dithiocarbamato)^50^ complexes.

**Figure 5:**
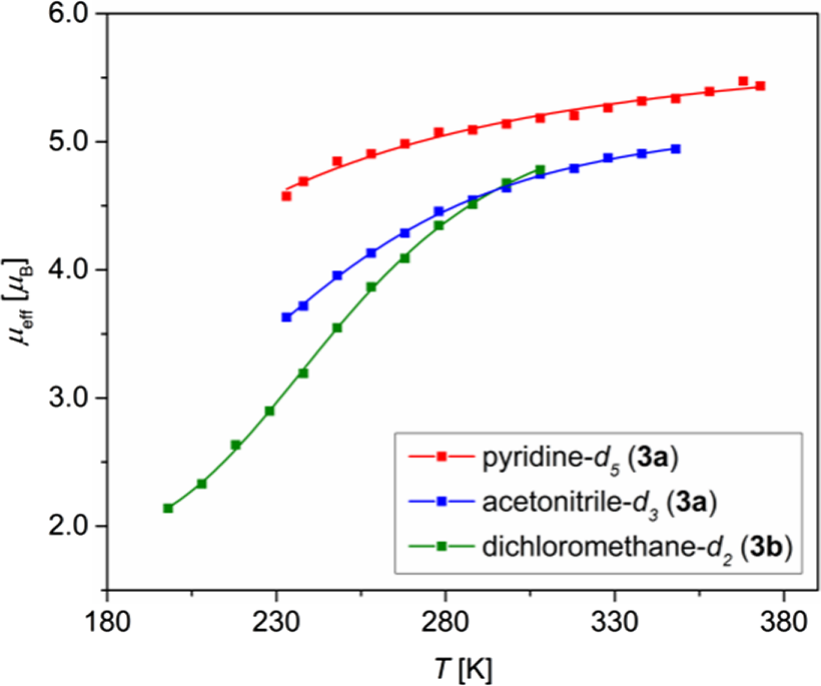
SCO curve for the [(TSMP)_2_Fe^III^]^−^ (**3**) complex as obtained by the Evans method in different solvents. Data points represent experimental measurements, whereas smooth curves are fits based on the regressive model in eq 1. Explored temperature ranges are limited by the freezing and boiling points of the respective solvents or precipitation of the compound at low temperatures.

The solid-state behavior of **3** parallels our observations in solution. The SCO between the *S* = ^1^/_2_ and ^5^/_2_ states is evident from the strongly temperature-dependent μ_eff_, as measured by Evans method.^52−54^ The results of such measurements are shown in Figure 5 for potassium salt **3a** in acetonitrile-d_3_ and pyridine-d_5_, and for tetraphenylphosphonium salt **3b** in DCM-d_2_ as **3a** is insoluble in this solvent. The change of the effective solution magnetic moment with temperature is also apparent from the paramagnetic ^1^H NMR shifts and line broadening, which are analyzed in more detail in ESI Section S13.2.2.

The solution SCO behavior can be fitted using eq 1 (Section S6), which allows to extract the enthalpy (Δ*H*) and entropy (Δ*S*) of the cross-over as well as the limiting magnetic moments for both low- and high-spin states, μ_LS_ and μ_HS_, respectively.
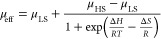
1where *T* is temperature and *R* is the universal gas constant.

The cross-over curve for **3b** in DCM-d_2_ is the most informative since it spans through both low- and high-μ_eff_ regions. A regressive thermodynamic analysis (Section S6.2) provides the following parameters: Δ*H* and Δ*S* of 18.1 ± 1.4 kJ/mol and 72.6 ± 5.8 J/(mol·K), respectively, with the critical temperature Δ*H/*Δ*S = T*_c_ of 249 ± 1 K. The limiting magnetic moments μ_LS_ and μ_HS_ are 1.81 ± 0.07 and 5.35 ± 0.10 μ_B_, respectively, which is close to the spin-only expectation values for the low-spin *S* = ^1^/_2_ (1.73 μ_B_) and high-spin *S* = ^5^/_2_ (5.92 μ_B_) states. A similar analysis for the SCO of **3a** in acetonitrile-d_3_ leads to slightly different values with higher standard errors (Section S6.2) Δ*H* of 14.4 ± 2.4 kJ/mol, Δ*S* of 59.6 ± 8.0 J/(mol·K) and *T*_c_ of 240 ± 9 K. Because the SCO curve does not cover the low-μ_eff_ range, a significant standard error is also associated with the lower limiting value μ_LS_ of 2.35 ± 0.45 μ_B_, while the higher one of 5.23 ± 0.08 μ_B_ has smaller uncertainty and is similar to that for **3b**. Lastly, since **3a** in pyridine-d_5_ has almost completely undergone SCO at the lowest accessible temperature (Figure 5), the corresponding thermodynamic parameters cannot be reliably extracted. Overall, these data indicate that the SCO thermodynamics of **3** is sensitive to solvent and/or counterion, possibly indicating a role of ion pairing in solution.

While the difference of ∼200 K in SCO critical temperatures (*T*_c_) in the solid state and in solution is very high, in some cases, the crystal packing is known to lock some SCO molecules in a fixed spin state,^55^ slow down the spin transition by anticooperative effects,^56,57^ or even prevent SCO from happening at all.^58^

To the best of our knowledge, **3** is the first synthetic complex with an Fe^III^N_6_ core that undergoes thermal SCO. This behavior is likely due to TSMP^2–^ being a relatively weak-field ligand with poor π-accepting properties caused by the presence of low-lying π-orbitals in the extended aromatic systems. Gradual transitions as observed for **3** in the solid state and in solution are commonly observed for SCO in Fe(III) compounds.^41^ A broad range of critical temperatures *T*_c_ (20 K–ca. 400 K) have been observed for Fe(III) compounds with different ligand sets.^42^ The pronounced difference between **3a** and **3c** and between solid-state and solution suggests that SCO in anion **3** is strongly sensitive to its environment.

## Electronic Structure of [(TSMP)_2_Fe^IV^] (**4**)

The presence of 10e̅ conjugated π-systems on the ligand raises the question of the actual electronic structure and metal oxidation state in **4**, which we address by the following electrochemical, spectroscopic, and computational studies.

### Cyclic Voltammetry

**Figure 6:**
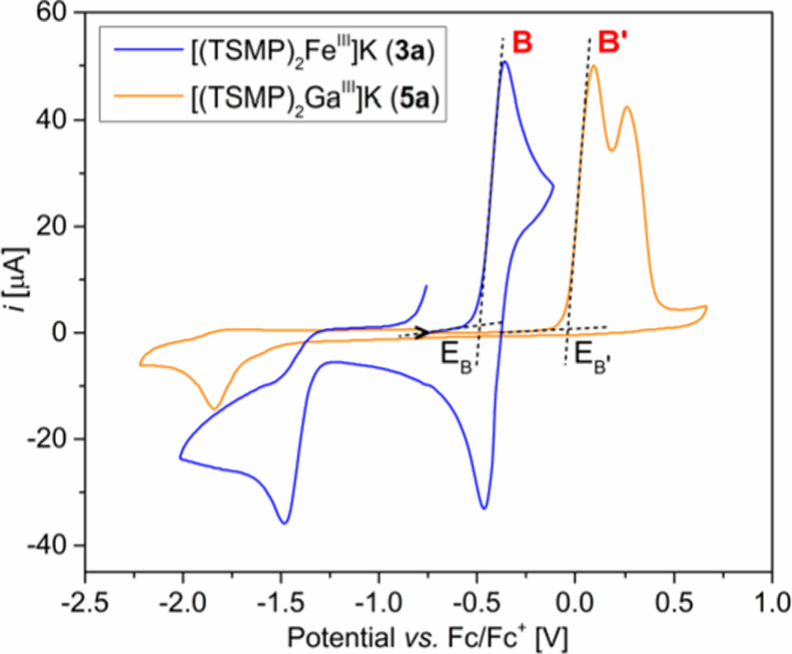
Overlay of CVs measured for **3a** and **5a**. The measurements were performed in ca. 8 mM solution in 0.1 M ^*n*^Bu_4_NPF_6_ acetonitrile electrolyte. Potentials are referenced with respect to the Fc^+^/Fc redox couple. The onsets of oxidation of **3a** and **5a** (*E*_B_ and *E*_B′_, respectively) are defined as the points of intersection between extrapolated baseline and a tangent to the oxidation feature **B**/**B′**.

A first insight is provided by comparing cyclic voltammograms of [(TSMP)_2_Fe^III^]K (**3a**) and its isostructural Ga analogue [(TSMP)_2_Ga^III^]K (**5a**) (Figure 6) synthesized using TSMPK_2_ salt (**1**) and GaCl_3_ in acetonitrile (Section S2.1; X-ray crystallographic details in Section S3). If the oxidation event leading from **3a** to **4** was mostly ligand-centered, one would expect a small first oxidation potential difference compared with **5a**. In contrast, the observed significant difference of 450 mV argues in favor of at least partially metal-centered oxidation of iron in **4**.

### ^57^Fe Mössbauer Spectroscopy

**Figure 7:**
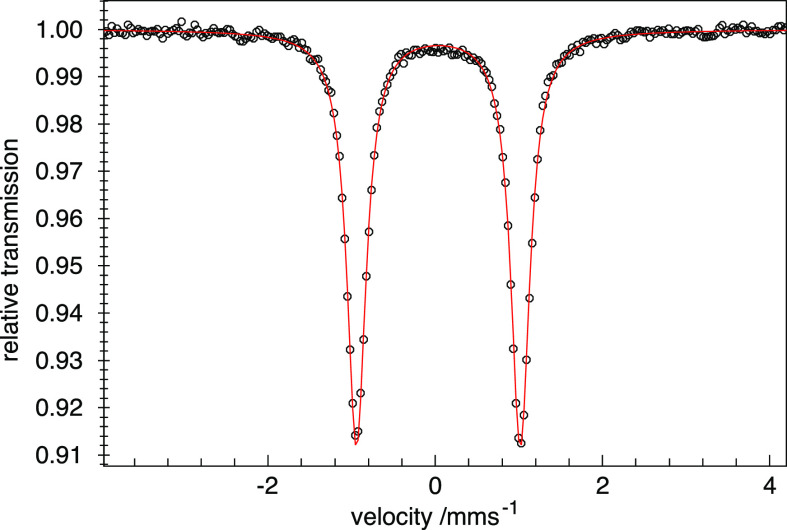
Zero-field Mössbauer spectrum of **4** measured on a powder sample at 80 K. The fitted parameters are δ = 0.04 mm/s and |Δ*E*_Q_| = 1.96 mm/s.

The electronic structure of the [(TSMP)_2_Fe^IV^] (**4**) complex was further probed by zero-field ^57^Fe Mössbauer spectroscopy at 80 K. The spectrum (Figure 7) shows a very clear quadrupole doublet. The isomer shift (δ) of 0.04 mm/s is substantially lower than 0.25 mm/s measured for the parent Fe(III) compound **3a** (Figure 4) and falls within the expected range for a Fe(IV) metal center,^59^ clearly indicating metal-centered oxidation. The quadrupole splitting |Δ*E*_Q_| of 1.96 mm/s suggests a significant deviation of the electronic configuration from the cubic symmetry. The measured isomer shift is similar to that of **E**(29) (0.045 mm/s) in Chart 1 but different from that of **F**(30) (−0.23 mm/s), as expected for their distinct donors (N^–^ vs C). At the same time, the measured quadrupole splitting is lower than 2.51 mm/s in **E** and 3.04 mm/s in **F**, likely reflecting their slightly different local Fe coordination environments.

### XAS Spectroscopy

**Figure 8:**
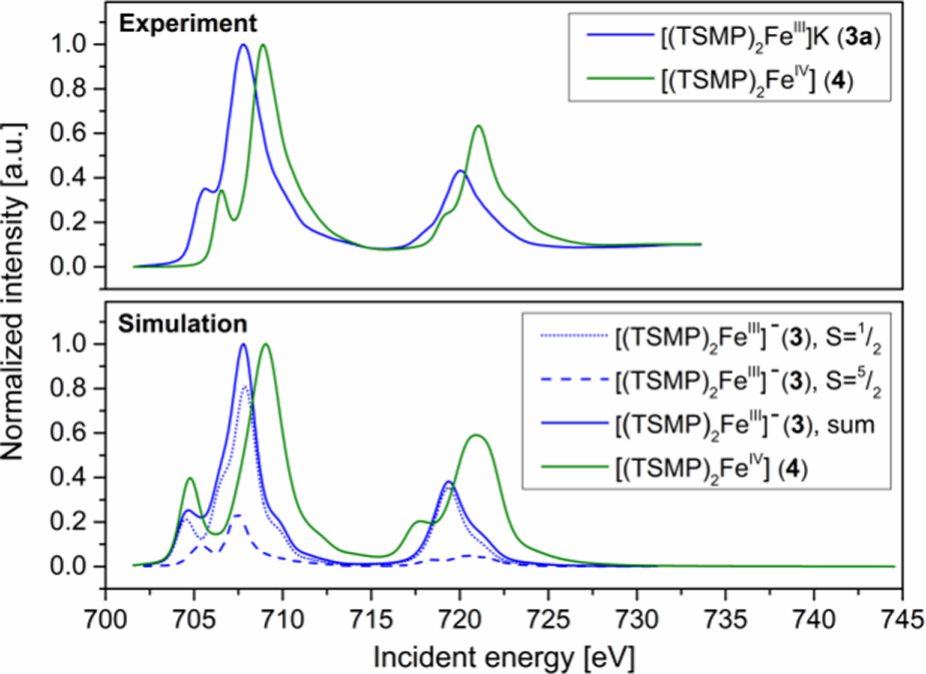
Experimental (top panel) and simulated (bottom panel) L_2,3_ edge XAS spectra of the Fe(III) (**3a**/**3**) and Fe(IV) (**4**) complexes.

Our Fe 2p (L_2,3_) X-ray absorption (XAS) measurements generally agree with the conclusions drawn from the cyclic voltammetry and Mössbauer spectroscopy above. In XAS, a 2p core electron is excited into an empty 3d state, which for a 3d^5^ iron center in [(TSMP)_2_Fe^III^]^−^ (**3**) leads to a 2p^5^3d^6^ configuration. The experimental XAS spectra of the potassium salt **3a** agree with simulations for anion **3** based on ligand field multiplet theory (Figure 8).^60^ Given the average excitation lifetime of <1 fs, the spectrum of **3a** at 300 K is adequately simulated as a sum of low- and high-spin components in a ratio of 80:20, which is consistent with the solid-state SCO behavior discussed above.

The experimental XAS spectrum of [(TSMP)_2_Fe^IV^] (**4**) is similar but shifted to higher energies by 0.9–1.1 eV compared to **3a** (Figure 8, top panel), indicating metal-centered oxidation. This spectrum can be simulated (Figure 8, bottom panel) considering *D*_3d_ symmetry of **4** with 1a_1_–1e–2e orbital splitting (see Section VI below), 2-2-0 occupancies, and a ^3^A_2_ ground state.^60^ In the simulation, the two biggest peaks can be approximated as transitions into the 1e and 2e orbitals. However, due to strong 2p3d multiplet effects, this assignment is not completely accurate as significant mixing occurs in the final 2p^5^3d^5^ state. The shoulder at 710 eV in the experimental spectrum is due to charge transfer, which was not included explicitly in the calculations to limit the number of parameters. These charge transfer excitations were omitted due to the use of the nephelauxetic effect along with reduced electron–electron interactions (factor of 0.85).^61^ An overview of the exact parameters used in the calculation is given in ESI Section S7.2.

Taken together, the 2p XAS spectra confirm the nature of the [(TSMP)_2_Fe^III^]^−^ (**3**) and [(TSMP)_2_Fe^IV^] (**4**) systems. At 300 K, **3a** has predominantly a low-spin configuration, while **4** has an ^3^A_2_ triplet ground state with a doubly occupied d_*z*^2^_orbital and a half-filled first e(*D*_3d_) state (vide infra, Figure 11).

### SQUID Magnetometry

**Figure 9:**
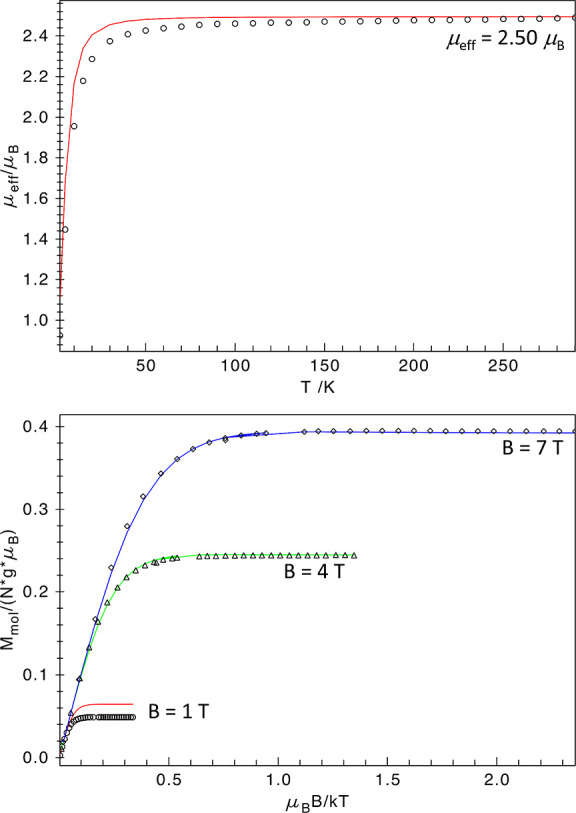
VT μ_eff_ at 0.1 T (top) and VTVH magnetization at 1, 4, and 7 T (bottom) SQUID measurements of **4**, fitted using the following spin Hamiltonian parameters: *g*_iso_ = 1.76, *D* = +15 cm^–1^, *E*/*D* = 0.

SQUID magnetometry was performed on a microcrystalline sample of **4** in order to determine its spin state and assess its zero-field splitting (ZFS). The fit of the VT measurement (Figure 9, top panel) gives a μ_eff_ value of 2.50 μ_B_ at room temperature, which is lower but still close to the spin-only expectation value for an intermediate-spin state (*S* = 1) Fe(IV) center (2.83 μ_B_). The corresponding *g*_iso_-value is 1.76. The magnetic susceptibility from the VTVH measurement (Figure 9, bottom panel) could be fitted consistently with an axial ZFS parameter *D* of +15 cm^–1^. The rhombicity *E*/*D* was taken as zero due to the C_3_ axis of symmetry in the molecule. However, these parameters gave a subpar fit for the measurement at 1 T. We attribute this to small antiferromagnetic intermolecular interactions which compete with the Zeeman effect under low external field (1 T) and become negligible under higher fields (4 and 7 T). These interactions could be caused by the presence of small amounts of paramagnetic impurities or interactions of the sample molecules with one another.

Overall, the abnormally low effective magnetic moment (corresponding to the unphysically small *g*_iso_) and small intermolecular interactions suggest that the measured sample of **4** contains some admixtures, be it paramagnetic impurities or residual solvent. The latter is likely due to the zwitterionic nature of **4**, which makes complete solvent removal very difficult even after prolonged drying in vacuo. However, the SQUID measurements still allow to get a qualitative estimate of the zero-field splitting (*D* ≈ +15 cm^–1^), as well as a clear determination of *S* = 1 spin state (^3^A_2_ ground state).

### THz-EPR Spectroscopy

A more direct way to quantify *g*-values and ZFS parameters of **4**, which does not require the mass of the sample to be precisely known, is through EPR spectroscopy. However, as **4** is an integer-spin (*S* = 1) system with low rhombicity and an intermediate, positive *D*-value, the transitions between the *m*_S_ = 0 sublevel and the non-Kramers doublet *m*_S_ = ±1 are too energetic to be observed using conventional EPR spectrometers.^62^ Indeed, X-band measurements on the microcrystalline samples gave no interpretable signal; featureless spectra were also obtained in parallel mode. Therefore, we resorted to high-energy (THz range) frequency-domain Fourier-transform THz-EPR spectroscopy (THz-EPR in short).^63^

**Figure 10:**
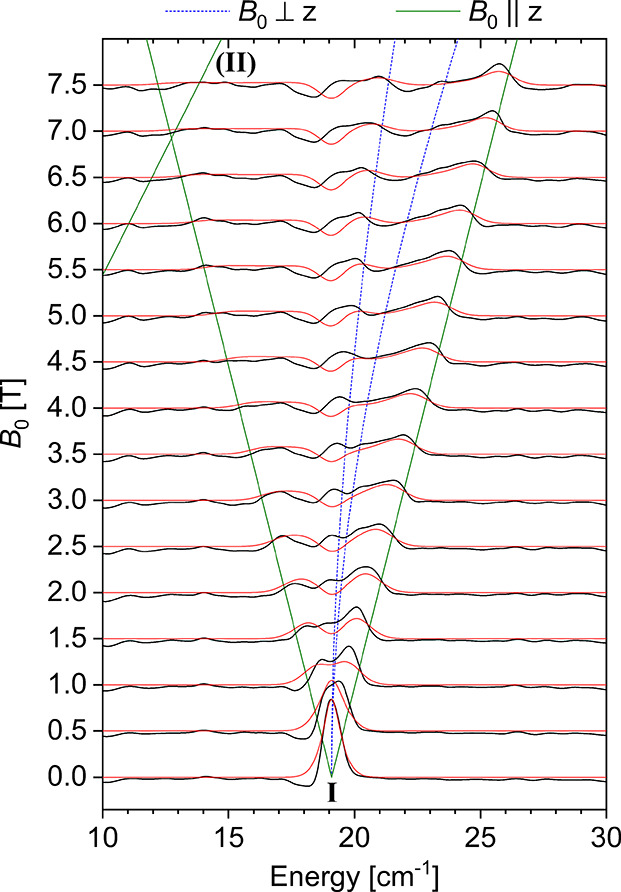
THz-EPR spectra of **4**. Relative absorbance spectra (black lines) are offset for the magnetic field *B*_0_ at which they were measured. Simulations using *D* = +19.1 cm^–1^, *E* = 0, and an isotropic *g*-value of 1.97 are shown in red. Calculated transition energies for magnetic fields applied parallel and perpendicular to the main anisotropy axis (*z*) are shown as solid green and dashed blue lines, respectively. Branch **II** corresponds to the formally forbidden transitions between the excited *m*_S_ = ±1 sublevels.

Variable-field THz-EPR spectra of **4** in Figure 10 are shown in relative absorbance as , where *I*_ref_ is a reference transmittance spectrum measured at 31 K and 0 T, and *I*(*B*_0_) is a transmittance spectrum measured at 4.8 K and magnetic field *B*_0_ (Figure S14). For *B*_0_ = 0, a clear absorption(**I**) can be observed at 19.1 cm^–1^. This feature broadens and splits with increasing external magnetic field. The field dependence allows for assignment of the peak at 19.1 cm^–1^ to the EPR transition from *m*_S_ = 0 to the *m*_S_ = ±1 sublevels of the *S* = 1 system (Section S9.2). Thus, the zero-field spectrum corresponds to an axial ZFS of *D* = 19.1 cm^–1^ with vanishing rhombicity (*E* ≈ 0) due to the lack of a visible splitting in the zero-field spectrum. From the linewidth of the signal, *E* ≤ 0.3 cm^–1^ can be estimated. Simulations using *D* = 19.1 cm^–1^, *E* = 0 and an isotropic *g*-value of 1.97 reproduce the zero-field transition energy as well as the field dependence very well (Figure 10). We note that the spectra feature a lowering of the relative absorbance around 18.4 cm^–1^, directly below the ZFS energy, which is discussed in Section S9.4. Further details of the THz-EPR measurement protocol and simulations, including demonstration of the unfeasibility of alternative EPR parameter sets, are discussed in Section S9.

### DFT Calculations

Geometries of the alternative spin states of **4** (*S* = 0, 1, 2) optimized at the BP86-D3BJ/def2-TZVP level of theory show that the *S* = 1 state is the most energetically favored. Its SCF energy is lower than that of the *S* = 0 and *S* = 2 states by 17.8 and 25.0 kcal/mol, respectively. Mössbauer spectral parameters calculated for all three geometries (B3LYP-D3BJ/def2-TZVP level of theory, CP(PPP) basis set for Fe) also support the assignment of the *S* = 1 spin state (Section S11.2) as derived from SQUID and THz-EPR measurements. More specifically, the calculated isomer shift (δ) of −0.05 mm/s is close to the experimental value of +0.04 mm/s within the uncertainty of computations.^64^ The calculated quadrupole splitting (Δ*E*_Q_) of −2.01 mm/s is also very close to the absolute experimental value of 1.96 mm/s, while the sign of the splitting cannot be inferred from the zero-field measurements.

**Figure 11:**
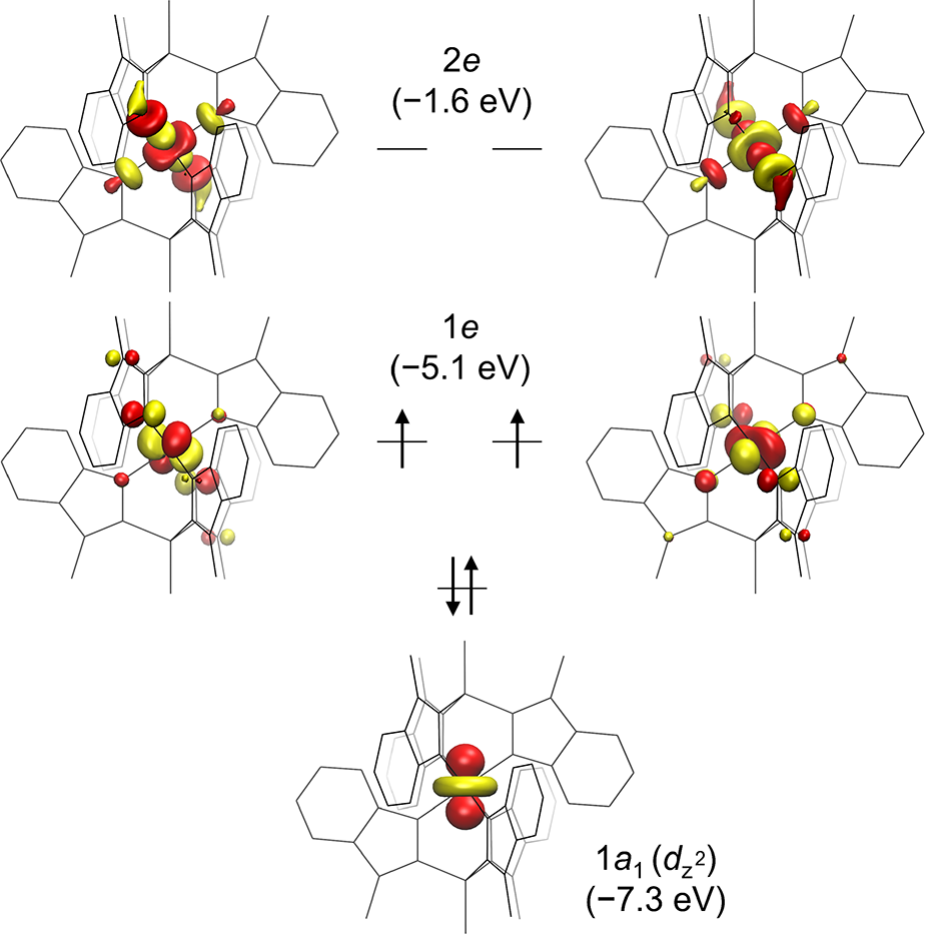
Quasi-restricted frontier orbitals (isocontour = 0.05) of the ground state of **4** calculated at the B3LYP-D3BJ/def2-TZVP (CP(PPP) for Fe) level of theory using a geometry optimized at the BP86-D3BJ/def2-TZVP level. Orbital energies are given in parentheses.

Given that the experimental Mössbauer parameters are well reproduced by the calculations for the *S* = 1 state, the computed electron density can be used for closer examination of the electronic structure of **4**. Despite the optimized geometry featuring a locally octahedral FeN_6_ center, the molecule of **4** itself has *D*_3d_ symmetry with a threefold rotation axis passing through the P–Fe–P atoms. This leads to orbital splitting typical for this kind of symmetry (Figure 11). The doubly occupied 1a_1_ orbital has almost entirely d_*z*^2^_ character, which can be rationalized based on the irreducible representations of d-orbitals in a *D*_3d_ point group. More specifically, the d_*z*^2^_ belongs to the representation *A*_1_ and therefore cannot mix with the four remaining d-orbitals, (d_*xy*_, d_*x*^2^ – *y*^2^_) and (d_*xz*_, d_*yz*_), belonging to the double representation *E*. At the same time, these four orbitals mix together, forming two degenerate pairs: 1e and 2e (Figure 11). Both these pairs bear an anti-bonding character: 1e along the π-manifold and 2e along the σ-manifold, leaving 1a_1_ to be the only nonbonding d-orbital. The fact that the 1e pair of SOMOs is primarily localized on the metal center implies that this is also where most of the spin density can be found (Section S11.3).

Considering the discussed orbital manifold, the negative sign of the calculated Mössbauer quadrupole splitting (Δ*E*_Q_= −2.01 mm/s) is dominated by the strongly negative valence contribution of the 1a_1_ orbital. Note that while the 1e SOMOs influence the quadrupole splitting as well, the positive contribution from the d_*xy*_ and d_*x*^2^ – *y*^2^_ orbitals is counterbalanced by the negative one from d_*xz*_ and d_*yz*_. A detailed analysis of the origin of the quadrupole splitting is given in the ESI (Section S10.2).

**Chart 2:**
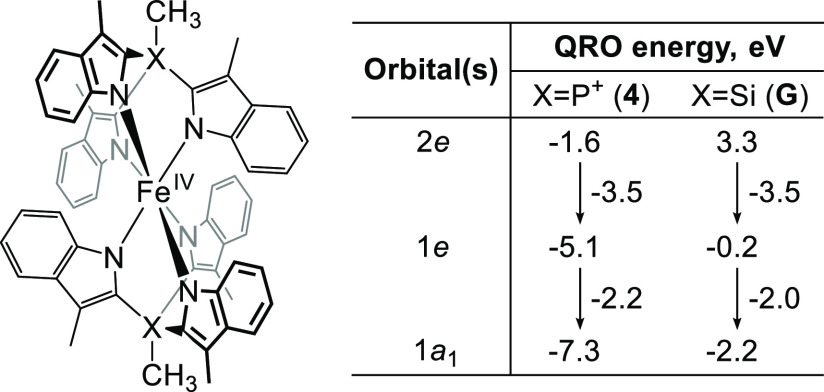
Comparison of the Quasi-Restricted Orbital (QRO) Energies of **4** and Its Si-tethered Analogue **G** Calculated at the B3LYP-D3BJ/def2-TZVP (CP(PPP) for Fe) Level of Theory for a Geometry Optimized at the BP86-D3BJ/def2-TZVP Level

It is important to note that the doubly occupied 1a_1_ orbital in Figure 11 points directly at the positively charged phosphonium atoms, which is expected to lower its energy by providing additional electrostatic stabilization. Indeed, comparison of the quasi-restricted orbital energies of **4** and its isoelectronic Si-tethered analogue (**G** in Chart 2) calculated at the same level of theory reveals that, while the 1e–2e gap is the same in both molecules, the 1a_1_ orbital in **4** is stabilized by an additional 0.2 eV. Given the close similarity of the molecular geometries of **4** and **G** (Section S11.4), this difference is most likely due to the electrostatic effects, although subtle influence of the bridgehead atom on π-donating ability of a ligand cannot be excluded.

### CASSCF Calculations

To gain more insight into the magnetic properties of **4**, its excited states were calculated using CASSCF. Because only the d–d excitations are significantly relevant for the ZFS and *g*-tensor calculations, the active space CAS(4,5) was restricted to only the metal-based d-orbitals. The energies were corrected with strongly-contracted NEVPT2 to recover dynamical correlation.

The results are consistent with the orbital picture given by DFT (Figure 11). They show large splitting between the doubly and singly occupied 1a_1_ (−11.2 eV) and 1e (−9.5 eV) orbitals and the unoccupied orbital pair 2e (−0.9 eV). The first and second excited states correspond to a single d–d excitation from the doubly occupied nonbonding orbital 1a_1_ to the π-antibonding pair 1e, and lie at ca. 10850 cm^–1^ above the ground state. The excitation from the 1a_1_ orbital to the highly σ-antibonding orbital pair 2e is 19000 cm^–1^ higher than the ground state (Section S12).

The ZFS and the *g*-tensor were calculated by the effective Hamiltonian theory (for technical details, see Section S12). The principal axes of the *g*- and *D*-tensor coincides with the P–Fe–P C_3_-axis of rotation. The calculated *D*-value is +15.3 cm^–1^, while the rhombicity is zero, consistently with the axial symmetry of the system. The calculated *g*-tensor exhibits only small anisotropy (*g*_∥_ = 2.00, *g*_⊥_ = 2.04, *g*_iso_ = 2.03). These parameters are in a good agreement with the data obtained from the SQUID and THz-EPR measurements (vide supra): *D* = +19.1 cm^–1^, *E* ≤ 0.3 cm^–1^, and *g*_iso_ of 1.97. The moderate ZFS and fairly isotropic *g*-value are not surprising since the lowest-energy d–d excited state, which is also spin-conserving, lies 10800 cm^–1^ above the ground state, which is more than 20 times larger than the effective spin-orbit coupling constant of the Fe(IV) ion (515 cm^–1^).^65^ Hence, the ground state is fairly isolated and the spin-orbit coupling effect represents a moderate perturbation.

### Paramagnetic ^1^H NMR Spectroscopy

**Figure 12:**
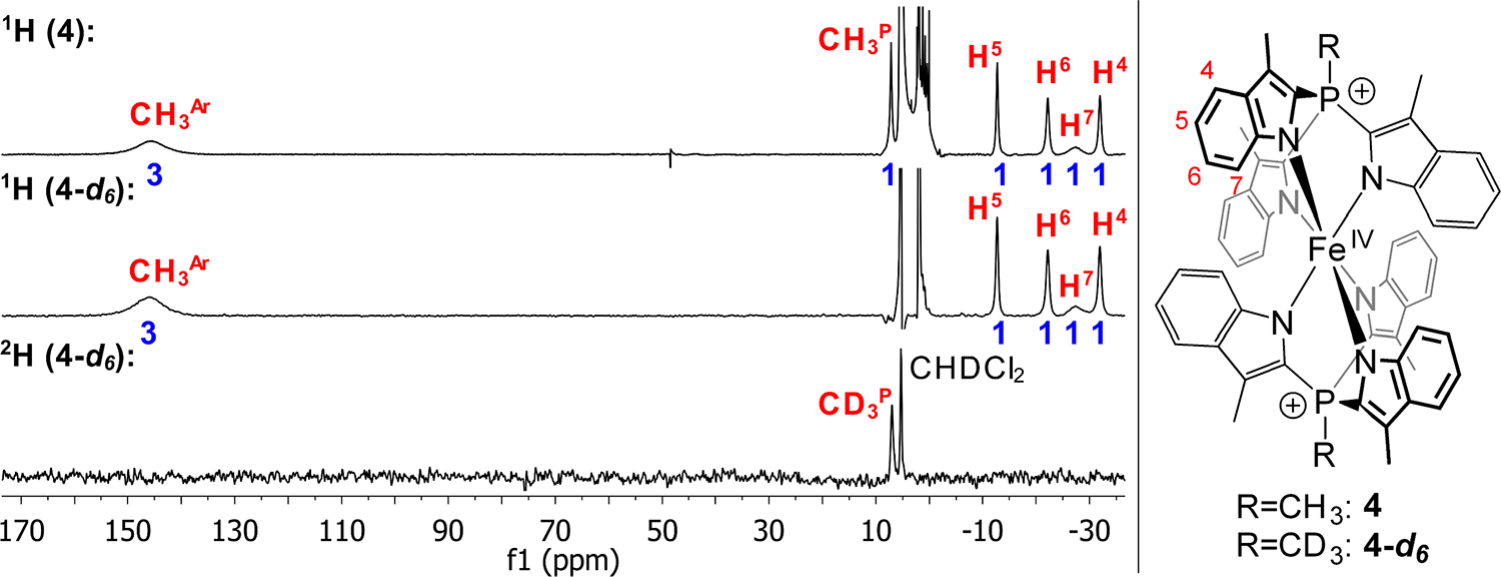
^1^H (400 MHz) and ^2^H (61 MHz) NMR spectra of **4** and its deuterated analogue **4-d_6_** in DCM at 298 K. Only paramagnetic signals are assigned. The integrals are given in blue and were rounded to the nearest integer.

Despite the moderate solubility of **4** in conventional solvents, we were able to acquire its ^1^H NMR spectra in DCM-d_2_ (Figure 12). The complex shows six paramagnetically shifted and broadened signals, as expected for the *D*_3d_ topology in solution. The signals were assigned based on their integral intensity, linewidth and by comparing the spectrum with that of a deuterated (^2^H_3_)methylphosphonium analogue, **4-d_6_** (Sections S13.1 and S13.3.1), which was synthesized independently.

**Figure 13:**
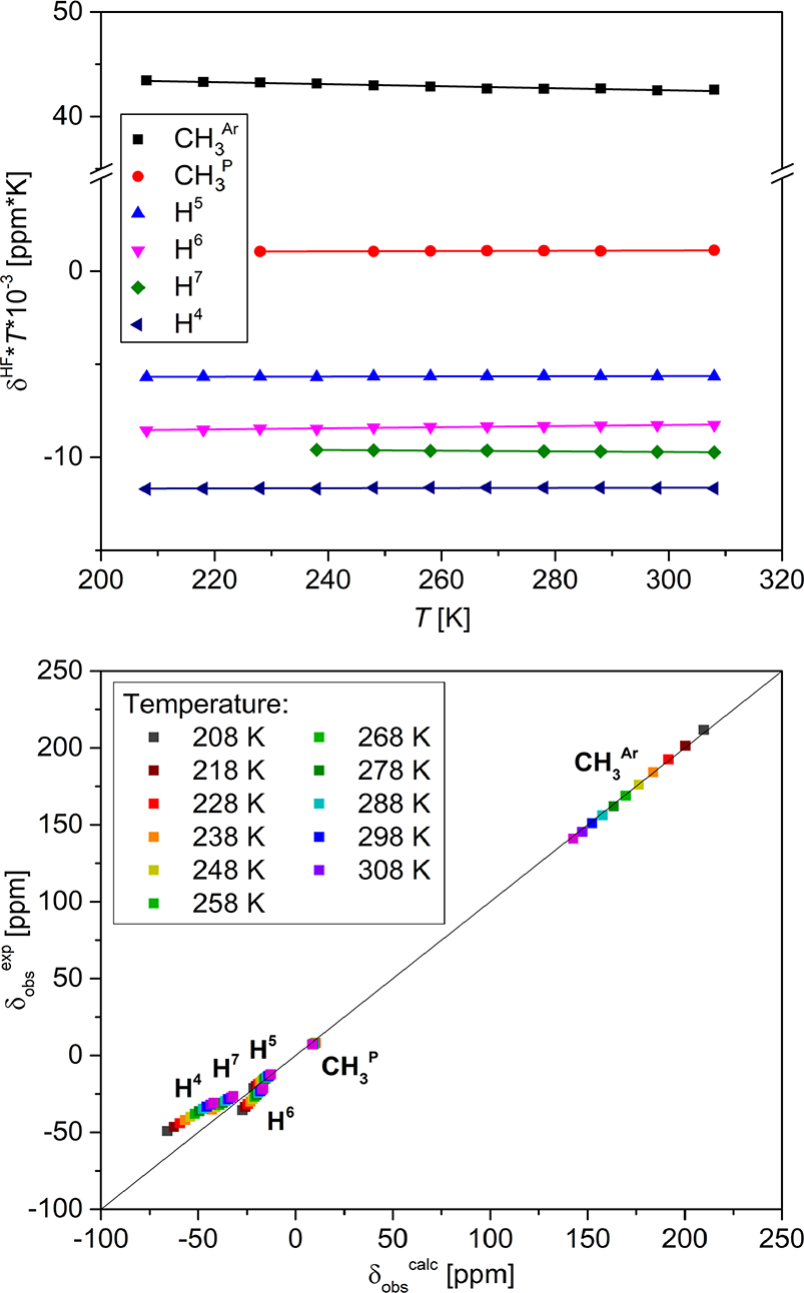
Top panel: variable-temperature ^1^H NMR (400 MHz) δ^HF^*T* products of **4** in DCM-d_2_. Dots show experimental values, straight lines show linear fits (Section S13.3.2). Bottom panel: correlation plots of experimental vs calculated observed chemical shifts for **4** (Section S13.3.3).

The ^1^H NMR spectra of **4** are strongly temperature-dependent (Section S13.3.2), as expected for a paramagnetic compound. Isolation of the hyperfine shifts (δ^HF^) from the observed ones (δ^obs^) by subtracting the diamagnetic contribution (δ^dia^, Section S13.3.2) approximated by an isostructural Ga(III) analogue, [(TSMP)_2_Ga^III^]PPh_4_ (**5b**), reveals that δ^HF^ does not deviate from the Curie behavior (δ^HF^*T* = const, Figure 13, top panel). This implies the dominance of the Fermi contact shifts, which is in line with a moderate axial ZFS parameter of +19.1 cm^–1^ and low rhombicity, as derived from the SQUID and THz-EPR studies as well as DFT and CASSCF calculations (vide supra).

The observed ^1^H chemical shifts can be computationally modeled using a molecular geometry optimized at the PBE-D3BJ/def2-TZVPP level of theory followed by properties calculation at the PBE0-D3BJ/def2-TZVPP level (Section S13.3.3). By using the calculated isotropic proton hyperfine constants (*A*_iso_), experimental *g*_iso_ of 1.97 obtained from THz-EPR (vide supra), diamagnetic shifts (δ^dia^) approximated using **5b**, and by substituting them into eq 2, which only takes into account the Fermi contact contribution (δ^FC^), one arrives to the chemical shifts that are >95% accurate with respect to the experiment (Figure 13, bottom panel). This reinforces our prior conclusions as for very strong dominance of the contact shifts, moderate anisotropy, and insignificant spin delocalization in the system.
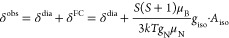
2where δ^dia^ −diamagnetic shift; δ^FC^ – Fermi contact shift; *S* – electronic spin quantum number of the complex; μ_B_ – Bohr magneton; *g*_iso_ – nuclear *g*-value; μ_N_ – nuclear magneton; *k* – Boltzmann constant; *T* – temperature; *g*_iso_ – isotropic electronic *g*-value of the system; *A*_iso_ – isotropic hyperfine coupling constant.

### Optical Spectroscopy and TD-DFT

**Figure 14:**
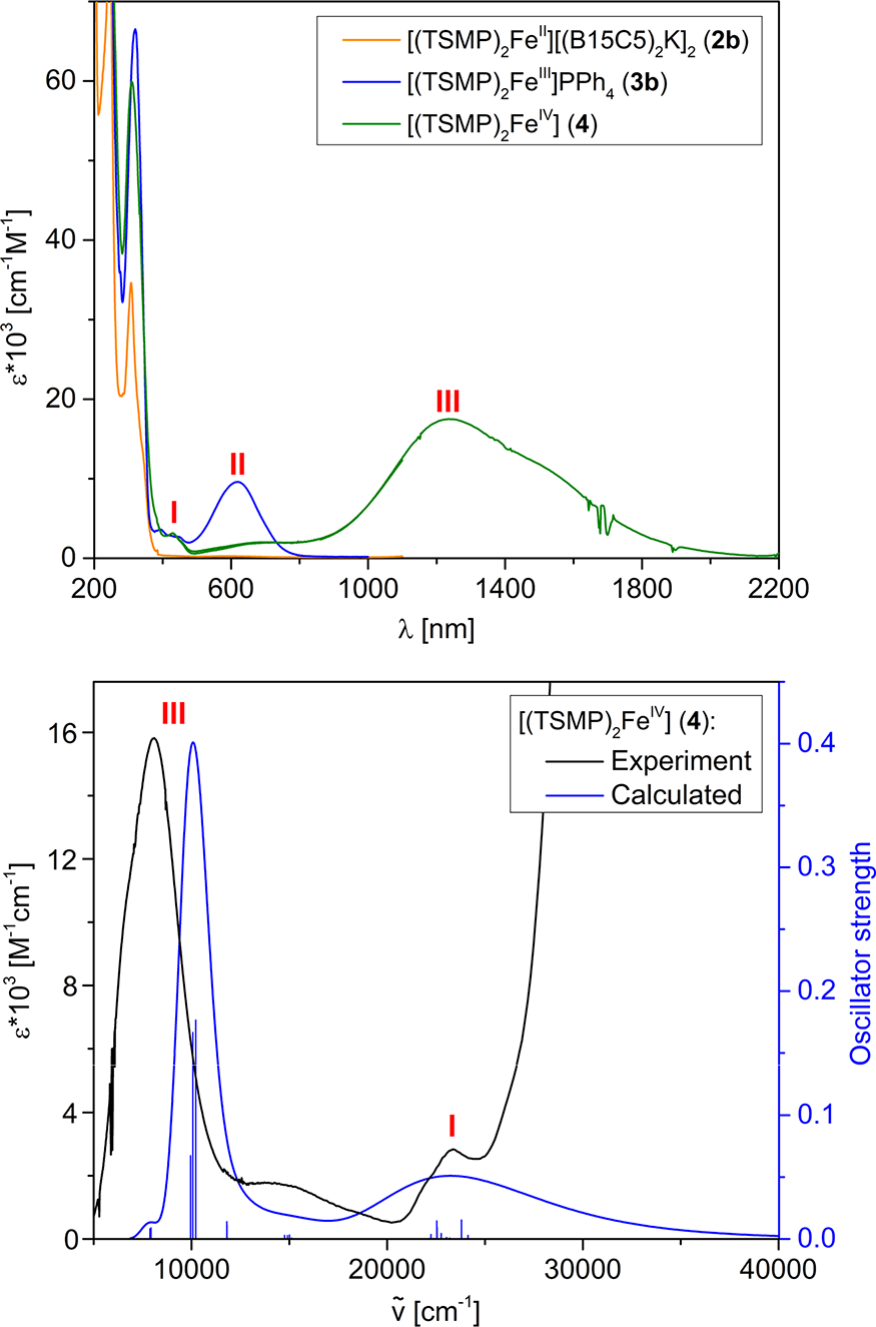
Top panel: UV–vis–NIR spectra of complexes **2b**, **3b**, and **4** in solution at 298 K. Compound **2b** was measured in acetonitrile,^40^**3b** and **4** were measured in DCM. Bottom panel: experimental and TD-DFT-calculated (first 50 excitations) optical spectra of **4**. Calculations were performed at the TPSSh-D3BJ/def2-SVP (def2-TZVP for Fe) level of theory in DCM. Gaussian broadening with FWHM of 170 nm was applied.

Optical spectra of complexes **2b**, **3b**, and **4** in solution are shown in Figure 14, top panel. All three compounds display a set of very intense (ε = 3–13·10^4^ cm^–1^ M^–1^) absorptions below 375 nm, which we assign to π → π* transitions in aromatic ligands and counterions. Furthermore, both **3b** and **4** display a cluster of fairly weak (ε = 2–4·10^3^ cm^–1^ M^–1^) absorptions within 480–375 nm (denoted as **I**), tentatively assigned to higher-lying ligand-to-metal charge transfers (LMCT). Compound **3b** also shows a rather strong (ε = 1·10^4^ cm^–1^ M^–1^) absorption **II** at 618 nm, likely being another LMCT due to its intensity and position. Considering that in DCM solution, where the spectrum was measured, **3b** exists in a spin-state equilibrium (80.8% of HS component at 298 K, vide supra), we undertook variable-temperature UV–vis studies to ascertain the exact origin of feature **II**. Cooling to 180 K, where the compound almost entirely exists in an LS state (3.4% of the HS component based on Evans method, vide supra), results in a feature of roughly the same intensity but shifted to 596 nm (Section S14.1). This leads us to conclude that both the LS and HS states of **3b** show LMCT within the same optical region. Interestingly, compound **4** displays an intense (ε = 1.8·10^4^ cm^–1^ M^–1^) absorption **III** that, rather unusually, peaks in the near-IR region (λ_max_ = 1234 nm). We interpret it as a higher-oxidation-state counterpart of the LMCT feature **II**, in line with our assignment of **4** as a true Fe(IV) compound.

In order to reinforce our assignments of the optical spectra of **4**, we performed time-dependent density functional theory (TD-DFT) calculations (for details, see Section S14.2). Calculated transitions roughly group in two clusters, which correspond to experimental features **I** and **III** (Figure 14, bottom panel). A detailed analysis of these transitions was done using the Natural Transition Orbital (NTO) theory, which performs separate unitary transformations of the occupied and virtual transition molecular orbitals so that only one or very few NTO donor-hole pairs are left, which represent the predominant contribution to the transition.^66^ According to NTO analysis, transitions in a lower-energy cluster **III** occur into the metal-based d_1e_ orbitals (Figure 11), while the donor NTOs are combinations of 3-methylindole HOMO or HOMO-1-like orbitals of individual indolide units with different weight for every inversion-related pair (Section S14.2). The higher-energy cluster **I** is composed of similar transitions into the metal-based d_2e_ orbitals or HOMO-LUMO intra-ligand charge transfer (ICT). In other words, cluster **III** is mostly π → d_1e_ LMCT, and cluster **I** is a mixture of π → d_2e_ LMCT and π → π* ICT. As a matter of fact, similar albeit more energetic transitions were observed for a structurally similar octahedral NHC-derived Fe(IV) phenylborate (**F** in Chart 1).^30^ There, the counterpart of the feature **III** in **F** is 0.74 eV higher in energy, peaking at 715 nm. This observation correlates with the stronger π-accepting properties of the NHC-based borate ligand in **F**, compared to the indolides in TSMP^2–^.

## Conclusions

In conclusion, we have shown that the tris-skatylmethylphosphonium (TSMP^2–^) ligand is capable of supporting both Fe(III) and Fe(IV) oxidation states in the respective isostructural complexes **3** and **4**. Both compounds are electrochemically connected and can be reversibly converted into one another as well as into the parent **2** by a series of one-electron redox reactions.

Complex **3** undergoes thermal *S* = ^1^/_2_ → ^5^/_2_ spin-cross-over both in solution and in the solid state and, to the best of our knowledge, is the only known synthetic system with an Fe^III^N_6_ core capable of doing so. The exact dynamics of this process is highly dependent on the aggregation state, solvent and the counterion. The cross-over is likely possible due to TSMP^2–^ being a relatively weak-field ligand with poor π-accepting properties caused by the absence of low-lying π*-orbitals in the extended aromatic systems.

Compound **4** features an Fe(IV) center with metal-centered oxidation and little spin delocalization on the ligand as shown by a series of electrochemical, spectroscopic, and computational studies. It possesses a triplet (*S* = 1) ground state, similarly to the related trigonal antiprismatic Fe(IV) hexahydrazide clathrochelate (**E** in Chart 1)^29^ and octahedral Fe(IV) tris(*NHC*)phenylborate (**F** in Chart 1)^30^ that were recently reported. All three compounds are C_3_-symmetrical, possessing an axial ZFS parameter *D* within the range of 19.1–23.1 cm^–1^ along with very low rhombicity. Having said that, while **4** features a fairly isotropic *g*-tensor (*g*_iso_ = 1.97), as indicated by THz-EPR, CASSCF calculations, and NMR spectroscopy, **F** has significant anisotropy with *g*_∥_ = 1.88 and *g*_⊥_ = 2.40.^30^ Remarkably, both compounds have green coloration due to an LCMT transition, which is 0.74 eV less energetic in **4**, peaking in the near-IR at 1234 nm. This observation correlates with the lower π-accepting strength of indolides compared to the NHC-based borate ligand in **F**.

Overall, the findings presented in this study demonstrate the utility of the dianionic TSMP^2–^ scorpionate ligand to access the high-valent Fe(IV) state in an octahedral N_6_ coordination environment. The self-consistent and detailed spectroscopic and computational characterization of the electronic structure of **4** will provide a valuable reference for the identification of related systems.

## Supporting Information Available

Synthesis and characterization of **3a-c**, **4**, as well as their deuterated analogues; details on X-ray crystal structure determination of **3a-c**, **4**; CV data; details of spin cross-over in **3**; additional details on spectroscopic and computational characterization of the electronic structure of **4**; and computational coordinates (PDF)
