# Supplementary material for: Homoleptic Fe(III) and Fe(IV) Complexes of a Dianionic C3-Symmetric Scorpionate
Source: Inorg Chem. 2023 Jun 27;62(27):10613–25. doi: 10.1021/acs.inorgchem.3c00871 (PMC10336972; doi:10.1021/acs.inorgchem.3c00871)
Supplement: Supplementary file 1 — ic3c00871_si_001.pdf [file ic3c00871_si_001.pdf]

Supporting information

Homoleptic Fe(III) and Fe(IV) Complexes of a Dianionic C_3-Symmetric Scorpionate

Serhii Tretiakov,[1] Martin Lutz,[2] Charles James Titus,[3] Frank de Groot,[4]* Joscha Nehrkorn,[5] Thomas Lohmiller,[6] Karsten Holldack,[7] Alexander Schnegg,[5]* Maxime François Xavier Tarrago,[8] Peng Zhang,[9,10] Shengfa Ye,[9]* Dmitry Aleshin,[11] Alexander Pavlov,[11,12] Valentin Novikov,[12]* Marc-Etienne Moret[1]*

[1]Utrecht University, Organic Chemistry & Catalysis, Institute for Sustainable and Circular Chemistry, 3584 CG Utrecht, the Netherlands. [2]Utrecht University, Structural Biochemistry, Bijvoet Centre for Biomolecular Research, Faculty of Science 3584 CG Utrecht, the Netherlands. [3]Stanford University, Department of Physics, CA 94305 Stanford, USA (present address: National Institute of Standards and Technology, Materials Measurement Laboratory, MD 20877 Gaithersburg, USA). [4]Utrecht University, Materials Chemistry & Catalysis, Debye Institute for Materials Science, 3584 CG Utrecht, the Netherlands. [5]EPR Research Group, Max-Planck-Institute for Chemical Energy Conversion, 45470 Mülheim/Ruhr, Germany. [6]EPR4Energy Joint Lab, Department Spins in Energy Conversion and Quantum Information Science, Helmholtz Zentrum Berlin für Materialien und Energie GmbH, 12489 Berlin, Germany (present address: Institut für Chemie, Humboldt-Universität zu Berlin, 12489 Berlin, Germany). [7]Department of Optics and Beamlines, Helmholtz Zentrum Berlin für Materialien und Energie GmbH, 12489 Berlin, Germany. [8]Department of Molecular Theory and Spectroscopy, Max Planck Institut für Kohlenforschung, 45470 Mülheim/Ruhr, Germany. [9]State Key Laboratory of Catalysis, Dalian Institute of Chemical Physics, Chinese Academy of Sciences. Dalian 116023, P. R. China. [10]University of Chinese Academy of Sciences, Beijing 10049, China. [11]A.N. Nesmeyanov Institute of Organoelement Compounds, Russian Academy of Sciences, Vavilova str. 28, Moscow, Russia. [12]Moscow Institute of Physics and Technology, Institutskiy per., 9, Dolgoprudny, Moscow Region, Russia.

Corresponding authors: [1]m.moret@uu.nl; [4]F.M.F.deGroot@uu.nl; [5]alexander.schnegg@cec.mpg.de; [9]shengfa.ye@dicp.ac.cn; [12]novikov.vv@mipt.ru.

Contents[a]

S1 General remarks ... S4
S2 Synthesis and basic characterization ... S5
S2.1 Metal complexes ... S5
$[(TSMP)_2Fe^{III}]K$ (**3a**) ... S5
$[(TSMP)_2Fe^{III}]PPh_4$ (**3b**) ... S6
$[(TSMP)_2Fe^{III}][(B15C5)_2K]$ (**3c**) ... S7
$[(TSMP)_2Fe^{IV}]$ (**4**) ... S8
$[(TSMP)_2Ga^{III}]K$ (**5a**) ... S9
Generation of $[(TSMP)_2Ga^{III}]PPh_4$ (**5b**) ... S10
S2.2 Deuterium-labeled ligand ... S11
tris-(*N*-Boc-3-methylindol-2-yl)($^2H_3$)methylphosphonium iodide (**9-*d₃***) ... S11
tris-(1*H*-3-methylindol-2-yl)($^2H_3$)methylphosphonium chloride (**11-d_3**) ... S12
(TSMP-d_3)K_2 (**1-d_3**) ... S12
S2.3 Deuterium-labeled complexes ... S13
$[(TSMP\text{-}d_3)_2Fe^{II}][(B15C5)_2K]_2$ (**2b-d_6**) ... S13
$[(TSMP\text{-}d_3)_2Fe^{III}]K$ (**3a-d_6**) ... S14
$[(TSMP\text{-}d_3)_2Fe^{III}]PPh_4$ (**3b-d_6**) ... S14
$[(TSMP\text{-}d_3)_2Fe^{IV}]$ (**4-d_6**) ... S15
S2.4 Additional experiments ... S15
Attempted synthesis of $[(TSMP)_2Fe^{III}]K$ (**3a**) using $FeCl_3$ ... S15
S3 X-ray crystal structure determinations ... S15
$[(TSMP)_2Fe^{III}]K$ (**3a**) ... S15
$[(TSMP)_2Fe^{III}]PPh_4$ (**3b**) ... S17
$[(TSMP)_2Fe^{III}][(B15C5)_2K]$ (**3c**) ... S18
$[(TSMP)_2Fe^{IV}]$ (**4**) ... S19
$[(TSMP)_2Ga^{III}]K$ (**5a**) ... S20
S4 Computational details ... S21
S5 Cyclic voltammetry ... S22
S5.1 Experimental details ... S22
S5.2 Cyclic voltammetry of $[(TMSP)_2Fe^{III}]K$ (**3a**) ... S22
S6 Solution magnetic moment and spin-crossover fitting ... S24
S6.1 Experimental details ... S24
S6.2 Spin-crossover fitting of $[(TSMP)_2Fe^{III}]^-$ (**3**) ... S24
S7. XAS spectroscopy ... S27
S7.1 Experimental details ... S27
S7.2 Computational details ... S27

[a] For the reader's convenience, this document is enhanced with hyperlinks. Clicking on any mention of a section/title/figure in the text will bring the reader to the object that was referred to.

S8. SQUID magnetometry studies S28
S8.1 Experimental details S28
S8.2 Solid state SQUID magnetometry of $[(TSMP)_2Fe^{III}][(B15C5)_2K]$ (**3c**) S28
S9. THz-EPR studies S29
S9.1 Experimental details S29
S9.2 General energy diagram S30
S9.3 Alternative interpretations S31
S9.4 The negative relative absorbance feature at ~18.4 cm^{-1} S32
S10. ^{57}Fe Mössbauer spectroscopy S34
S10.1 Experimental details S34
S10.2 Detailed breakdown of the quadrupole splitting S34
S11. DFT calculations S36
S11.1 Calculated Mössbauer parameters of $[(TSMP)_2Fe^{IV}]^0$ (**4**) S36
S11.2 Spin density in $[(TSMP)_2Fe^{IV}]^0$ (**4**) S37
S11.3 Computational geometries of $[(TSMP)_2Fe^{IV}]^0$ (**4**) and its Si-tethered analogue S38
S12. CASSCF calculations S38
S12.1 Calculation of the ZFS parameters of $[(TSMP)_2Fe^{IV}]^0$ (**4**) S38
S13 Paramagnetic NMR studies S40
S13.1 Theoretical background S40
S13.1.1 On the origin of paramagnetic shifts S40
S13.1.2 Assignments of paramagnetic NMR signals S44
S13.2 $[(TSMP)_2Fe^{III}]^-$ (**3**) complex S45
S13.2.1 Signal assignments S45
S13.2.2 Variable-temperature behavior S47
S13.3 $[(TSMP)_2Fe^{IV}]$ (**4**) complex S53
S13.3.1 Signal assignments S53
S13.3.2 Variable-temperature behavior S54
S13.3.3 Partitioning of hyperfine shifts S57
S14. Optical spectroscopy S62
S14.1 Variable-temperature UV-Vis spectra of $[(TSMP)_2Fe^{III}]PPh_4$ (**3b**) S62
S14.2 TDDFT calculations S62
S15 Spectra of isolated compounds S81
S16 Computational coordinates S134
S16.1 $[(TSMP)_2Fe^{III}]^-$ (**3**) S134
S16.2 $[(TSMP)_2Fe^{IV}]^0$ (**4**) S138
S16.3 Si-tethered analogue of $[(TSMP)_2Fe^{IV}]^0$ (**4**) S154
S16.4 3-methylindole S156
S17 Literature references S158

S1 General remarks

All reactions involving air-sensitive compounds were conducted under a N_2 atmosphere by using standard glovebox or Schlenk techniques.

Acetonitrile, diethyl ether and *n*-hexane were dried with an MBRAUN MB SPS-79 system. Acetonitrile was additionally dried by passing through a column of activated neutral alumina after being kept over ca. 5 vol.% of 3Å molecular sieves over 48h. THF was distilled from benzophenone/Na. Pyridine, DCM and butyronitrile were distilled from CaH_2. Other solvents were used as supplied. Dried solvents were degassed by sparging with dry nitrogen for 30 min. and stored in a glovebox under nitrogen atmosphere and over molecular sieves, except for acetonitrile which was stored without the sieves. Deuterated solvents were acquired from Cambridge Isotope Laboratories, Inc. Chloroform-*d* and methylene chloride-d_2 were used as supplied, all other deuterated solvents were dried as indicated above for their proteo-analogues. Dried deuterated solvents were degassed by four freeze-pump-thaw cycles and stored in a glovebox over molecular sieves, except for acetonitrile-d_3 which was stored without the sieves. Phosphorus trichloride, trifluoroacetic acid, methyl iodide and methyl iodide-d_3 (>99 atom%) were purchased from Acros. All other chemicals were purchased from Sigma-Aldrich. Potassium hydride was supplied as a 30 wt.% suspension in mineral oil and was washed with dry and degassed *n*-hexane prior to use. $FeCl_2$ and $GaCl_3$ were purchased in an anhydrous form. Benzo-15-crown-5 was recrystallized from heptane. All other commercially obtained chemicals were used as received.

All NMR measurements were performed at 298 K (unless stated otherwise) on a Varian VNMRS400 or Varian MRF400 spectrometer, chemicals shifts are reported relative to TMS with the residual solvent signal as internal standard.[1] All NMR experiments involving air-sensitive compounds were conducted in J. Young NMR tubes under an N_2 atmosphere. Peak multiplicity was quoted as s (singlet), d (doublet), t (triplet) and so on. In cases of unresolved couplings that strongly affect the line shape of individual components of an otherwise well-defined multiplet, the apparent multiplicity was quoted as 's' ('singlet'), 'd' ('doublet'), 't' ('triplet') and so on. In case of an overlap with solvent or impurity signals, where possible, integral intensities were extracted using MNova[2] peak deconvolution tool. ASAPHMQC experiments were conducted using the corresponding pulse sequence[3] as implemented in the VnmrJ 4.2 software.[4]

IR spectra were recorded on a Perkin-Elmer Spectrum Two FT-IR spectrometer. The bands were classified by an absorption intensity as: very weak (VW; 0-10% of the most intense absorption in the spectrum), weak (W; 10-30%), medium (M; 30-60%), strong (S; 60-90%), very strong (VS; 90-100%).

UV-Vis spectra were measured on a PerkinElmer Lambda 35 spectrometer. UV-Vis-NIR spectra were recorded using double-beam Perkin Elmer Lambda 950 UV/Vis spectrophotometer. ESI-MS measurements were performed on a Waters LCT Premier XE KE317 spectrometer or, alternatively, Adrion Expression CMS spectrometer. Elemental analysis was conducted by Medac Ltd.

S2 Synthesis and basic characterization

S2.1 Metal complexes

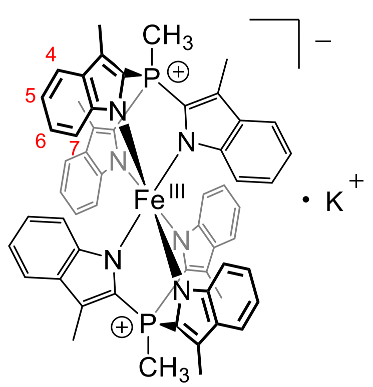

3a

[(TSMP)$_2$Fe^{III}]K (3a). Complex **3a** was prepared by *in situ* oxidation of the precursor [(TSMP)$_2$Fe^{II}]K$_2$ (**2a**), formed from FeCl$_2$ and TSMPK$_2$ salt (**1**) and characterized by us elsewhere.[5] The oxidizing agents, ferrocenium tetrafluoroborate and tritilium tetrafluoroborate, are interchangeable in terms of the procedure below.

Dry THF (100 ml) was added to a mixture of TSMPK$_2$ salt (**1**) (contains 28.6 wt% of THF, 2.00 g, 2.79 mmol, 2.00 equiv.) and anhydrous FeCl$_2$ (0.177 g, 1.40 mmol, 1.00 equiv.) under dinitrogen atmosphere, followed by stirring overnight at room temperature. The stirring was stopped, and the fine bright-yellow suspension was allowed to separate in order to facilitate the subsequent filtration. The reaction was then filtered through a fine sintered glass funnel. The potassium chloride filter cake was washed with three small portions of THF (3 x 3.0 ml), which were combined with the filtrate. The solvent from the latter was removed *in vacuo* to form a viscous dark-yellow oil, which was then redissolved in anhydrous acetonitrile (50 ml). This was followed by dropwise addition of a solution of an oxidizing agent (ferrocenium or tritylium tetrafluoroborate, 0.95 equiv.) in acetonitrile (30 ml), immediately accompanied by the change of coloration from yellow to green and, finally, navy blue. The mixture was stirred for 30 min. followed by filtration through a fine sintered glass funnel. The potassium tetrafluoroborate filter cake was washed with three small portions of acetonitrile (3 x 3.0 ml), which were combined with the filtrate. The latter was dried *in vacuo* to form a distinctly microcrystalline deep-blue, almost black, solid. The solid was washed with multiple small portions of THF (5 x 15.0 ml) until the washings became pale-blue with no changes in hue between the subsequent washes. The solid was then dried *in vacuo* to afford a black-blue microcrystalline powder of **3a** with 64.6% yield (0.626 g). Crystals suitable for X-ray diffraction analysis were grown by vapor diffusion of diethyl ether into an acetonitrile solution of **3a** at room temperature.

Anion [(TSMP)$_2$Fe^{III}]$^-$ (**3**) and, by extension, compound **3a** undergo thermal spin-crossover (SCO) both in the solid state and solution (see main text as well as *Sections S6.2* and *S8.2*). Moreover, the SCO dynamics in solution depends on the specific solvent, on top of temperature dependence of the hyperfine NMR chemical shifts (*Section S13.2*). Solvent- and temperature dependence are also true for the effective molecular magnetic moment in solution as determined by the Evans method.[6–8] Importantly, the variable-temperature Evans method measurements performed using TMS and a residual underdeuterated NMR solvent signal as a reference are identical within the accuracy of the method (5-10%[9]). This implies that the solvent chemical shifts have no observable hyperfine component which, otherwise, would be a result of a specific interaction such as coordination. Furthermore, the hyperfine ^{1}H NMR shifts of **3** follow the same trend regardless of solvent (with correction for the SCO critical temperature; see *Section S13.2.2*). These two observations indicate that dissociation of the ligand arms in **3**

followed by the solvent coordination is very unlikely. As will be shown below, this is in contrast with the solution behavior of the $[(TSMP)_2Ga^{III}]^-$ (**5**) analogue.

All measurements described below were performed at 298 K. The assignments of ^1H NMR signals are discussed in detail in *Section S13.2.1*. In case of overlap with solvent or impurity signals, where possible, integral intensities were extracted using the MNova[2] peak deconvolution tool. **^{1}H NMR** (400 MHz, acetonitrile-d_3) δ 70.1 (br. s., 3H, Ar-CH_3), 29.9 (br. s., 1H, H^4), 11.8 (s, 1H, P^+-CH_3), -4.5 (s, 1H, H^6), -7.0 (br. s., 1H, H^5). ^1H NMR (400 MHz, pyridine-d_5) δ 76.6 (br. s., 3H, CH_3^{Ar}), 34.8 (br. s., 1H, H^4), 10.8 (br. s., 1H, P^+-CH_3), -4.2 (br. s., 1H, H^6), -7.4 (br. s., 1H, H^5). **^{31}P NMR** (162 MHz, acetonitrile-d_3) δ -236.2 (br. s.). **^{31}P NMR** (162 MHz, pyridine-d_5) δ -295.7 (br. s.). **Effective magnetic moment in solution** determined in acetonitrile-d_3 is 4.64 μ_B, which indicates a mid-spin-crossover situation ($S = {}^1/_2 \rightarrow {}^5/_2$; see detailed discussion in *Sections S6.2*). Effective magnetic moment of 5.14 μ_B determined in pyridine-d_5 supports this conclusion. **UV-Vis** (acetonitrile): λ_{max} (ε)= 211 ($8.91 \cdot 10^4$), 238 ($8.87 \cdot 10^4$), 318 ($5.46 \cdot 10^4$), 392 ($2.17 \cdot 10^3$), 422 ($1.84 \cdot 10^3$), 447 ($1.80 \cdot 10^3$), 609 nm ($7.71 \cdot 10^3$ $cm^{-1}M^{-1}$). **ATR-FTIR** (neat) ṽ (cm^{-1}): 440 (M), 465 (M), 530 (W), 540 (W), 567 (W), 623 (M), 661 (S), 738 (S), 813 (M), 838 (M), 889 (S), 912 (W), 1004 (W), 1042 (M), 1100 (M), 1130 (M), 1198 (S), 1209 (S), 1248 (VS), 1291 (M), 1339 (M), 1362 (M), 1421 (M), 1450 (W), 1499 (M), 1597 (W), 2245 (W), 2249 (VW), 2865 (M), 2928 (M), 2989 (M), 2998 (M), 3040 (M). **ESI-TOF-MS** in CH_3CN: 922.2214 $[M]^-$ (calcd. 922.2767 $[M]^-$). No satisfactory elemental analysis could be obtained due to the high reactivity of **3a** and its ability to retain variable amounts of solvent.

Appended spectra: NMR in acetonitrile-d_3: ^1H (*Figure S40*), ^{31}P (*Figure S41*); NMR in pyridine-d_5: ^1H (*Figure S42*), ^{31}P (*Figure S43*); UV-Vis in acetonitrile (*Figure S44*); ATR-FTIR (neat, *Figure S45*).

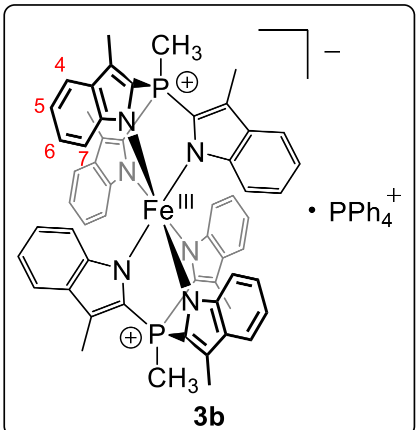

3b

$[(TSMP)_2Fe^{III}]PPh_4$ (3b). Complex **3b** was prepared by ion exchange of $[(TSMP)_2Fe^{III}]K$ (**3a**) and tetraphenylphosphonium iodide (PPh_4I) in DCM. The procedure is as follows.

Dry DCM (25 ml) was added to a mixture of PPh_4I (0.230 g, 0.494 mmol, 0.950 equiv.) and **3a** (0.500 g, 0.520 mmol, 1.00 equiv.), which is otherwise insoluble in DCM. Immediately, formation of a navy blue solution ensued, and the reaction was stirred for 30 min. followed by solvent removal *in vacuo* (to completely precipitate KI), resuspension in DCM, filtration and another vacuum solvent removal. The above manipulations yielded 0.623 g of a dark-blue, almost black, solid in quantitative yield yield with respect to PPh_4I. In principle, this material is sufficiently pure for further spectroscopic characterization and synthetic use. An additional purification step can be undertaken by recrystallization from DCM solution layered with *n*-hexane (crytallization yield: 88.5%), which is how the crystals for X-ray diffraction analysis were grown.

As discussed in the synthesis of $[(TSMP)_2Fe^{III}]K$ (**3a**) (*vide supra*), anion $[(TSMP)_2Fe^{III}]^-$ (**3**) undergoes thermal spin-crossover, which also, by extension, applies to **3b**. Therefore, it is important to note that all characterization described below was performed at 298 K. The measurements were performed on the recrystallized material. The assignments of the ^1H NMR signals are discussed in detail in *Section S13.2.1*. While the ^1H NMR spectra show an increased intensity of the $[PPh_4]^+$ signals with respect to what is needed to match the stoichiometry of **3b** (see *Appended spectra*), the ^{31}P spectra display the expected 1:2 intensity ratio. We connect the mismatch with problematic base line correction in ^1H spectra due to the presence of an intense dichloromethane signal. **^{1}H NMR** (400 MHz, dichloromethane-d_2) δ 67.7 (br. s., 3H, Ar-CH_3), 27.5 (br. s., 1H, H^4), 12.2 (br. s., 1H, P^+-CH_3), 8.14 – 6.89 (m, intensity unreliable/see above, $[PPh_4]^+$), -4.5 (br. s., 1H, H^6), -6.7 (br. s., 1H, H^5). **^{31}P NMR** (162 MHz, dichloromethane-d_2) δ 23.1 (s, carbon satellites: $J_{P,C}$ = 89.5 Hz, 1P, $[PPh_4]^+$), -213.5 (br. s., 2P, P^+-CH_3). **Effective magnetic moment in solution** determined in dichloromethane-d_2 at 298K is 4.68 μ_B, which indicates a mid-spin-crossover situation ($S = {}^1/_2 \rightarrow {}^5/_2$; see detailed discussion in *Sections S6.2*). **UV-Vis** (dichloromethane): λ_{max} (ε)= 236 ($1.31\cdot10^5$), 276 ($3.61\cdot10^4$), 320 ($6.65\cdot10^4$), 394 ($3.62\cdot10^3$), 423 ($2.86\cdot10^3$), 444 ($2.71\cdot10^3$), 618 nm ($9.58\cdot10^3$ $cm^{-1}M^{-1}$). No satisfactory elemental analysis could be obtained due to high reactivity of **3b** and its ability to retain variable amounts of solvent.

Appended spectra: NMR in dichloromethane-d_2: ^1H (*Figure S46*), ^{31}P (*Figure S47*); UV-Vis in dichloromethane (*Figure S44*).

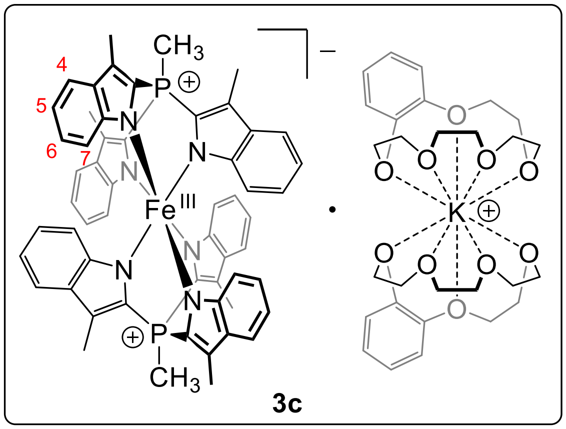

3c

$[(TSMP)_2Fe^{III}][(B15C5)_2K]$ (3c). Complex **3c** was prepared by chelating the potassium cation in $[(TSMP)_2Fe^{III}]K$ (**3a**) with an excess of benzo-15-crown-5. The procedure is as follows.

Dry acetonitrile (40 ml) was added to a mixture of **3a** (0.500 g, 0.520 mmol, 1.00 equiv.) and benzo-15-crown-5 (0.286 g, 1.07 mmol, 2.05 equiv.), and the mixture was stirred for 1h, upon which the solvent was removed *in vacuo*. The resulting blue solid was thorougly washed with small portions of *n*-hexane (5 x 10.0 ml) and dried *in vacuo* to yield 0.779 g of fine blue powder in a quantitative yield. In principle, this material is sufficiently pure for furter spectroscopic characterization. An additional purification step can be undertaken by recrystallization from pyridine solution layered with *n*-pentane (crytallisation yield: 93.7%), which is how the crystals for X-ray diffraction analysis were grown.

As discussed in the synthesis of $[(TSMP)_2Fe^{III}]K$ (**3a**) (*vide supra*), anion $[(TSMP)_2Fe^{III}]^-$ (**3**) undergoes thermal spin-crossover, which also, by extension, applies to **3c**. Therefore, it is important to note that all characterization described below was performed at 298 K. The measurements were performed on the recrystallized material. The assignments of the ^1H NMR signals are discussed in detail in *Section S13.2.1*. The ^1H NMR spectra show an increased

intensity of the bis(benzo-15-crown-5)potassium signals with respect to what in needed to match the stoichiometry of **3c** (see *Appended spectra*). A similar situation was observed for $[(TSMP)_2Fe^{III}]PPh_4$ (**3b**) salt (*vide supra*), which we connect with problematic base line correction due to the presence of intense solvent signals. **^{1}H NMR** (400 MHz, acetonitrile-d_3) δ 70.2 (br. s., 3H, Ar-CH_3), 30.0 (br. s., 1H, H^4), 11.8 (br. s., 1H, P^+-CH_3), 7.01 – 6.76 (m, intensity unreliable/see above, Ar of benzo-15-crown-5), 4.12 – 3.41 (m, intensity unreliable/see above, CH_2 of benzo-15-crown-5), -4.5 (br. s., 1H, H^6), -7.1 (br. s., 1H, H^5). **^{31}P NMR** (162 MHz, acetonitrile-d_3) δ -236.9 (br. s.). No satisfactory elemental analysis could be obtained due to the high reactivity of **3c** and its ability to retain variable amounts of solvent.

Appended spectra: NMR in acetonitrile-d_3: ^{1}H (*Figure S48*), ^{31}P (*Figure S49*).

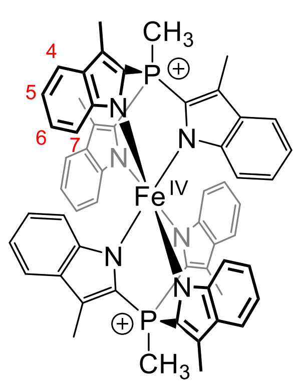

4

$[(TSMP)_2Fe^{IV}]$ (4). Complex **4** was prepared by oxidation of $[(TSMP)_2Fe^{III}]PPh_4$ (**3b**) with elemental iodine. The advantage of using a PPh_4^+ salt **3b** is that the reaction product PPh_4I is more soluble in dichloromethane than the target complex **4**, which allows to isolate the latter by washing with the solvent.

A solution of elemental iodine (0.046 g, 0.180 mmol, 0.475 equiv.) in dichloromethane (4.0 ml) was added dropwise over a minute to a stirred dichloromethane solution of **3b** (0.480 g, 0.380 mmol, 1.00 equiv. in 10.0 ml). The dark suspension was stirred over 40 min, after which it was filtered, and the dark-green filter cake was washed with DCM (3 x 2.0 ml) followed by drying *in vacuo*. The above manipulations yielded a fine bottle green powder with 90.1% yield (0.318 g). Crystals suitable for X-ray diffraction analysis were grown by vapor diffusion of diethyl ether into a solution of **4** in pyridine-butyronitrile 2:1 (v/v) mixture at –40 °C.

All measurements described below were performed at 298 K. The assignments of ^{1}H NMR signals are discussed in detail in *Section S13.3.1*. ^{31}P signals could not be detected within the probed range (±400 ppm). **^{1}H NMR** (400 MHz, dichloromethane-d_2) δ 145.4 (br. s., 3H, Ar-CH_3), 7.2 (br. s., 1H, P^+-CH_3), -12.8 (s, 1H, H^5), -22.2 (s, 1H, H^6), -27.4 (br. s., 1H, H^7), -32.0 (br. s., 1H, H^4). **UV-Vis-NIR** (dichloromethane): λ_{max} (ε)= 239 ($9.77\cdot10^4$), 309 ($5.99\cdot10^4$), 429 ($3.15\cdot10^3$), 702 ($2.00\cdot10^3$), 1234 nm ($1.75\cdot10^4$ $cm^{-1}M^{-1}$). **ATR-FTIR** (neat) $\tilde{\nu}$ (cm^{-1}): 441 (W), 539 (W), 574 (VW), 591 (W), 627 (W), 651 (W), 736 (M), 771 (W), 814 (W), 842 (M), 913 (M), 1061 (M), 1141 (S), 1194 (M), 1238 (VS), 1271 (M), 1323 (M), 1330 (M), 1380 (M), 1413 (M), 1425 (M), 1500 (M), 1559 (VW), 1595 (VW), 2251 (VW), 2901 (S), 2972 (VS), 2988 (VS), 3662 (W), 3675 (M), 3685 (W). **ESI-TOF-MS** spectra in CH_3CN recorded in a negative mode show a product of one-electron reduction of **4**: 922.2103 $[M]^-$ (calcd. 922.2767 $[M]^-$). No satisfactory elemental analysis could be obtained because of **4** retaining an unknown amount of solvent due to the presence of cationic charges.

Appended spectra: NMR in dichloromethane-d_2: ^{1}H (*Figure S50*); UV-Vis in dichloromethane (*Figure S51*); ATR-FTIR (neat, *Figure S52*).

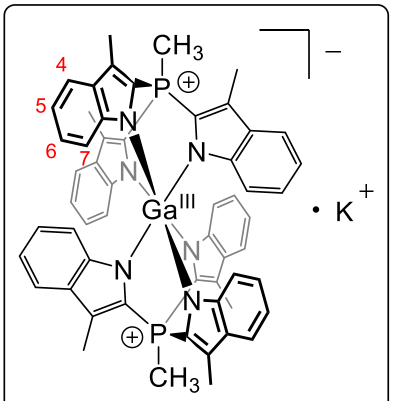

5a

[(TSMP)$_2$Ga^{III}]K (5a). Complex [(TSMP)$_2$Ga]$^-$ (**5**) was used to approximate the orbital component of the chemical shifts of [(TSMP)$_2$Fe^{III}]$^-$ (**3**) and [(TSMP)$_2$Fe^{IV}] (**4**). The potassium salt **5a** was prepared by reaction of $GaCl_3$ with two equivalents of the TSMPK$_2$ (**1**). The procedure is as follows.

A solution of $GaCl_3$ (0.068 g, 0.38 mmol, 1.0 equiv.) in acetonitrile (3.0 ml) was added dropwise over a minute to a solution of TSMPK$_2$ salt (**1**) (contains 21.5 wt% of THF, 0.500 g, 0.768 mmol, 2.00 equiv.) in acetonitrile (7.0 ml). The reaction mixture was allowed to stir for 16 h, after which it was filtered and the filter cake was washed with acetonitrile (3 x 2.0 ml). The filtrate was concentrated in vacuo to ca. 2 ml, and the precipitated solid was collected and combined with the filter cake while the yellow liquor was discarded. Combined solids were extracted with acetonitrile (100 ml in total), which was then evaporated *in vacuo* to afford a white powder (102 mg, yield: 27.5%). Crystals suitable for X-ray diffraction analysis were grown by vapor diffusion of diethyl ether into an acetonitrile solution of **5a** at room temperature.

The procedure yields a highly pure material as evidenced by the NMR spectra in acetonitrile-*d$_3$* (see *Appended spectra*). The fact that **5a** retains the assigned geometry on the ^{1}H measurement time scale in acetonitrile-*d$_3$* solution is evident from only six signals present in the spectrum, which can only correspond to the C$_3$-symmetric heterobicyclo[2.2.2]octane topology of the TSMP scaffold. Furthermore, some of the aromatic signals appear at unusually low chemical shifts with the lowest one corresponding to the H^7 proton at 5.16 ppm followed by H^6 at 5.46 ppm, H^5 at 6.15 ppm and H^4 at 7.12 ppm, the latter being a normal value for an aromatic proton. Such a progression can only be the case if two TSMP ligands “interlock” around a Ga^{III} center forming an D$_{3d}$-symmetric system, within which the protons with a higher assigned index are shielded due to magnetic anisotropy of the aromatic rings. Interestingly, in pyridine-*d$_5$* solution, one can still observe the D$_{3d}$-symmetric configuration of **5a** but accompanied by half the molar amount of a highly-fluxional species with 2:1 symmetry as evidenced by the aromatic methyl group signals. This is in contrast with the analogous [(TSMP)$_2$Fe^{III}]K (**3a**) complex, which retains its D$_{3d}$-geometry in pyridine-*d$_5$* solution as indicated by variable-temperature measurements of the effective molecular magnetic moment by Evans method and variable-temperature hyperfine ^{1}H NMR chemical shifts (see the synthesis of [(TSMP)$_2$Fe^{III}]K (**3a**) above for more detail).

^{1}H NMR spectral assignments of **5a** in pyridine-*d$_5$* below only feature the signals that correspond to the S$_6$-symmetric configuration. In case of an overlap with solvent or impurity signals, where possible, integral intensities were extracted using MNova[2] peak deconvolution tool. **^{1}H NMR** (400 MHz, acetonitrile-*d$_3$*) δ 7.12 (d, $J_{H,H}$ = 7.9 Hz, 1H, H^4), 6.15 (t, $J_{H,H}$ = 7.3 Hz, 1H, H^5), 5.46 (t, $J_{H,H}$ = 7.5 Hz, 1H, H^6), 5.16 (d, $J_{H,H}$ = 8.6 Hz, 1H, H^7), 3.57 (d, $J_{H,P}$ = 15.2 Hz, 1H, P$^+$-CH$_3$), 2.79 (d, $J_{H,P}$ = 1.1 Hz, 3H, Ar-CH$_3$). ^{1}H NMR (400 MHz, pyridine-*d$_5$*) δ 7.40 (d, $J_{H,H}$ = 7.8 Hz, 1H, H^4), 6.34 (t, $J_{H,H}$ = 7.3 Hz, 1H, H^5), 6.25 (d, $J_{H,H}$ = 8.6 Hz, 1H, H^6), 5.90 (t, $J_{H,H}$ = 7.6 Hz, 1H, H^7), 3.74 (d, $J_{H,P}$ = 15.1 Hz, 1H, P$^+$-CH$_3$), 2.85 (s, 3H, Ar-CH$_3$). **^{13}C NMR**

(101 MHz, acetonitrile-d_3) δ 148.1 (d, $J_{C,P}$ = 15.2 Hz, indole-C8), 129.4 (d, $J_{C,P}$ = 14.4 Hz, indole-C9 or C3), 125.7 (d, $J_{C,P}$ = 112.1 Hz, indole-C2), 119.8 (s, indole-C6), 118.0 (d, $J_{C,P}$ = 1.6 Hz, indole-C4), 117.4 (d, $J_{C,P}$ = 1.8 Hz, indole-C7), 116.5 (d, $J_{C,P}$ = 1.3 Hz, indole-C5), 115.4 (d, $J_{C,P}$ = 20.0 Hz, indole-C3 or C9), 10.7 (s, Ar-CH_3), 5.6 (d, $J_{C,P}$ = 57.8 Hz, P^+-CH_3). **^{31}P NMR** (162 MHz, acetonitrile-d_3) δ -14.9 (br. s.). ^{31}P NMR (162 MHz, pyridine-d_5) δ -12.8 (br.s.). **UV-Vis** (acetonitrile): λ_{max} (ε)= 227 ($1.21\cdot10^5$), 238 ($1.17\cdot10^5$), 318 nm ($8.13\cdot10^4$ $cm^{-1}M^{-1}$). **ESI-TOF-MS** in CH_3CN: 935.2298 $[M]^-$ (calcd. 935.2672 $[M]^-$). No satisfactory elemental analysis could be obtained due to high reactivity of **5a** and its ability to retain variable amounts of solvent.

Appended spectra: NMR in acetonitrile-d_3: ^{1}H (*Figure S53*), ^{13}C (*Figure S54*), ^{31}P{^{1}H} (*Figure S55*), ^{1}H-^{13}C ASAPHMQC (*Figure S56*), NOESY (*Figure S57*); NMR in pyridine-d_5: ^{1}H (*Figure S58*), ^{31}P (*Figure S59*), ^{31}P{^{1}H} (*Figure S60*); UV-Vis in acetonitrile (*Figure S61*).

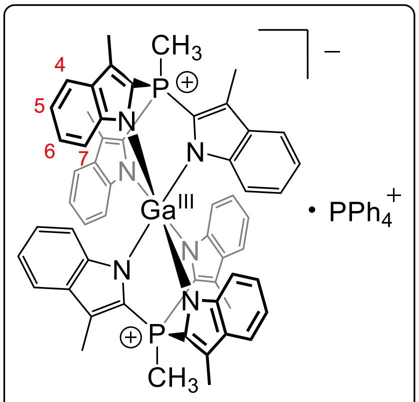

5b

Generation of [(TSMP)$_2$Ga^{III}]PPh$_4$ (5b). Complex [(TSMP)$_2$Ga]$^-$ (**5**) was used to approximate the orbital component of the chemical shifts of [(TSMP)$_2$Fe^{III}]$^-$ (**3**) and [(TSMP)$_2$Fe^{IV}] (**4**). An attempt to solubilize the synthesized above [(TSMP)$_2$Ga^{III}]K (**5a**) (10 mg, 0.010 mmol, 1.0 equiv.) in dichloromethane-d_2 (0.6 ml) in the presence of PPh_4I (3.5 mg, 0.0076 mmol, 0.75 equiv.), led to a homogenous solution. The ^{1}H NMR spectrum indicates the presence of two species in a ratio of 1.6 to 1, the minor of which can be identified by the unusually low aromatic chemical shifts (*vide supra*) as an D_{3d}-symmetric configuration of [(TSMP)$_2$Ga^{III}]$^-$ (**5**). The major species is higly fluxional (broad aromatic signals) and shows 1:2 symmetry breaking as indicated by the aromatic methyl group signals. Since the ion exchange clearly happens as evidenced by solubilization of [(TSMP)$_2$Ga^{III}]K (**5a**), yet no KI precipitation occurs, we speculate that the major species forms due to coordination of an iodide anion to the Ga^{III} center in **5**. ^{1}H NOE spectra indicate that the major and the minor species are in dynamic exchange with one another. Similar spectra are observed if ion exchange is undertaken with an equal amount of PPh_4Cl instead of PPh_4I.

Since the speciation in solution is complicated and a subequivalent amount of PPh_4I was used, the NMR integral intensities of the $[PPh_4]^+$ cation are devoid of the straightforward chemical meaning and, therefore, are not provided in the assignment below. **^{1}H NMR** (400 MHz, dichloromethane-d_2) δ 7.83 (t, J = 7.4 Hz, intensity uninformative/see above, $[PPh_4]^+$: H^4), 7.65 (td, J = 7.8, 3.6 Hz, intensity uninformative/see above, $[PPh_4]^+$: H^3), 7.51 (dd, J = 13.0, 7.8 Hz, intensity uninformative/see above, $[PPh_4]^+$: H^2), 7.11 (d, $J_{H,H}$ = 7.9 Hz, 1H, H^4), 6.15 (t, $J_{H,H}$ = 7.2 Hz, 1H, H^5), 5.55 (t, $J_{H,H}$ = 7.5 Hz, 1H, H^6), 5.21 (d, $J_{H,H}$ = 8.6 Hz, 1H, H^7), 3.47 (d, $J_{H,P}$ = 15.2 Hz, 1H, P^+-CH_3), 2.78 (s, 3H, Ar-CH_3). **^{31}P NMR** (162 MHz, dichloromethane-d_2) δ 23.2 (s, carbon satellites: $J_{P,C}$ = 89.5, 12.9, 10.2 Hz, intensity uninformative/see above), -14.7 (br.s.).

Appended spectra: NMR in dichloromethane-d_2: ^{1}H (*Figure S62*), ^{1}H NOE (*Figure S63*), ^{1}H 1D zTOCSY (*Figure S64*), ^{31}P{^{1}H} (*Figure S65*), ^{31}P (*Figure S66*).

S2.2 Deuterium-labeled ligand

Deuterium-labeled (TSMP-d_3)K_2 (**1-*d₃***) salt was prepared following the synthetic pathway for the corresponding proteo-analogue published by us elsewhere,[5] with the exception that we used CD_3I as a methylating agent at the stage of the synthesis of **9-*d₃*** (*Scheme S1*).

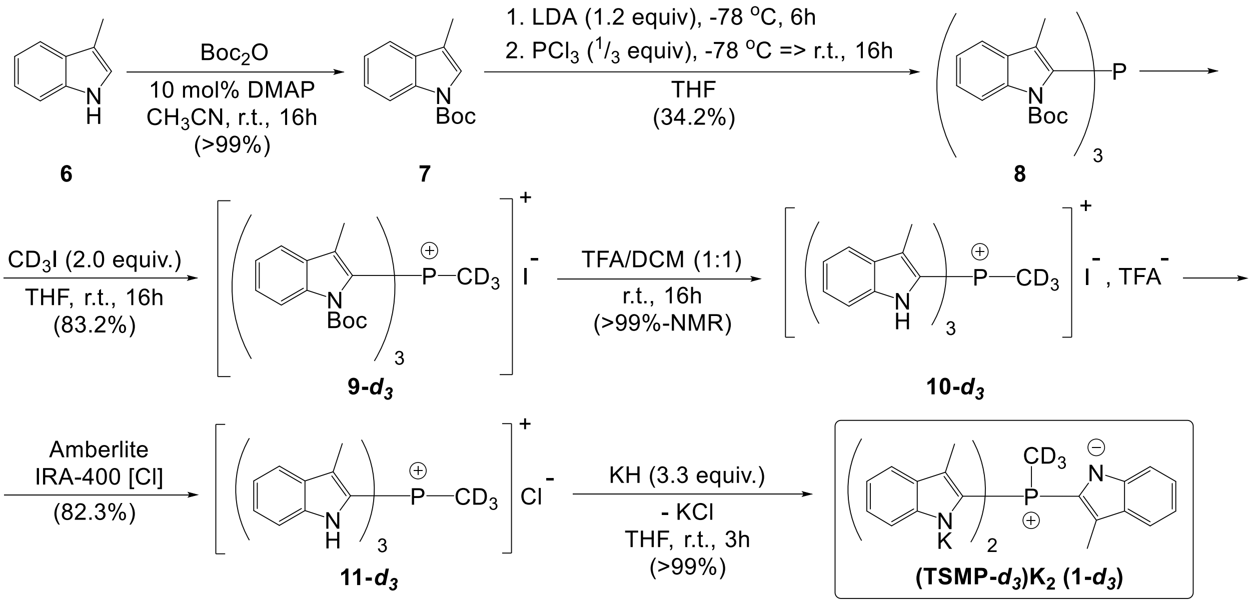

Scheme S1. Synthesis of a deuterium labeled dipotassium salt, (TSMP-d_3)K_2 (**1-*d₃***).

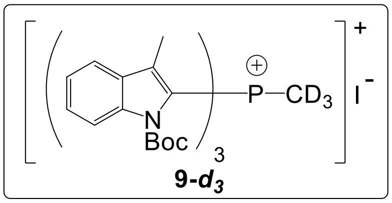

tris-(*N*-Boc-3-methylindol-2-yl)($^{2}H_3$)methylphosphonium iodide (9-*d₃*). Salt **9-*d₃*** was synthesized following a synthetic procedure for the analogous proteo-compound published by us elsewhere.[5] Yield: 1.54 g (83.2%).

NMR spectroscopic assignments are based on those for the proteo-analogue. In dichloromethane-d_2 solution, the compound exists as a ~1:0.15 mixture of rotamers. ^{1}H NMR signals from the major rotamer are labeled with an asterisk. NMR signal intensities of the minor rotamer were rounded up to be divisible by 0.15 with no remainder for the sake of simplicity; see the actual integral intensities in the appended spectra below. **^{1}H NMR** (400 MHz, dichloromethane-d_2) δ 8.18 (d, $J_{H,H}$ = 8.6 Hz, 0.05H, Ar-H), 8.12* (d, $J_{H,H}$ = 8.6 Hz, 1H, Ar-H), 8.05 (d, $J_{H,H}$ = 8.5 Hz, 0.05H, Ar-H), 7.86 (d, $J_{H,H}$ = 8.4 Hz, 0.05H, Ar-H), 7.69* (d, $J_{H,H}$ = 8.0 Hz, 1H, Ar-H), 7.63* (t, $J_{H,H}$ = 7.9 Hz, 1H, Ar-H), 7.44* (t, $J_{H,H}$ = 7.5 Hz, 1H, Ar-H), 2.38 (s, 0.15 H, Ar-CH_3), 2.15 (s, 0.15H, Ar-CH_3), 2.05 (s, 0.15H, Ar-CH_3), 1.97* (d, $J_{H,P}$ = 2.1 Hz, 3H, Ar-CH_3), 1.67 (s, 0.45H, ^{t}Bu), 1.55* (s, 9H, ^{t}Bu), 1.48 (s, 0.45H, ^{t}Bu), 1.15 (s, 0.45H, ^{t}Bu). **^{2}H NMR** (61 MHz, dichloromethane-h_2) δ 2.73 (br. s, 1H, P^{+}-CD_3). **^{13}C**

NMR (101 MHz, dichloromethane-d_2) δ 150.9 (s, C=O^{Boc}), 137.0 (d, $J_{C,P}$ = 5.6 Hz, indole-C9 or C8, or C3), 136.4 (d, $J_{C,P}$ = 12.2 Hz, indole-C8 or C9, or C3), 131.0 (d, $J_{C,P}$ = 14.4 Hz, indole-C3 or C8, or C9), 129.6 (s, C^{Ar}-H), 124.6 (s, C^{Ar}-H), 120.9 (s, C^{Ar}-H), 116.5 (s, C^{Ar}-H), 116.3 (d, $J_{C,P}$ = 128.7 Hz, indole-C2), 87.8 (s, qC$^{t\text{-}Bu}$), 27.9 (s, $CH_3^{t\text{-}Bu}$), 24.2 (br. s, P$^+$-CD_3), 10.1 (s, Ar-CH_3). **^{31}P NMR** (162 MHz, dichloromethane-d_2) δ -2.5 (s, carbon satellites: $J_{P,C}$ = 128.9, 79.9 Hz, 1P), -4.2 (br.s., 0.15P). **ATR-FTIR (neat)** ṽ (cm^{-1}): 422 (VW), 461 (VW), 602 (VW), 658 (VW), 701 (W), 722 (W), 741 (W), 760 (M), 822 (W), 846 (W), 886 (W), 1001 (W), 1026 (W), 1043 (VW), 1117 (S), 1151 (M), 1204 (W), 1240 (S), 1314 (S), 1367 (S), 1445 (W), 1478 (W), 1531 (W), 1586 (VW), 1721 (VS), 1737 (M), 2931 (VW), 2966 (W). **ESI-TOF-MS** in CH_3CN: found 739.4 [*M*]$^+$ (calcd. 739.4 [*M*]$^+$).

Appended spectra: NMR in dichloromethane-d_2: ^{1}H (*Figure S67*), ^{2}H (*Figure S68*), ^{13}C{^{1}H} (*Figure S69*), ^{31}P{^{1}H} (*Figure S70*); ATR-FTIR (neat, *Figure S71*).

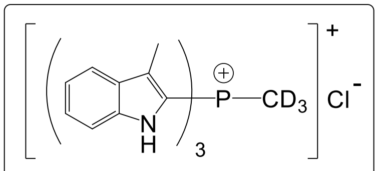

11-*d*$_3$

tris-(1*H*-3-methylindol-2-yl)($^2H_3$)methylphosphonium chloride (11-*d*$_3$). Salt **11-*d*$_3$** was synthesized following a synthetic procedure for the analogous proteo-compound published by us elsewhere.[5] Yield: 1.12 g (82.3%).

NMR spectroscopic assignments are based on the assignments for the proteo-analogue. **^{1}H NMR** (400 MHz, DMSO-d_6) δ 12.51 (s, 1H, NH), 7.73 (d, $J_{H,H}$ = 8.1 Hz, 1H, Ar-H), 7.60 (d, $J_{H,H}$ = 8.4 Hz, 1H, Ar-H), 7.40 (t, $J_{H,H}$ = 7.6 Hz, 1H, Ar-H), 7.19 (t, J = 7.6 Hz, 1H, Ar-H), 1.90 (s, 3H, Ar-CH_3). **^{2}H NMR** (61 MHz, DMSO-h_6) δ 3.23 (s, 1H, P$^+$-CD_3). **^{13}C NMR** (101 MHz, DMSO-d_6) δ 139.8 (d, $J_{C,P}$ = 11.4 Hz, indole-C8), 128.0 (d, $J_{C,P}$ = 13.0 Hz, indole C9 or C3), 126.9 (d, $J_{C,P}$ = 15.8 Hz, indole C3 or C9), 126.1 (s, C^{Ar}-H), 120.34 (s, C^{Ar}-H), 120.25 (s, C^{Ar}-H), 112.7 (s, C^{Ar}-H), 108.6 (d, $J_{C,P}$ = 120.2 Hz, indole-C2), 12.1 (br.s., P$^+$-CD_3), 8.2 (s, Ar-CH_3). **^{31}P NMR** (162 MHz, DMSO-d_6) δ -14.2 (s, carbon satellites: $J_{P,C}$ = 120,1, 58.2 Hz, 1H). **ATR-FTIR (neat)** ṽ (cm^{-1}): 425 (S), 453 (W), 515 (W), 557 (W), 607 (W), 703 (S), 741 (VS), 812 (S), 892 (W), 942 (W), 1024 (M), 1042 (M), 1130 (M), 1151 (M), 1203 (S), 1238 (M), 1295 (W), 1331 (S), 1383 (W), 1430 (M), 1513 (S), 1578 (W), 1616 (W), 2118 (W), 2220 (W), 2847 (S), 2914 (S), 2973 (S), 3019 (S), 3058 (S), 3444 (W). **ESI-TOF-MS** in CH_3CN: found 439.2 [*M*]$^+$ (calcd. 439.2 [*M*]$^+$).

Appended spectra: NMR in DMSO-d_6: ^{1}H (*Figure S72*), ^{2}H (*Figure S73*), ^{13}C{^{1}H} (*Figure S74*), ^{31}P{^{1}H} (*Figure S75*); ATR-FTIR (neat, *Figure S76*).

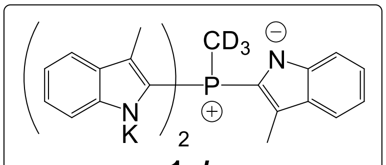

1-*d*$_3$

(TSMP-*d*$_3$)K_2 (1-*d*$_3$). Salt **1-*d*$_3$** was synthesized following a synthetic procedure for the analogous proteo-compound using KH as a base and published by us elsewhere.[5] Deprotonation occurs quantitatively with the yield of 1.45 g. Due to the presence of the K^+ cations, **1-*d*$_3$** always retains a certain of THF,

which, in this particular batch, was identified to be 16.8%, using quantitative ^{1}H NMR experiments.

NMR spectroscopic assignments are based on the assignments for the proteo-analogue. **^{1}H NMR** (400 MHz, acetonitrile-*d*$_3$) δ 7.42 (d, $J_{H,H}$ = 8.0 Hz, 1H, Ar-H), 7.31 (d, $J_{H,H}$ = 8.3 Hz, 1H, Ar-H), 6.85 (t, $J_{H,H}$ = 7.2 Hz, 1H, Ar-H), 6.75 (t, $J_{H,H}$ = 7.1 Hz, 1H, Ar-H), 2.08 (d, $J_{H,P}$ = 1.2 Hz, 3H, Ar-CH_3). **^{2}H NMR** (61 MHz, acetonitrile-*h*$_3$) δ 2.57 (s, 1H, P^+-CD_3). **^{13}C NMR** (101 MHz, acetonitrile-*d*$_3$) δ 149.6 (d, $J_{C,P}$ = 24.2 Hz, indole-C8), 132.2 (d, $J_{C,P}$ = 10.9 Hz, indole-C9 or C3), 128.5 (d, $J_{C,P}$ = 117.6 Hz, indole-C2), 119.3 (s, C^{Ar}-H), 119.0 (d, J = 0.9 Hz, C^{Ar}-H), 118.6 (d, J = 1.0 Hz, C^{Ar}-H), 117.2 (d, $J_{C,P}$ = 26.4 Hz, indole-C3 or C9), 115.9 (d, J = 1.3 Hz, C^{Ar}-H), 14.0 (m, J = 32.0, 63.5 Hz, P^+-CD_3), 10.3 (s, Ar-CH_3). **^{31}P NMR** (162 MHz, acetonitrile-*d*$_3$) δ -8.2 (s).

<u>Appended spectra:</u> NMR in acetonitrile-*d*$_3$: ^{1}H (*Figure S77*), ^{2}H (*Figure S78*), ^{13}C{^{1}H} (*Figure S79*), ^{31}P{^{1}H} (*Figure S80*).

S2.3 Deuterium-labeled complexes

Deuterium-labeled complexes [(TSMP-*d*$_3$)$_2$Fe^{III}]K (**3a-*d*$_6$**), [(TSMP-*d*$_3$)$_2$Fe^{III}]PPh_4 (**3b-*d*$_6$**) and [(TSMP-*d*$_3$)$_2$Fe^{IV}] (**4-*d*$_6$**), were prepared following the procedures for the respective proteo-analogues **3a**, **3b** and **4** (see *Section S2.1*). Even though compound **3a-*d*$_6$** was synthesized by *in situ* oxidation of the [(TSMP-*d*$_3$)$_2$Fe^{II}]K_2 (**2a-*d*$_6$**) intermediate, the tretrakis(benzo-15-crown-5)adduct of the latter, [(TSMP-*d*$_3$)$_2$Fe^{II}][(B15C5)$_2$K]$_2$ (**2b-*d*$_6$**), was still isolated for the sake of completeness. It can be compared with the corresponding proteo-analogue published by us elsewhere.[5]

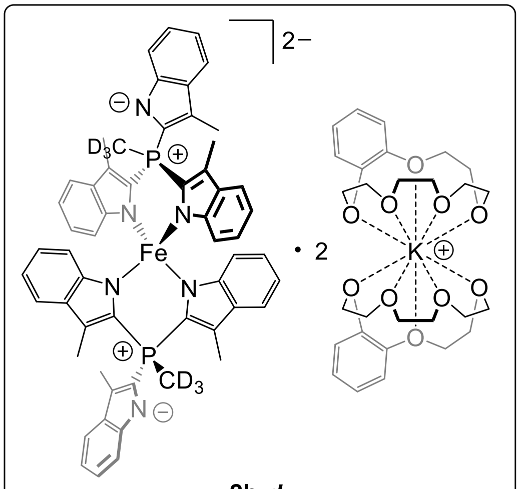
2b-*d*$_6$

[(TSMP-*d*$_3$)$_2$Fe^{II}][(B15C5)$_2$K]$_2$ (2b-*d*$_6$). Salt **2b-*d*$_6$** was synthesized following a synthetic procedure for the analogous proteo-compound published by us elsewhere.[5] Yield: 0.315 g (42.5%).

Our previous studies suggest that the proteo-analogue **2b** exists in solution in more than one form,[5] therefore the assignment of paramagnetic ^{1}H NMR signals is expected to be highly non-trivial. Hence, we did not expend effort for establishing the signal identities.

^{1}H NMR (400 MHz, Acetonitrile-*d*$_3$) δ 66.3 (br. s.), 21.6 (br. s.), 14.5 (br. s.), 6.88 (br. s., Ar-H of benzo-15-crown-5), 6.72 (br. s., Ar-H of benzo-15-crown-5), 6.1 (br. s.), 4.42 – 2.97 (m, aliphatic C-H of benzo-15-crown-5), -0.6 (br. s.), -0.9 (br. s.), -6.1 (br. s., P^+-CH_xD_{3-x}, due to ^{1}H isotopic impurities in the starting CD_3I). **^{2}H NMR** (61 MHz, Acetonitrile-*h*$_3$) δ -6.0 (br. s., P^+-CD_3). **^{31}P NMR** (162 MHz, Acetonitrile-*d*$_3$) δ 104.8 (br. s.). **ATR-FTIR** (neat) ṽ (cm^{-1}): 431 (M), 563 (W), 612 (W), 708 (M), 740 (S), 813 (M), 853 (W), 937 (M), 1044 (S), 1076 (M), 1098 (S), 1122 (VS), 1216 (S),

1252 (VS), 1296 (M), 1336 (M), 1362 (M), 1454 (M), 1504 (S), 1597 (W), 2863 (M), 2904 (M), 3041 (W). **ESI-TOF-MS** spectra in CH_3CN recorded in a negative mode show a product of one-electron oxidation of **2b-*d*$_6$**, which is in line with its high air-sensitivity: 928.3 [M]$^-$ (calcd. 928.3 [M]$^-$).

Appended spectra: NMR in acetonitrile-d_3: ^{1}H (*Figure S81*), ^{2}H (*Figure S82*), ^{31}P (*Figure S83*); ATR-FTIR (neat, *Figure S84*).

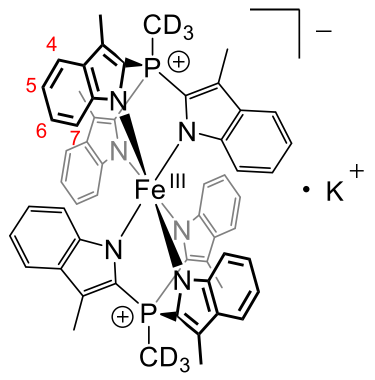

3a-*d*$_6$

[(TSMP-*d*$_3$)$_2$Fe^{III}]K (3a-*d*$_6$). Salt **3a-*d*$_6$** was synthesized following a synthetic procedure for the analogous proteo-compound **3a** (see *Section S2.1*). Yield: 0.215 g (58.3%).

All measurements described below were performed at 298K. The assignments of ^{1}H NMR signals are discussed in detail in *Section S13.2.1*. **^{1}H NMR** (400 MHz, acetonitrile-d_3) δ 70.3 (br. s., 3H, Ar-CH_3), 30.1 (br. s., 1H, H^4), 11.8 (br. s., 0.09H, P^+-CH_xD_{3-x}, due to ^{1}H isotopic impurities in the starting CD_3I), -4.5 (br. s., 1H, H^6), -7.1 (br. s., 1H, H^5). **^{2}H NMR** (61 MHz, acetonitrile-h_3) δ 11.6 (br. s., 1H, P^+-CD_3). **^{31}P NMR** (162 MHz, acetonitrile-d_3) δ -237.5 (br. s.). **ESI-TOF-MS** spectra in CH_3CN: 928.3 [M]$^-$ (calcd. 928.3 [M]$^-$).

Appended spectra: NMR in acetonitrile-d_3: ^{1}H (*Figure S85*), ^{2}H (*Figure S86*), ^{31}P (*Figure S87*).

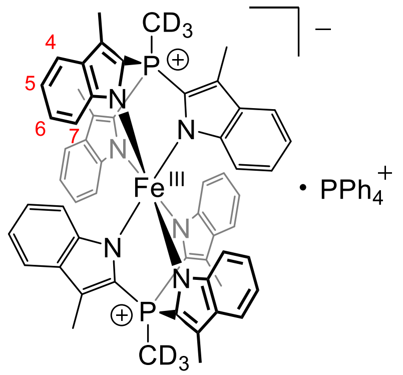

3b-*d*$_6$

[(TSMP-*d*$_3$)$_2$Fe^{III}]PPh$_4$ (3b-*d*$_6$). Salt **3b-*d*$_6$** was synthesized following a synthetic procedure for the analogous proteo-compound **3b** (see *Section S2.1*). Yield: 0.083 g (quantitative yield).

All measurements described below were performed at 298K. The assignments of the ^{1}H NMR signals are discussed in detail in *Section S13.2.1*. While the ^{1}H NMR spectra show the increased intensity of the $[PPh_4]^+$ signals with respect to what in needed to match the stoichiometry of **3b-*d*$_6$** (see *Appended spectra*), the ^{31}P spectra display the expected 1:2 intensity ratio. A similar situation was observed for the proteo-analogue **3b** (see *Section S2.1*), which we connect with problematic base line correction due to the presence of an intense dichloromethane signal. **^{1}H NMR** (400 MHz, dichloromethane-d_2) δ 68.6 (br. s., 3H, Ar-CH_3), 27.7 (br. s., 1H, H^4), 8.55 – 6.51 (m, intensity unreliable/see above, $[PPh_4]^+$), -4.5 (br. s., 1H, H^6), -6.8 (br. s., 1H, H^5). **^{2}H NMR** (61 MHz, dichloromethane-h_2) δ 12.2 (br. s., 1H, P^+-CH_3). **^{31}P NMR** (162 MHz, dichloromethane-d_2) δ 23.1 (s, 1P, $[PPh_4]^+$), -216.7 (br. s., 2P, P^+-CD_3).

Appended spectra: NMR in dichloromethane-d_2: ^{1}H (*Figure S88*), ^{2}H (*Figure S89*), ^{31}P (*Figure S90*).

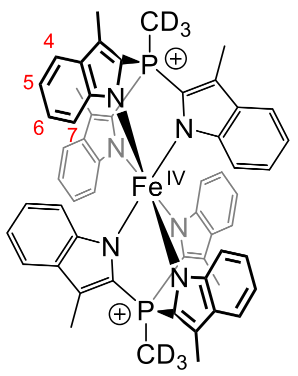

4-*d*$_6$

[(TSMP-*d*$_3$)$_2$Fe^{IV}] (4-*d*$_6$). Complex **4-*d*$_6$** was synthesized following a synthetic procedure for the analogous proteo-compound **4** (see *Section S2.1*). Yield: 0.040 g (92.4%).

All measurements described below were performed at 298K. The assignments of ^{1}H NMR signals are discussed in detail in *Section S13.3.1*. ^{31}P NMR signals were not observed in the probed spectral range (-500 to +500 ppm). **^{1}H NMR** (400 MHz, dichloromethane-d_2) δ 145.9 (br. s., 3H, Ar-CH$_3$), -12.8 (s, 1H, H^5), -22.2 (s, 1H, H^6), -27.5 (br. s., 1H, H^7), -32.0 (br. s., 1H, H^4). **^{2}H NMR** (61 MHz, dichloromethane-h_2) δ 6.98 (br. s., 1H, P$^+$-CH$_3$). **ESI-TOF-MS** spectra in CH$_3$CN recorded in a negative mode show a product of one-electron reduction of **4**: 928.3 [*M*]$^-$ (calcd. 928.3 [*M*]$^-$).

Appended spectra: NMR in dichloromethane-d_2: ^{1}H (*Figure S91*), ^{2}H (*Figure S92*).

S2.4 Additional experiments

Attempted synthesis of [(TSMP)$_2$Fe^{III}]K (3a) using FeCl$_3$. A solution of FeCl$_3$ (0.0055 g, 0.035 mmol, 1.0 equiv.) in acetonitrile (4.0 ml) was added dropwise over a minute to a solution of TSMPK$_2$ salt (**1**) (contains 28.6 wt% of THF, 0.050 g, 0.070 mmol, 2.1 equiv.) in acetonitrile (8.0 ml), which resulted in deep-blue coloration of the mixture. The reaction was allowed to stir for 4h, upon which it was filtered, and the solvent was evaporated *in vacuo*. ^{1}H NMR spectrum in acetonitrile-d_3 shows the presence of multiple paramagnetic species, one which is target complex **3a**, although in rather low relative abundance.

S3 X-ray crystal structure determinations

CCDC 2245041-2245045 contain the supplementary crystallographic data for this paper. These data can be obtained free of charge from The Cambridge Crystallographic Data Centre *via* www.ccdc.cam.ac.uk/data_request/cif.

[(TSMP)$_2$Fe^{III}]K (3a). $C_{60}H_{54}FeKN_8P_2 \cdot 2C_2H_3N$, Fw = 1126.11, black block, 0.44 × 0.23 × 0.17 mm^3, triclinic, $P\overline{1}$ (no. 2), a = 10.9377(4), b = 14.5055(5), c = 18.1385(5) Å, α = 96.885(2), β = 97.281(2), γ = 101.149(1) °, V = 2769.74(15) Å^3, Z = 2, D_x = 1.350 g/cm^3, μ = 0.46 mm^{-1}. The diffraction experiment was performed on a Bruker Kappa ApexII diffractometer with sealed tube and Triumph monochromator (λ = 0.71073 Å) at a temperature of 150(2) K up to a resolution of $(\sin \theta/\lambda)_{max}$ = 0.65 Å^{-1}. The crystal appeared to

be twinned with a twofold rotation about uvw=[1,0,0] as twin operation. Consequently, two orientation matrices were used for the intensity integration with the Eval15 software.[10] The integration results were written in HKLF5 format.[11] A multi-scan absorption correction and scaling was performed with TWINABS[12,13] (correction range 0.63-0.75). A total of 106370 reflections was measured, 12737 reflections were unique (R_{int} = 0.026), 11548 reflections were observed [I>2σ(I)]. The structure was solved with Patterson superposition methods using SHELXT.[14] Structure refinement was performed with SHELXL-2018[15] on F^2 of all reflections. Non-hydrogen atoms were refined freely with anisotropic displacement parameters. All hydrogen atoms were located in difference Fourier maps and refined with a riding model. 719 Parameters were refined with no restraints. R1/wR2 [I > 2σ(I)]: 0.0322 / 0.0902. R1/wR2 [all refl.]: 0.0360 / 0.0927. S = 1.044. Twin fraction BASF=0.1631(7). Residual electron density between -0.41 and 0.44 e/Å^3. Geometry calculations and checking for higher symmetry was performed with the PLATON program.[16]

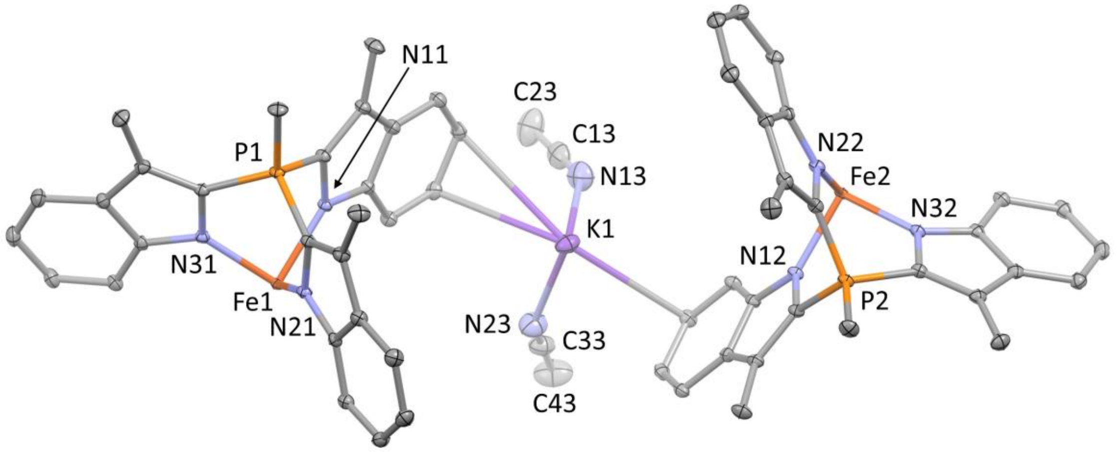

Figure S1. Asymmetric unit in the crystal of [$(TSMP)_2Fe^{III}$]K (**3a**). Iron atoms are on special positions, therefore only half of the coordination environment is shown. Displacement ellipsoids are drawn at the 30% probability level. Hydrogen atoms, potassium cation and acetonitrile solvent molecules are omitted for clarity. Symmetry code *i*: 1-x, 1-y, -z. Selected bond distances (Å) and angles (°): Fe1-N11 1.9885(11), Fe1-N21 2.0020(12), Fe1-N31 1.9805(12), N11-Fe1-N21 91.11(5), N21-Fe1-N31 90.70(5), N31-Fe1-N11 90.73(5); Fe2-N12 1.9889(12), Fe2-N22 1.9885(12), Fe2-N32 1.9552(12), N12-Fe2-N22 91.56(5), N22-Fe2-N32 90.44(5), N32-Fe2-N12 91.16(5).

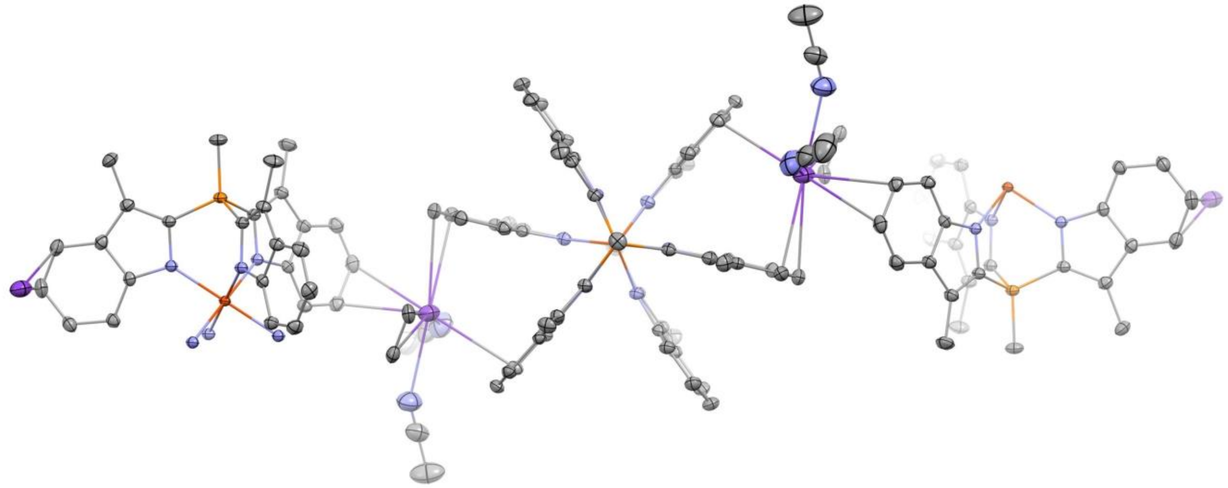

Figure S2. Coordination polymer in the [0,1,-1] direction in the crystal structure of $[(TSMP)_2Fe^{III}]K$ (**3a**). Displacement ellipsoids are drawn at the 30% probability level. Hydrogen atoms and non-coordinated acetonitrile molecules are omitted for clarity. Symmetry codes *i*: 1-x, -y, 1-z; *ii*: 1-x, 1-y, -z.

$[(TSMP)_2Fe^{III}]PPh_4$ (3b). $[C_{56}H_{48}FeN_6P_2](C_{24}H_{20}P)$ + disordered solvent, Fw = 1262.16,[a] black plate, 0.36 × 0.16 × 0.05 mm^3, triclinic, $P\overline{1}$ (no. 2), a = 14.1299(3), b = 15.4113(4), c = 17.9403(3) Å, α = 85.134(1), β = 83.361(1), γ = 85.674(1) °, V = 3857.99(13) Å^3, Z = 2, D_x = 1.087 g/cm^3,[a] μ = 0.30 mm^{-1}.[a] The diffraction experiment was performed on a Bruker Kappa ApexII diffractometer with sealed tube and Triumph monochromator (λ = 0.71073 Å) at a temperature of 150(2) K up to a resolution of $(\sin \theta/\lambda)_{max}$ = 0.65 Å^{-1}. The Eval15 software[10] was used for the intensity integration. A multi-scan absorption correction and scaling was performed with SADABS[12,13] (correction range 0.67-0.75). A total of 85393 reflections was measured, 17698 reflections were unique (R_{int} = 0.068), 12087 reflections were observed [I>2σ(I)]. The structure was solved with Patterson superposition methods using SHELXT.[14] Structure refinement was performed with SHELXL-2018[15] on F^2 of all reflections. The crystal structure contains voids (957 Å^3 / unit cell) filled with disordered solvent molecules. Their contribution to the structure factors was secured by back-Fourier transformation using the SQUEEZE algorithm[17] resulting in 285 electrons / unit cell. Non-hydrogen atoms were refined freely with anisotropic displacement parameters. One phenyl ring in the PPh_4 ion was refined with a disorder model. All hydrogen atoms were introduced in calculated positions and refined with a riding model. 877 Parameters were refined with 280 restraints (distances, angles, displacement parameters and ring flatness in PPh_4). R1/wR2 [I > 2σ(I)]: 0.0483 / 0.1174. R1/wR2 [all refl.]: 0.0787 / 0.1291. S = 1.033. Residual electron density between -0.41 and 0.76 e/Å^3. Geometry calculations and checking for higher symmetry was performed with the PLATON program.[16]

---

[a] Derived values do not contain the contribution of the disordered solvent molecules.

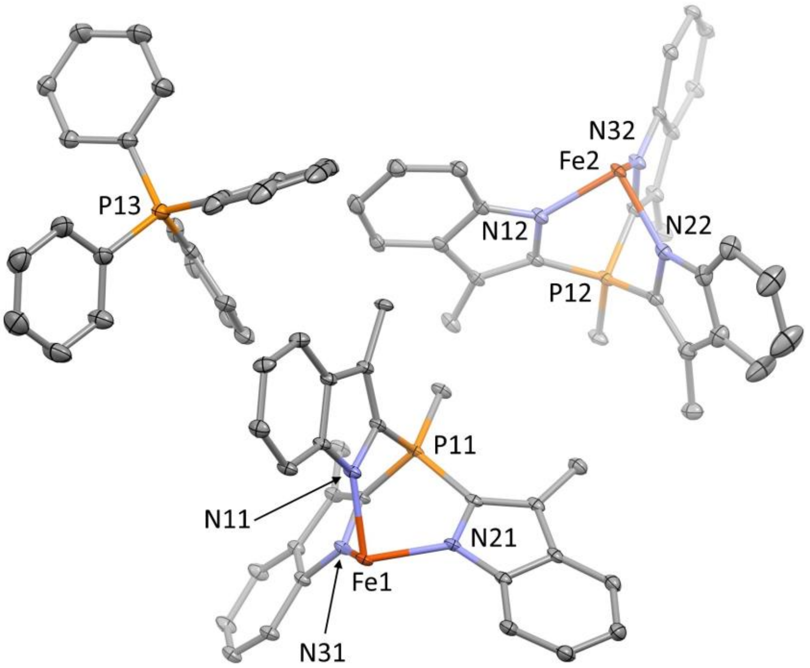

Figure S3. Asymmetric unit in the crystal of $[(TSMP)_2Fe^{III}]PPh_4$ (**3b**). Iron atoms are on special positions, therefore only half of the coordination environment is shown. Displacement ellipsoids are drawn at the 30% probability level. Hydrogen atoms, PPh_4 cation and severely disordered solvent molecules are omitted for clarity. Symmetry code *i*: -x, 1-y, 1-z. Selected bond distances (Å) and angles (°): Fe1-N11 1.9717(16), Fe1-N21 2.0025(17), Fe1-N31 1.9802(17), N11-Fe1-N21 90.52(7), N21-Fe1-N31 90.76(7), N31-Fe1-N11 91.24(7); Fe2-N12 2.0369(17), Fe2-N22 2.0313(18), Fe2-N32 2.0343(17), N12-Fe2-N22 89.64(7), N22-Fe2-N32 90.17(7), N32-Fe2-N12 90.06(7).

$[(TSMP)_2Fe^{III}][(B15C5)_2K]$ (3c). $[C_{56}H_{48}FeN_6P_2](C_{28}H_{40}KO_{10}) \cdot 1.5(C_5H_5N)$, Fw = 1617.14, dark-blue needle, 0.34 × 0.07 × 0.05 mm^3, triclinic, $P\overline{1}$ (no. 2), a = 15.1846(9), b = 17.2956(8), c = 17.6868(7) Å, α = 111.250(2), β = 101.333(2), γ = 105.495(2) °, V = 3943.1(3) Å^3, Z = 2, D_x = 1.362 g/cm^3, μ = 0.35 mm^{-1}. The diffraction experiment was performed on a Bruker Kappa ApexII diffractometer with sealed tube and Triumph monochromator (λ = 0.71073 Å) at a temperature of 100(2) K up to a resolution of $(\sin\theta/\lambda)_{max}$ = 0.61 Å^{-1}. The Eval15 software[10] was used for the intensity integration. A numerical absorption correction and scaling was performed with SADABS[12,13] (correction range 0.73-1.00). A total of 61310 reflections was measured, 14678 reflections were unique (R_{int} = 0.092), 9168 reflections were observed [I>2σ(I)]. The structure was solved with Patterson superposition methods using SHELXT.[14] Structure refinement was performed with SHELXL-2018[15] on F^2 of all reflections. Non-hydrogen atoms were refined freely with anisotropic displacement parameters. One pyridine molecule was disordered on an inversion center. All hydrogen atoms were introduced in calculated positions and refined with a riding model. 1056 Parameters were refined with 205 restraints (distances, angles, displacement parameters and ring flatness in the pyridine molecules). R1/wR2 [I > 2σ(I)]: 0.0516 / 0.1067. R1/wR2 [all refl.]: 0.1058 / 0.1267. S =

1.010. Residual electron density between -0.50 and 0.47 e/Å^3. Geometry calculations and checking for higher symmetry was performed with the PLATON program.[16]

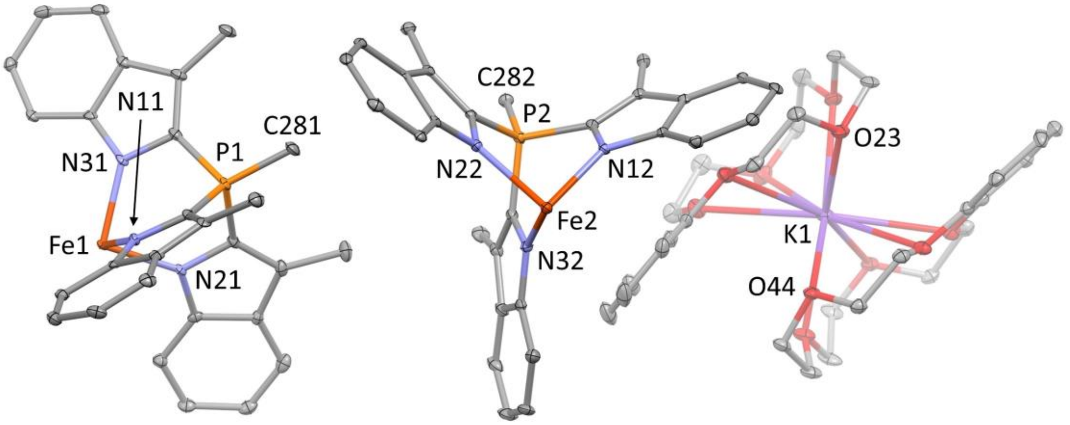

Figure S4. Asymmetric unit in the crystal of [$(TSMP)_2Fe^{III}$][$(B15C5)_2K$] (**3c**). Iron atoms are on special positions, therefore only half of the coordination environment is shown. Displacement ellipsoids are drawn at the 30% probability level. Hydrogen atoms, K[benzo-15-crown-5] cation and pyridine solvent molecules are omitted for clarity. Symmetry code *i*: -x, 1-y, -z. Selected bond distances (Å) and angles (°): Fe1-N11 1.970(2), Fe1-N21 1.976(2), Fe1-N31 2.008(2), N11-Fe1-N21 90.67(10), N21-Fe1-N31 91.00(10), N31-Fe1-N11 90.94(10); Fe2-N12 2.136(2), Fe2-N22 2.136(2), Fe2-N32 2.123(3), N12-Fe2-N22 87.56(9), N22-Fe-N32 87.51(9), N32-Fe2-N12 87.69(9).

[$(TSMP)_2Fe^{IV}$] (4). $C_{56}H_{48}FeN_6P_2 \cdot 6(C_5H_5N)$, Fw = 1397.39, black plate, 0.23 × 0.20 × 0.05 mm^3, triclinic, $P\overline{1}$ (no. 2), a = 11.5781(6), b = 12.1341(10), c = 14.4610(10) Å, α = 106.027(3), β = 94.678(3), γ = 114.093(2) °, V = 1738.3(2) Å^3, Z = 1, D_x = 1.335 g/cm^3, μ = 0.32 mm^{-1}. The diffraction experiment was performed on a Bruker Kappa ApexII diffractometer with sealed tube and Triumph monochromator (λ = 0.71073 Å) at a temperature of 150(2) K up to a resolution of $(\sin \theta/\lambda)_{max}$ = 0.53 Å^{-1}. The Eval15 software[10] was used for the intensity integration. A multi-scan absorption correction and scaling was performed with SADABS[12,13] (correction range 0.58-0.74). A total of 22469 reflections was measured, 4263 reflections were unique (R_{int} = 0.104), 2766 reflections were observed [I>2σ(I)]. The structure was solved with Patterson superposition methods using SHELXT.[14] Structure refinement was performed with SHELXL-2018[15] on F^2 of all reflections. Non-hydrogen atoms were refined freely with anisotropic displacement parameters. All hydrogen atoms were introduced in calculated positions and refined with a riding model. 461 Parameters were refined with no restraints. R1/wR2 [I > 2σ(I)]: 0.0745 / 0.2036. R1/wR2 [all refl.]: 0.1229 / 0.2310. S = 1.167. Residual electron density between -0.32 and 0.57 e/Å^3. Geometry calculations and checking for higher symmetry was performed with the PLATON program.[16]

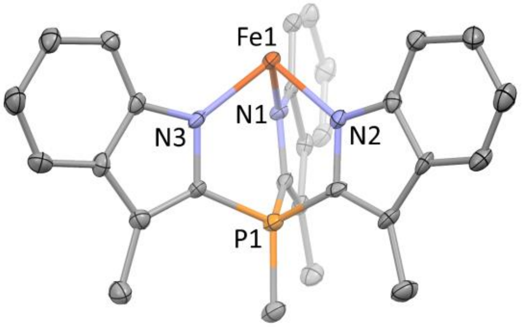

Figure S5. Asymmetric unit in the crystal of [$(TSMP)_2Fe^{IV}$] (**4**). Iron atom is on a special position, therefore only half of the coordination environment is shown. Displacement ellipsoids are drawn at the 30% probability level. Hydrogen atoms and pyridine solvent molecules are omitted for clarity. Symmetry code *i*: 1-x, 1-y, 1-z. Selected bond distances (Å) and angles (°): Fe1-N1 1.966(6), Fe1-N2 1.975(5), Fe1-N3 1.966(6), N1-Fe1-N2 91.6(2), N2-Fe1-N3 91.0(2), N3-Fe1-N1 90.7(2).

[$(TSMP)_2Ga^{III}$]K (5a). $C_{58}H_{51}GaKN_7P_2 \cdot C_4H_{10}O \cdot C_2H_3N$, Fw = 1131.99, colourless plate, 0.45 × 0.25 × 0.11 mm^3, triclinic, $P\overline{1}$ (no. 2), a = 11.0948(4), b = 14.7420(5), c = 17.6613(9) Å, α = 98.145(2), β = 99.810(3), γ = 100.918(2) °, V = 2749.36(19) Å^3, Z = 2, D_x = 1.367 g/cm^3, μ = 0.69 mm^{-1}. The diffraction experiment was performed on a Bruker Kappa ApexII diffractometer with sealed tube and Triumph monochromator (λ = 0.71073 Å) at a temperature of 150(2) K up to a resolution of $(\sin \theta/\lambda)_{max}$ = 0.65 Å^{-1}. The crystal appeared to be broken in several fragments. The three major fragments were integrated using three orientation matrices with the Eval15 software.[10] The integration results were written in HKLF5 format.[11] A multi-scan absorption correction and scaling was performed with TWINABS[12,13] (correction range 0.66-0.75). A total of 85413 reflections was measured, 14706 reflections were unique (R_{int} = 0.038), 12207 reflections were observed [$I>2\sigma(I)$]. The structure was solved with Patterson superposition methods using SHELXT.[14] Structure refinement was performed with SHELXL-2018[15] on F^2 of all reflections. Non-hydrogen atoms were refined freely with anisotropic displacement parameters. The diethyl ether solvent molecule was refined with a disorder model. All hydrogen atoms were introduced in calculated positions and refined with a riding model. 722 Parameters were refined with 68 restraints (distances, angles and displacement parameters in the disordered diethyl ether). R1/wR2 [$I > 2\sigma(I)$]: 0.0433 / 0.1191. R1/wR2 [all refl.]: 0.0541 / 0.1275. S = 1.039. Scale factors of additional fragments, BASF=0.193(4) and 0.074(3). Residual electron density between -1.41 and 1.05 e/Å^3. Geometry calculations and checking for higher symmetry was performed with the PLATON program.[16]

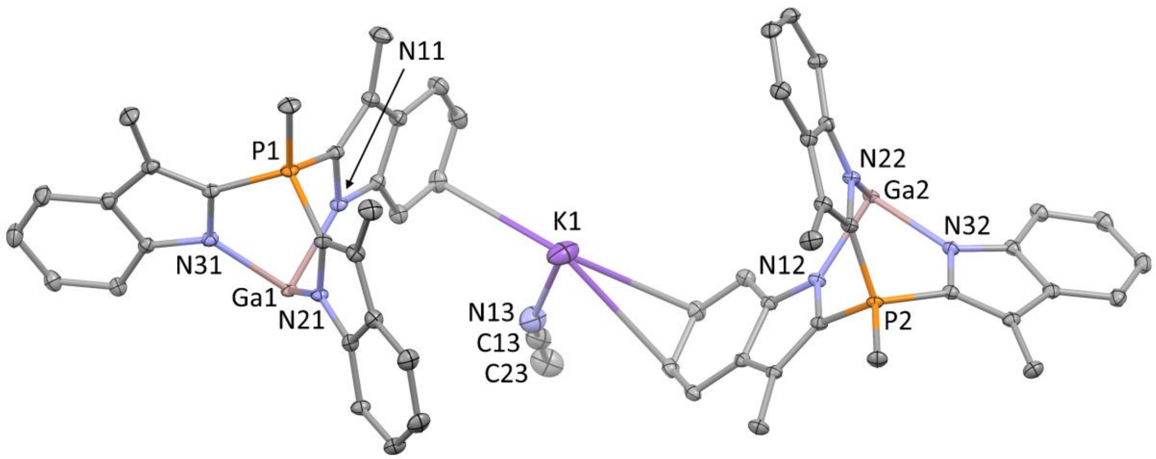

Figure S6. Asymmetric unit in the crystal of [(TSMP)$_2$Ga^{III}]K (**5a**). Gallium atoms are on special positions, therefore only half of the coordination environment is shown. Displacement ellipsoids are drawn at the 30% probability level. Hydrogen atoms, potassium cation and solvent molecules are omitted for clarity. Symmetry code *i*: 1-x, 1-y, -z. Selected bond distances (Å) and angles (°): N31-Ga1 2.0470(18), N11-Ga1 2.075(2), N21-Ga1 2.0845(19), N31-Ga1-N11 89.11(7), N31-Ga1-N21 88.93(7), N21-Ga1-N11 89.17(8); Ga2-N22 2.0783(18), Ga2-N32 2.0636(19), Ga2-N12 2.0691(19), N12-Ga2-N22 89.89(7), N12-Ga2-N32 88.78(8), N32-Ga2-N22 88.69(7).

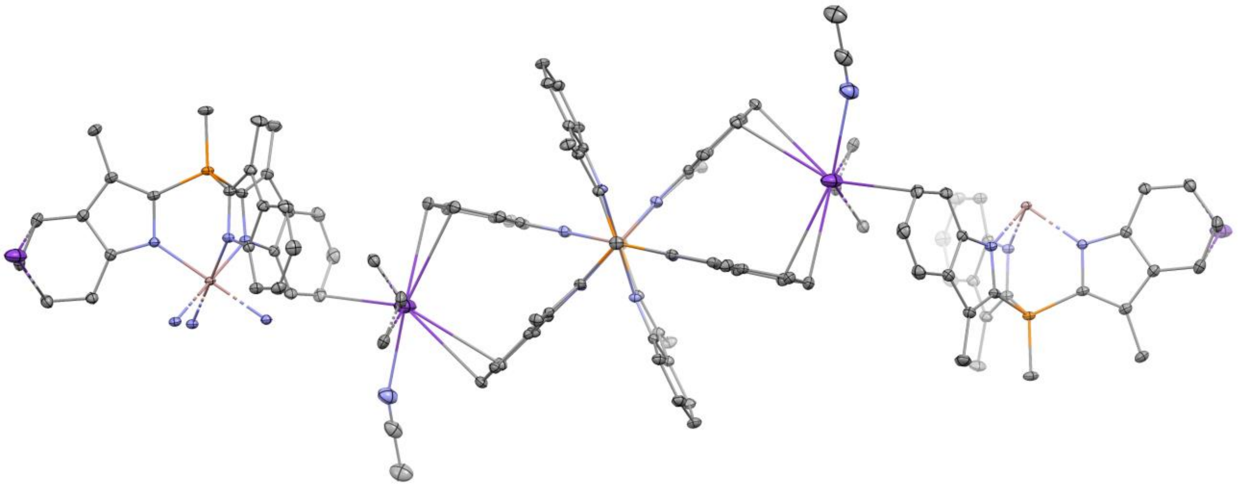

Figure S7. Coordination polymer in the [0,1,-1] direction in the crystal structure of [(TSMP)$_2$Ga^{III}]K (**5a**). Displacement ellipsoids are drawn at the 30% probability level. Hydrogen atoms and non-coordinated acetonitrile and diethyl ether molecules are omitted for clarity. Symmetry codes *i*: 1-x, -y, 1-z; *ii*: 1-x, 1-y, -z.

S4 Computational details

All calculations were performed using ORCA 4.2.[18,19] The absence of imaginary frequencies was confirmed for all structures. Optimized geometries are given in *Section S16*.

Ground-state electronic structure calculations and Mössbauer parameter predictions were performed at the B3LYP-D3BJ/def2-TZVP (CP(PPP) for Fe) level of theory using a geometry

optimized at the BP86-D3BJ/def2-TZVP level. The BP86 functional generally provides accurate geometries at affordable computational cost, and no relevant discrepancy with the crystallographic geometry was observed. The hybrid B3LYP functional generally reproduces Mössbauer parameters well with appropriate calibration.[20]

Hyperfine coupling constants were calculated using B3LYP, TPSSH and PBE0 functionals with D3BJ dispersion correction and def2-TZVPP basis set. The respective geometries were optimized with the same functionals (PBE was used instead of PBE0 to save computational time), same dispersion correction and def2-TZVP basis set. The hyperfine coupling constant calculated using PBE0 showed the best agreement with the experiment and, therefore, were chosen for further discussion.

TD-DFT spectra were calculated using B3LYP, TPSSH and PBE functionals along with D3BJ dispersion correction and 6-31G(d,p) basis set due to limitations of the computational infrastructure. TPSSH showed the best agreement with the experiment and, therefore, was chosen for further discussion. TDDFT spectra and the corresponding Natural Transition Orbitals (NTOs) were calculated for the first 50 excitations.

S5 Cyclic voltammetry

S5.1 Experimental details

Cyclic voltammetry (CV) was performed using an IVIUM Technologies Vertex Potentiostat/Galvanostat. All measurements were performed in 0.1 M $^{n}Bu_4NPF_6$ acetonitrile electrolyte in a glovebox under dinitrogen atmosphere with strict exclusion of air and moisture.

S5.2 Cyclic voltammetry of [(TMSP)$_2$Fe^{III}]K (3a)

Cyclic voltammograms (CVs) measured for the [(TSMP)$_2$Fe^{II}][(B15C5)$_2$K]$_2$ (**2b**) and [(TSMP)$_2$Fe^{III}]K (**3a**) salts show common features but also a few differences (*Figure S8*). Specifically, events **B** and **C**, which correspond to the [(TSMP)$_2$Fe^{III}]$^-$ (**3**) / [(TSMP)$_2$Fe^{IV}]^0 (**4**) redox couple, are identical for both **2b** and **3a**, and are centered at $E_{1/2}$= -0.41 V *vs.* Fc/Fc$^+$. Similarly to **2b** (see main text), **B** and **C** in **3a** represent a quasi-reversible electron transfer[21] as shown by the linear dependence of peak current (i_P) *vs.* square root of the scan rate ($v^{1/2}$; *Figure S9*, *right panel*) and strong dependence of the peak separation on the scan rate.

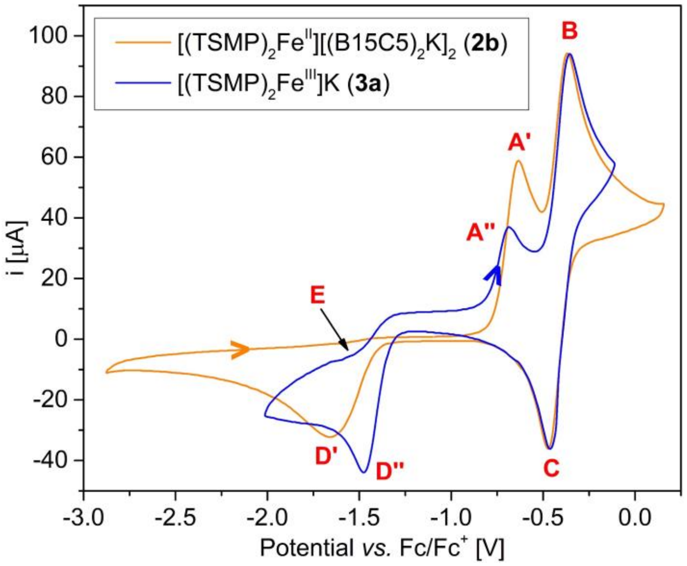

Figure S8. Overlay of CVs measured for **2b** and **3a**. The measurements were performed in ca. 8 mM solution in 0.1 M $^{n}Bu_4NPF_6$ acetonitrile electrolyte. Potentials are referenced with respect to Fc/Fc^+ redox couple. The CV of **2b** can be repeated at least a hundred times at the rates within 25–250 mV/s with no visible changes, which implies chemical reversibility of the redox cycle it represents.

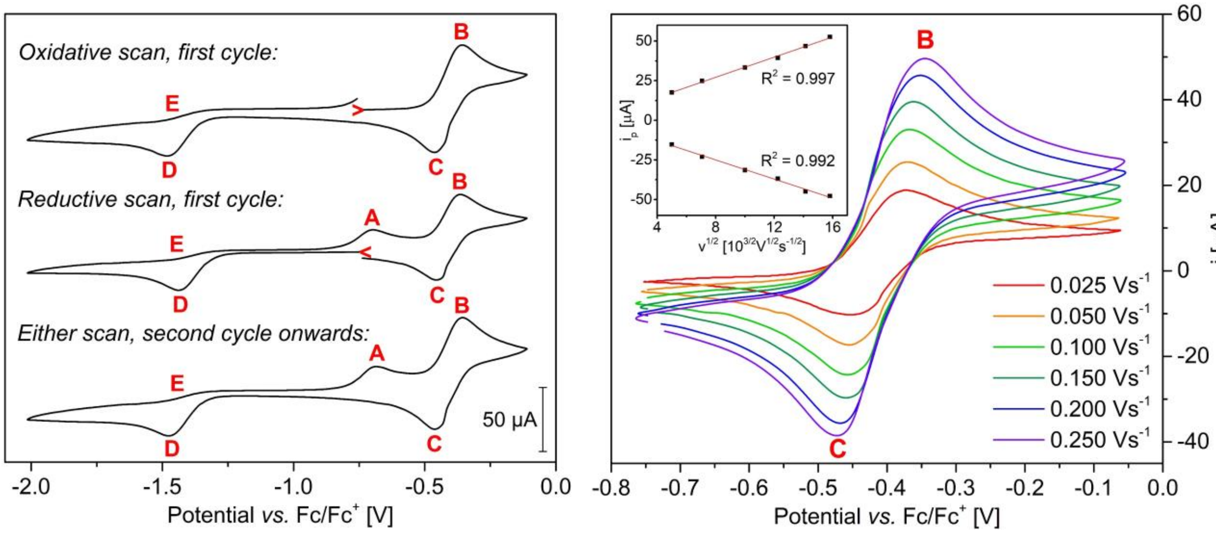

Figure S9. Cyclic voltammogram of compound **3a** (ca. 8 mM solution) in 0.1 M $^{n}Bu_4NPF_6$ acetonitrile electrolyte. Potentials are referenced with respect to Fc^+/Fc redox couple. Left panel: overview scans at the rate of 100 mV/s; the full scan starts from an open-circuit potential of -0.75 V). Right panel: a quasi-reversible redox pair **B**-**C** centered at $E_{1/2}$=-0.41 V; an insert shows linear dependence of the peak current vs. square root of the scan rate.

At the same time, the events associated with the $[(TSMP)_2Fe^{II}]^{2-}$ (**2**) species, coded as **A’/A’’** and **D’/D’’** in *Figure S8*, occur at slightly different potentials for $[(TSMP)_2Fe^{III}]K$ (**3a**) and for $[(TSMP)_2Fe^{II}][(B15C5)_2K]_2$ (**2b**). Moreover, the CV of **3a** exhibits an additional feature **E**, which can only be related to the oxidation of **2** as shown by the reductive CV scan of **3a** (*Figure S9*, *left panel*). Our previous studies and, in particular, variable-temperature ^{1}H NMR

spectroscopy,[5] indicate that **2** exists in more than one form in solution, which may be due to oligomerization and/or an equilibrium between pseodotetrahedral and octahedral coordination modes. The reorganization energy between these forms and the octahedral solution structure of $[(TSMP)_2Fe^{III}]^-$ (**3**) (see *Section S13.2.1*) is expected to be rather large, which is consistent with the electrochemical irreversibility of events **A**, **D** and **E**. We hypothesize that this reorganization energy as well as speciation of **2** in solution are modified by ion pairing, yielding the observed potential shifts and the new feature **E** in *Figure S8*.

S6 Solution magnetic moment and spin-crossover fitting

S6.1 Experimental details

Effective solution magnetic moments were obtained by the Evans method[6–8] using J. Young NMR tubes with a coaxial capillary insert as a reference. The latter was filled with a deuterated solvent with ~1% of TMS (or, alternatively, the same amount of a proteo-analogue of the deuterated solvent). The outer space contained a solution of the paramagnetic complex (5-10 mg/ml) in a deuterated solvent with the same concentration of TMS (alternatively, proteo-solvent). The absence of precipitation in the low-temperature measurements was controlled by comparing the effective magnetic moments measured at 25 °C before and after the cycle of cooling.

Molar magnetic susceptibility was calculated from the difference between the chemical shifts of TMS (or proteo-solvent) signals in the capillary and the outer solution (Δδ in Hz) using the following equation:

$$\chi_M = \frac{\Delta\delta M}{\nu_0 S_f c} - \chi_M^{dia}, \qquad \textit{Eq. S1}$$

where M – molecular weight of the studied compound (g/mol); ν_0 – frequency of the spectrometer (Hz); S_f – shape factor of the magnet (4π/3); c – concentration of the paramagnetic complex (mg/ml); χ_M^{dia} – molar diamagnetic contribution to the paramagnetic susceptibility calculated using Pascal's constants.[22] For variable-temperature measurements, the concentration c was adjusted so to take into account volumetric solvent expansion/contraction.

The effective magnetic moment was calculated as follows:

$$\mu_{eff} = \sqrt{8\chi_M T}, \qquad \textit{Eq. S2}$$

where T is temperature.

S6.2 Spin-crossover fitting of $[(TSMP)_2Fe^{III}]^-$ (3)

The complex $[(TSMP)_2Fe^{III}]^-$ (**3**) undergoes thermal spin-crossover (SCO) in solution. It is evident from strongly temperature-dependent effective solution magnetic moment (μ_{eff}) as measured by Evans method.[6–8] The results of such measurements are shown in *Figure S10* for

potassium salt [(TSMP)$_2$Fe^{III}]K (**3a**) in acetonitrile-d_3 and pyridine-d_5, and for tetraphenylphosphonium salt [(TSMP)$_2$Fe^{III}]PPh$_4$ (**3b**) in dichloromethane-d_2. Different NMR solvents were chosen to cover broader temperature range; salt **3b** was used for the experiments in dichloromethane-d_2 because otherwise **3a** is virtually insoluble in this solvent.

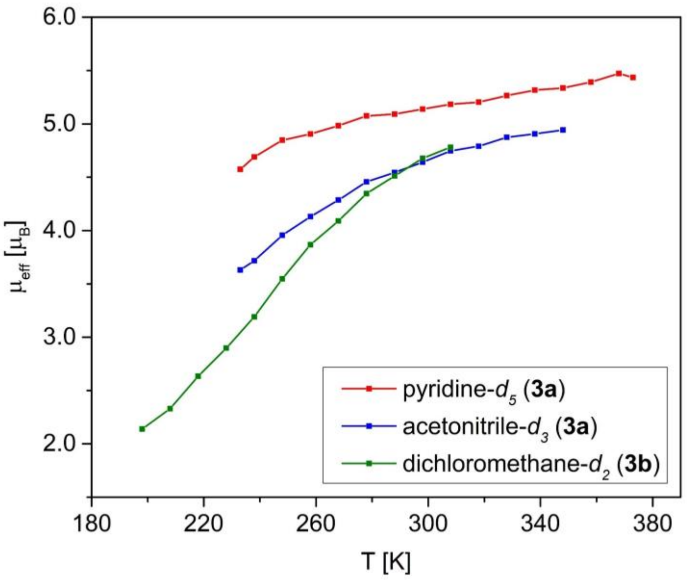

Figure S10. SCO curve for the [(TSMP)$_2$Fe^{III}]$^-$ (**3**) complex as obtained by Evans method in different solvents. Experimental datapoints are connected with straight segments for clarity. Explored temperature ranges are limited by the freezing and boiling points of the respective NMR solvents or precipitation of the compound at low temperature. Every curve is an average of four measurements.

Despite a relatively high error associated with the Evans method (5-10% depending on concentration),[9] the curvature of the plots as well as the absolute μ_{eff} values show that spin-crossover of [(TSMP)$_2$Fe^{III}]$^-$ (**3**) in solution is solvent- and, potentially, counterion-dependent. Thus, the difference between μ_{eff} in acetonitrile-d_3 and pyridine-d_5 is larger at 233 K than at 348 K. SCO of [(TSMP)$_2$Fe^{III}]K (**3a**) in acetonitrile-d_3 and pyridine-d_5 cannot be compared with that of [(TSMP)$_2$Fe^{III}]PPh$_4$ (**3b**) in dichloromethane-d_2 due to a different cation and possibility of ion-pairing in low-polar dichloromethane. Nevertheless, the crossover curve for **3b** is the most informative since it spans though both low- and high-μ_{eff} regions. It is possible to fit this transition and extract its thermodynamic parameters using regressive thermodynamic analysis.

The effective magnetic moment μ_{eff} of [(TSMP)$_2$Fe^{III}]PPh$_4$ (**3b**) can be expressed *via* magnetic moments of pure low- and high-spin states, μ_{LS} and μ_{HS} respectively, using their molar fractions:

$$\begin{array}{lcc} & Fe(III)^{LS} \leftrightarrow & Fe(III)^{HS} \\ molar\ fraction: & (1-x) & x \end{array}$$

$$\mu_{eff} = \mu_{LS} + (\mu_{HS} - \mu_{LS})x \qquad \textit{Eq. S3}$$

Molar fractions and μ_{eff}, in turn, can be expressed *via* the crossover equilibrium constant K:

$$K = \frac{x}{1-x} \qquad \textit{Eq. S4}$$

$$\mu_{eff} = \mu_{LS} + (\mu_{HS} - \mu_{LS})\frac{K}{1+K} \qquad \textit{Eq. S5}$$

And equilibrium constant K can be related to thermodynamics of the SCO as shown below:

$$-RTlnK = \Delta H - T\Delta S \qquad \textit{Eq. S6}$$

$$K = exp\left(\frac{\Delta S}{R} - \frac{\Delta H}{RT}\right) \qquad \textit{Eq. S7}$$

Rearranging the above equations gives the following regression model:

$$\mu_{eff} = \mu_{LS} + \frac{\mu_{HS} - \mu_{LS}}{1 + exp\left(\frac{\Delta H}{RT} - \frac{\Delta S}{R}\right)} \qquad \textit{Eq. S8}$$

Due to having twelve data points, in addition to crossover enthalpy and entropy, one can also fit the limiting magnetic moments μ_{LS} and μ_{HS}. The fitted curve is shown in a *Figure S11*.

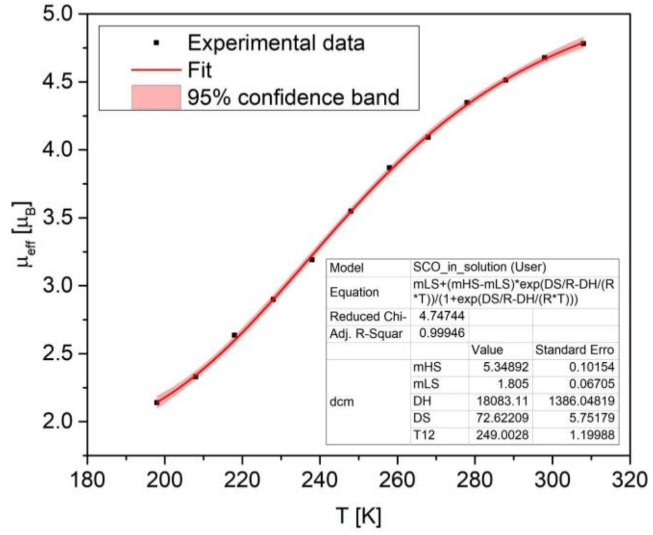

Figure S11. Fitted SCO of **3b** in dichloromethane-d_2 using regression model in *Eq. S8*.

Derived ΔH and ΔS are 18.1±1.4 kJ/mol and 72.6±5.8 J/(mol·K), respectively. This gives the critical temperature $\Delta H/\Delta S = T_c$ of 249±1 K. The limiting magnetic moments μ_{LS} and μ_{HS} of 1.81±0.07 and 5.35±0.10 μ_B, respectively, are close to the spin-only expectation values for the low-spin S=$^1/_2$ (1.73 μ_B) and high-spin S=$^5/_2$ (5.92 μ_B) states.

A similar fit can be performed on the data for $[(TSMP)_2Fe^{III}]K$ (**3a**) in acetonitrile-d_3, although with higher uncertainty (*Figure S12*). Thus, while the limiting μ_{HS} value is close to the that obtained for $[(TSMP)_2Fe^{III}]PPh_4$ (**3b**) in dichloromethane-d_2 (5.23 ± 0.08 *vs.* 5.35±0.10 μ_B), there are rather big errors associated with the lower value μ_{LS} (2.35±0.45 *vs.* 1.81±0.07 μ_B). This is mainly due to the fact that the SCO in acetonitrile-d_3 does not cover the low μ_{eff} range, which leads to high uncertainty in that part of the curve if predicted from the experimental

values. Hence, the ΔH of 14.4±2.4 kJ/mol, ΔS of 59.6±8.0 J/(mol·K) and T_c of 240±9 K, which are similar to those fitted for **3b** in dichloromethane-d_2 but with a higher standard error.

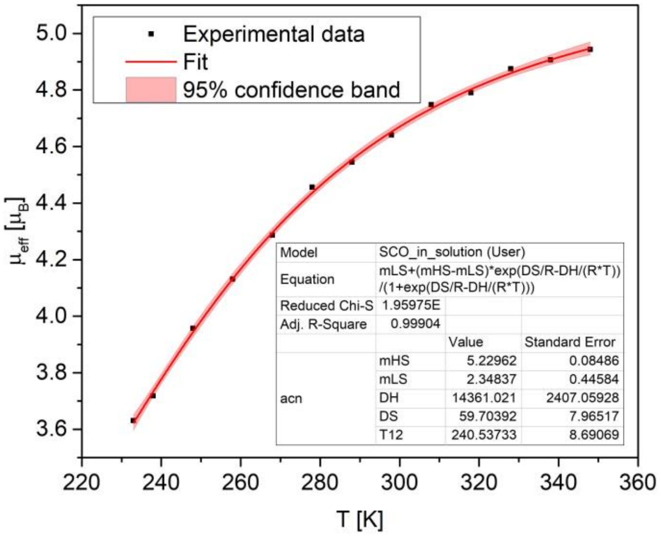

Figure S12. Fitted SCO of **3a** in acetonitrile-d_3 using regression model in *Eq. S8*.

Attempted fit for the SCO of $[(TSMP)_2Fe^{III}]K$ (**3a**) in pyridine-d_5 leads to an unusable curve with large uncertainties for the crossover parameters. It is not surprising, considering that the system is very close to the top plateau of the SCO sigmoid with only 3-4 data point in the low-temperature region containing information about the steep part of the curve.

S7. XAS spectroscopy

S7.1 Experimental details

The experimental spectra were measured with electron yield (EY) detection at 300 K on the 10-1 beamline at SSRL, Stanford.[23] . Samples were mounted to an aluminum sample holder using conductive carbon tape, placed in the measurement chamber, and pumped to 1e-8 Torr vacuum. The iron edge for each sample was scanned using monochromatic X-rays from a 1200 line/mm spherical grating monochromator, with a fixed exit slit of 25 microns, yielding a resolution of 0.2 eV. The total electron yield signal was collected by measuring the drain current from the sample holder, which was held at 37 degrees with respect to the incident beam. To avoid X-ray damage, a large beam of 1 x 1 mm was used, and the sample spot was changed every two scans. No change in the signal was observed from scan to scan.

S7.2 Computational details

The ligand field multiplet calculations have been performed with the CTM4XAS software.[24] The Fe(III) calculation used a $3d^5$ ground state with atomic parameters for the electron-electron interactions and the 2p and 3d spin-orbit coupling. The ligand field parameter 10Dq was set equal to 3.0 eV, which makes the high-spin $S = {}^5/_2$ and low-spin $S = {}^1/_2$ states almost

degenerate. The spectra of the $S = {}^{5}/_{2}$ and $S = {}^{1}/_{2}$ states have been calculated and added as described in the text. The Fe(IV) spectrum has been calculated using DFT-derived ordering of the 3d orbitals, which relates to a 10Dq value of 4.0 eV and a Ds value of 1.0 eV.

S8. SQUID magnetometry studies

S8.1 Experimental details

Magnetic susceptibility data were measured from powder samples of solid material in the temperature range of 2 to 300 K by using a SQUID susceptometer with a field of 0.1 T (MPMS-7, Quantum Design, calibrated with standard palladium reference sample, error <2%). Multiple-field variable-temperature magnetization (VTVH) measurements were done at 1, 4 and 7 T also in the range of 2 to 300 K with the magnetization equidistantly sampled on a 1/T temperature scale. The experimental data were corrected for underlying diamagnetism by use of tabulated Pascal's constants. An additional correction was done for temperature-independent paramagnetism. The susceptibility and magnetization data were simulated with the package julX written by E. Bill (Max-Planck Institute for Chemical Energy Conversion, Mülheim, Germany).

S8.2 Solid state SQUID magnetometry of [(TSMP)$_2$Fe^{III}][(B15C5)$_2$K] (3c)

Interestingly, the bis-benzo-15-crown-5 adduct [(TSMP)$_2$Fe^{III}][(B15C5)$_2$K] (**3c**) displays a μ_{eff} of 3.61 μ_B, in between the low- and high-spin states (similar to 3.88 μ_B for the case of an intermediate-spin ($S = {}^{3}/_{2}$)) and starts crossing over towards the high-spin (S = ${}^{5}/_{2}$) state upon heating to ca. 140 K. As discussed in the main text, this is not due to an intermediate-spin state, but actually represents an average of the low-and high-spin components co-existing in the crystal, as shown by X-ray crystallographic studies (see *Section S3*).

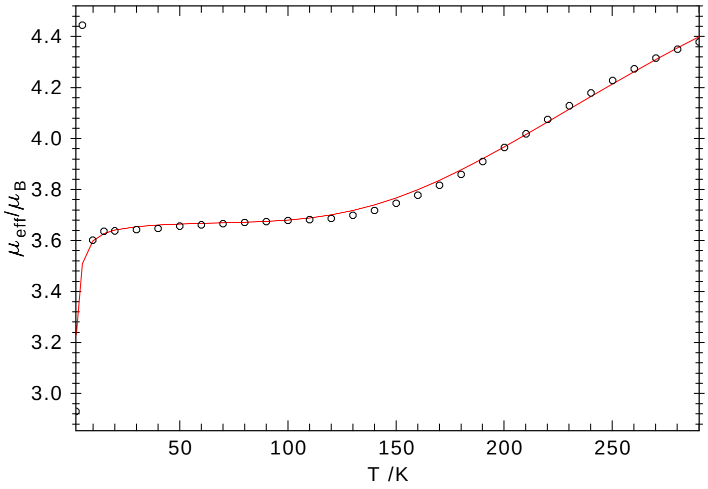

Figure S13. VT SQUID μ_{eff} measurement of microcrystalline solid sample of **3c**. The simulation with the domain model gave T_c = 500 K and nΔH = 10.9 kJ/mol.

S9. THz-EPR studies

S9.1 Experimental details

Frequency-Domain Fourier-Transform THz-EPR, or THz-EPR in short, was performed at the THz beamline at BESSY II, Helmholtz-Zentrum Berlin. The experiment is described in detail elsewhere.[25–27] Coherent synchrotron radiation provided through the low-α operation mode was used. Experiments were performed in Voigt geometry (radiation propagation direction $\boldsymbol{k}$ perpendicular to the external magnetic field B_0) and perpendicular mode (B_1 component of radiation perpendicular to the external magnetic field B_0). As detector, a liquid He-cooled Si bolometer (Infrared Laboratories) was used. The FTIR settings were: scanner velocity of 20 kHz, resolution of 1 cm^{-1}, and phase resolution of 2. Spectra were measured on a pressed pellet, prepared by homogenizing in a mortar 79 mg polycrystalline **4** and 101 mg polyethylene powder. All preparations were done under N_2 atmosphere. The spectra were measured at magnetic fields B_0 varying from 7.5 to 0 T and at a temperature of 4.8 K. A reference spectrum was measured at 31 K and 0 T. All spectra, including the reference, were averaged over 96 scans. Unless stated otherwise, the spectra in the discussion are shown in (relative) absorbance as $A(B_0) = \log_{10}\frac{I_{ref}}{I(B_0)}$, with I_{ref} and $I(B_0)$ corresponding to the reference transmittance spectrum and a transmittance spectrum measured at magnetic field B_0, respectively. Raw transmittance spectra are shown in *Figure S14*. Simulations were performed with EasySpin[28–30] using the spin Hamiltonian in *Eq. S9*.

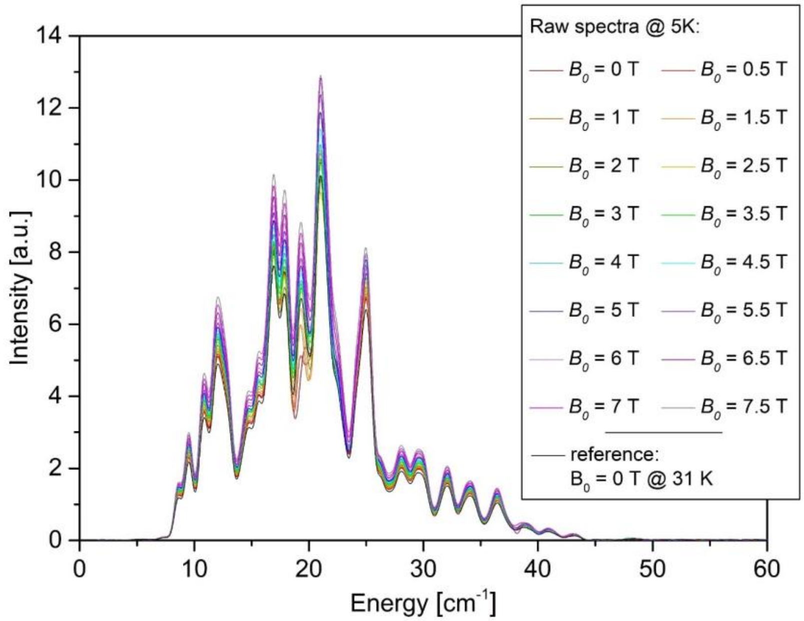

Figure S14. Transmittance THz-EPR spectra of **4**.

S9.2 General energy diagram

Assuming that nothing is known about **4** aside from the fact that it is an $S = 1$ system with a positive D-value, as shown by SQUID magnetometry (see the main text), its spin Hamiltonian can be written as follows:

$$\hat{H} = D\left(\hat{S}_z^2 - \tfrac{1}{3}\hat{\boldsymbol{S}}^2\right) + E\left(\hat{S}_x^2 - \hat{S}_y^2\right) + \mu_B \boldsymbol{B}_0 \boldsymbol{g} \hat{\boldsymbol{S}}, \qquad \textit{Eq. S9}$$

where the first and second terms describe the axial and rhombic zero-field splitting (ZFS) with $\hat{\boldsymbol{S}} = \left(\hat{S}_x, \hat{S}_y, \hat{S}_z\right)^T$ being the vector spin operator. The third term describes the interaction of the spin with the external magnetic field $\boldsymbol{B}_0$, where μ_B is the Bohr magneton and $\boldsymbol{g}$ is the *g*-tensor.

The corresponding energy eigenvalues can be found by diagonalization of this Hamiltonian. Assuming for a general case the rhombic parameter $E \neq 0$, the field-dependent energy diagram depicted in *Figure S15* is obtained.

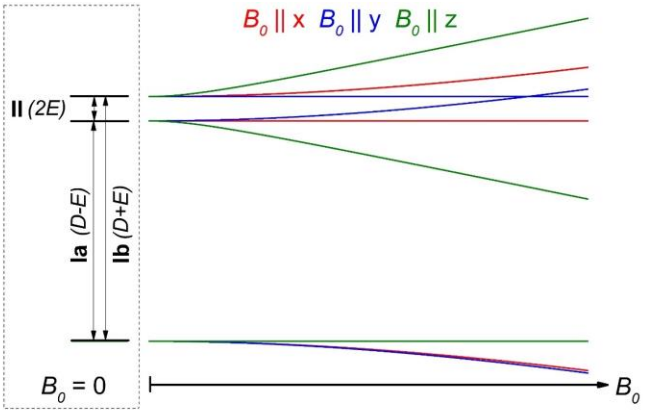

Figure S15. Energy splitting in an $S = 1$ system according to the spin Hamiltonian in *Eq. S9*. The left-hand side shows energy levels in the absence of an external magnetic field ($B_0 = 0$). On the right-hand side are shown the energies for different external field orientations assuming the *g*-tensor is isotropic and $D \gg \mu_B B_0$.

A positive D-value leads to a ground state singlet, while non-zero rhombicity ($E \neq 0$) lifts the degeneracy of the excited state, yielding two sublevels with an energy gap of $2E$ (transition **II**). Respectively, transitions from the ground to the excited state, **Ia** and **Ib**, have energies of $D - E$ and $D + E$. Due to the presence of ZFS, the direction of an external magnetic field (B_0) modulates the splitting of the energy levels. More specifically, for a field applied along the major anisotropy axis (*z*-axis), the splitting of the excited state changes linearly with the external magnetic field, while the energy of the ground-state remains constant. Contrarily, for directions perpendicular to the *z*-axis, the energy of the ground state decreases at higher fields, and as for the excited state, the energy of one sublevel increases, while that of the other remains constant.

S9.3 Alternative interpretations

It is worth to discuss the alternative interpretations of the experimental data. Firstly, since the THz-EPR experiments are incapable of distinguishing the sign of D, simulations with a negative value (*Figure S16*), while differing slightly, still give acceptable fits. However, a negative D would contradict the SQUID magnetometry results (see main text), therefore this possibility can be dismissed. Secondly, notwithstanding the estimated E of ≤ 0.3 cm^{-1}, it may be that E is actually much larger so that some transitions lie outside the experimentally probed spectral range. However, such simulations reveal that one would observe a field dependence that is unlike the experiment (*Figure S16*). Hence, this scenario can also be excluded.

While the relative absorbance spectra do not allow to precisely determine the transverse ($\perp z$) components of the g-tensor, its axial component ($\parallel z$) is clearly constrained as 1.97, since it defines the maximum and minimum transition energies and thus the total width of the signals.

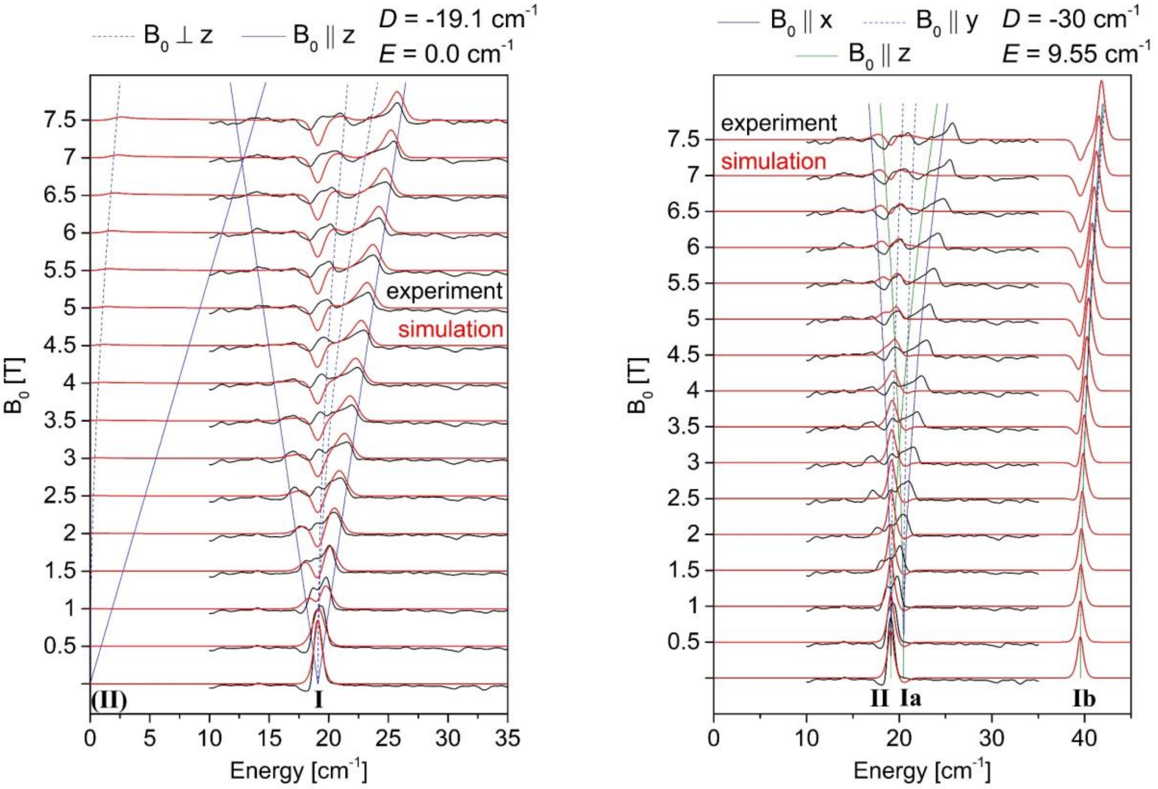

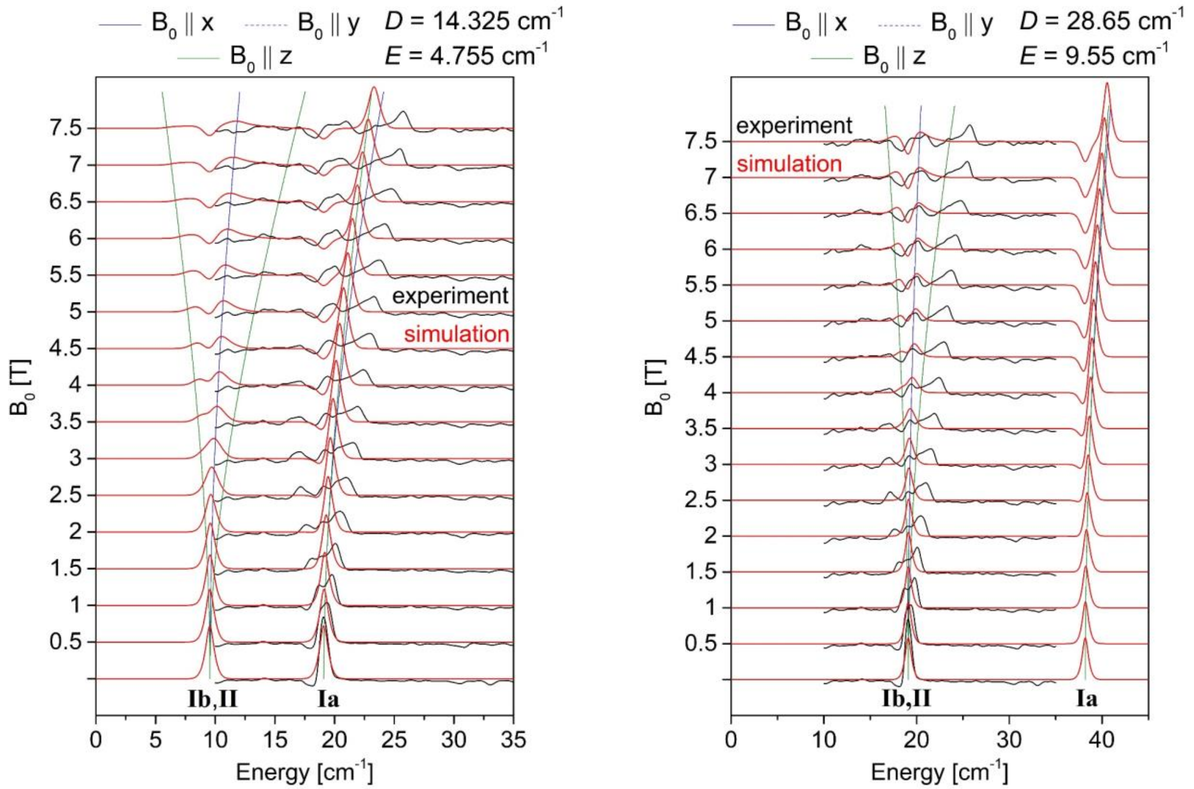

Figure S16. Dismissed alternative interpretations of THz-EPR spectra of **4**. Relative absorbance spectra (black lines) are offset for the magnetic field B_0 at which they were measured. Simulations for the spin Hamiltonian in *Eq. S9* are shown in red. The spectra were simulated with an axial g-value of 1.97 and the ZFS parameters indicated separately at the top right corner of each set of spectra. Calculated transition energies for magnetic fields applied parallel to the x, y, and z-axes are shown as dashed and solid blue and green lines, respectively. For the two bottom spectra, $E = D/3$ leads to invariance with respect to the sign of D. Furthermore, for these spectra, transitions **Ib** and **II** have the same energy. Their field dependence is, however, not entirely identical, which is not shown in the respective spectra. More specifically, the field dependences of **Ib** and **II** are identical for $B_0 \parallel x$. The field dependence of **Ib** for $B_0 \parallel y$ is identical to the field dependence of **II** for $B_0 \parallel z$ and vice versa.

S9.4 The negative relative absorbance feature at ~18.4 cm^{-1}

Interestingly, the experimental zero-field spectrum has a feature with negative relative absorbance around 18.4 cm^{-1}, directly below the ZFS energy, indicative of a higher absorption in the reference transmission spectrum measured at 31 K as opposed to the transmittance measured at 4.8 K (*Figure S17*). A similar situation was observed for another S = 1 molecule.[31] This feature does not react to increasing B_0, and also manifests as a lowering of the relative absorbance at higher B_0, which allows to assign it to a high-temperature effect in the zero-field reference spectrum. All potential reasons for this phenomenon are discussed elsewhere[31] and go beyond the used spin Hamiltonian in *Eq. S9*. This explains why this feature is not reflected in our simulations, although the simulations at higher magnetic fields do show some negative absorbance, albeit centered at 19.1 cm^{-1}. A closer examination of the zero-field reference

transmittance at 31 K and the spectrum at 4.8 K (*Figure S17*) shows that the former is more intense at around 19.1 cm^{-1} (transition **I**), which is the cause of the positive absorbance in *Figure 10* (see main text). As the 4.8 K signals broaden with increasing B_0, while the same zero-field 31 K absorbance is used as reference throughout, the central signal intensity becomes lower at 4.8 K than in the reference (*Figure S18*), resulting in the calculated negative relative absorbance.

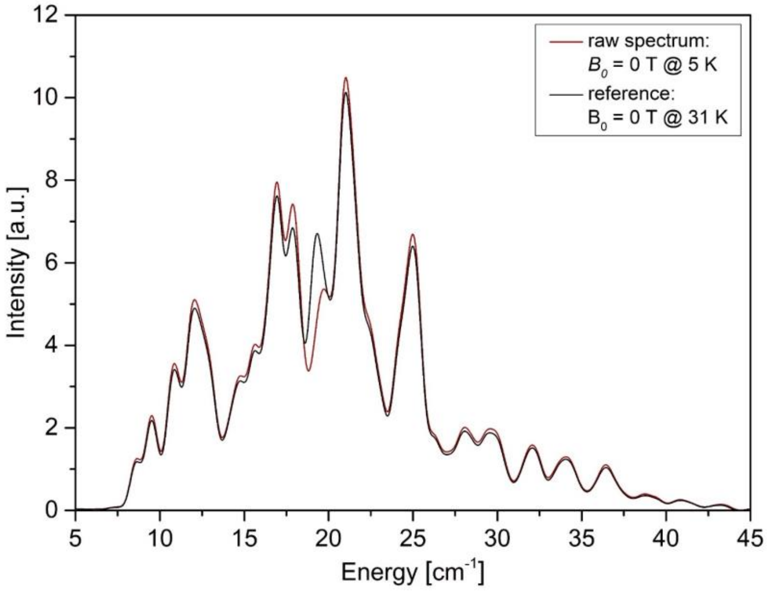

Figure S17. Comparison of the zero-field transmittance spectrum of **4** at 4.8 K and the reference spectrum at 31 K.

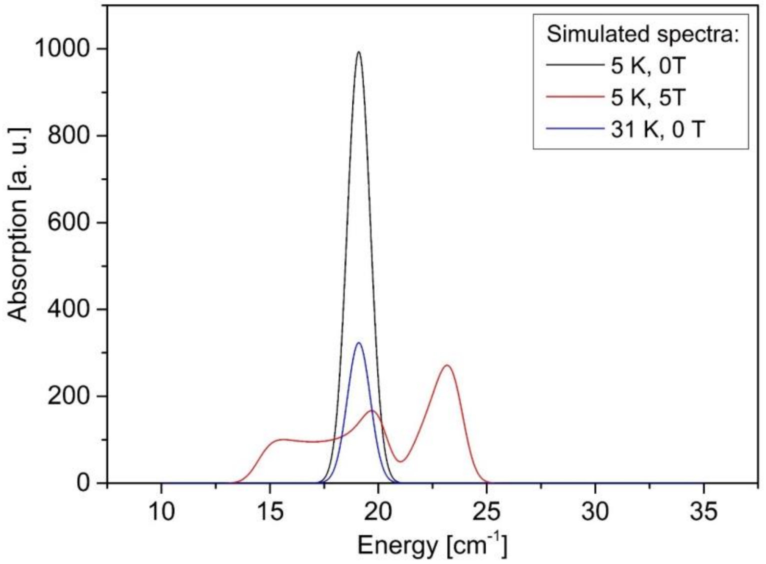

Figure S18. Simulated absorption spectra for **4** using the spin Hamiltonian in *Eq. S9* with D = 19.1 cm^{-1}, E = 0 cm^{-1} and an isotropic g-value of 1.97.

S10. ^{57}Fe Mössbauer spectroscopy

S10.1 Experimental details

Mössbauer spectra were recorded on conventional spectrometers with alternating constant acceleration of the g-source. The minimum experimental line width was 0.24 mm/s (full width at half-height). The sample temperature was maintained constant using an Oxford Instruments Variox cryostat. The detector was an Ar/10%CH_4-filled end-window type proportional counter. Isomer shifts are quoted relative to iron metal at 300 K. The spectra were simulated as Lorentzian doublets with the program *mf.SL* written by E. Bill (Max-Planck Institute for Chemical Energy Conversion, Mülheim, Germany).

S10.2 Detailed breakdown of the quadrupole splitting

The quadrupole splitting is related to the electric field gradient (EFG) tensor, which is a symmetric 3×3 matrix. This tensor possesses a set of proper axes in which it is diagonal. Additionally, it is traceless, meaning that only two parameters are sufficient to describe it: the axial component V_{zz} and the asymmetry parameter η, which can be expressed as follows:

$$\eta = \frac{V_{xx} - V_{yy}}{V_{zz}}, \qquad \textit{Eq. S10}$$

where $|V_{zz}| \geq |V_{xx}| \geq |V_{yy}|$ by convention.

The quadrupole splitting ΔE_Q is related to these parameters *via* the following expression:

$$\Delta E_Q = \frac{eQ\,V_{zz}}{2}\sqrt{1 + \frac{\eta^2}{3}}, \qquad \textit{Eq. S11}$$

where eQ is the quadrupolar moment of a ^{57}Fe nucleus.

Due to the high symmetry of the molecule of **4** (C_3-axis), the asymmetry parameter is very close to zero (η = 0.05). Hence, V_{zz} is the only parameter determining the quadrupolar splitting.

It is possible to partition V_{zz} into a set of atomic orbital contributions:

$$V_{zz} = \sum_{\mu}\sum_{\nu} P_{\mu\nu}\langle\phi_\mu|V_{zz}|\phi_\nu\rangle, \qquad \textit{Eq. S12}$$

where $P_{\mu\nu} = \sum_i c_{\mu i}{}^* c_{\nu i}$, the $c_{\mu i}$ are the coefficients of the atomic orbital $|\phi_\mu\rangle$ in the molecular orbital i.

Differentiation of the atomic orbitals on iron and those on the ligand gives:

$$V_{zz} = \sum_{\mu}^{Fe}\sum_{\nu}^{Fe} P_{\mu\nu}\langle\phi_\mu|V_{zz}|\phi_\nu\rangle + \sum_{\mu}^{Fe}\sum_{\nu}^{L}\left(P_{\mu\nu}\langle\phi_\nu|V_{zz}|\phi_\mu\rangle + P_{\nu\mu}\langle\phi_\nu|V_{zz}|\phi_\mu\rangle\right) \qquad \textit{Eq. S13}$$

$$+\sum_{\mu}^{L}\sum_{\nu}^{L}P_{\mu\nu}\langle\phi_\mu|V_{zz}|\phi_\nu\rangle+\sum_{\mu}^{L}\sum_{\nu}^{L'}P_{\mu\nu}\langle\phi_\mu|V_{zz}|\phi_\nu\rangle\,.$$

The first term corresponds to the one-center contribution, the second one corresponds to the two-center bond contribution, the third one corresponds to the two-center point charge contribution, and the fourth term relates to the three-center contribution.

The one-center contribution is normally a dominant term, and can be further expanded into a core orbital and molecular orbital (or valence) contributions:

$$\sum_{\mu}^{Fe}\sum_{\nu}^{Fe}P_{\mu\nu}\langle\phi_\mu|V_{zz}|\phi_\nu\rangle=$$
$$=\sum_{i}^{core}\sum_{\mu}^{Fe}\sum_{\nu}^{Fe}c_{\mu i}^{*}c_{\nu i}\langle\phi_\mu|V_{zz}|\phi_\nu\rangle+\sum_{i}^{valence}\sum_{\mu}^{Fe}\sum_{\nu}^{Fe}c_{\mu i}^{*}c_{\nu i}\langle\phi_\mu|V_{zz}|\phi_\nu\rangle=\qquad \textit{Eq. S14}$$
$$=V_{zz}^{1c-core}+V_{zz}^{1c-valence}.$$

This gives a total of five terms:

$$V_{zz}=V_{zz}^{1c-core}+V_{zz}^{1c-valence}+V_{zz}^{2c-bond}+V_{zz}^{2c-PC}+V_{zz}^{3c}\,,\qquad \textit{Eq. S15}$$

where $V_{zz}^{1c-core}$ – a one-center core orbital contribution; $V_{zz}^{1c-valence}$ – a one-center valence contribution; $V_{zz}^{2c-bond}$ – a two-center bond contribution; V_{zz}^{2c-PC} – a two-center point charge contribution; V_{zz}^{3c} – a three-center contribution.

The values of these contributions are given in *Table S1*. As emphasized in the main text, the valence contribution ($V_{zz}^{1c-valence}$) is dominant over all others. At the same time, the three-center contribution (V_{zz}^{3c}) is so weak that it can be neglected.

Table S1. Contributions to the axial component V_{zz} of the EFG tensor (expressed in a.u.$^{-3}$).

$V_{zz}^{1c-core}$	$V_{zz}^{1c-valence}$	$V_{zz}^{2c-bond}$	V_{zz}^{2c-PC}	**Total (V_{zz})**
+0.06	-0.92	-0.40	0.04	-1.22

The valence contribution can be further partitioned between non-bonding $1a_1$, π-antibonding $1e$, σ-bonding and π-bonding orbitals (*Table S2*). The analysis shows that the $1a_1$ contribution dominates over that of $1e$ orbitals. As specified in the main text, this is due to the mixing between the $(d_{xy}, d_{x^2-y^2})$ and (d_{xz}, d_{yz}) orbital pairs, which have opposite contributions to the $1e$-related part of $V_{zz}^{1c-valence}$. Furthermore, the $1e$ orbitals are significantly π-antibonding, which reduces their one-center contribution. However, the corresponding π-bonding orbitals have a significant 1e character. Therefore, they have a non-negligible positive valence contribution, especially considering that these orbitals are doubly-occupied.

Table S2. Orbital contributions to the valence axial component of $V_{zz}^{1c-valence}$ of the EFG tensor (expressed in a.u.$^{-3}$).

$1a_1$	$1e$	σ-bonding	π-bonding	Total ($V_{zz}^{1c-valence}$)
-5.00	+2.04	+0.17	+2.05	-0.92

S11. DFT calculations

S11.1 Calculated Mössbauer Parameters and Electronic Structure of $[(TSMP)_2Fe^{III}]K$ (3a)

The electronic structure of the ferric species within the crystal structure of **3a** was computed. Its unit cell contains two independent molecular anions; molecule **A** has four shorter Fe–N bond lengths of ca. 1.988 Å and two longer Fe–N bond length of ca. 2.002 Å, and molecule **B** has four longer Fe–N bond lengths of ca. 1.988 Å and two shorter Fe–N bond length of ca. 1.955 Å. The coordination geometry of both isomers is best described as an elongated or a compressed octahedron. Both isomers are nearly isoenergetic with a marginal energy difference of 0.6 kcal/mol. Despite these similarities, **A** and **B** have distinct electron configurations, namely, $(d_{xy})^1(d_{xz})^2(d_{yz})^2$ for **A** and $(d_{xy})^2(d_{xz})^2(d_{yz})^1$ for **B** (*Figure S19*). As such, the ground state of the system possesses orbital near-degeneracy and hence sizeable unquenched orbital angular momentum, consistent with the measured effective magnetic moment being larger than the spin-only value. The computed Mössbauer parameters (*Table S3*) are both in reasonable agreement with the experiment.

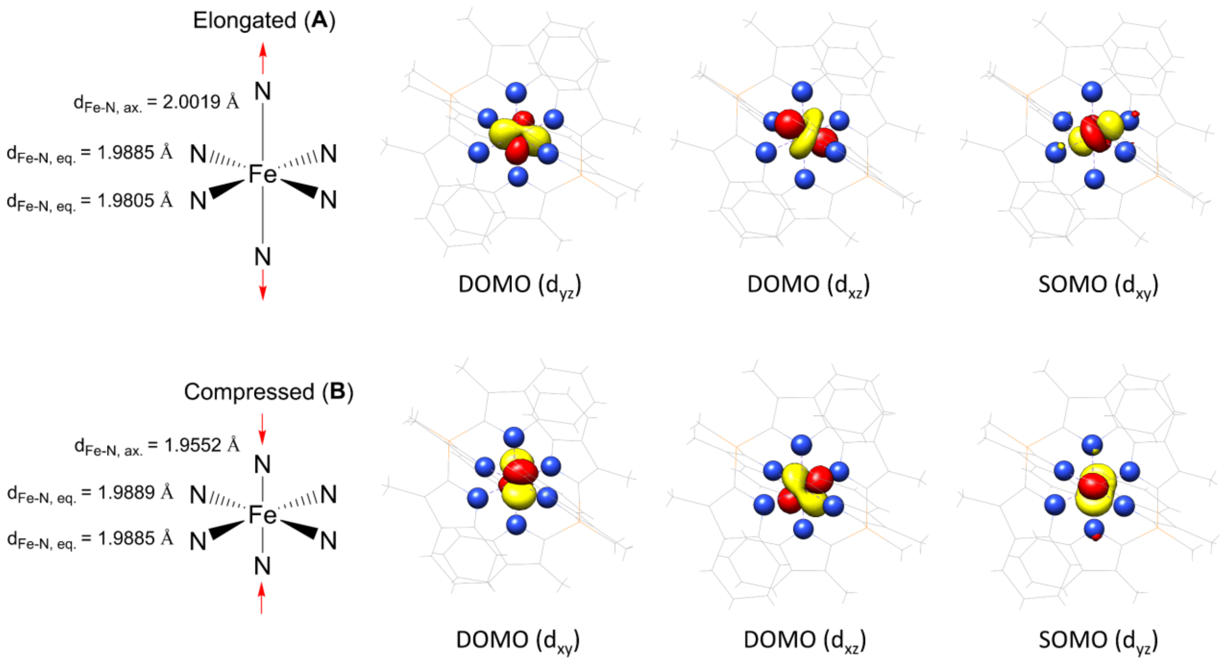

Figure S19. Localized filled and half-filled molecular orbitals of anions **A** and **B** calculated on X-ray crystallographic geometries at B3LYP-D3BJ/def2-TZVP level of theory with the CP(PPP) basis set for Fe. The orbitals were labeled after the dominant contributor.

Table S3. Relative SCF energies and ^{57}Fe Mössbauer spectral parameters calculated at the B3LYP-D3BJ/def2-TZVP level of theory with the CP(PPP) basis set for Fe for the two independent molecules of **3a**, compared to the experimental parameters.

Molecule	Relative energy, kcal/mol	δ, mm/s	ΔE_Q, mm/s
**A**	0.6	0.29	2.19
**B**	0.0	0.26	2.18
Exp.	-	0.25	1.63

S11.2 Calculated Mössbauer parameters of $[(TSMP)_2Fe^{IV}]^0$ (4)

Table S4. Relative SCF energies and ^{57}Fe Mössbauer spectral parameters calculated for alternative spin states of **4** (S = 0, 1, 2), compared to the experimental parameters. The structures were optimized at the BP86-D3BJ/def2-TZVP level of theory. Energies and Mössbauer spectral parameters were calculated using the B3LYP-D3BJ/def2-TZVP level of theory with the CP(PPP) basis set for Fe.

Spin state	Relative energy, kcal/mol	δ, mm/s	ΔE_Q, mm/s
$S = 0$	17.8	0.04	+2.75
$S = 1$	0	–0.05	–2.01
$S = 2$	25.0	0.10	+1.49
Exp.	-	0.04	±1.96

S11.3 Spin density in $[(TSMP)_2Fe^{IV}]^0$ (4)

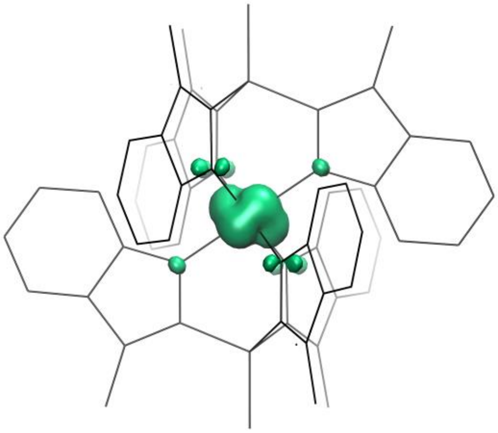

Figure S20. Spin density distribution in **4** (isocontour=0.01). Calculations were performed at B3LYP-D3BJ/def2-TZVP (CP(PPP) for Fe) level of theory using a geometry optimized at BP86-D3BJ/def2-TZVP level.

S11.4 Computational geometries of $[(TSMP)_2Fe^{IV}]^0$ (4) and its Si-tethered analogue

Table S5. Comparison of selected parameters from computationally optimized geometries of $[(TSMP)_2Fe^{IV}]$ (**4**) and its Si-tethered analogue **G**. Level of theory: BP86-D3BJ/def2-TZVP.

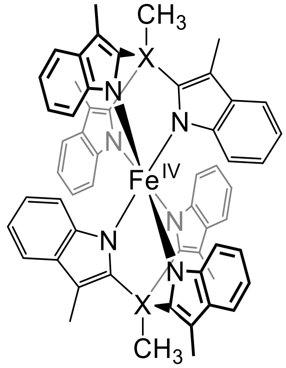

Geometric parameter	X=P⁺ (4)	X=Si (G)
Fe-N	1.95	1.97
N-C^{Ar}	1.39	1.40
C^{Ar}-X	1.76	1.85
X-Me	1.80	1.89
Fe···X	3.16	3.25
N^Fe^N	91.9	92.7
Fe-N-C^{Ar}	124.1	124.8
N-C^{Ar}-X	112.5	113.3
C^{Ar}^X^Me	106.0	114.7

S12. CASSCF calculations

S12.1 Calculation of the ZFS parameters of $[(TSMP)_2Fe^{IV}]^0$ (4)

The zero-field splitting arises from two physical effects, namely spin-spin coupling and spin-orbit coupling. The axial parameter of the zero-field splitting may be calculated by effective Hamiltonian theory. It is a sum of contributions arising from the ground state (spin-spin coupling) and each excited Born-Oppenheimer states (spin-orbit coupling). Only the states with a spin quantum number S identical to or different from that of the ground state by ± 1 have a non-zero contribution to the zero-field splitting.

A state contribution is proportional to $\frac{\zeta}{\Delta E}$, where ζ is the spin-orbit coupling constant and ΔE the excitation energy of the state. The threshold of 24 000 cm^{-1} (48 ζ) was chosen as the limit above which the states contributions can be neglected. The energies and individual contributions of each state are reported in *Table S6*.

Table S6. Individual contributions of the excited states to the axial parameter *D* of the zero-field splitting.

**Energy (cm^{-1})**	**Configuration**	**Multiplicity**	**Contribution to *D* (cm^{-1})**
0	98% $(1a_1)^2(1e_x)^1(1e_y)^1$	3	0
10 827	97% $(1a_1)^1(1e_x)^2(1e_y)^1$	3	+2.111
10 864	97% $(1a_1)^1(1e_x)^1(1e_y)^2$	3	+2.095
12 249	68 % $(1a_1)^2(1e_x)^1(1e_y)^1$<br>10% $(1a_1)^2(1e_x)^2$<br>10% $(1a_1)^2(1e_y)^2$	1	-0.253
12 257	34% $(1a_1)^2(1e_x)^2$<br>34% $(1a_1)^2(1e_y)^2$<br>21% $(1a_1)^2(1e_x)^1(1e_y)^1$	1	-0.253
19 054	$(1a_1)^1(1e_x)^1(1e_y)^1(2e_x)^1$	5	-0.810
19 083	$(1a_1)^1(1e_x)^1(1e_y)^1(2e_y)^1$	5	-0.810
20 478	31% $(1e_x)^2(1e_y)^2$<br>29% $(1a_1)^2(1e_x)^2$<br>29% $(1a_1)^2(1e_y)^2$	1	+10.267
24 142	89% $(1a_1)^1(1e_x)^2(1e_y)^1$	1	-0.864
24 170	89% $(1a_1)^1(1e_x)^1(1e_y)^2$	1	-0.860
37 787	43% $(1e_x)^2(1e_y)^2$<br>11% $(1a_1)^2(1e_x)^1(2e_y)^1$<br>11% $(1a_1)^2(1e_y)^1(2e_x)^1$<br>8% $(1e_x)^1(1e_y)^2(2e_y)^1$<br>8% $(1e_x)^2(1e_y)^1(2e_x)^1$<br>6% % $(1a_1)^2(1e_x)^2$<br>6% % $(1a_1)^2(1e_y)^2$	1	+3.763
43 878	20% $(1a_1)^1(1e_x)^1(1e_y)^1(2e_x)^1$<br>19% $(1a_1)^2(1e_y)^1(2e_x)^1$<br>18% $(1a_1)^2(1e_x)^1(2e_y)^1$<br>10% $(1a_1)^1(1e_x)^2(2e_y)^1$<br>10% $(1a_1)^1(1e_y)^2(2e_y)^1$<br>8% $(1a_1)^1(1e_x)^1(1e_y)^1(2e_y)^1$	1	+0.943
45 689	22% $(1a_1)^2(1e_x)^1(2e_x)^1$<br>21% $(1a_1)^2(1e_y)^1(2e_y)^1$<br>16% $(1a_1)^1(1e_x)^1(1e_y)^1(2e_y)^1$<br>8% $(1a_1)^1(1e_x)^2(2e_x)^1$<br>8% $(1a_1)^1(1e_y)^2(2e_x)^1$<br>6% $(1a_1)^1(1e_x)^1(1e_y)^1(2e_x)^1$	1	0

S13 Paramagnetic NMR studies

S13.1 Theoretical background

S13.1.1 On the origin of paramagnetic shifts

Despite the plethora of information that can be extracted from the NMR experiments on paramagnetic systems, their potential remains severely underused. There are several literature sources that cover paramagnetic NMR at a rather introductory[32,33] or a more fundamental level.[34–38] However, for the sake of further discussion, the origin of chemical shifts and linewidths in such systems will be briefly covered below.

Consider a system of coupled nuclear ($I = {}^1/_2$) and electronic ($S = {}^1/_2$) spins in an external magnetic field (*Figure S21*). Nuclear Zeeman splitting leads to two states with separation of ΔE_N. Every nuclear state undergoes subsequent electron Zeeman splitting of ΔE_e yielding four states with different combinations of projected spin angular momenta, m_I and m_S, on the external field. These states are further modified by $\pm{}^A/_4$, where A is a hyperfine coupling constant between the corresponding nucleus and the unpaired electron, yielding states characterized by energies E_1 through E_4, between which transitions take place.

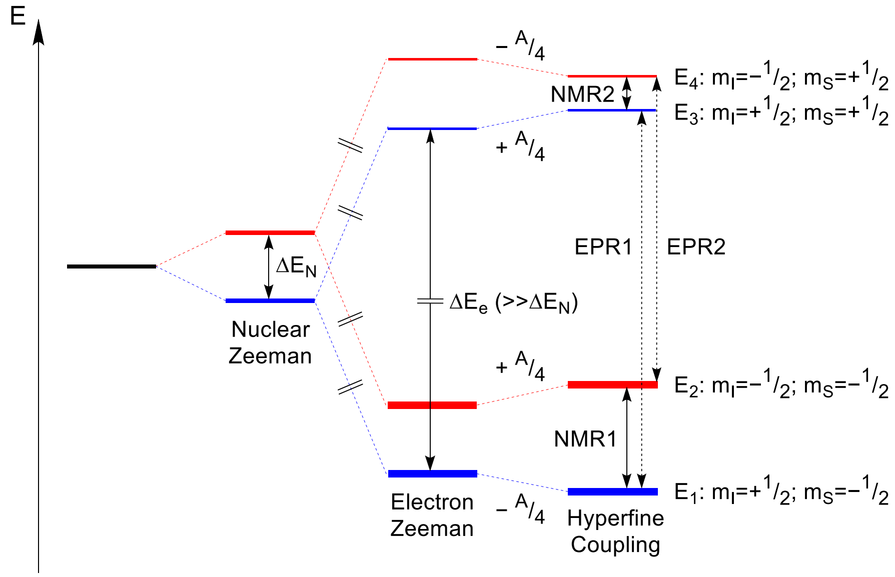

Figure S21. A system of nuclear and electronic spins that couple with a hyperfine constant A. The scale in the figure is distorted: electronic Zeeman splitting (ΔE_e) is four orders of magnitude larger than nuclear Zeeman splitting (ΔE_N). Relative line thickness denotes a difference in thermal population.

Depending on the quantum numbers that change, NMR ($\Delta m_I = \pm 1$) or EPR ($\Delta m_S = \pm 1$) transitions can occur. Yet, regardless of the kind of transition, they contain the same structural and electronic information that can often be extracted.[32]

An astute reader may have noticed that, despite two NMR transitions shown in *Figure S21*, one normally observes singlets in paramagnetic NMR spectra. This is due to the fact that nuclei relax on the timescale of seconds whereas the lifetime of electronic states is within 10^{-5} to 10^{-13} s.[33,38] Therefore, a nucleus experiences an average oscillating field of an electron rapidly

flipping between m_S=$-^1/_2$ and $+^1/_2$ states. Normally, the frequency of this field is many orders of magnitude higher than the nuclear Larmor frequency, which yields a singlet that is an average of the exchanging states. In principle, this should cancel the hyperfine coupling leading to a chemical shift indistinguishable from that in the absence of an unpaired electron (orbital or diamagnetic shift, δ^{dia}). However, due to the high gyromagnetic ratio of the electron, the Zeeman splitting (ΔE_e) is so large that thermal populations of m_S = $-^1/_2$ and $+^1/_2$ states are not equal, therefore complete cancellation does not occur, giving a singlet shifted away from δ^{dia} by a certain value called a hyperfine shift (δ^{HF}). Thus, in effect, a paramagnetic NMR signal is a weighted average of a hyperfine doublet where the weights are thermal populations of the m_S = $-^1/_2$ and $+^1/_2$ electronic states.

Summing up the above, the observed paramagnetic NMR chemical shift (δ^{obs}) can be broken down in two parts: an orbital or diamagnetic term (δ^{dia}) and a hyperfine term (δ^{HF}). The former is a would-be chemical shift should an unpaired electron not be present. It is defined by the nuclear Zeeman splitting energy (ΔE_N) and, to a first approximation, is temperature-independent due to a small magnitude of ΔE_N (radiofrequency range). The hyperfine term, δ^{HF}, originates from coupling between electronic and nuclear magnetic moments and is strongly temperature-dependent because of the different thermal populations of the m_S electronic states.

There are many possible contributors to the δ^{HF},[35] however Fermi contact (δ^{FC}) and pseudocontact (δ^{PC}, also named dipolar) shifts are the most important.

$$\delta^{obs} = \delta^{dia} + \delta^{HF} \cong \delta^{dia} + \delta^{FC} + \delta^{PC}. \qquad \textit{Eq. S16}$$

Fermi contact shift (δ^{FC}) originates from scalar electron-nuclear coupling through chemical bonds. More specifically, metal-centered unpaired electrons result into non-zero spin density on other atoms in the molecule either *via* direct delocalization though σ- and π-bonds or indirectly through spin polarization.[39] Spin density influences NMR chemical shifts if it resides in orbitals of s-character which have non-zero electron density on a nucleus. In a way, this mechanism is analogous to scalar or *J*-coupling in solution NMR experiments on diamagnetic systems. Both effects also quickly vanish with the number of bonds separating coupled spins unless π-conjugated systems are involved. Fermi-contact shift can be expressed *via* isotropic hyperfine coupling constant A_{iso} as shown below:

$$\delta^{FC} = \frac{S(S+1)\mu_B}{3kTg_N\mu_N} g_{iso} \cdot A_{iso}\,, \qquad \textit{Eq. S17}$$

where S – electronic spin quantum number of the complex; μ_B – Bohr magneton; g_N – nuclear g-value; μ_N – nuclear magneton; k – Boltzmann constant; T – temperature; g_{iso} – isotropic electronic g-value of the system; A_{iso} – isotropic hyperfine coupling constant.

Pseudocontact shift (δ^{PC}) is a result of non-mediated through-space coupling between nuclear and electronic magnetic moments. It is analogous to dipolar coupling in the solid-state NMR experiments. In general, δ^{PC} are more difficult to describe mathematically since, due to spin delocalization, there may be several sources of the magnetic moment. In the simplest case,

assuming a metal center to be a point magnetic dipole and the only source of the magnetic moment (the so-called point-dipole approximation), δ^{PC} can be expressed as follows:

$$\delta^{PC} = \frac{1}{12\pi r^3}\left[\Delta\chi_{ax}(3cos^2\theta - 1) + \frac{3}{2}\Delta\chi_{rh}sin^2\theta cos2\varphi\right], \qquad \textit{Eq. S18}$$

where r, θ and φ – spherical coordinates of a nucleus within the intrinsic frame of magnetic susceptibility tensor; $\Delta\chi_{\mathrm{ax}}$ and $\Delta\chi_{\mathrm{rh}}$ – temperature-dependent axial anisotropy and rhombicity of the above tensor. The latter two are defined as:

$$\Delta\chi_{\mathrm{ax}} = \tilde{\chi}_{zz} - \frac{\tilde{\chi}_{xx} - \tilde{\chi}_{yy}}{2}, \qquad \textit{Eq. S19}$$

$$\Delta\chi_{\mathrm{rh}} = \tilde{\chi}_{xx} - \tilde{\chi}_{yy}, \qquad \textit{Eq. S20}$$

where $\tilde{\chi}_{xx}$, $\tilde{\chi}_{yy}$ and $\tilde{\chi}_{zz}$ are components of a diagonalized magnetic susceptibility tensor. The origin of magnetic susceptibility tensor is at the position of the paramagnetic center.

For system with more than one unpaired electron, $\Delta\chi_{\mathrm{ax}}$ and $\Delta\chi_{\mathrm{rh}}$ can be further expressed *via* other spectroscopic observables as:[40–42]

$$\Delta\chi_{\mathrm{ax}} = -W \cdot \left[g_{zz}^2 D_z - \frac{g_{xx}^2 D_x + g_{yy}^2 D_y}{2}\right], \qquad \textit{Eq. S21}$$

$$\Delta\chi_{\mathrm{rh}} = -W \cdot \left[g_{xx}^2 D_x - g_{yy}^2 D_y\right], \qquad \textit{Eq. S22}$$

with

$$W = \frac{\mu_0 \mu_B^2 S(S+1)(2S-1)(2S+3)}{30(k_B T)^2}, \qquad \textit{Eq. S23}$$

where g_{xx}, g_{yy}, g_{zz}, D_x, D_y, D_z are diagonal components of g- and ZFS-tensors, respectively; μ_0 – vacuum permeability; μ_B – Bohr magneton; S – spin quantum number of the system; k_B – Boltzmann constant; T – temperature. In turn, the diagonal components of the ZFS-tensor are connected with (measurable) ZFS parameters D and E as follows:

$$D = \frac{3}{2}D_z, \qquad \textit{Eq. S24}$$

$$E = \frac{1}{2}(D_x - D_y). \qquad \textit{Eq. S25}$$

This, however, does not mean that systems with a single unpaired electron have no pseudocontact shifts due to having no ZFS. On the contrary, such systems may have sizeable δ^{PC} that can be taken into account by departing from the ZFS formalism and directly considering the spin-orbit coupling.

As can be seen from the *Eq. S18*, pseudocontact shifts are strongly orientation-dependent and inversely scale with cubed distance r. Nevertheless, while contact shifts tend to vanish after several bonds, pseudocontact shifts can persist up to 50 Å. In most cases, the point-dipole approximation gives reasonable results, especially if M···H distances are >5Å, but sometimes more sophisticated models must be used.[43,44]

By using observed and diamagnetic chemical shifts (δ^{obs} and δ^{dia}, respectively) and by knowing the equations for hyperfine shift (δ^{HF}), it is possible to extract structural and electronic information about a paramagnetic species.[33] Several approximations are normally employed. First of all, diamagnetic shifts (δ^{dia}) are taken from an isostructural closed-shell analogue, for instance δ^{dia} for an Fe(III) metal complex can be taken from a Ga(III) one or even from a free ligand. As another approximation, very often only either a contact (δ^{FC}) or a pseudocontact (δ^{PC}) term is considered. The former is thought to dominate in organic radicals and covalent d-block compounds[45–48] with the latter being prominent for f-block compounds.[36] A good diagnostic criterion for a strong Fermi-contact contribution is a linear dependence of hyperfine shift on inverse temperature (T^{-1}), which follows from *Eq. S17*. In other words, a Fermi contact-dominated hyperfine shift can be regarded as local paramagnetic susceptibility and should follow Curie's law: $\delta^{HF}T = const$. Should the pseudocontact shift contribution be significant, T^{-1} behavior is complemented by the T^{-2} dependence[32] with the weight of the latter being related to the zero-field splitting (ZFS) or spin-orbit coupling in the system (*Eq. S18–Eq. S23*).[49]

Discussed sources of hyperfine shift considered in time can lead to fast nuclear relaxation. Combined with Curie relaxation, which is analogous to chemical shift anisotropy relaxation in solution NMR on diamagnetic systems, they define the linewidth. To generalize, for any kind of nuclear relaxation to happen, a nucleus must experience a magnetic field oscillating at or close to its own Larmor frequency. In solution, it can be provided by electron Zeeman transitions, mutual rotation and tumbling of individual magnetic moments. Therefore, mechanics of nuclear relaxation is nuanced with strong dependence on spin density, temperature, molecular mass, viscosity of the solution and availability of several relaxation channels that may act simultaneously. It is often the case, however, that dipolar relaxation dominates for small molecules. If so, for molecular motion that is much faster that nuclear Larmor frequency (the so-called fast motion limit), longitudinal (T_{1M}) and transverse (T_{2M}) relaxation constants become equal and can be expressed as:[39]

$$\frac{1}{T_{1M}} = \frac{1}{T_{2M}} = \frac{4}{3}\left(\frac{\mu_0}{4\pi}\right)^2 \frac{\gamma^2 g_e^2 \mu_B^2 S(S+1)}{r^6}\tau_c\,, \qquad \textit{Eq. S26}$$

where μ_0 – vacuum magnetic permeability; γ – nuclear gyromagnetic ratio; g_e– isotropic g-value of the complex; μ_B – Bohr magneton; S – spin number of the complex; r – distance between nuclear and electronic spins (for point dipole approximation, M···H distance); τ_c – correlation time for electron-nucleus interaction.

An important consequence of the *Eq. S26* is that the signal linewidth, which is inversely proportional to transverse relaxation time constant T_{2M}, is also inversely proportional to r^6, which allows to roughly assign paramagnetic NMR signals from their linewidth.[36,37] However, the exact linear dependence may be disturbed by the ligand-centered dipolar relaxation or intramolecular rotations. Another important consequence is that linewidth grows quadratically with the number of unpaired electrons. In other words, out of two spin states with similar geometry, the state with higher multiplicity will give broader signals.

S13.1.2 Assignments of paramagnetic NMR signals

In general, the strategies used for assigning NMR signals in diamagnetic molecules are not applicable for paramagnetic species. Mainly, due to a much larger range of chemical shifts and the absence of fine structure due to line broadening. Despite that, a number of additional strategies are available based on the understanding of the origin of paramagnetic chemical shifts (see *Section S13.1.1*). The most common of them are as follows:[33]

1. Signal widths. As stated in *Section S13.1.1*, if dipolar relaxation is predominant, which is usually the case, the peak full width at half maximum should be inversely proportional to the sixth power of the distance between the resonating proton and the paramagnetic center ($\Delta\nu_{1/2} \sim r^{-6}$). However, the exact linear dependence may be disturbed by the ligand-centered dipolar relaxation or intramolecular rotations. Still, as a rule of thumb, the broader of the two signals can be assigned to the proton that is closer to a paramagnetic center.
2. Signal areas. Despite unusual chemical shift range, the peak integral intensities are still proportional to the number of resonating protons. However, the integration may be complicated due to the extreme line broadening and residual "ringing" from the excitation pulse,[36] which may distort the base line.
3. (Isotopic) Substitution. Systematic substitution of certain protons in the molecule (mostly, for methyl groups or deuteria) may allow to assign some signals.
4. Signal shifts. Oftentimes, one can make an educated guess as for the mechanism of spin delocalization, which may allow to deduce the sign of a hyperfine shift.[33,36] High-level spin density calculations can be helpful in such situations.
5. Temperature dependence. As discussed in *Section S13.1.1*, paramagnetic NMR shifts are extremely temperature-dependent. Given strong domination of the Fermi contact term (δ^C), which is normally the case for covalent d-block compounds, observed shift δ^{obs} should obey the following law: $\delta^{obs} = \delta^{dia} + \delta^C = \delta^{dia} + \frac{C}{T}$. In other words, the dependence of δ^{obs} from $\frac{1}{T}$ should be linear. In such a case, extrapolation to $\frac{1}{T} = 0$ or $T \to \infty$ will eliminate the contact component and reveal the diamagnetic contribution (δ^{dia}). The latter can, in turn, be compared with the shifts of the diamagnetic analogue of the paramagnetic compound in question.
6. Internuclear couplings. As mentioned above, paramagnetic relaxation is fast, which precludes $J_{H,H}$ coupling from evolving sufficiently long to be seen, with sporadic exceptions.[50] Contrarily, signal assignment can often be aided using $J_{C,H}$ couplings discernable from the ^{13}C NMR spectra.
7. Nuclear Overhauser Effect (NOE), saturation transfer and correlation spectra in general. In some cases, for relatively narrow signals, one can obtain correlation spectra that will aid the assignment. These techniques have been worked out in great detail for bioinorganic molecules.[37,51,52]
8. Redox titration. If a paramagnetic species can be synthesized by oxidation/reduction of the diamagnetic precursor, gradual oxidation/reduction of the said precursor may

allow to match paramagnetic signals to the assigned signals in the precursor, provided the redox equilibrium is fast on the NMR measurement timescale.

S13.2 $[(TSMP)_2Fe^{III}]^-$ (3) complex

S13.2.1 Signal assignments

In the assignment of the paramagnetic NMR signals in the $[(TSMP)_2Fe^{III}]^-$ complex (**3**), we use the line width argument, signal areas and isotopic substitution (see *Section S13.1.2*). More specifically, in the ^{1}H NMR spectrum of $[(TSMP)_2Fe^{III}]$K salt (**3a**) in acetonitrile-d_3 at 298 K (*Figure S22*), one can see five out of six expected paramagnetically shifted signals. The missing sixth signal can be found in the spectrum of $[(TSMP)_2Fe^{III}]PPh_4$ (**3b**) salt at 173K in dichloromethane-d_2 (*Figure S23*), where **3b** is more soluble that **3a**. The signal shows as a broad (FWHM >5 ppm) singlet at -36.0 ppm, and is only visible because the $[(TSMP)_2Fe^{III}]^-$ (**3**) anion almost completely crosses over into the low-spin state (2.1% of the high-spin contribution as estimated from measurements according to Evans mehod, see *Sections S6.2*). Due to a smaller number of unpaired electrons, this leads to less efficient dipolar relaxation (see *Eq. S26*) and, thus, narrower line width. Being the broadest in the entire spectrum, this signal should belong to a proton that is by far the closest to the Fe center, assuming there is no significant spin delocalization to the ligand. Computationally optimized geometry of the low-spin (S = $^1/_2$) state at the B3LYP-D3BJ/def2-TZVP level of theory (*Figure S24*) shows that H^7 at 3.27 Å is the likeliest source of the signal with the second closest protons, CH_3^P, being 5.43 Å away from the metal.

Next, among five paramagnetically shifted signals in the spectrum of $[(TSMP)_2Fe^{III}]$K (**3a**; *Figure S22*), only one at 67.8 ppm has triple integral intensity and can be unabiguously assigned to the aryl-bound methyl groups (CH_3^{Ar}). Another signal at 12.3 ppm is not present in the ^{1}H spectrum of the deuterium-labelled analogue **3a-*d₆*** but shows in the corresponding ^{2}H spectrum, and, thus, belongs to the phosphonium-bound methyl group (CH_3^P). The three remaining signals, one at 27.5ppm and two between 0 and -10 ppm, belong to one proton each and have distinctly different width. Despite the spectrum reflecting a mid-spin-crossover situation (80.8% according to Evans method, see *Sections S6.2*), the relative ordering of the Fe···H distances in the the calculated geometries of both spin states retains (*Figure S24*). Therefore, assuming there is no significant spin delocalization to the ligand in either of the spin states and that the rate of spin-crossover is much faster than any of the Larmor frequencies,[a] we assign the remaining signals inversely to the respective Fe···H distances as H^4, H^6 and H^5.

[a] The assumption that the rate of the SCO is faster than nuclear Larmor frequencies is justified. Unless geometries of the involved spin states differ significantly,[55] SCO occurs at picosecond time scale (THz frequencies). This is six orders of magnitude faster than ^{1}H Larmor frequencies (MHz range) on modern NMR machines.

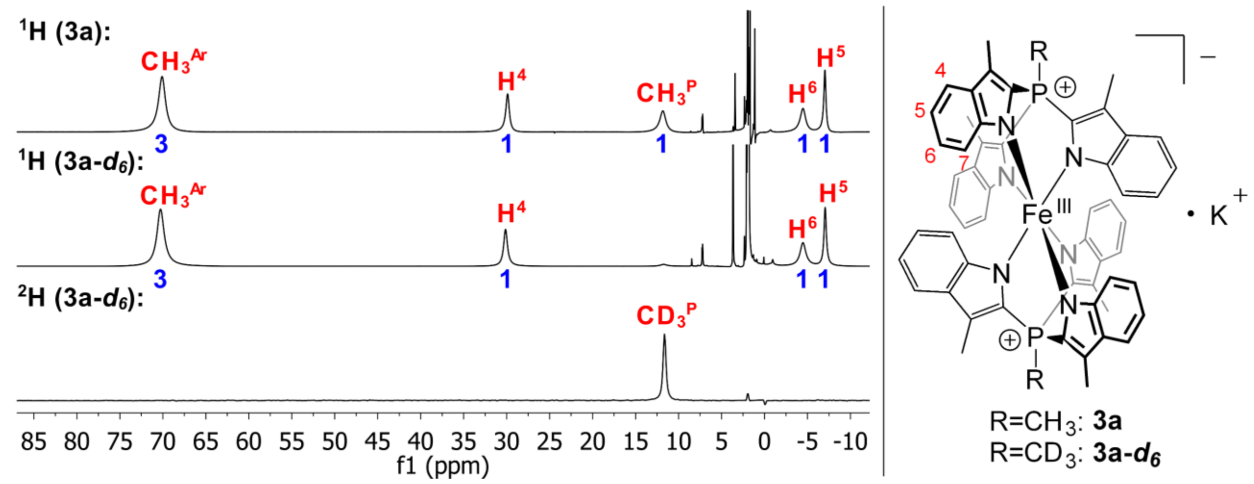

Figure S22. Stacked ^{1}H (400 MHz) and ^{2}H (61 MHz) NMR spectra of **3a** and its deuterated analogue ***3a-d₆*** in acetonitrile at 298 K. Only paramagnetic signals are assigned. The integrals are given in blue and were rounded to the nearest integer.

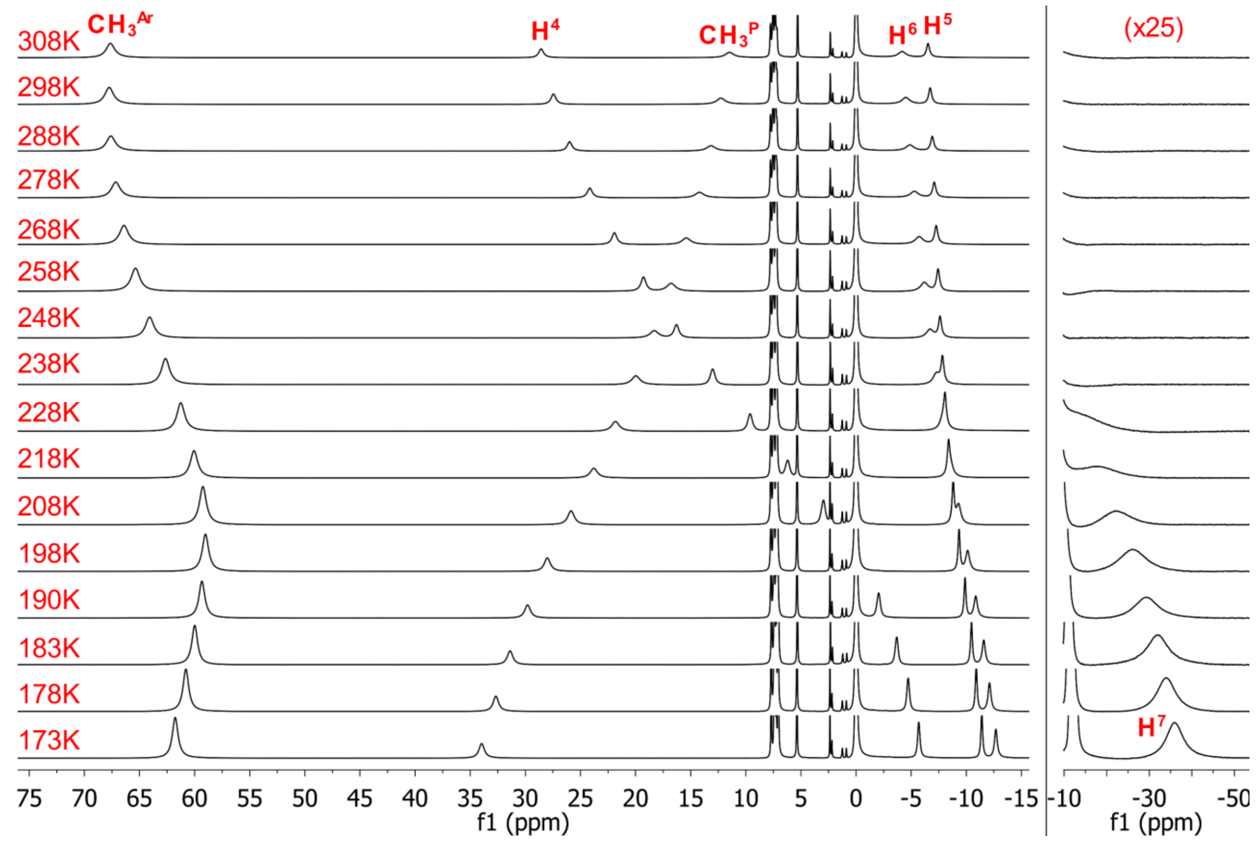

Figure S23. Stacked variable-temperature ^{1}H (400 MHz) NMR spectra of **3b** in dichloromethane-d_2. Only paramagnetic signals are assigned.

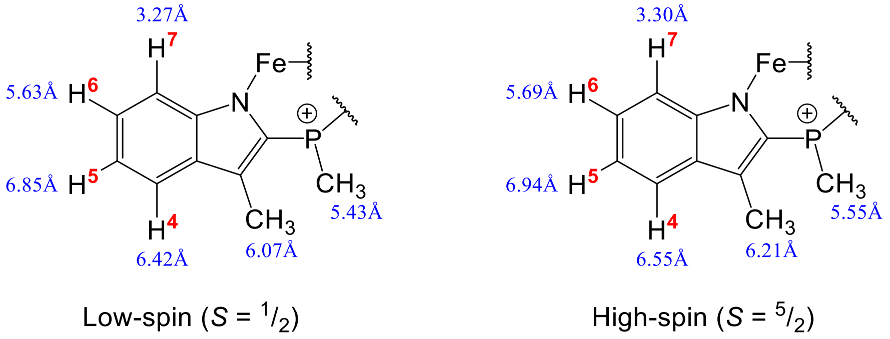

Figure S24. Calculated Fe···H distances in low- and high-spin geometries of $[(TSMP)_2Fe^{III}]^-$ (**3**) optimized at B3LYP-D3BJ/def2-TZVP level of theory in vacuum. The average values for methyl protons were derived by arithmetic averaging.

S13.2.2 Variable-temperature behavior

Variable-temperature ^{1}H NMR chemical shifts of the $[(TSMP)_2Fe^{III}]^-$ (**3**) complex in different solvents (*Figure S23*, *Figure S25*, *Figure S26*) are consistent with the ongoing SCO. To elaborate, the hyperfine parts (δ^{HF}) of the observed shifts, obtained by subtraction of the diamagnetic contributions approximated using an isostructural $[(TSMP)_2Ga^{III}]^-$ (**5a**) analogue (*Table S7*, *Table S8*, *Table S9*), do not obey the Curie's law ($\delta^{HF}T \neq const$; *Figure S27*). Moreover, temperature behavior of the $\delta^{HF}T$ product closely follows the SCO curves obtained by Evans method (*Figure S10*), implying that the hyperfine shifts are heavily dominated by the Fermi contact term (δ^{FC}), as expected for covalent *d*-block compounds.[53]

The signals in the discussed ^{1}H spectra become sharper in all three solvents at either end of the SCO curve. At high temperatures in acetonitrile-d_3 (*Figure S25*) and, especially, pyridine-d_5 (*Figure S26*), the system is close to completely crossing over to the high-spin state (*Figure S10*). In these circumstances, despite more efficient dipolar relaxation with a higher number of unpaired electrons (see *Eq. S26*), faster temperature-induced electronic relaxation leads to slower nuclear relaxation, causing somewhat sharper signals. Contrarily, at low temperatures in dichloromethane-d_2 (*Figure S23*), the system is close to completely crossing over to the low-spin state (*Figure S10*). Even though low temperatures should prolong electronic relaxation and broaden the signals, dipolar nuclear relaxation, which generally dominates such broadening, is less efficient with fewer unpaired electrons (see *Eq. S26*). As a result, the SCO influence prevails, leading to sharper signals at lower temperatures.

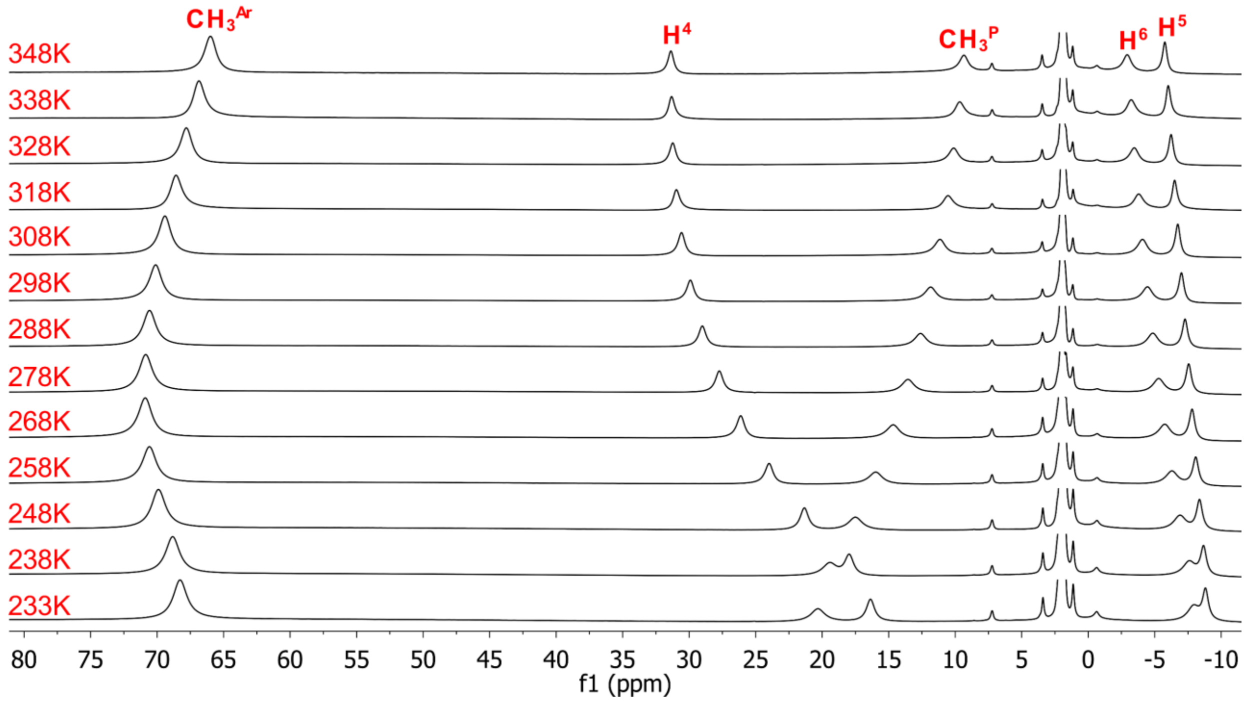

Figure S25. Stacked variable-temperature ^{1}H (400 MHz) NMR spectra of **3a** in acetonitrile-d_3. Only paramagnetic signals are assigned.

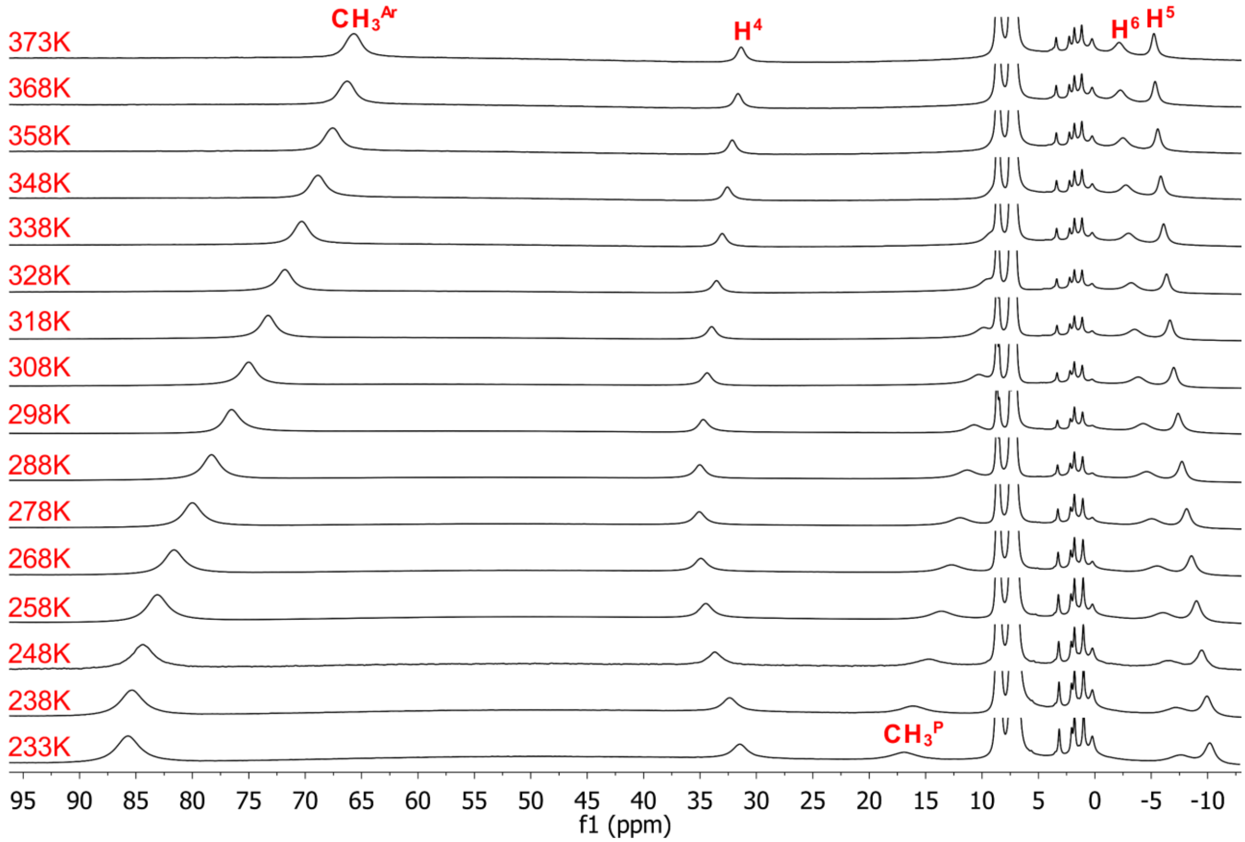

Figure S26. Stacked variable-temperature ^{1}H (400 MHz) NMR spectra of **3a** in pyridine-d_5. Only paramagnetic signals are assigned.

Table S7. Variable-temperature ^1H NMR (400 MHz) chemical shifts of $[(TSMP)_2Fe^{III}]PPh_4$ (**3b**) salt in dichloromethane-d_2. Diamagnetic shifts (assumed to be temperature-independent) were measured at 298 K on an isostructural $[(TSMP)_2Ga^{III}]K$ (**5a**) complex solubilized in the same solvent in the presence of a subequivalent amount of PPh_4I (see *Section S2.1*).

**T, K**	**Chemical shift, ppm**					
	**CH_3^{Ar}**	**H^4**	**CH_3^P**	**H^6**	**H^5**	**H^7**
308	67.6	28.6	11.5	-4.2	-6.5	-
298	67.8	27.5	12.3	-4.5	-6.7	-
288	67.6	26.0	13.2	-4.9	-6.9	-
278	67.2	24.2	14.2	-5.3	-7.1	-
268	66.4	21.9	15.4	-5.7	-7.3	-
258	65.4	19.3	16.8	-6.2	-7.4	-
248	64.1	16.3	18.3	-6.7	-7.6	-
238	62.6	13.0	20.0	-7.3	-7.8	-
228	61.3	9.6	21.8	-8.1	-8.1	-
218	60.1	6.2	23.8	-8.4	-8.4	-18.3
208	59.3	3.0	25.9	-9.3	-8.8	-22.3
198	59.0	0.5	28.0	-10.1	-9.4	-26.0
190	59.3	-2.1	29.8	-10.9	-9.9	-29.2
183	60.0	-3.7	31.4	-11.6	-10.5	-32.0
178	60.8	-4.7	32.7	-12.1	-10.9	-34.0
173	61.8	-5.7	34.0	-12.7	-11.4	-36.0
diamagnetic	2.78	7.11	3.47	5.55	6.15	5.21

Table S8. Variable-temperature ^1H NMR (400 MHz) chemical shifts of $[(TSMP)_2Fe^{III}]K$ (**3a**) salt in acetonitrile-d_3. Diamagnetic shifts (assumed to be temperature-independent) were measured at 298 K on an isostructural $[(TSMP)_2Ga^{III}]K$ (**5a**) complex in the same solvent.

**T, K**	**Chemical shift, ppm**				
	**CH_3^{Ar}**	**H^4**	**CH_3^P**	**H^6**	**H^5**
348	66.0	31.4	9.3	-3.0	-5.8
338	66.9	31.3	9.7	-3.2	-6.0
328	67.8	31.2	10.1	-3.5	-6.3
318	68.6	31.0	10.6	-3.8	-6.5
308	69.4	30.6	11.1	-4.1	-6.8
298	70.1	29.9	11.8	-4.5	-7.0
288	70.6	29.0	12.6	-4.9	-7.3
278	70.8	27.7	13.5	-5.3	-7.6
268	70.9	26.1	14.7	-5.8	-7.8
258	70.6	24.0	16.0	-6.3	-8.1
248	69.9	21.3	17.5	-6.9	-8.4
238	68.8	18.0	19.4	-7.6	-8.7
233	68.3	16.3	20.3	-8.0	-8.8
diamagnetic	2.79	7.12	3.57	5.46	6.15

Table S9. Variable-temperature ^{1}H NMR (400 MHz) chemical shifts of [(TSMP)$_2$Fe^{III}]K (**3a**) salt in pyridine-d_5. Diamagnetic shifts (assumed to be temperature-independent) were measured at 298 K on an isostructural [(TSMP)$_2$Ga^{III}]K (**5a**) complex in the same solvent.

T, K	Chemical shift, ppm				
	CH_3^{Ar}	H^4	CH_3^P	H^6	H^5
373	65.8	31.4	-	-2.1	-5.2
368	66.3	31.7	-	-2.2	-5.3
358	67.6	32.2	-	-2.5	-5.5
348	69.0	32.7	-	-2.7	-5.8
338	70.4	33.1	9.4	-3.0	-6.1
328	71.8	33.6	9.7	-3.2	-6.3
318	73.3	34.0	10.0	-3.5	-6.6
308	75.1	34.4	10.3	-3.9	-7.0
298	76.6	34.8	10.8	-4.2	-7.4
288	78.3	35.1	11.4	-4.6	-7.7
278	80.0	35.1	12.0	-5.0	-8.1
268	81.7	35.0	12.8	-5.5	-8.5
258	83.2	34.5	13.7	-6.0	-9.0
248	84.4	33.7	14.8	-6.6	-9.4
238	85.4	32.4	16.3	-7.2	-9.9
233	85.8	31.5	17.0	-7.7	-10.1
diamagnetic	2.85	7.40	3.74	6.25	6.34

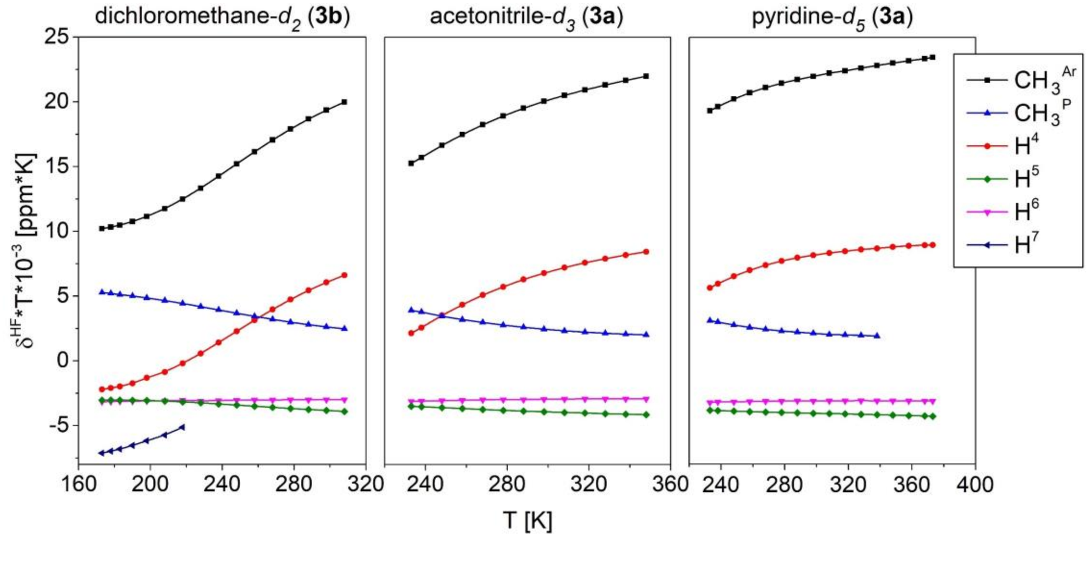

Figure S27. Variable-temperature ^{1}H NMR (400 MHz) hyperfine shift times temperature products of complex [(TSMP)$_2$Fe^{III}]$^-$ (**3**) in different solvents. Due to solubility considerations, [(TSMP)$_2$Fe^{III}]K (**3a**) salt was used for experiments in acetonitrile-d_3 and pyridine-d_5, and [(TSMP)$_2$Fe^{III}]PPh$_4$ (**3b**) salt was measured in dichloromethane-d_2.

S13.3 [(TSMP)$_2$Fe^{IV}] (4) complex

S13.3.1 Signal assignments

The ^{1}H NMR spectrum of complex [(TMSP)$_2$Fe^{IV}] (**4**) in DCM-d_2 at 298K shows six paramagnetically shifted and broadened signals (*Figure S28*), as expected for C$_3$-symmetric heterobicyclo[2.2.2]octane topology of the TSMP scaffold. These signals can be assigned using the set of arguments outlined in *Section S13.1.2*. To elaborate, out of six signals, only the one at 145.4 ppm has triple integral intensity and, thus, can be unabiguously attributed to the aryl-bound methyl groups (CH$_3^{Ar}$). Next, the peak at 7.2 ppm is absent in the ^{1}H spectrum of the deuterium-labelled analogue **4-*d*$_6$** but shows in the corresponding ^{2}H spectrum, which indicates that it belongs to a phosphonium-bound methyl group (CH$_3^P$). A broad signal at -27.4 ppm clearly corresponds to a proton that is by far the closest to the source of the magnetic moment in the molecule. Considering that the spin density is mainly localized on the Fe atom (see the main text), this means that the signal belongs to the proton with the shortest Fe···H distance, i. e. H^7 as can be deduced from the molecular geometry optimized at PBE-D3BJ/def2-TZVP level of theory (*Figure S29*). The spectral assignment can be completed by inversely matching the full widths at half maximum (FWHM) of the remaining signals to the calculated Fe···H distances for unassigned protons. More specifically, the relatively sharp signals between -10 and -35 ppm have the FWHM of 156.2, 251.0 and 228.7 Hz from left to right,[a] which allows to assign them as H^5, H^6 and H^4.

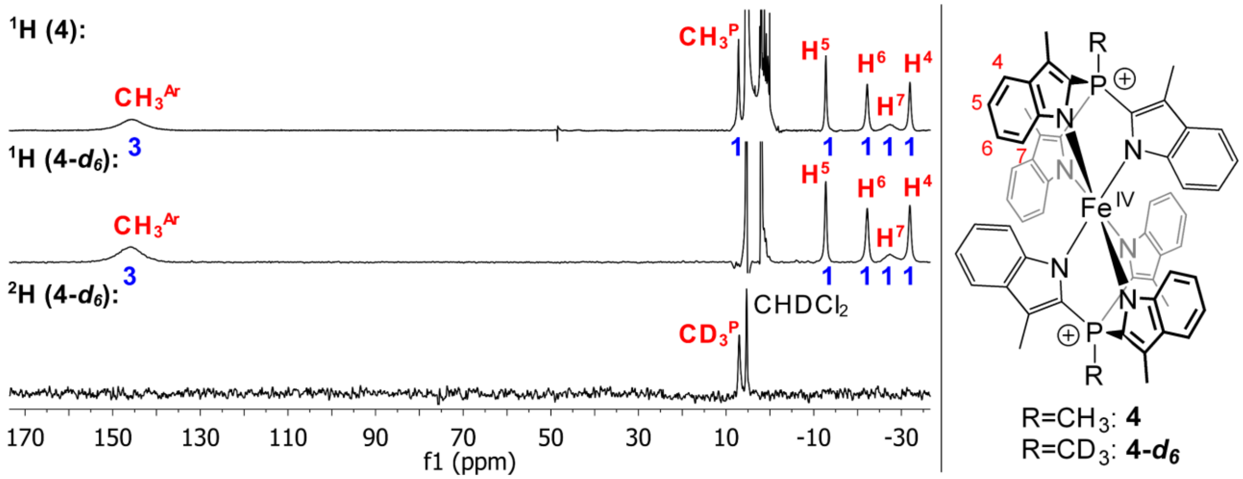

Figure S28. Stacked ^{1}H (400 MHz) and ^{2}H (61 MHz) NMR spectra of **4** and its deuterated analogue **4-*d*$_6$** in dichloromethane at 298 K. Only paramagnetic signals are assigned. The integrals are given in blue and were rounded to the nearest integer.

[a] Signal linewidths were extracted using MNova peak deconvolution tool.[2]

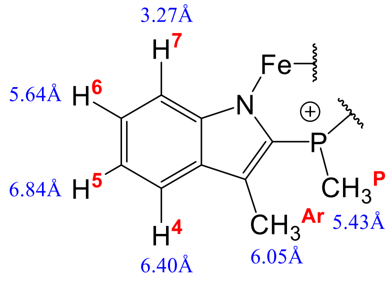

Figure S29. Calculated Fe···H distances in [$(TSMP)_2Fe^{IV}$] (**4**) optimized at PBE-D3BJ/def2-TZVP level of theory in vacuum. The average values for methyl protons were derived by arithmetic averaging.

S13.3.2 Variable-temperature behavior

Variable-temperature ^{1}H NMR chemical spectra of **4** are shown in *Figure S30*. The spread of chemical shifts becomes monotonously narrower at increased temperatures, which is typical for paramagnetic compounds. The temperature-dependent signal linewidths also evolve in the way one would expect for a paramagnetic system: lower temperatures result in broader signals due to slower electronic relaxation causing faster nuclear relaxation and vice versa. The hyperfine shifts (δ^{HF}), obtained by subtraction of the diamagnetic contributions approximated using an isostructural [$(TSMP)_2Ga^{III}$]$^-$ (**5**) analogue (*Table S10*), follow Curie's law. This is evident from the $\delta^{HF}T$ product being nearly temperature-independent for most signals (*Figure S31*), ruling out the possibility of SCO. The small resudual slope for some signals can be attributed to incomplete subtraction of diamagnetic components since the [$(TSMP)_2Ga^{III}$]$^-$ (**5**) model only approximates δ^{dia}, or to a very small pseudocontact contribution. The only exception is the CH_3^{Ar} signal, whose slope is relatively noticeable, likely due to incomplete rotational averaging of the chemical shifts of separate protons. Yet, overall, since the hyperfine shifts do not show a $1/T^2$ dependence, the pseudocontact contribution can be considered very low (see *Section S13.1.1*), being consistent with isotropic g-tensor, moderate zero-field splitting of +19.1 cm^{-1} and very low rhombicity as derived from SQUID magnetometry and THz-EPR studies as well as computational studies (see main text).

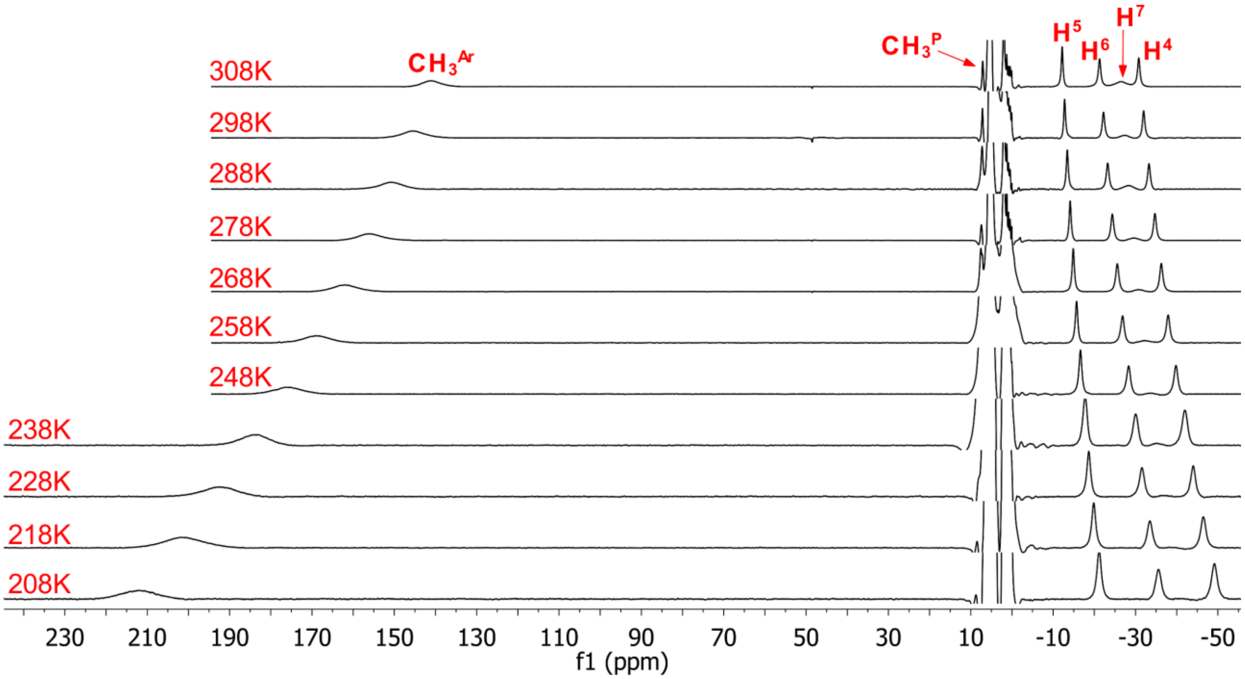

Figure S30. Stacked variable-temperature ^{1}H (400 MHz) NMR spectra of **4** in dichloromethane-*d*$_2$. Only paramagnetic signals are assigned.

Table S10. Variable-temperature ^{1}H NMR (400 MHz) chemical shifts of [(TSMP)$_2$Fe^{IV}] (**4**) in dichloromethane-d_2. Some chemical shifts could not be reliably extracted due to extreme line broadening at low temperatures or the presence of an intense solvent signal in the vicinity. Diamagnetic shifts (assumed to be temperature-independent) were measured at 298 K on an isostructural [(TSMP)$_2$Ga^{III}]K (**5a**) complex solubilized in dichloromethane-d_2 in the presence of a subequivalent amount of PPh$_4$I (see *Section S2.1*).

**T, K**	**Chemical shift, ppm**					
	**CH_3^{Ar}**	**CH_3^{P}**	**H^5**	**H^6**	**H^7**	**H^4**
308	141.0	7.1	-12.2	-21.3	-26.4	-30.8
298	145.4	7.2	-12.8	-22.2	-27.4	-32.0
288	151.0	7.2	-13.4	-23.3	-28.4	-33.3
278	156.2	7.4	-14.1	-24.4	-29.6	-34.7
268	162.0	7.6	-14.9	-25.6	-30.8	-36.3
258	168.9	7.7	-15.7	-26.9	-32.2	-37.9
248	176.1	7.8	-16.6	-28.3	-33.6	-39.8
238	184.2	-	-17.7	-30.0	-35.1	-42.0
228	192.5	8.1	-18.7	-31.5	-	-44.0
218	201.4	-	-19.9	-33.5	-	-46.5
208	211.7	-	-21.2	-35.6	-	-49.2
diamagnetic	2.78	3.47	6.15	5.55	5.21	7.11

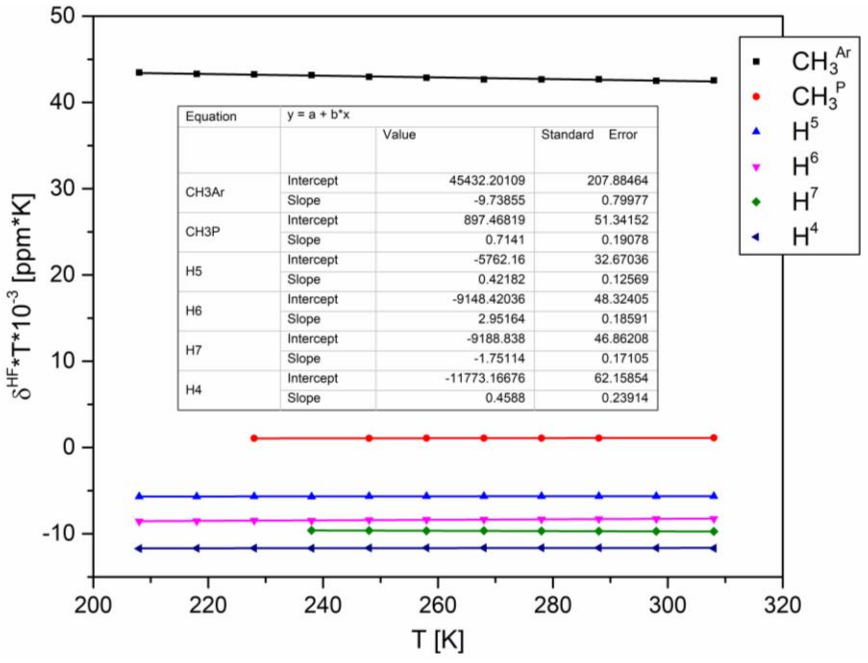

Figure S31. Variable-temperature ^{1}H NMR (400 MHz) $\delta^{HF}T$ products of complex [(TSMP)$_2$Fe^{IV}] (**4**) in dichloromethane-d_2 and its linear fits. Dots show experimental values, straight lines show the fits.

S13.3.3 Partitioning of hyperfine shifts

An additional confirmation as for the low magnitude of the pseudocontact shifts can be obtained by fitting observed chemical shifts (δ^{obs}) of **4** within the framework of the point dipole approximation (see *Section S13.1.1*) combined with DFT calculations. The equation for δ^{obs} can be written as follows:

$$\delta^{obs} = \delta^{dia} + \delta^{HF} = \delta^{dia} + \delta^{FC} + \delta^{PC}, \qquad \textit{Eq. S27}$$

$$\delta^{obs} = \delta^{dia} + \frac{S(S+1)\mu_B}{3kTg_N\mu_N} g_{iso} \cdot A_{iso} + \frac{1}{12\pi r^3}\left[\Delta\chi_{ax}(3cos^2\theta - 1) + \frac{3}{2}\Delta\chi_{rh} sin^2\theta cos2\varphi\right], \qquad \textit{Eq. S28}$$

where, δ^{dia} – diamagnetic shift; δ^{HF}– hyperfine shift; δ^{FC} – Fermi contact shift; δ^{PC} – pseudocontact shift; S – electronic spin quantum number of the complex; μ_B – Bohr magneton; g_N – nuclear g-value; μ_N – nuclear magneton; k – Boltzmann constant; T – temperature; g_{iso} – isotropic electronic g-value of the system; A_{iso} – isotropic hyperfine coupling constant; r, θ and φ – spherical coordinates of a nucleus within the intrinsic frame of magnetic susceptibility tensor; $\Delta\chi_{\mathrm{ax}}$ and $\Delta\chi_{\mathrm{rh}}$ – axial anisotropy and rhombicity of the above tensor.

Since according to SQUID magnetometry and THz-EPR studies (see main text), the zero-field splitting parameter E is close to zero, magnetic susceptibility rhombicity $\Delta\chi_{\mathrm{rh}}$ also becomes vanishingly small (see *Eq. S22* and *Eq. S25*), allowing to simplify *Eq. S28* as follows:

$$\delta^{obs} = \delta^{dia} + \frac{S(S+1)\mu_B}{3kTg_N\mu_N} g_{iso} \cdot A_{iso} + \frac{1}{12\pi r^3}\Delta\chi_{ax}(3cos^2\theta - 1). \qquad \textit{Eq. S29}$$

Out of all terms and coefficients in the equation, δ^{dia} and S are known experimentally (the former are approximated using an isostructural $[(TSMP)_2Ga^{III}]^-$ (**5**) analogue, the latter equals one according to SQUID magnetometry); μ_B, k, g_N and μ_N are constants; r and θ are geometric parameters that can be extracted from a DFT-optimized structure; A_{iso} and g_{iso} can also be either calculated from DFT or measured experimentally. This leaves only one parameter to be fitted, $\Delta\chi_{ax}$, which defines the strength of the pseudocontact contribution.

Following the symmetry of the spin Hamiltonian for $[(TSMP)_2Fe^{IV}]$ (**4**), the principal axis of the magnetic susceptibility tensor has to coincide with the molecular axis of symmetry of the highest order, which is a C_3-axis passing through the P–Fe–P atoms. Graphically it can be represented as shown in *Figure S32*, which features a plane with two out of six symmetry-equivalent indolide rings within the D_{3d} point group. The principal axis (z) points from the iron atom towards one of the phosphorus atoms. The polar angle θ is measured with respect to this axis and the origin of coordinates, which is a metal center. The grey plot in polar coordinates in the background corresponds to the $3cos^2\theta - 1$ function modulating the magnitude of the pseudocontact shift as can be seen from the *Eq. S29*. This function changes its sign at the magic angle (ca. 54.7°) with respect to the principal axis, thus also flipping the sign of the pseudocontact shift.

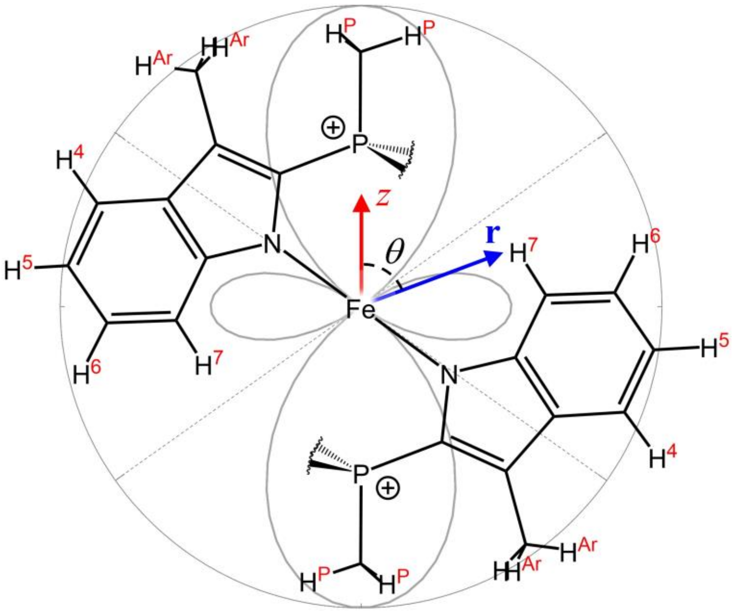

Figure S32. Graphical representation of the molecular geometry of **4** optimized at PBE-D3BJ/def2-TZVPP level of theory; angles and relative interatomic distances match the calculated values. Only two out of six symmetry-equivalent (D_{3d} point group) indolide rings are shown. The hydrogens of the CH_3^{Ar} and CH_3^{P} methyl groups are slightly out of plane but are still shown for clarity. The principal axis of the magnetic susceptibility tensor is given in red. The polar plot in the background corresponds to the $3cos^2\theta - 1$ function.

Table S11. Results of geometry optimization of **4** at PBE-D3BJ/def2-TZVPP level of theory followed by properties calculation at the PBE0-D3BJ/def2-TZVPP level. No symmetry constraints were imposed on a molecule, which results in small numerical differences between symmetry-equivalent positions. Numbers following underscores indicate separate aromatic rings. The average values (av) were obtained by arithmetic averaging.

Proton	r, Å	r_{av}, Å	$cos\theta$	$\|cos\theta\|_{av}$	A_{iso}, MHz	$\delta^{FC}T$, ppm·K	$\delta^{FC}T_{av}$, ppm·K
H^4_1	6.40	6.40	0.42	0.42	-0.733	-15179.11	-15182.22
H^4_2	6.40		0.42		-0.732	-15170.82	
H^4_3	6.40		0.42		-0.734	-15197.76	
H^4_4	6.40		-0.42		-0.734	-15197.76	
H^4_5	6.40		-0.42		-0.732	-15168.75	
H^4_6	6.40		-0.42		-0.733	-15179.11	
H^5_1	6.84	6.84	0.06	0.06	-0.280	-5809.75	-5780.74
H^5_2	6.84		0.06		-0.278	-5762.10	
H^5_3	6.84		0.06		-0.279	-5770.38	
H^5_4	6.84		-0.06		-0.279	-5770.38	
H^5_5	6.84		-0.06		-0.278	-5760.02	
H^5_6	6.84		-0.06		-0.281	-5811.82	
H^6_1	5.64	5.64	-0.29	0.29	-0.330	-6827.08	-6841.93
H^6_2	5.64		-0.29		-0.330	-6833.29	
H^6_3	5.64		-0.29		-0.331	-6866.45	
H^6_4	5.64		0.29		-0.331	-6866.45	
H^6_5	5.64		0.29		-0.330	-6833.29	
H^6_6	5.64		0.29		-0.329	-6825.01	

Table S10 (continued). Results of geometry optimization of **4** at PBE-D3BJ/def2-TZVPP level of theory followed by properties calculation at the PBE0-D3BJ/def2-TZVPP level. No symmetry constraints were imposed on a molecule, which results in small numerical differences between symmetry-equivalent positions. Numbers following underscores indicate separate aromatic rings. The average values (av) were obtained by arithmetic averaging.

Proton	r, Å	r_{av}, Å	$cos\theta$	$\|cos\theta\|_{av}$	A_{iso}, MHz	$\delta^{FC}T$, ppm·K	$\delta^{FC}T_{av}$, ppm·K	
H^7_1	3.27		-0.45		-0.556	-11509.69		
H^7_2	3.27		-0.45		-0.551	-11422.67		
H^7_3	3.27	3.27	-0.45	0.45	-0.553	-11453.74	-11461.69	
H^7_4	3.27		0.45		-0.553	-11453.74		
H^7_5	3.27		0.45		-0.551	-11420.59		
H^7_6	3.27		0.45		-0.556	-11509.69		
$CH_3{}^{Ar}{}_{a}$_1	5.85		0.87		2.332	48317.89		
$CH_3{}^{Ar}{}_{b}$_1	6.34		0.72		0.155	3211.52	43082.07	
$CH_3{}^{Ar}{}_{c}$_1	5.96		0.83		3.751	77716.81		
$CH_3{}^{Ar}{}_{a}$_2	6.33		0.72		0.212	4396.68		
$CH_3{}^{Ar}{}_{b}$_2	5.97		0.83		3.833	79413.73	43169.79	
$CH_3{}^{Ar}{}_{c}$_2	5.84		0.87		2.206	45698.95		
$CH_3{}^{Ar}{}_{a}$_3	5.96		0.83		3.754	77776.89		
$CH_3{}^{Ar}{}_{b}$_3	5.85		0.87		2.296	47567.85	42920.46	
$CH_3{}^{Ar}{}_{c}$_3	6.34	6.05	0.72	0.81	0.165	3416.65		43056.87
$CH_3{}^{Ar}{}_{a}$_4	5.96		-0.83		3.751	77723.02		
$CH_3{}^{Ar}{}_{b}$_4	6.34		-0.72		0.155	3219.81	43082.77	
$CH_3{}^{Ar}{}_{c}$_4	5.85		-0.87		2.331	48305.46		
$CH_3{}^{Ar}{}_{a}$_5	6.34		-0.72		0.165	3422.86		
$CH_3{}^{Ar}{}_{b}$_5	5.85		-0.87		2.295	47553.34	42917.70	
$CH_3{}^{Ar}{}_{c}$_5	5.96		-0.83		3.754	77776.89		
$CH_3{}^{Ar}{}_{a}$_6	5.84		-0.87		2.205	45682.37		
$CH_3{}^{Ar}{}_{b}$_6	5.97		-0.83		3.833	79415.81	43168.41	
$CH_3{}^{Ar}{}_{c}$_6	6.33		-0.72		0.213	4407.04		
$CH_3{}^{P}{}_{a}$	5.43		0.99		0.075	1549.82		
$CH_3{}^{P}{}_{b}$	5.43		0.99		0.075	1543.60		
$CH_3{}^{P}{}_{c}$	5.43	5.43	0.99	0.99	0.076	1568.47	1553.27	
$CH_3{}^{P}{}_{a}{}'$	5.43		-0.99		0.074	1541.53		
$CH_3{}^{P}{}_{b}{}'$	5.43		-0.99		0.075	1547.75		
$CH_3{}^{P}{}_{c}{}'$	5.43		-0.99		0.076	1568.47		

With these considerations in mind, we used the DFT-optimized structure of **4** to calculate the geometric parameters needed for fitting as well as Fermi contact shifts (δ^{FC}) in the form of temperature-independent Curie constants ($\delta^{FC}T$) (*Table S12*). The latter were derived using the experimental g-value of 1.97 obtained from THz-EPR studies (see main text), although the calculated g-value of 2.03 would give only marginally different results (see *Eq. S17*). The fits performed using the model described by *Eq. S29* reveal that $\Delta\chi_{\mathrm{ax}}$ is $2.5*10^{-32}$ m^3, which is very low. As a matter of fact, it is lower than the expected error of the DFT calculations, which precludes us from more detailed analysis using *Eq. S18-Eq. S23*. A separate fit performed for the protons of the $CH_3{}^P$ group, for which the pseudocontact shift should be the largest in the entire molecule (the lowest r_{av} and $|cos\theta|_{av}$ in *Table S12*), gives an even lower $\Delta\chi_{\mathrm{ax}}$ of $5.3\cdot10^{-33}$ m^3, which is comparable to $3.2*10^{-33}$ m^3 calculated by substituting the values derived from THz-EPR into *Eq. S21*.

In any case, since the fitted $\Delta\chi_{ax}$ is very small, the calculated observed shifts show a very good agreement with the experiment even without taking δ^{PC} into account (*Figure S33*), which also reinforces the paramagnetic signal assignments we made above (*Section S13.3.1*).

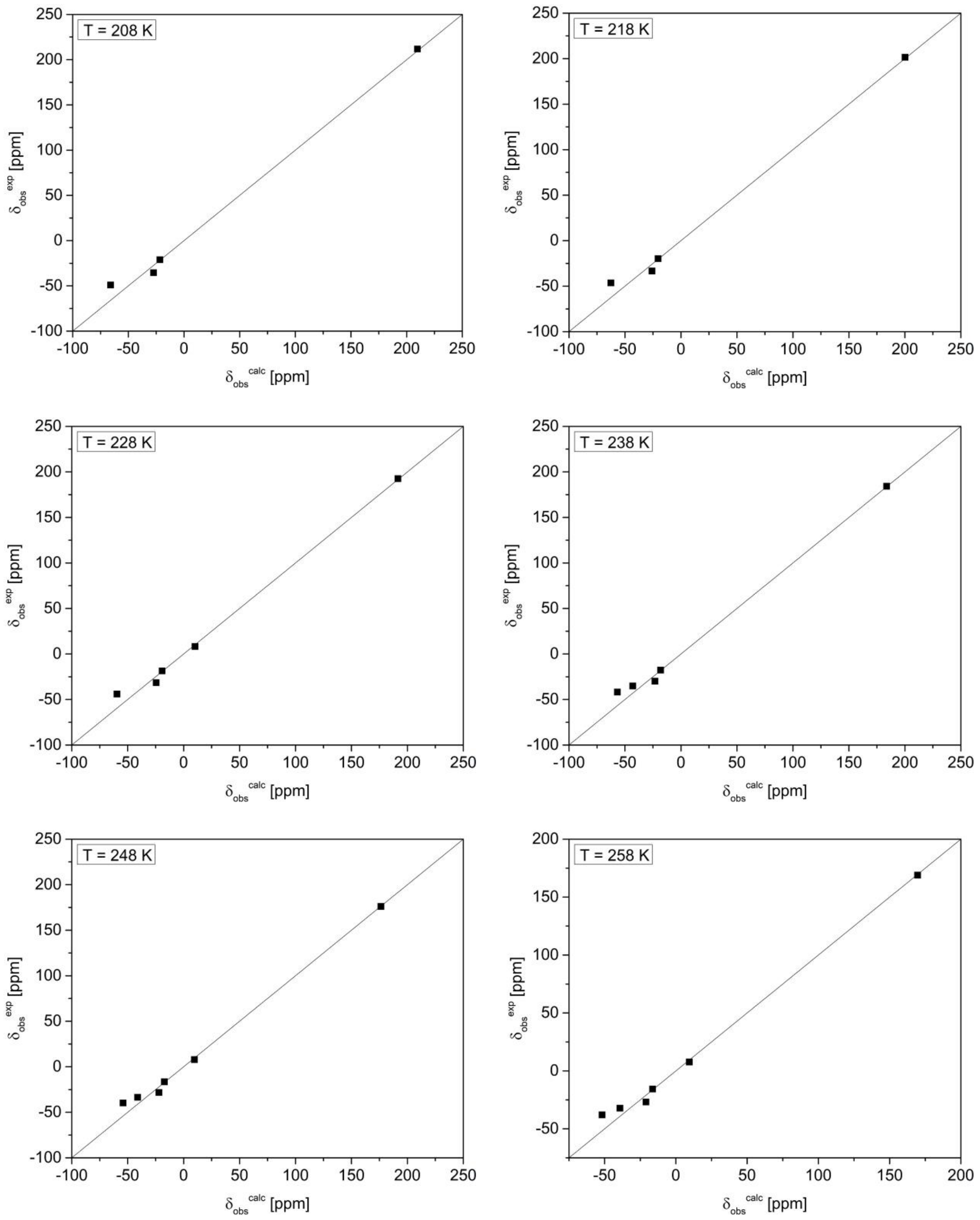

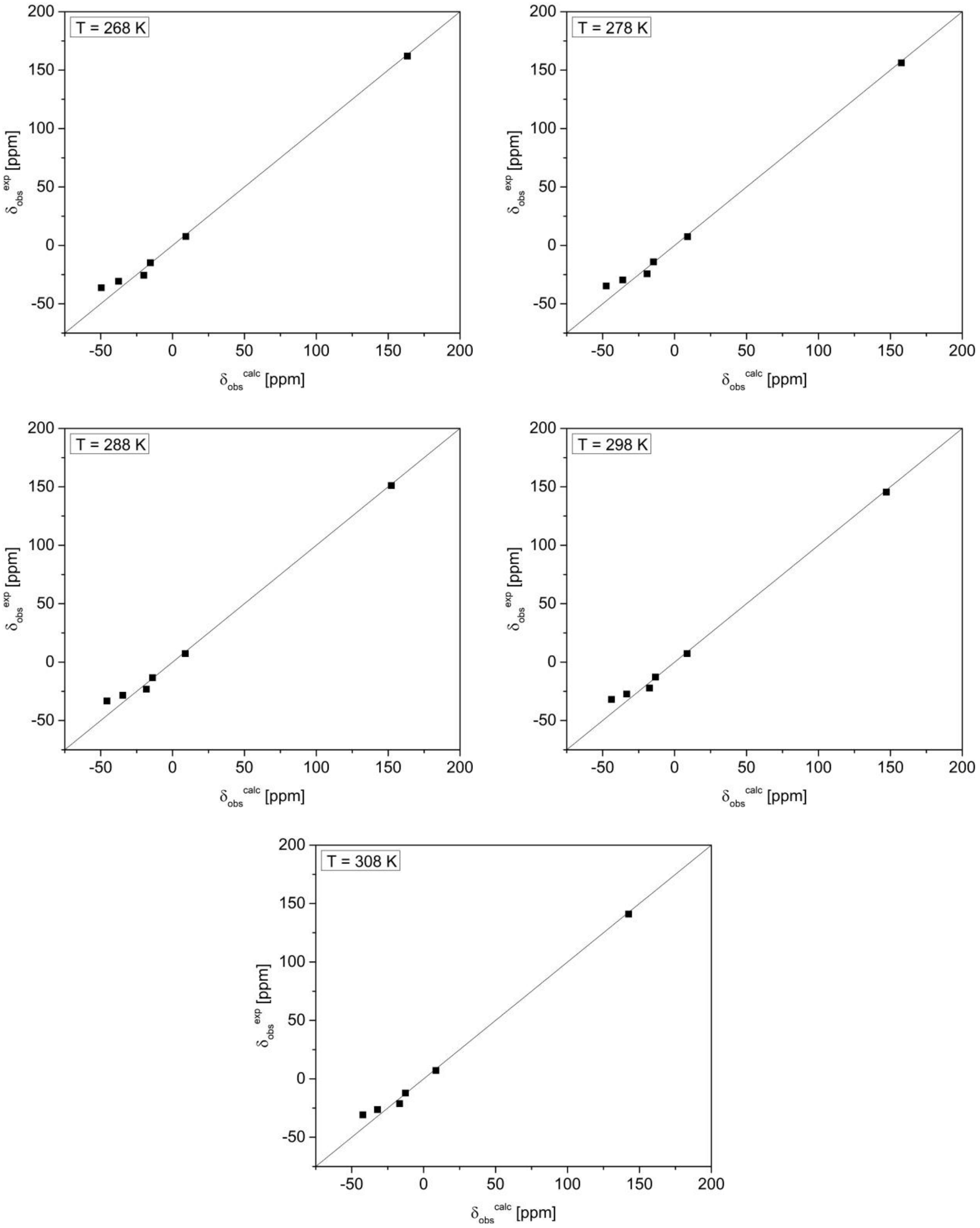

Figure S33. Correlation plots of experimental *vs.* calculated observed chemical shifts for complex **4**.

S14. Optical spectroscopy

S14.1 Variable-temperature UV-Vis spectra of [(TSMP)$_2$Fe^{III}]PPh$_4$ (3b)

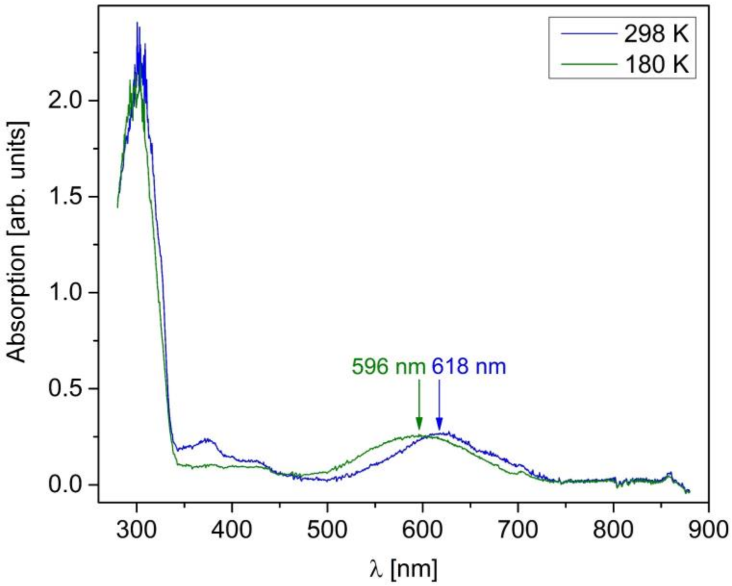

Figure S34. Change in UV-Vis spectrum of [(TSMP)$_2$Fe^{III}]PPh$_4$ (**3b**) in dichloromethane solution upon cooling. Visual color change is shown in *Figure S35*.

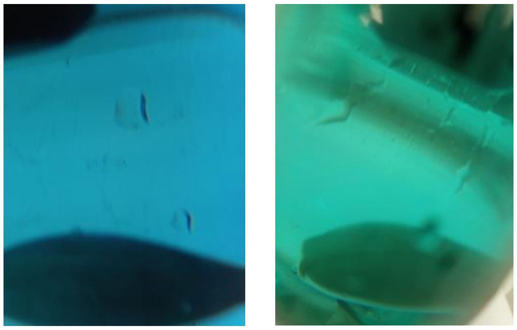

293 K 180 K

Figure S35. Color change of [(TSMP)$_2$Fe^{III}]PPh$_4$ (**3b**) in dichloromethane solution upon cooling.

S14.2 TDDFT calculations

Initial time-dependent density functional theory (TDDFT) calculations for **4** were performed in vacuum at three different levels of theory: TPSSh-D3BJ, B3LYP-D3BJ and PBE0-D3BJ with def2-TZVP basit set for Fe and def2-SVP set for all other atoms, employing optimized geometries from TPSSh-D3BJ/6-31G(d,p), B3LYP-D3BJ/6-31G(d,p) and PBE-D3BJ/6-31G(d,p) levels, respectively. Overall, all three functionals give the spectra that qualitatively reproduce the experiment (*Figure S36*), considering that only the first 50 excitations were

calculated. Nevertheless, the best agreement with the experiment is achieved when using TPSSh-D3BJ functional.

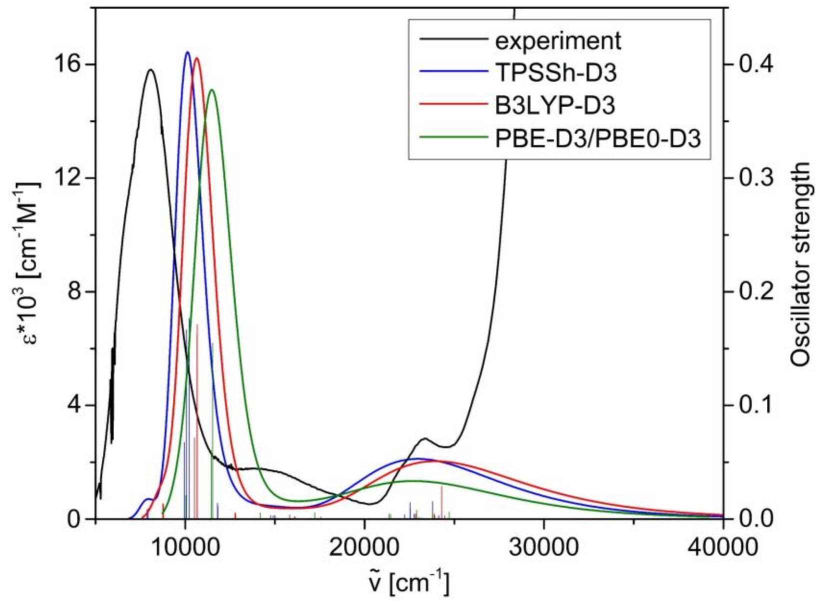

Figure S36. Experimental UV-Vis-NIR spectrum of **4** in dichloromethane solution and TDDFT transitions calculated using different functionals. In order to generate realistic spectra, Gaussian broadening with FWHM parameter of 170 nm was applied.

We further explored how calculated TDDFT transitions react to implicit solvation (PCM model) using the media with varied dielectric permeability (ε) from vacuum ($\varepsilon = \varepsilon_0$) to water ($\varepsilon = 80.4$) (*Figure S37*). Both the energies of the maxima and transition intensities are weakly sensitive to the medium.

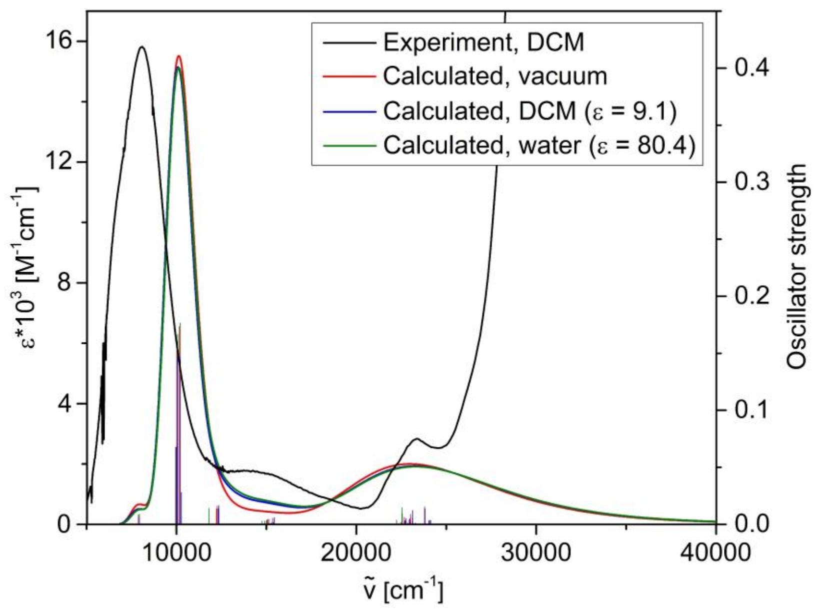

Figure S37. Experimental UV-Vis-NIR spectrum of **4** in dichloromethane solution and TDDFT transitions calculated in different media at TPSSh-D3BJ/def2-SVP (with def2-TZVP for Fe) level of theory. In order to generate realistic spectra, Gaussian broadening with FWHM parameter of 170 nm was applied.

Finally, we analyzed the structure of the calculated TDDFT transitions in more detail using Natural Transition Orbital (NTO) analysis.[54] This method performs separate unitary transformations of the occupied and virtual transition molecular orbitals so that only one or very few NTO donor-hole pairs are left with the predominant contribution to the transition. The most intense TDDFT transitions are labeled in *Figure S38* and expanded into the respective NTO donor-hole pairs in *Table S12*. These transitions can be roughly grouped in two categories: numbers 3 to 22 mostly correspond to $\pi \rightarrow d_{1e}$ ligand-to-metal charge transfer (LMCT), while numbers 40, 44 and 48 are a mixture of $\pi \rightarrow d_{2e}$ LMCT and $\pi \rightarrow \pi^*$ intra-ligand transitions (ICT). It is worth noting that ligand-based donor π- and aceptor π^*-orbitals are always pure combinations of 3-methylindole HOMO, HOMO-1 and LUMO-like orbitals (*Figure S39*) of separate indolide arms of the TSMP ligand. Hence, the notations in *Table S12*: $\pi_{(HOMO)_6}$, $\pi_{(HOMO-1)_6}$, and $\pi^*_{(LUMO)_6}$.

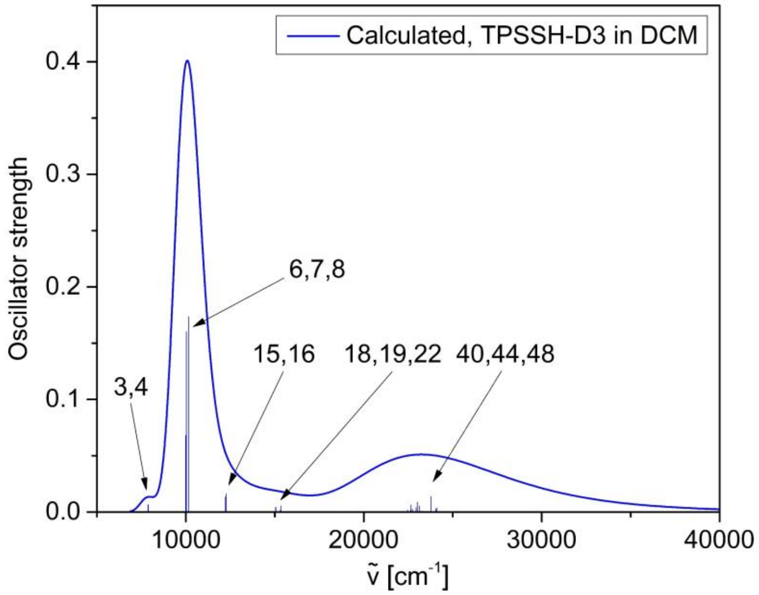

Figure S38. UV-Vis-NIR transitions in **4** calculated using TDDFT at TPSSh-D3BJ/def2-SVP (with def2-TZVP for Fe) level of theory in DCM. The most intense transitions are labeled in numbers and are further expanded upon in *Table S12*. In order to generate realistic spectrum, Gaussian broadening with FWHM parameter of 170 nm was applied.

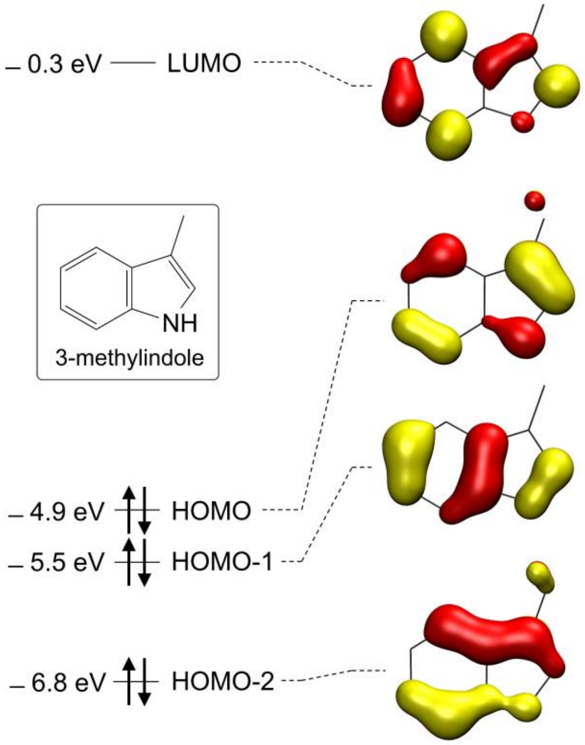

Figure S39. Molecular orbitals if 3-methylindole calculated at TPSSh-D3BJ/def2-SVP level of theory in vacuum. All orbitals have π-character, hence only a half of antisymmetric orbital lobes can be seen.

Table S12. Energy, difference density, NTO donor-hole pairs with the respective weights and assignments for the calculated TDDFT transitions labelled in *Figure S38*. All difference densities and NTO donor-hole pairs are shown as top- and sideways-projections.

**Transition number and energy [cm^{-1}]**	**Difference density**	**NTO donor**	**NTO hole**	**Weight**	**Type of transition**
3: 7873.7	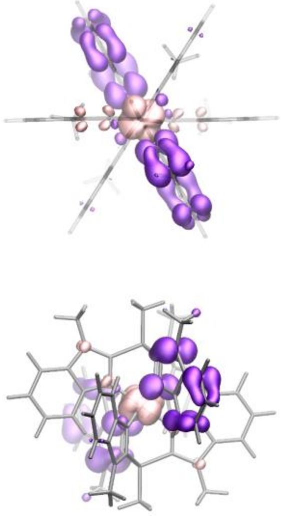	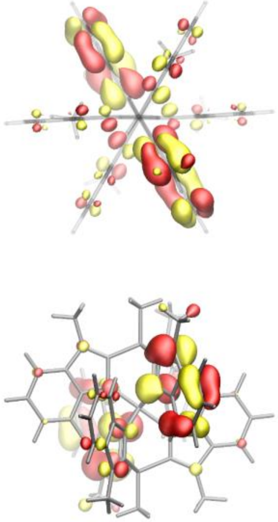	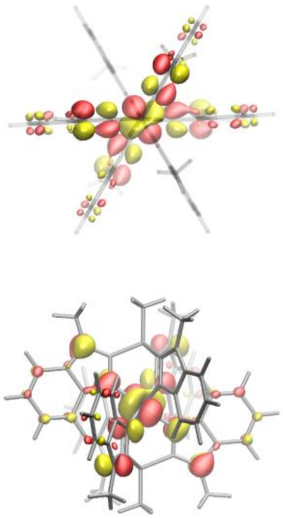	0.83	$\pi_{(HOMO)_6} \rightarrow d_{1e}$
		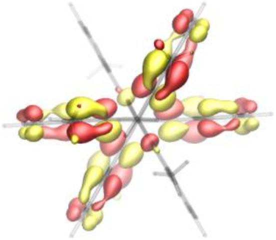	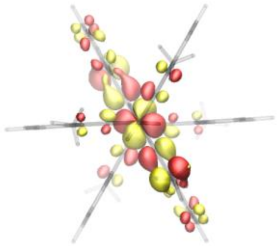	0.17	$\pi_{(HOMO)_6} \rightarrow d_{1e}$

		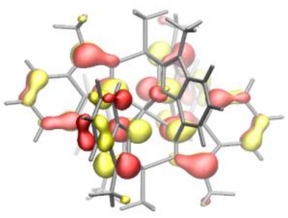	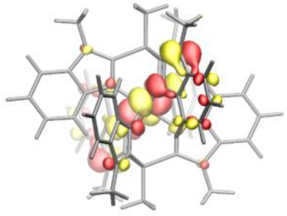		
4: 7885.3	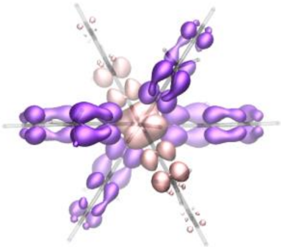 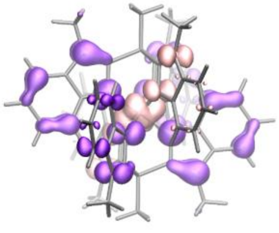	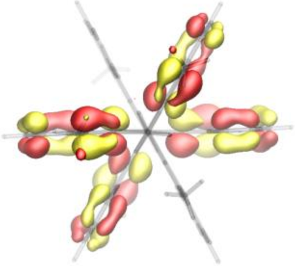 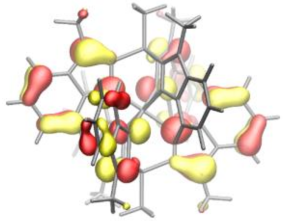	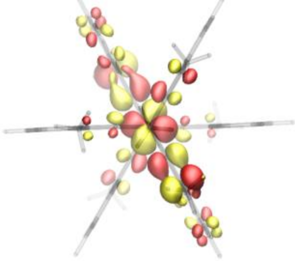 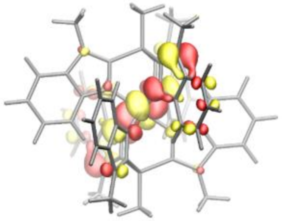	0.83	$\pi_{(HOMO)_6} \rightarrow d_{1e}$

		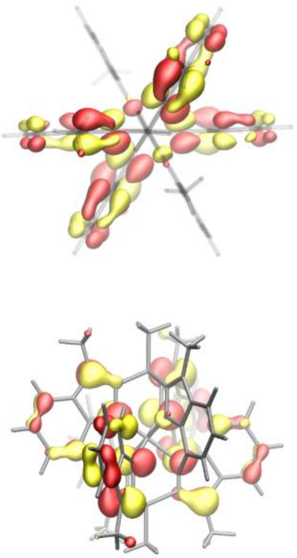	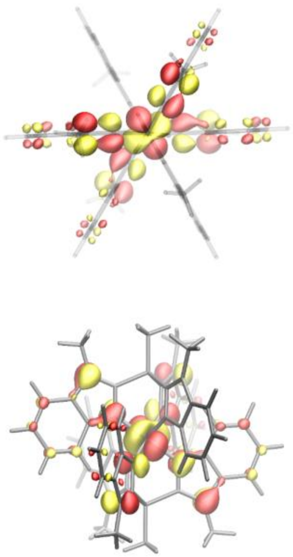	0.17	$\pi_{(HOMO)_6} \rightarrow d_{1e}$
6: 9984.5	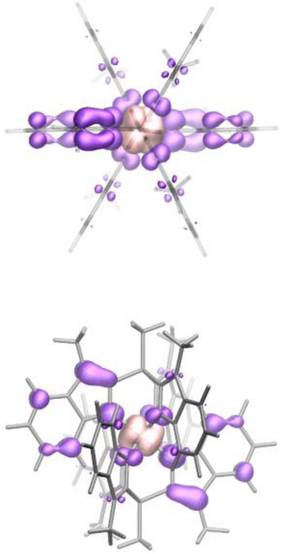	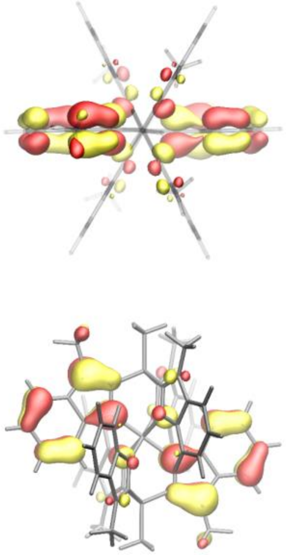	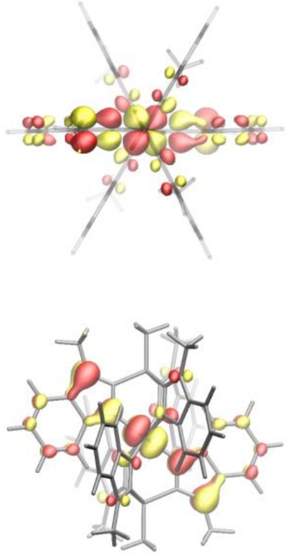	0.67	$\pi_{(HOMO)_6} \rightarrow d_{1e}$

		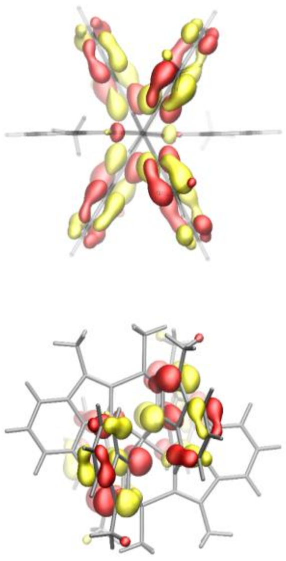	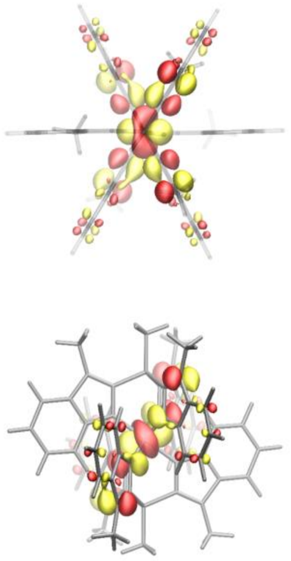	0.31	$\pi_{(HOMO)_6} \rightarrow d_{1e}$
7: 10027.0	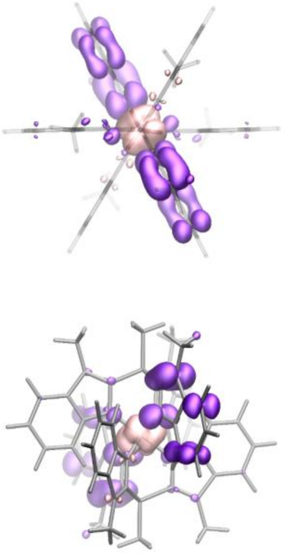	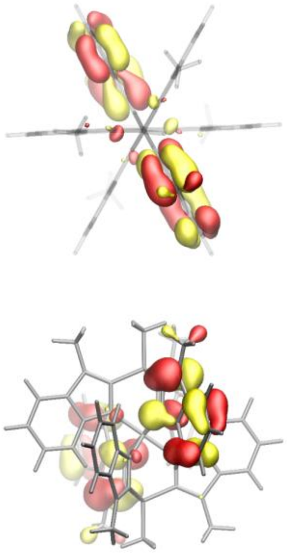	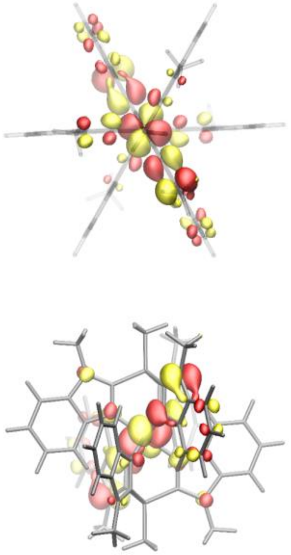	0.71	$\pi_{(HOMO)_6} \rightarrow d_{1e}$

		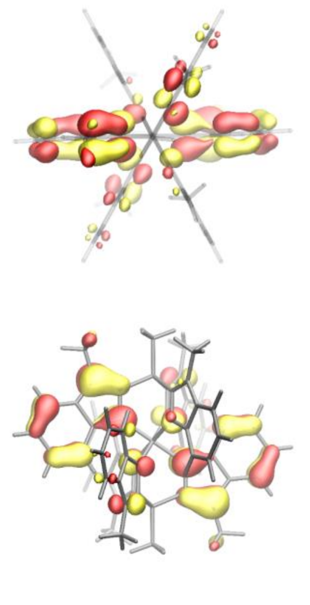	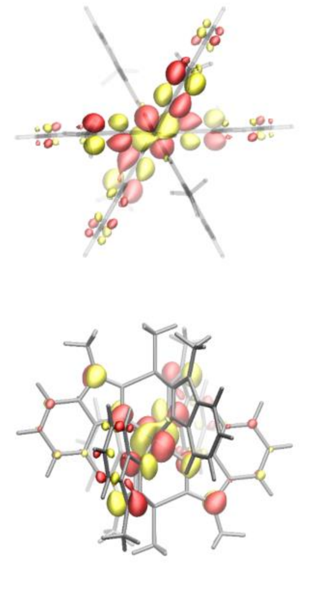	0.27	$\pi_{(HOMO)_6} \rightarrow d_{1e}$
8: 10154.3	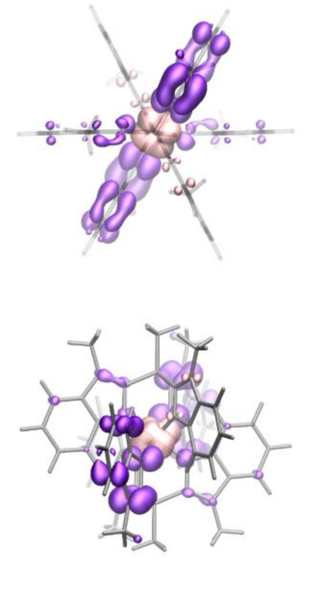	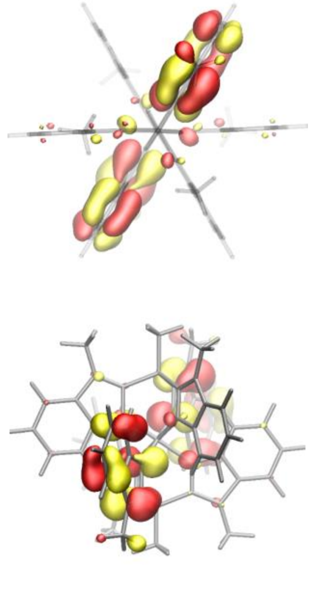	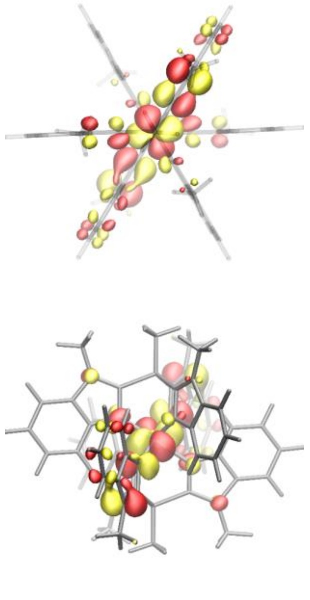	0.75	$\pi_{(HOMO)_6} \rightarrow d_{1e}$

		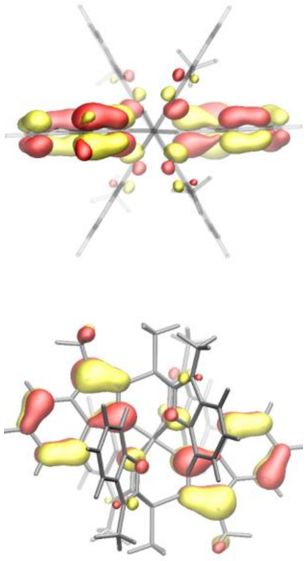	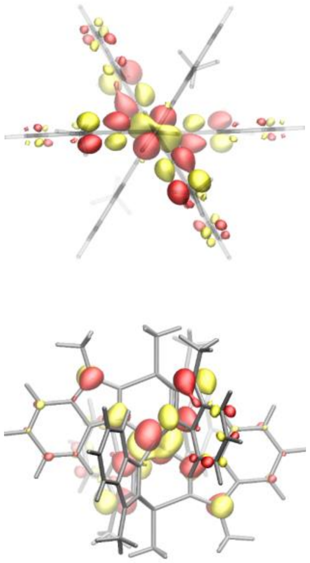	0.22	$\pi_{(HOMO)_6} \rightarrow d_{1e}$
15: 12226.7	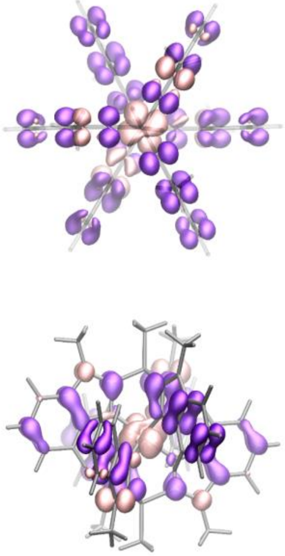	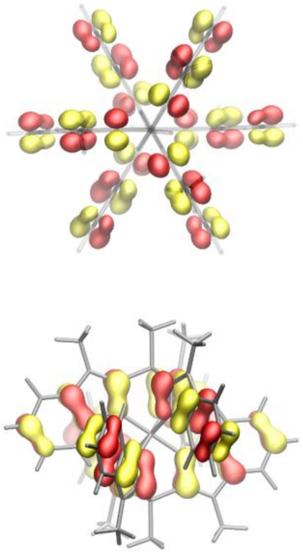	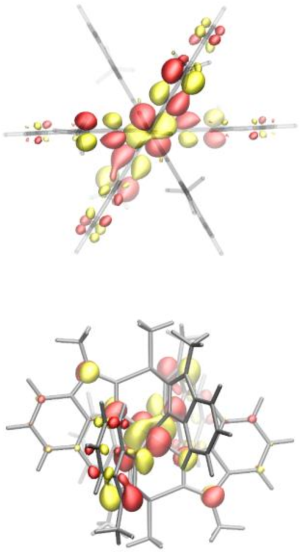	0.99	$\pi_{(HOMO-1)_6} \rightarrow d_{1e}$

16: 12259.3	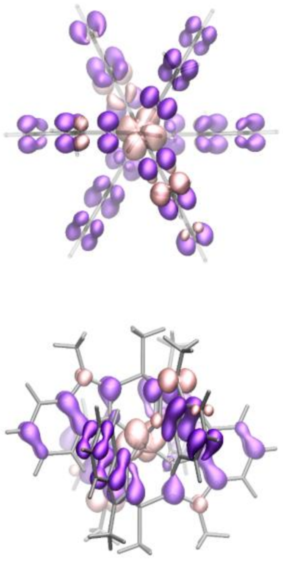	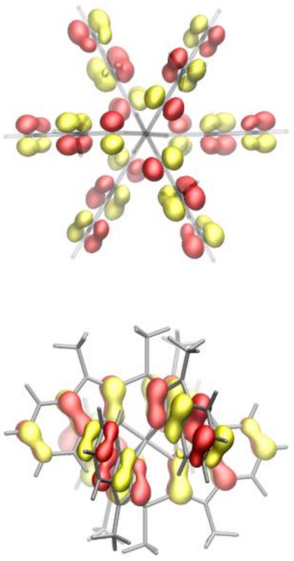	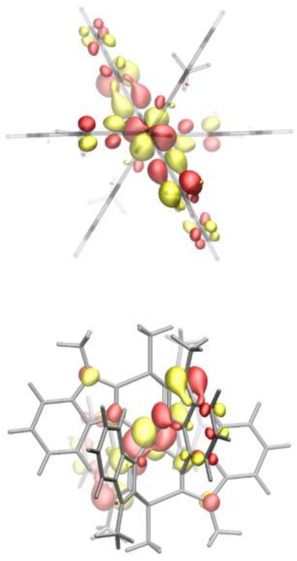	0.99	$\pi_{(HOMO-1)_6} \rightarrow d_{1e}$
18: 15060.7	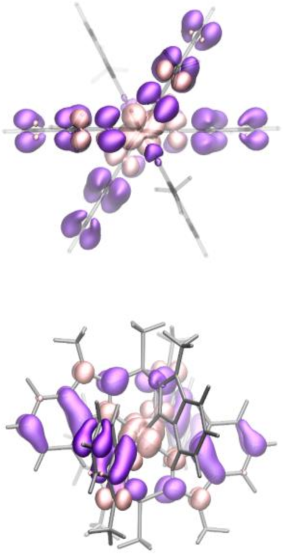	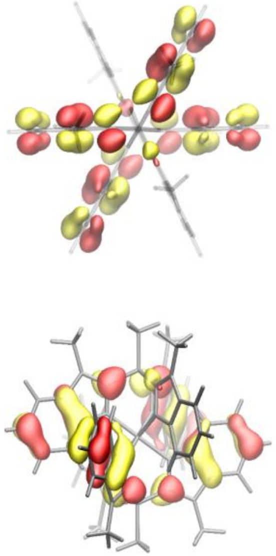	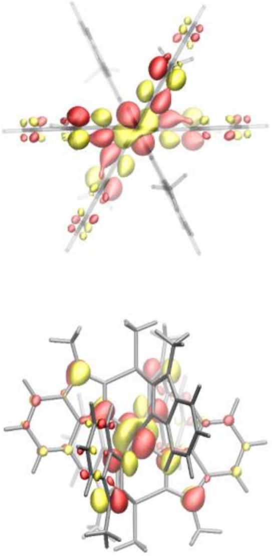	0.95	$\pi_{(HOMO-1)_6} \rightarrow d_{1e}$

		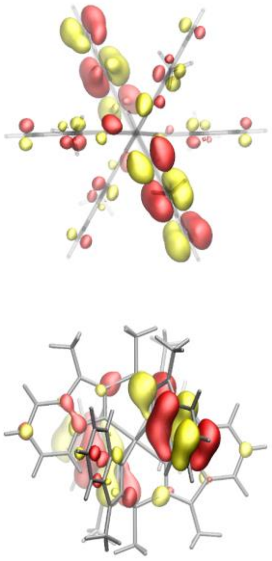	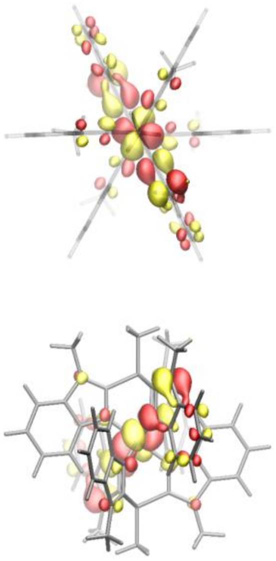	0.05	$\pi_{(HOMO-1)_6} \rightarrow d_{1e}$
19: 15048.8	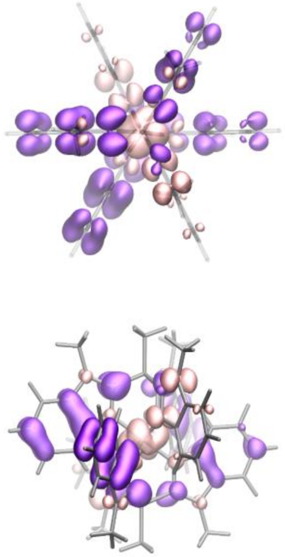	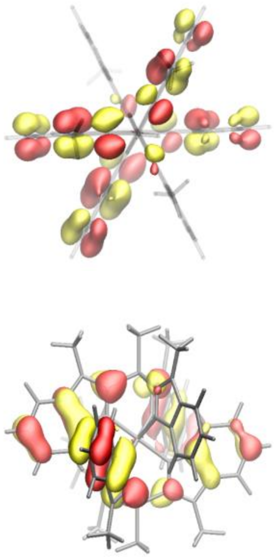	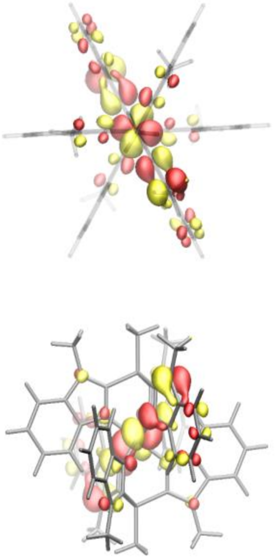	0.87	$\pi_{(HOMO-1)_6} \rightarrow d_{1e}$

		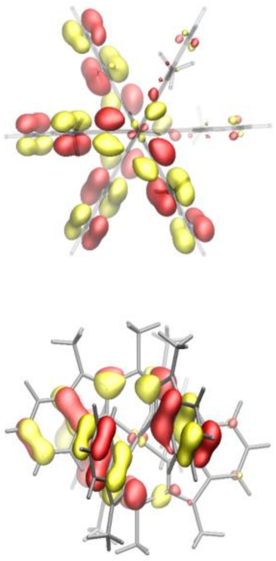	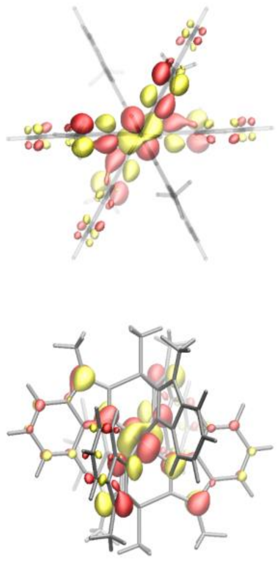	0.13	$\pi_{(HOMO-1)_6} \rightarrow d_{1e}$
22: 15293.9	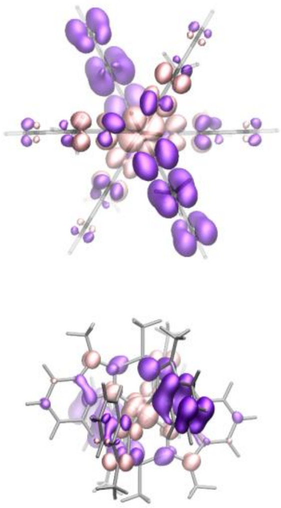	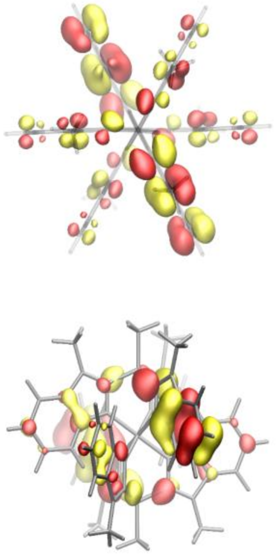	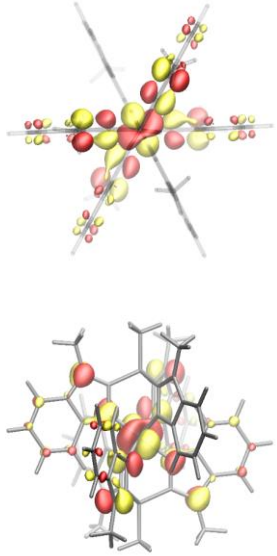	0.91	$\pi_{(HOMO-1)_6} \rightarrow d_{1e}$

		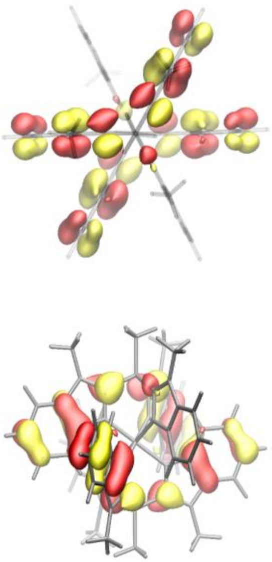	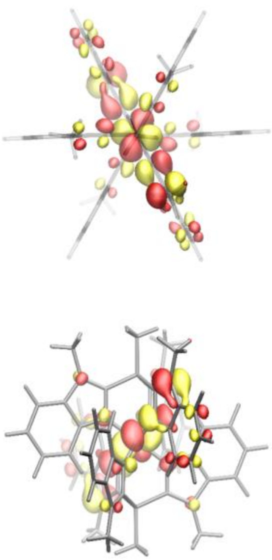	0.09	$\pi_{(HOMO-1)_6} \rightarrow d_{1e}$
40: 22662.8	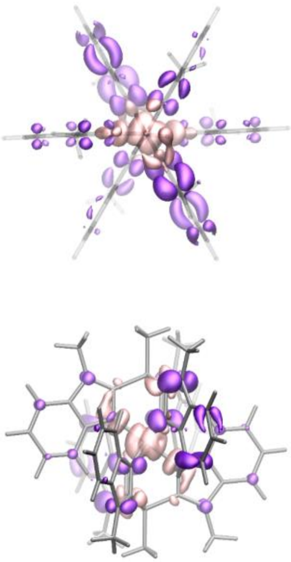	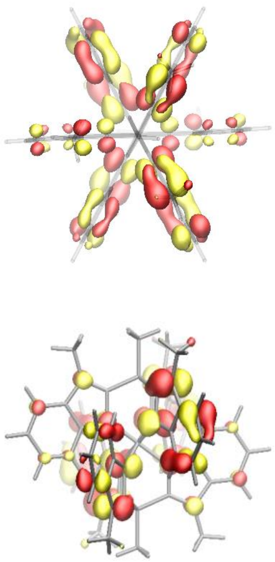	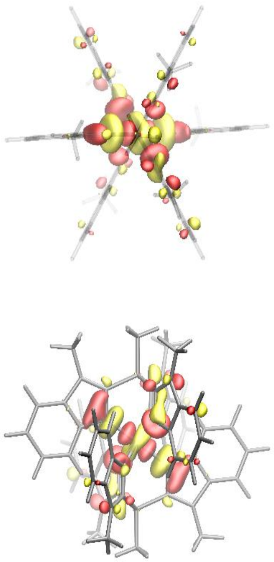	0.43	$\pi_{(HOMO)_6} \rightarrow d_{2e}$

		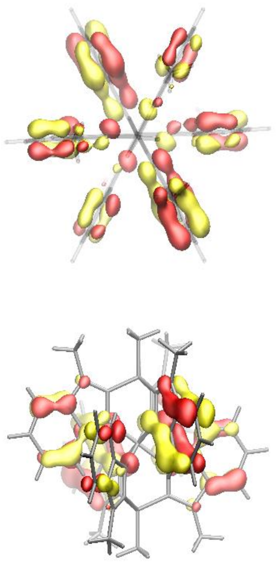	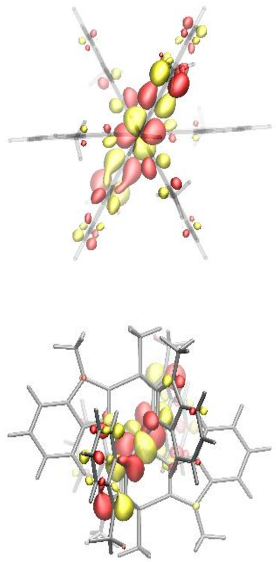	0.16	$\pi_{(HOMO)_6} \rightarrow d_{1e}$
		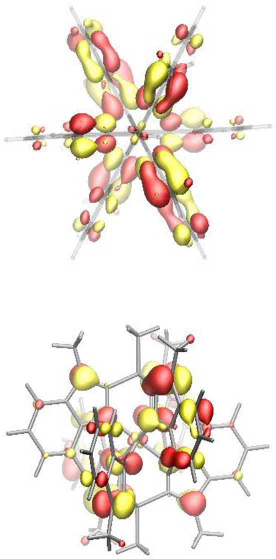	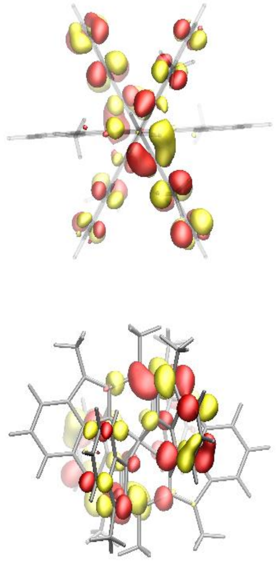	0.13	$\pi_{(HOMO)_6} \rightarrow \pi^*_{(LUMO)_6}$

		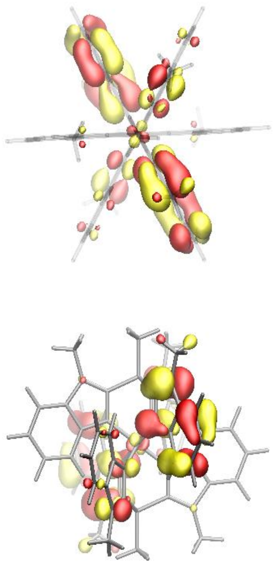	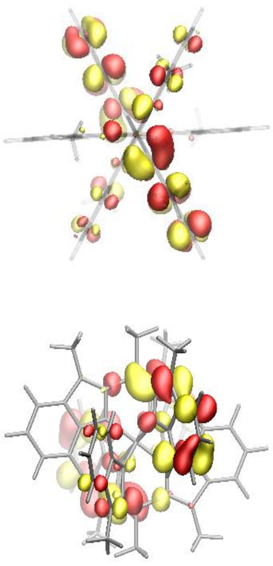	0.09	$\pi_{(HOMO)_6} \rightarrow \pi^*_{(LUMO)_6}$
		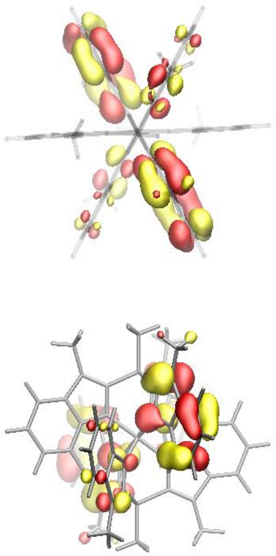	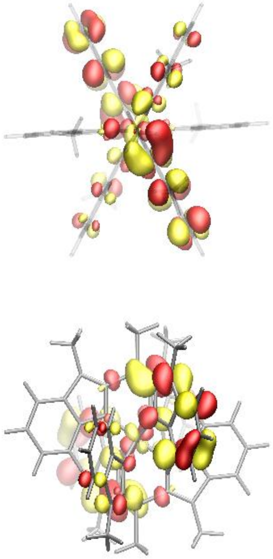	0.08	$\pi_{(HOMO)_6} \rightarrow \pi^*_{(LUMO)_6}$

44: 23010.4	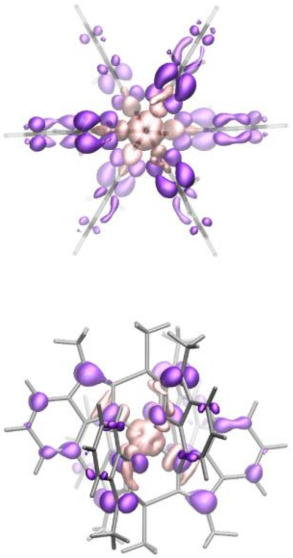	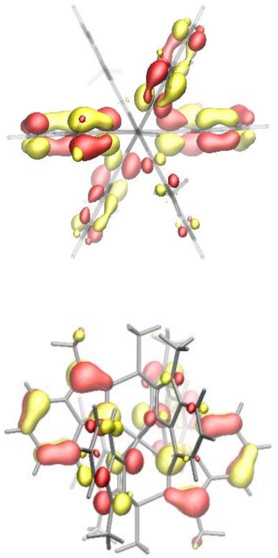	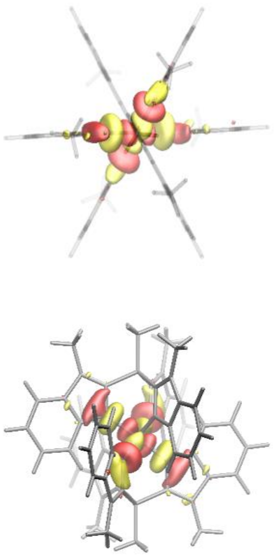	0.44	$\pi_{(HOMO)_6} \rightarrow d_{2e}$
		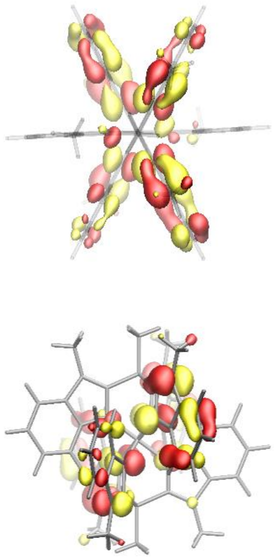	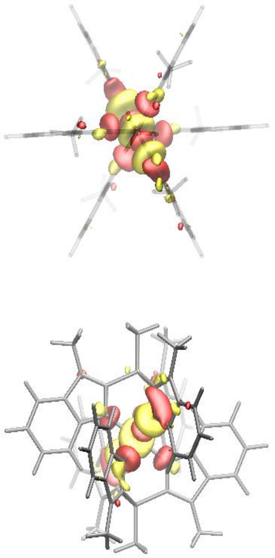	0.28	$\pi_{(HOMO)_6} \rightarrow d_{2e}$

		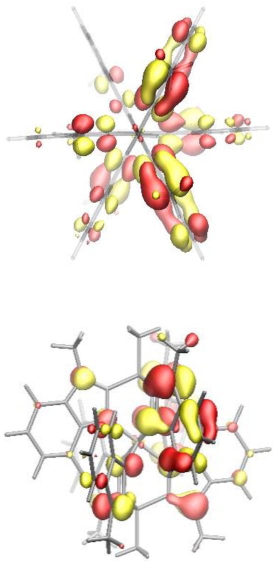	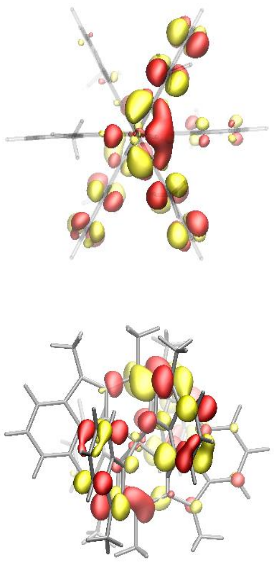	0.09	$\pi_{(HOMO)_6} \rightarrow \pi^*_{(LUMO)_6}$
48: 23787.7	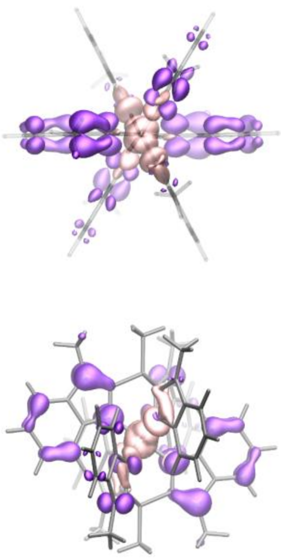	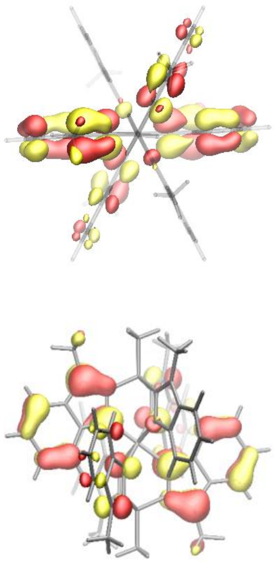	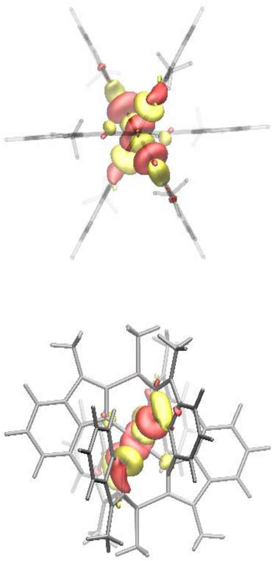	0.62	$\pi_{(HOMO)_6} \rightarrow d_{2e}$

		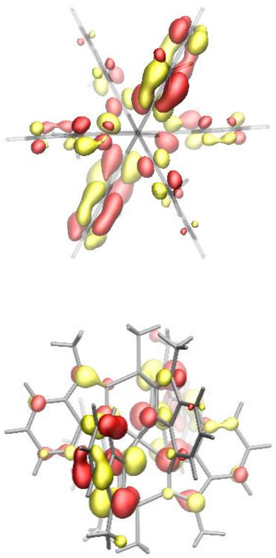	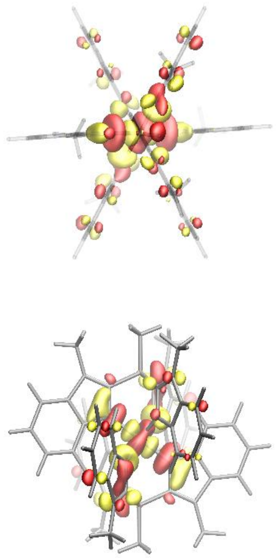	0.12	$\pi_{(HOMO)_6} \rightarrow d_{2e}$
		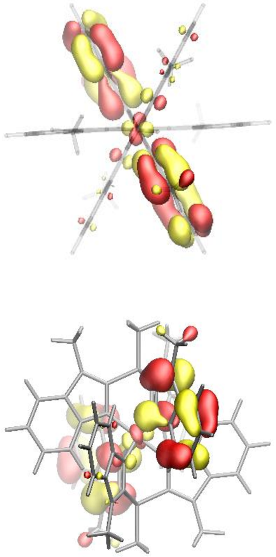	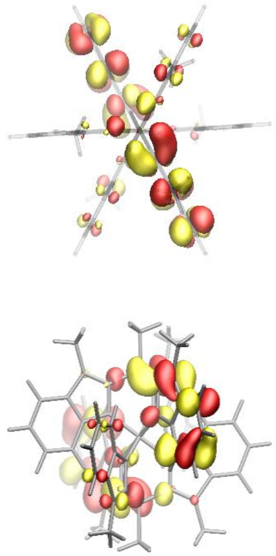	0.06	$\pi_{(HOMO)_6} \rightarrow \pi^*_{(LUMO)_6}$

S15 Spectra of isolated compounds

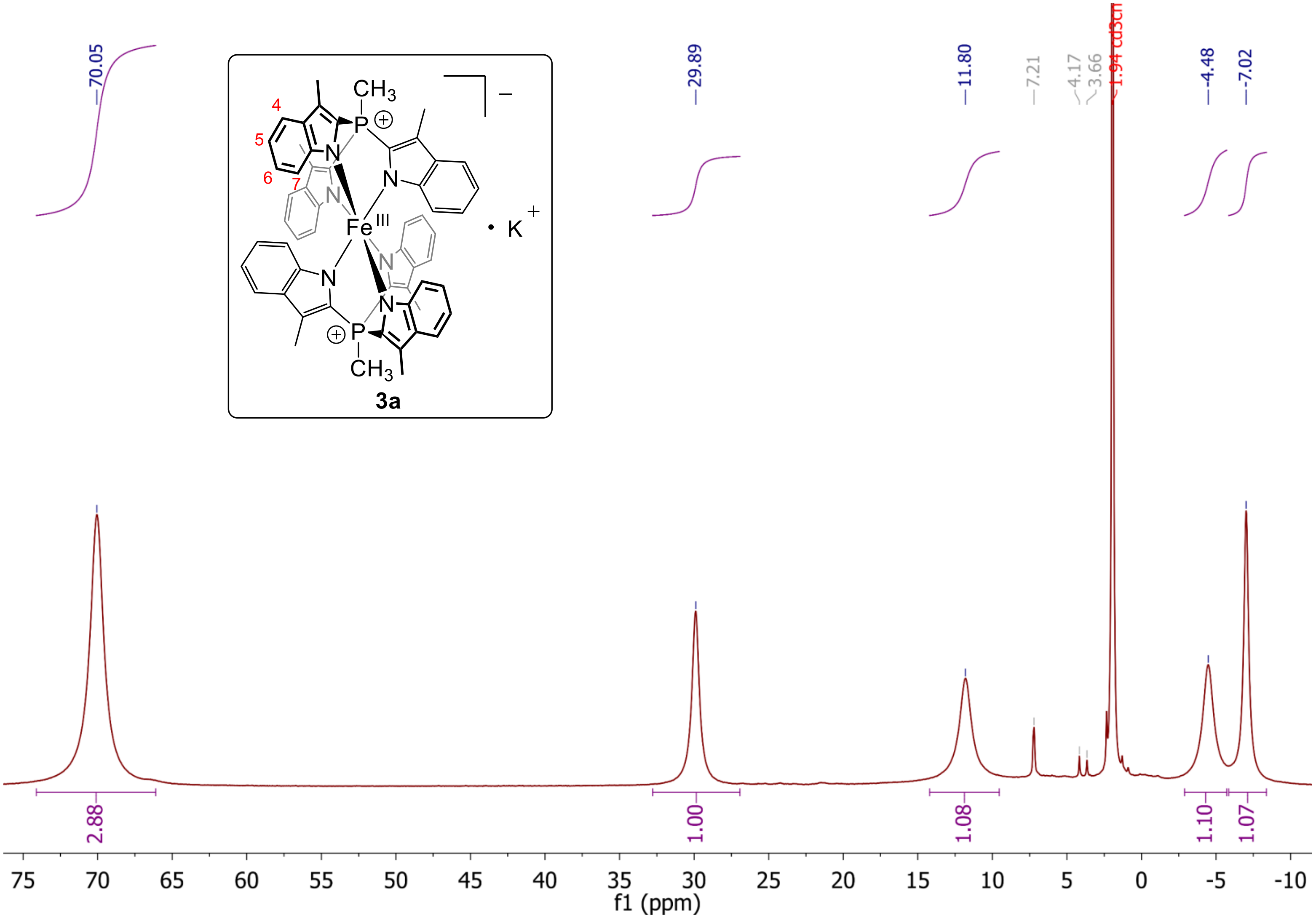

Figure S40. ^{1}H NMR (400 MHz) spectrum of compound **3a** in acetonitrile-d_3. Impurities: 3.66 ppm – THF, 7.21 ppm – toluene.

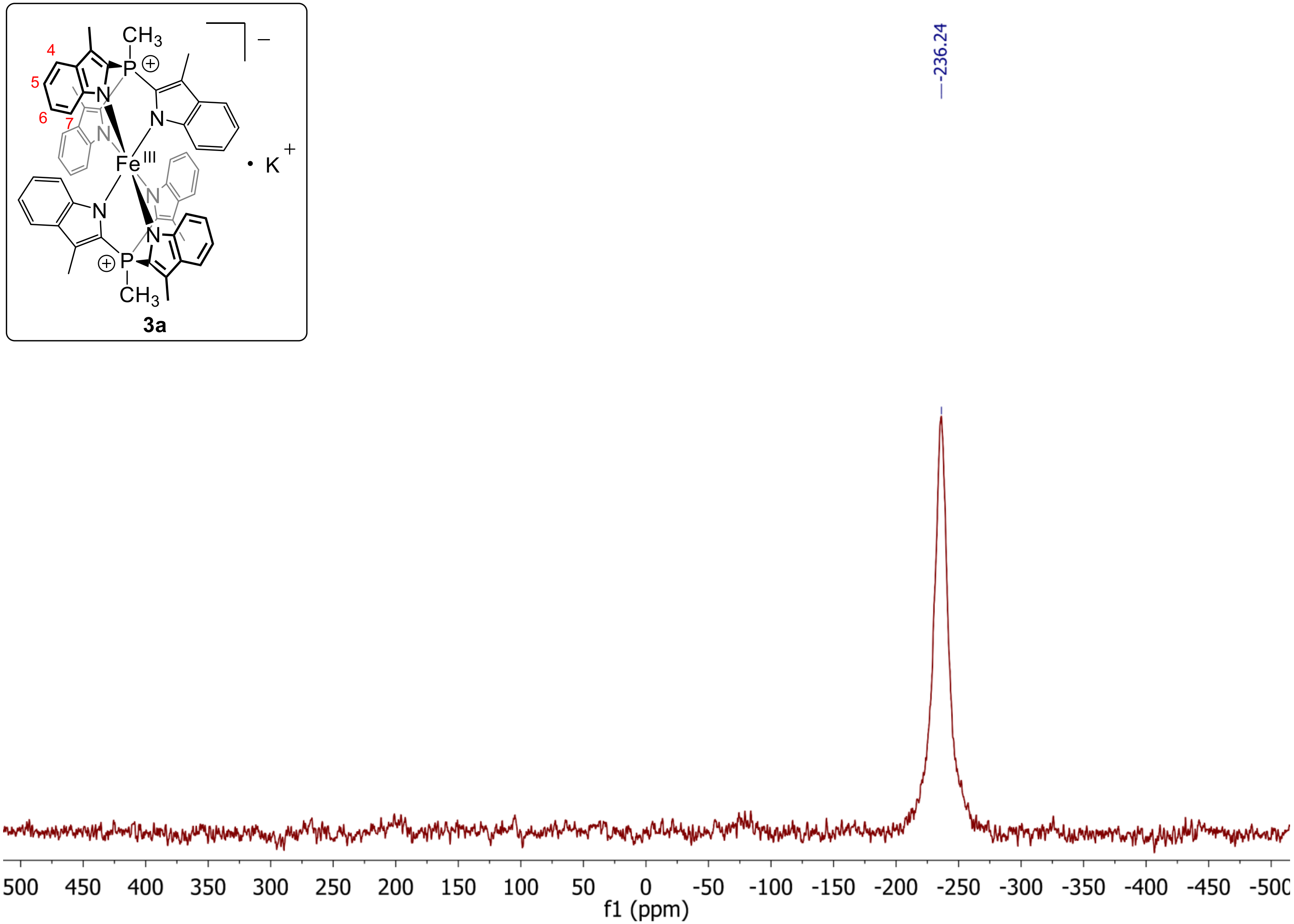

Figure S41. ^{31}P NMR (162 MHz) spectrum of compound **3a** in acetonitrile-d_3.

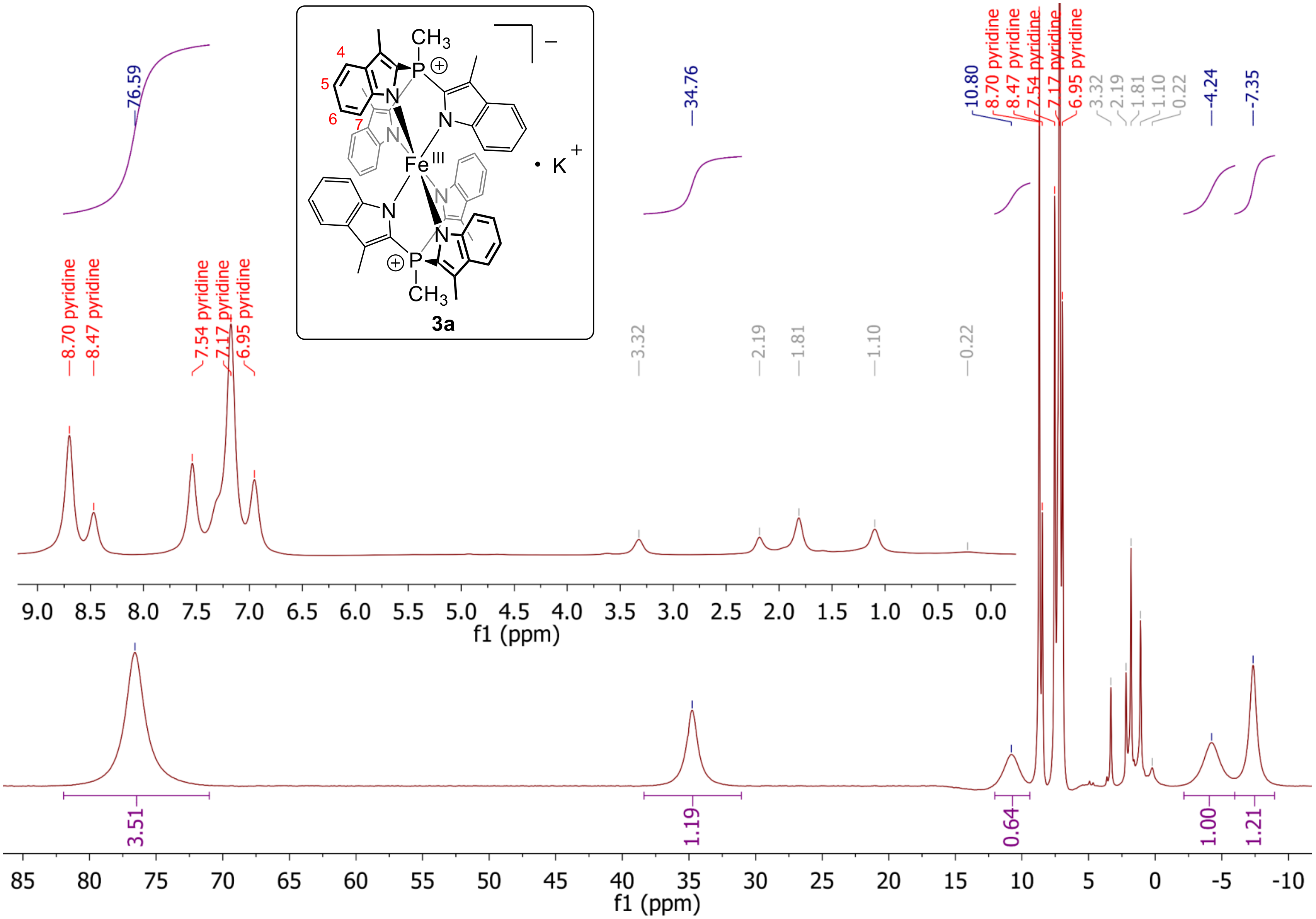

Figure S42. ^{1}H NMR (400 MHz) spectrum of compound **3a** in pyridine-d_5. Impurities: 3.32 and 1.81 ppm – THF, other signals are unidentified.

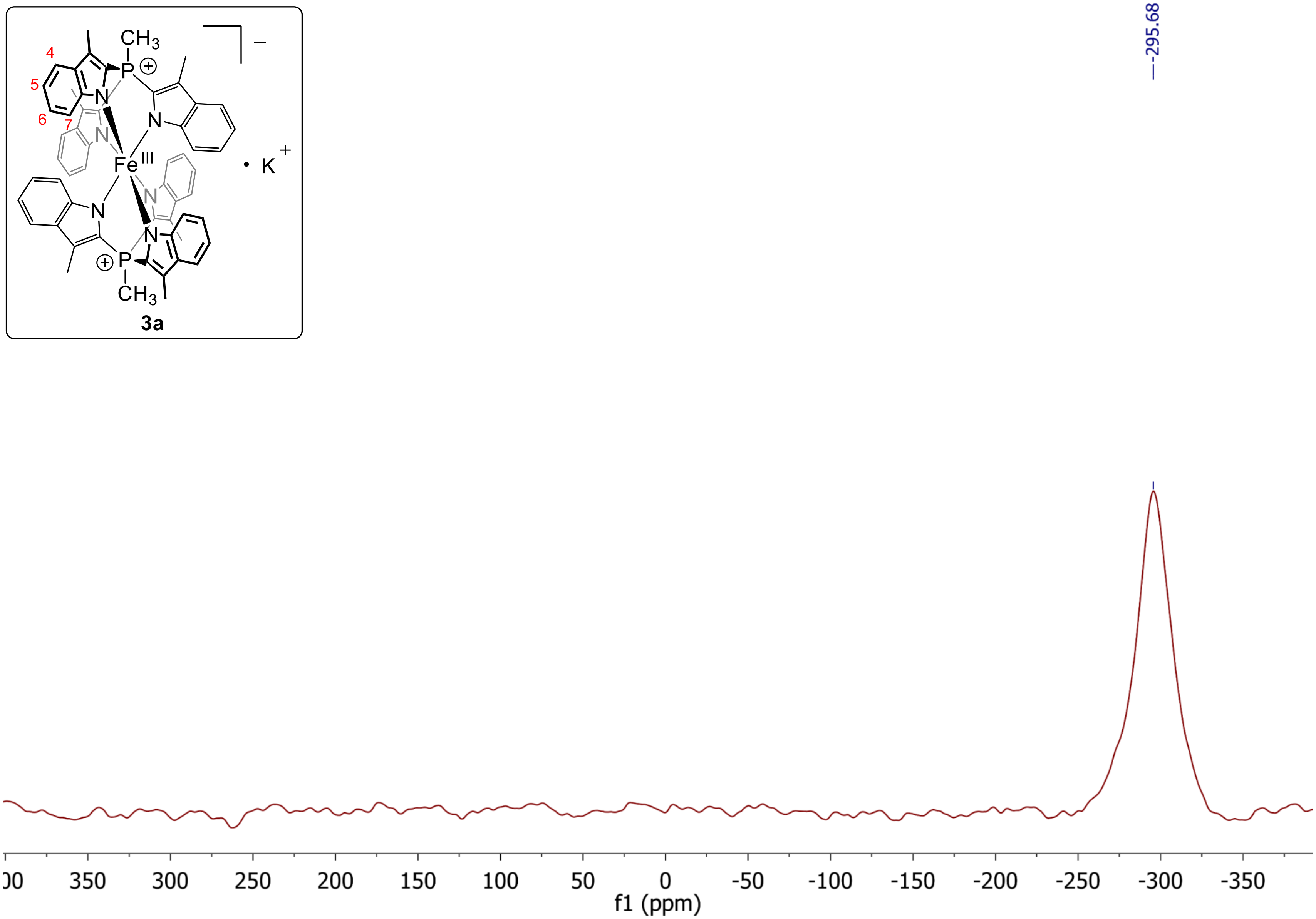

Figure S43. ^{31}P NMR (162 MHz) spectrum of compound **3a** in pyridine-*d*$_5$.

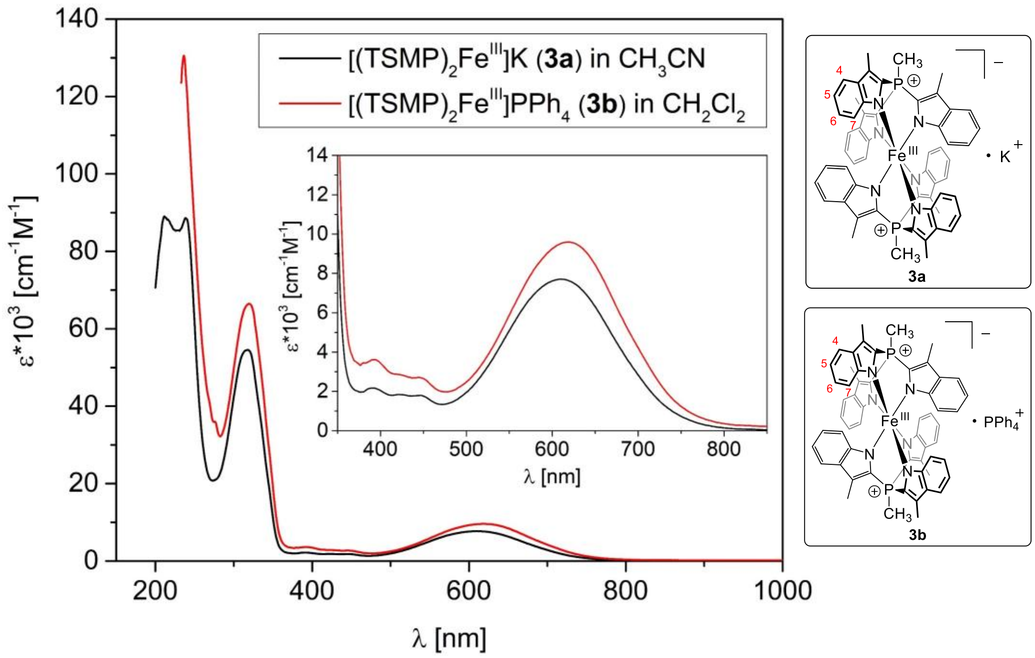

Figure S44. UV-Vis spectra of $[(TSMP)_2Fe^{III}]^-$ (**3**) complex with different counterions in acetonitrile and dichloromethane.

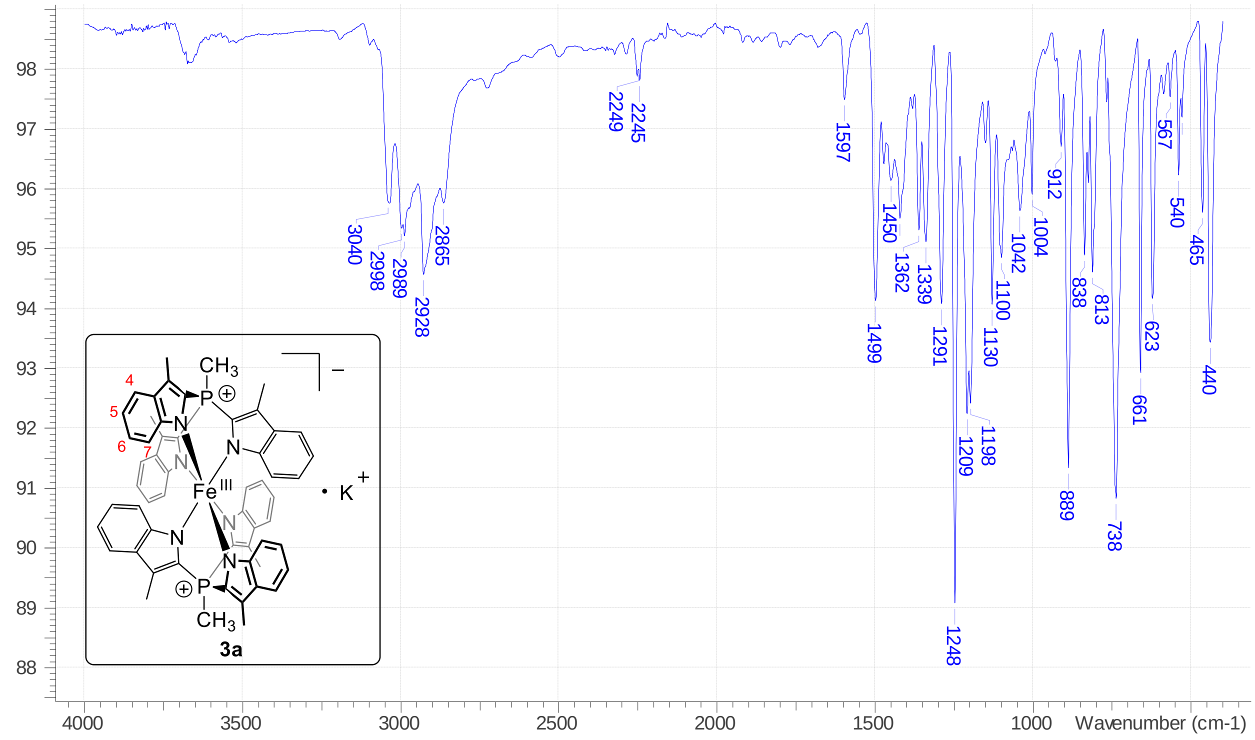

Figure S45. ATR-FTIR (neat) spectrum of compound **3a**.

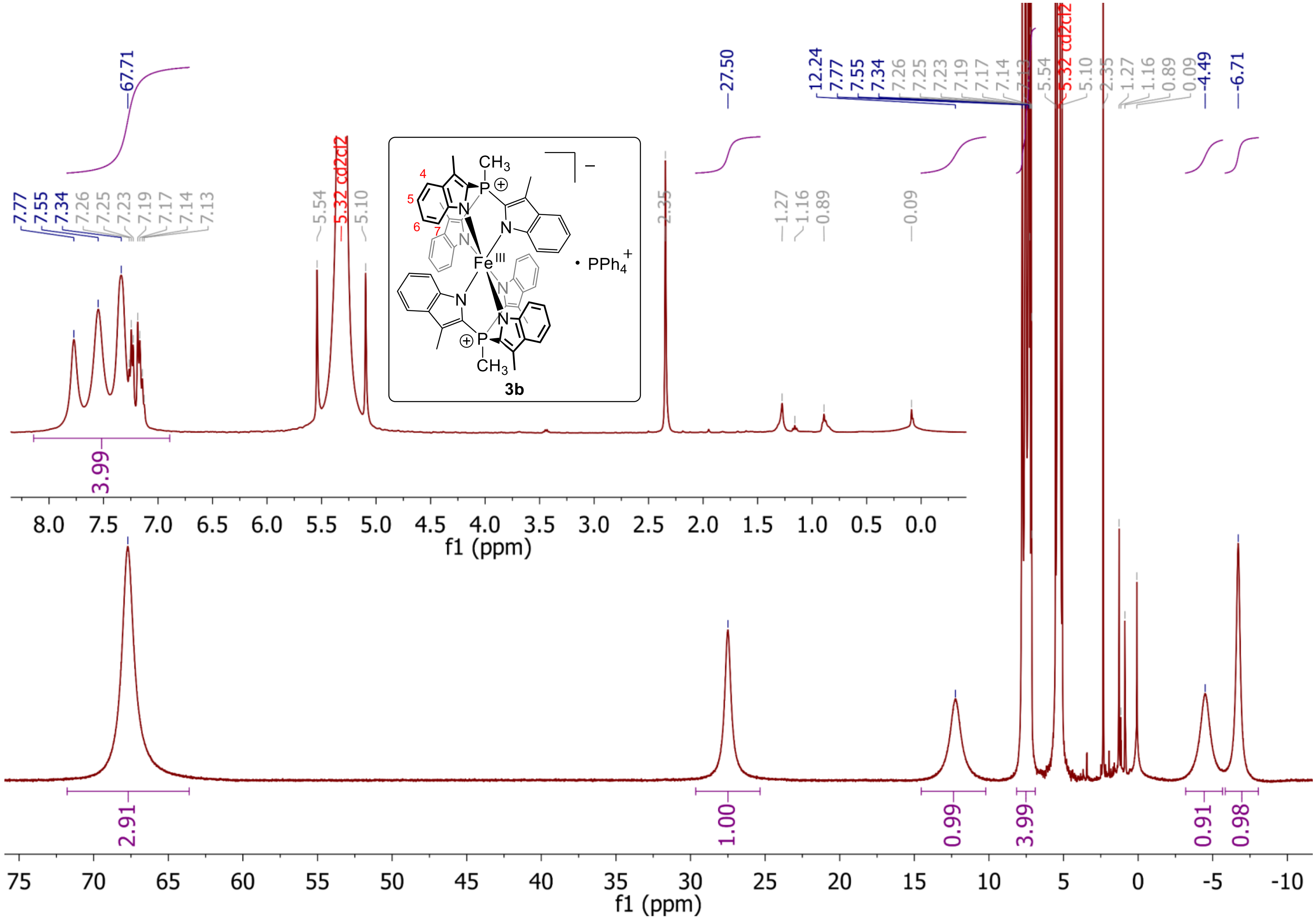

Figure S46. ^{1}H NMR (400 MHz) spectrum of compound **3b** in dichloromethane-d_2. Impurities: 1.27 and 0.89 ppm – *n*-hexane; 0.09 ppm – silicon grease; other signals are unidentified.

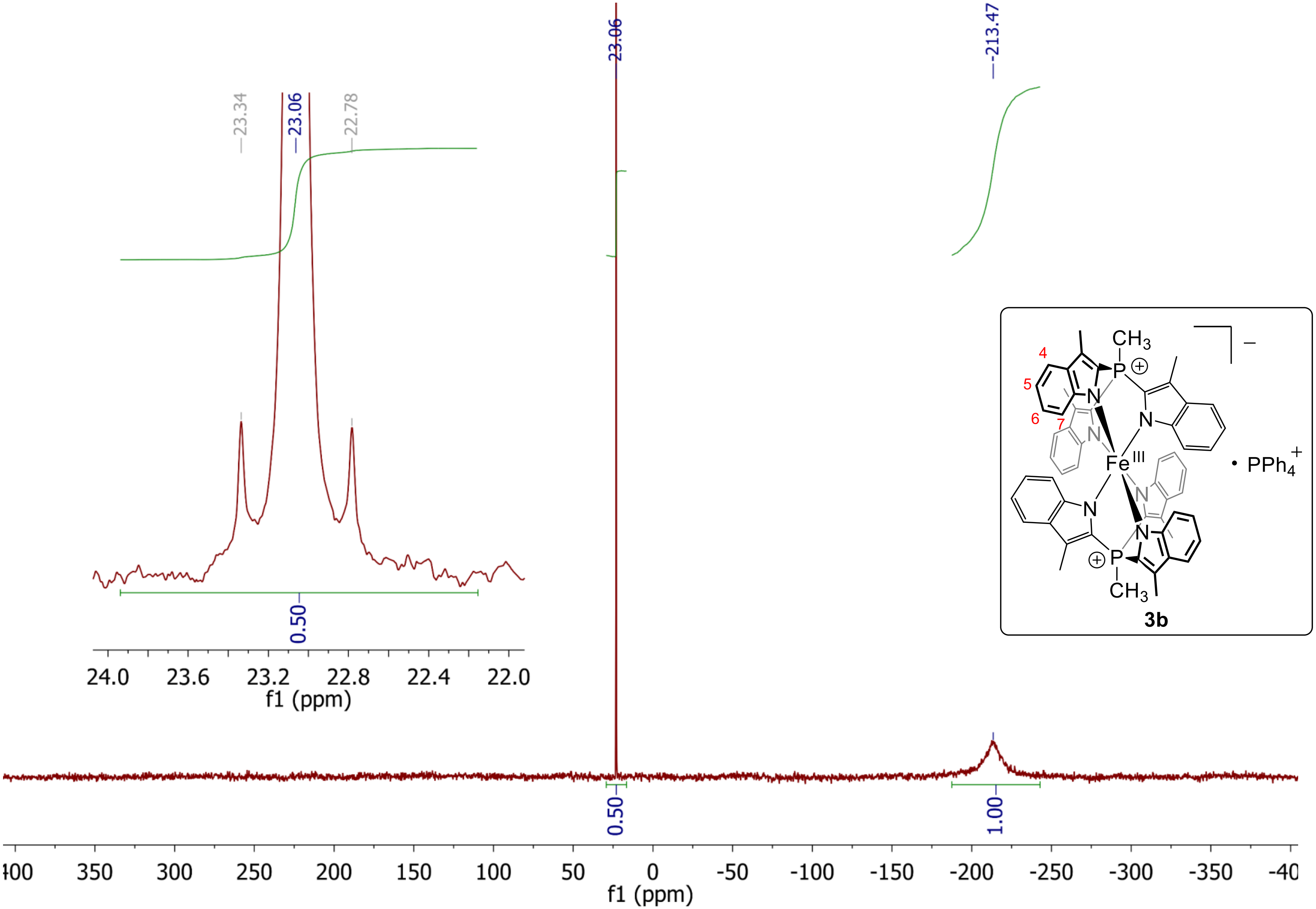

Figure S47. ^{31}P NMR (162 MHz) spectrum of compound **3b** in dichloromethane-d_2.

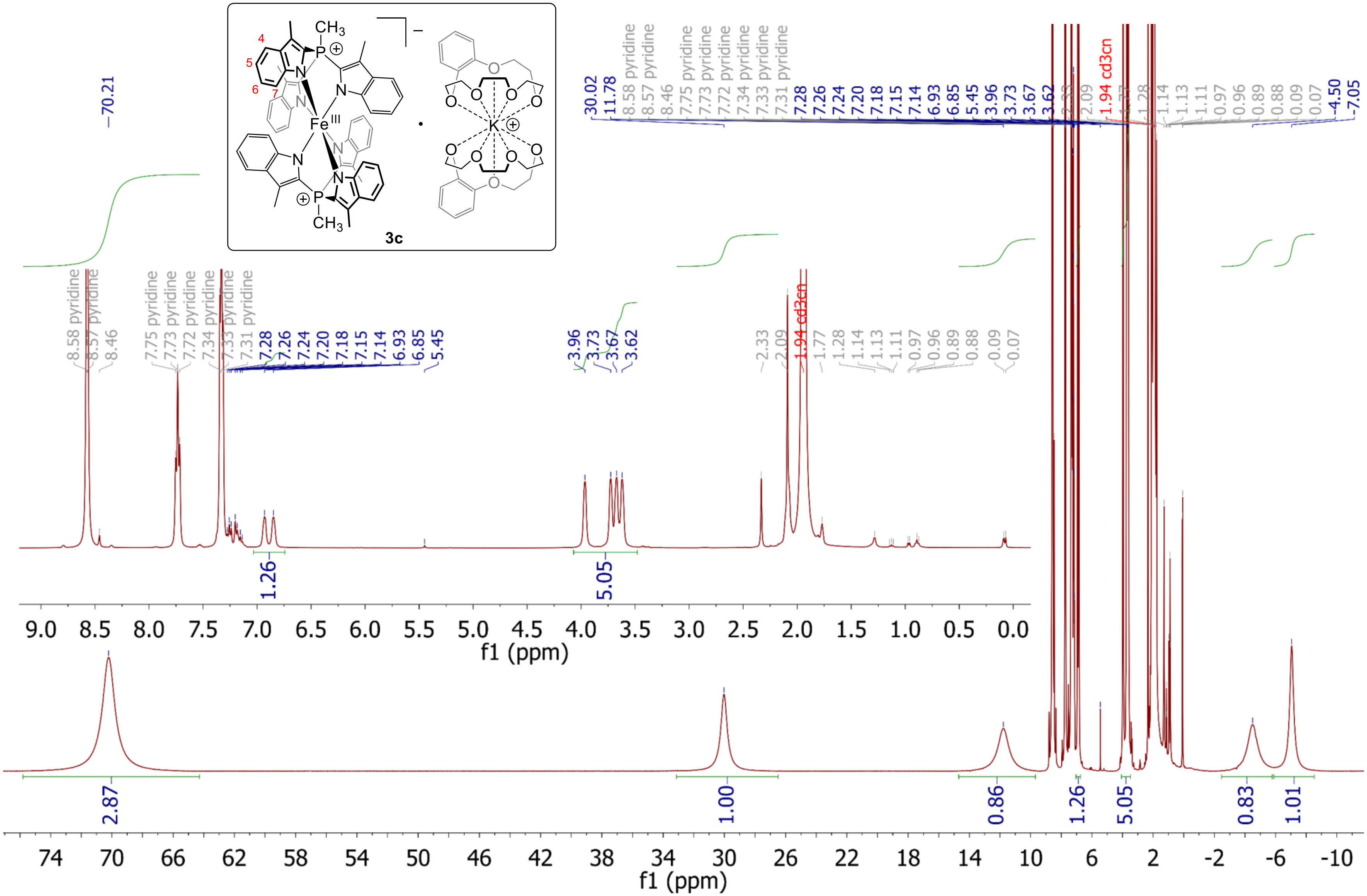

Figure S48. ^{1}H NMR (400 MHz) spectrum of compound **3c** in acetonitrile-d_3. Impurities: 7.28 and 2.33 ppm – toluene, 1.27 and 0.89 ppm – *n*-hexane; 0.09 ppm – silicon grease; other signals are unidentified.

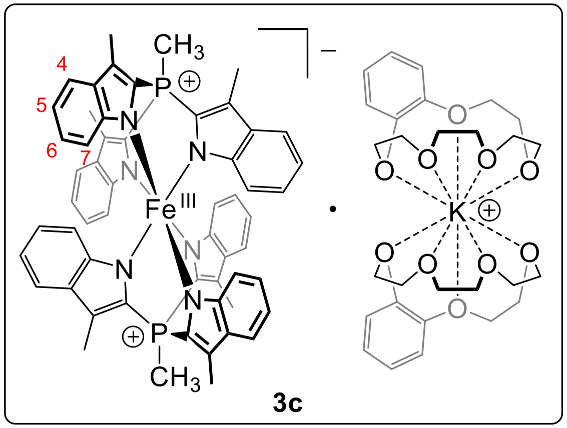

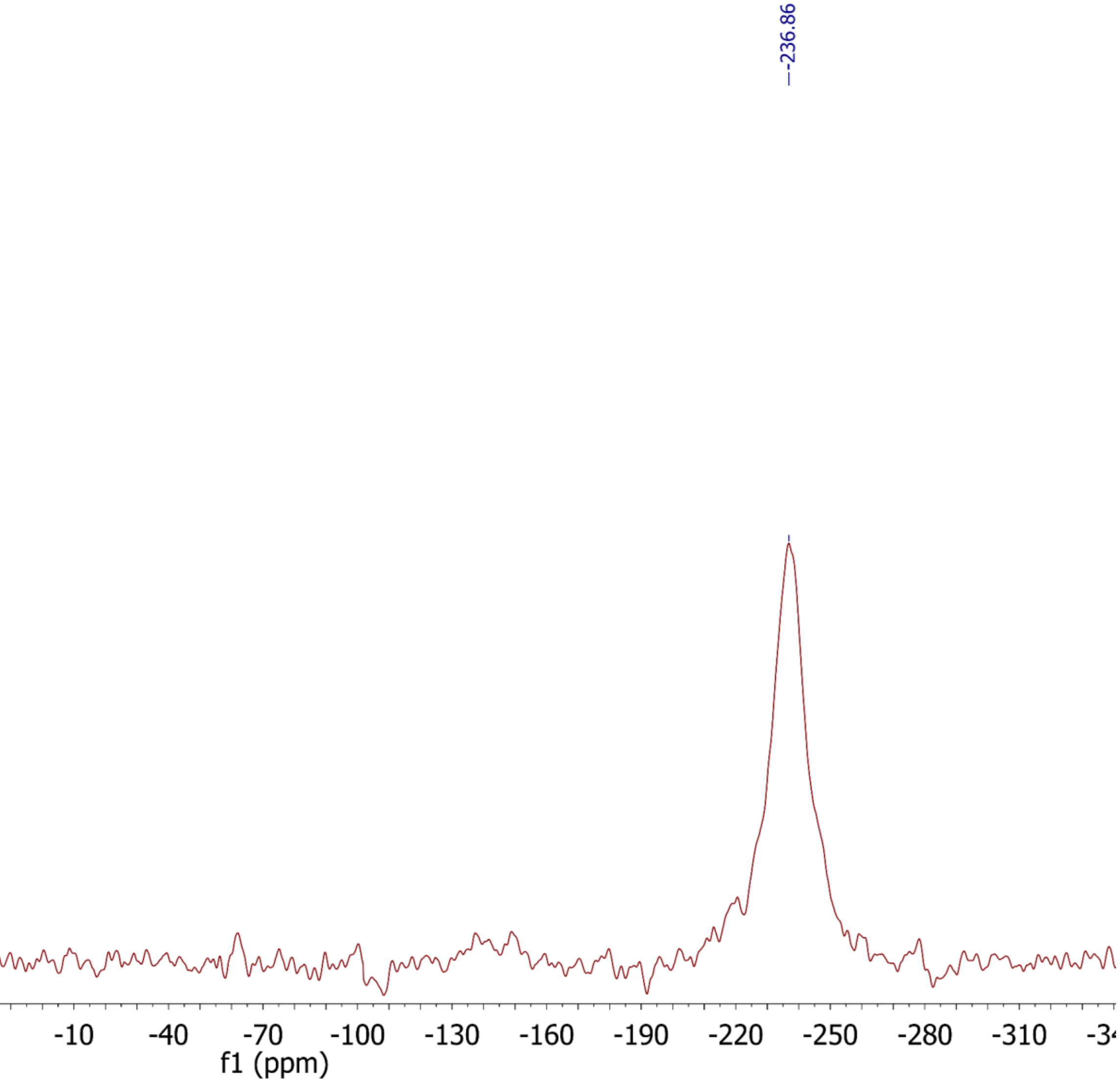

Figure S49. ^{31}P NMR (162 MHz) spectrum of compound **3c** in acetonitrile-d_3.

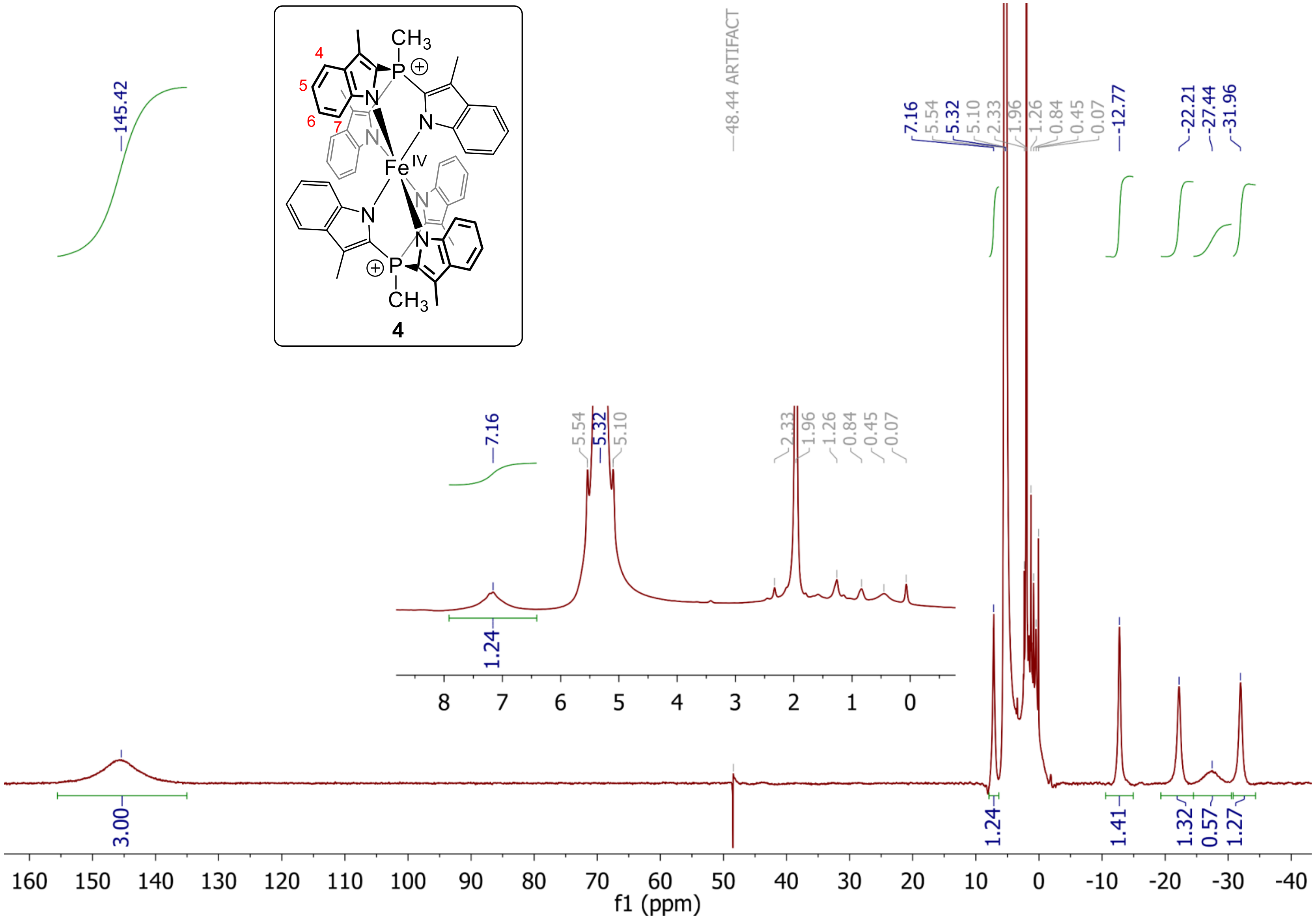

Figure S50. ^{1}H NMR (400 MHz) spectrum of compound **4** in dichloromethane-d_2.

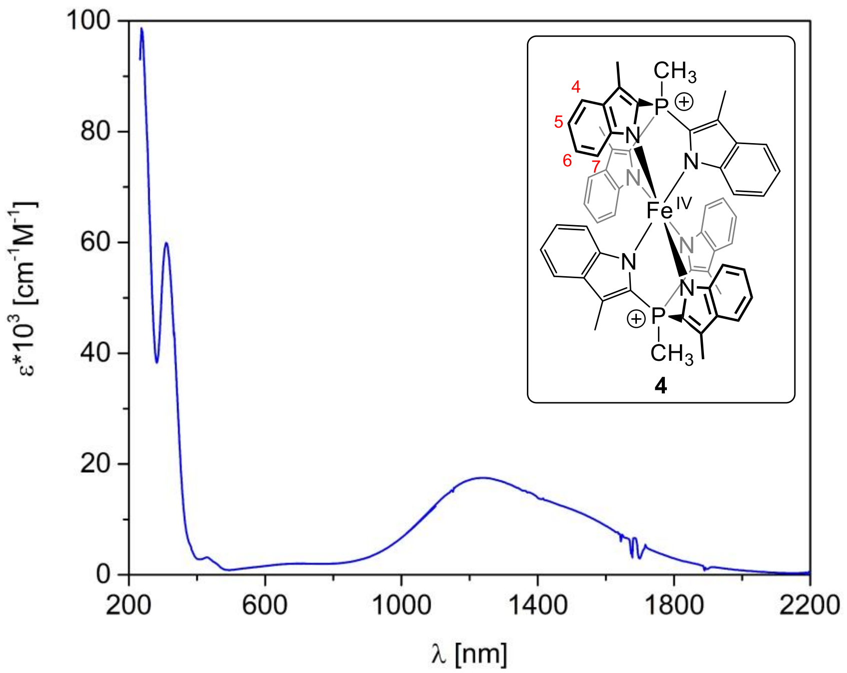

Figure S51. UV-Vis-NIR spectra of [(TSMP)$_2$Fe^{IV}] (**4**) complex in dichloromethane. Small aberrations in absorption between 1600 and 2000 nm are artifacts.

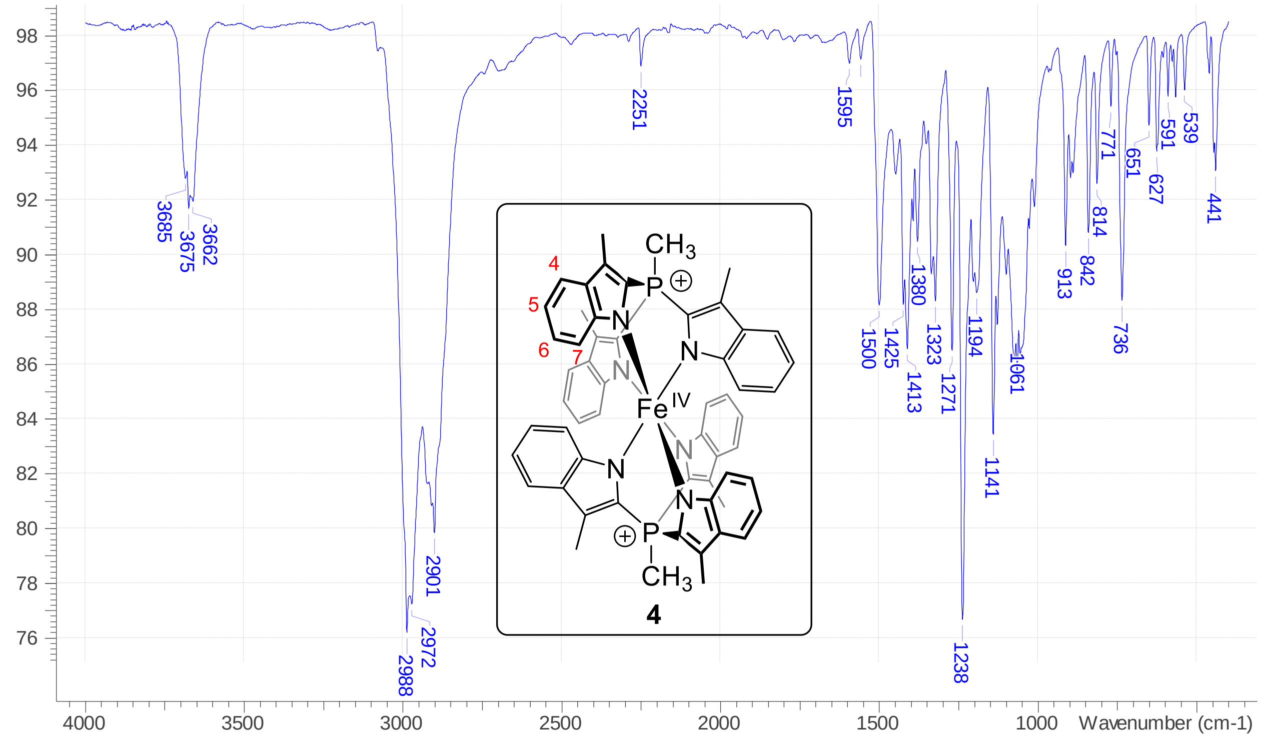

Figure S52. ATR-FTIR (neat) spectrum of compound **4**.

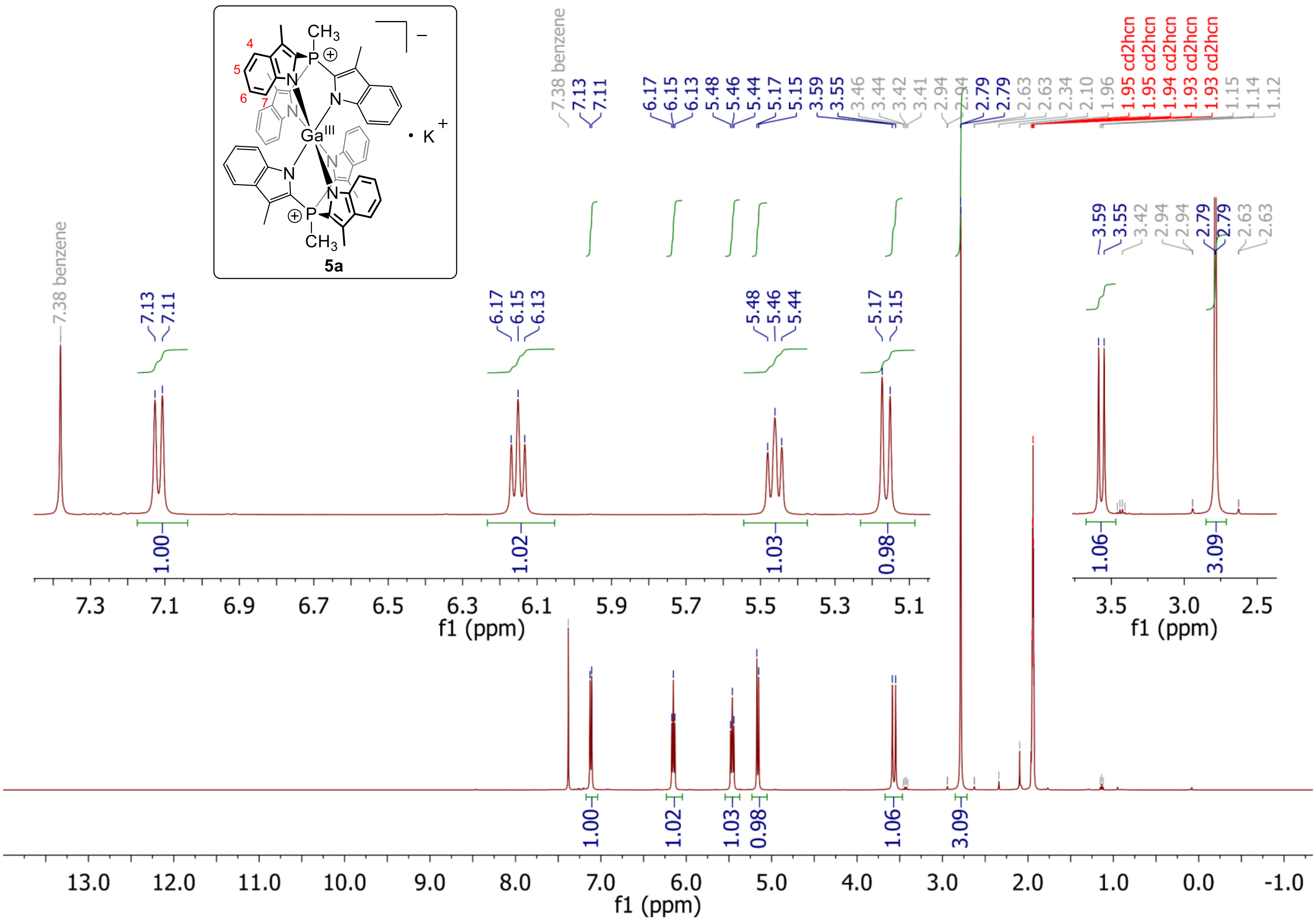

Figure S53. ^{1}H NMR (400 MHz) spectrum of compound **5a** in acetonitrile-d_3.

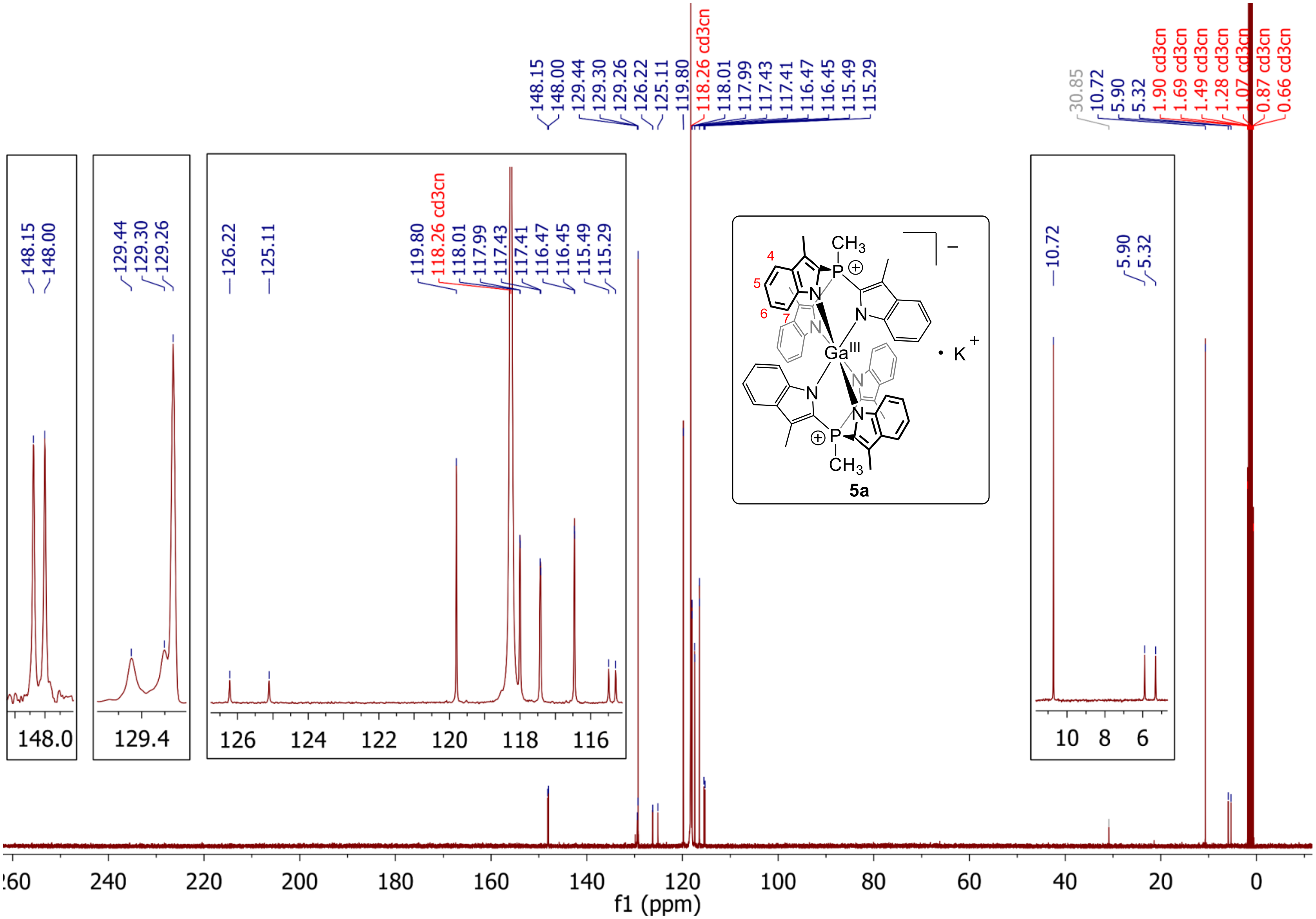

Figure S54. ^{13}C NMR (101 MHz) spectrum of compound **5a** in acetonitrile-d_3.

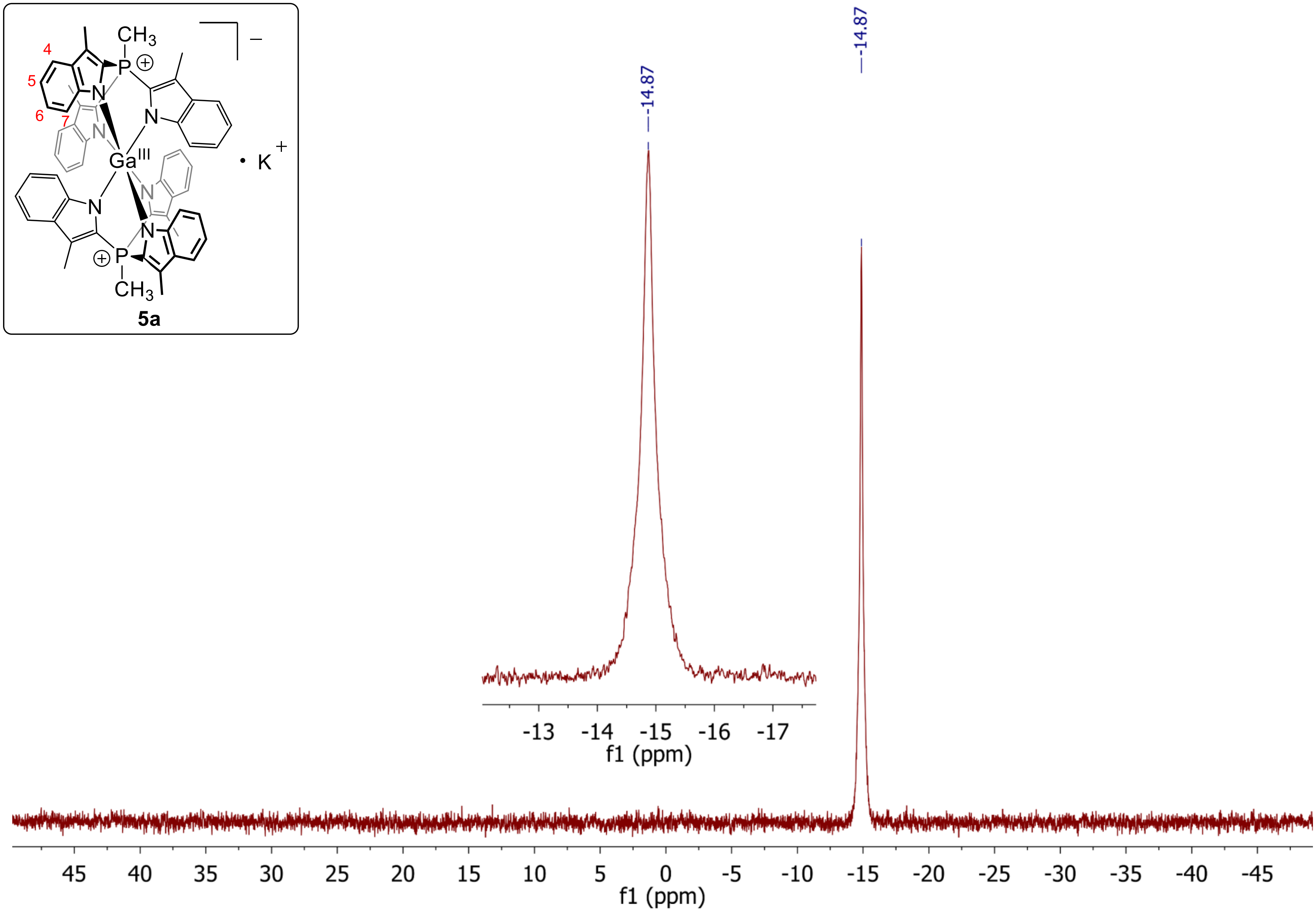

Figure S55. $^{31}P\{^{1}H\}$ NMR (162 MHz) spectrum of compound **5a** in acetonitrile-d_3.

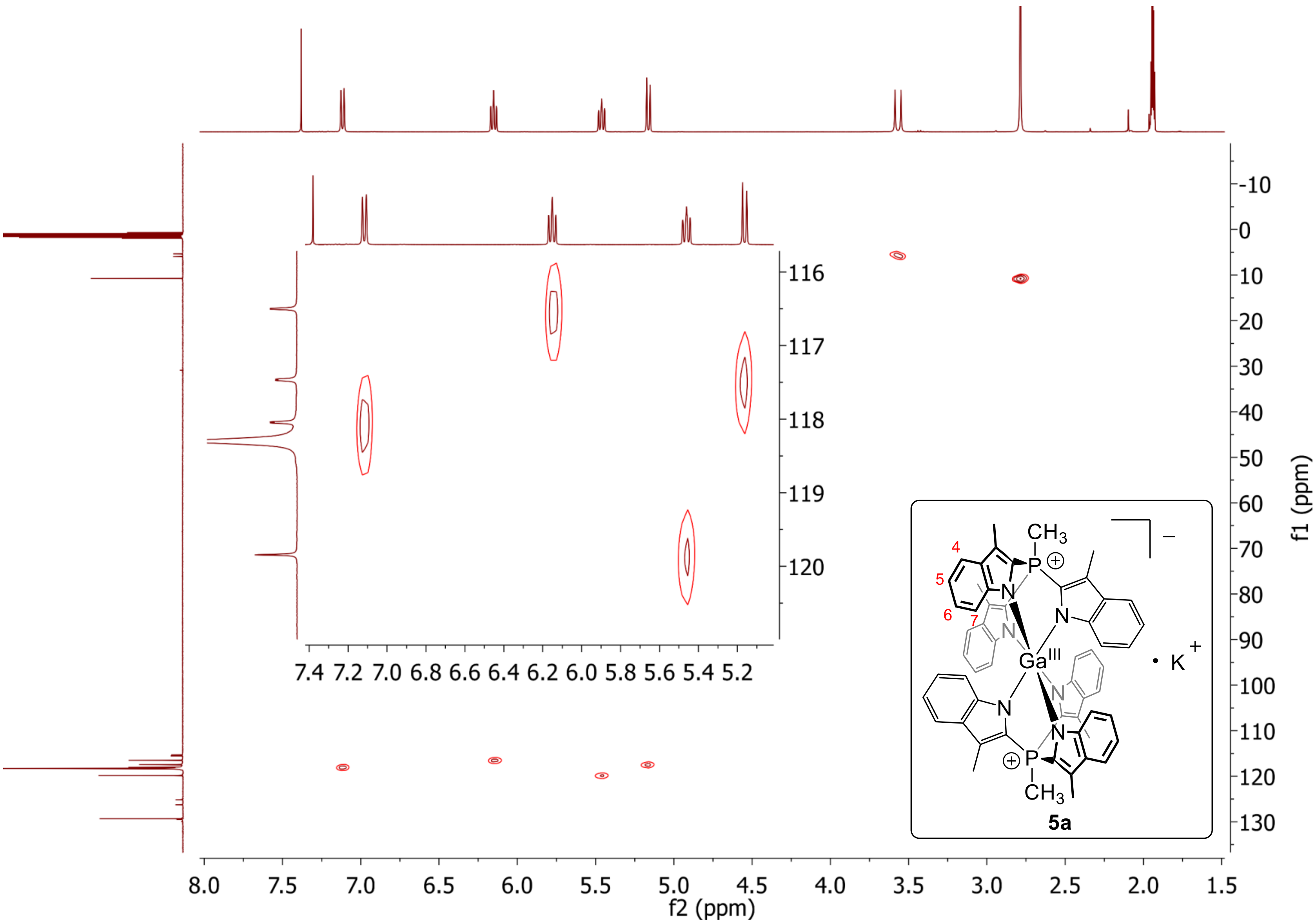

Figure S56. ^{1}H-^{13}C ASAPHMQC spectrum of compound **5a** in acetonitrile-d_3.

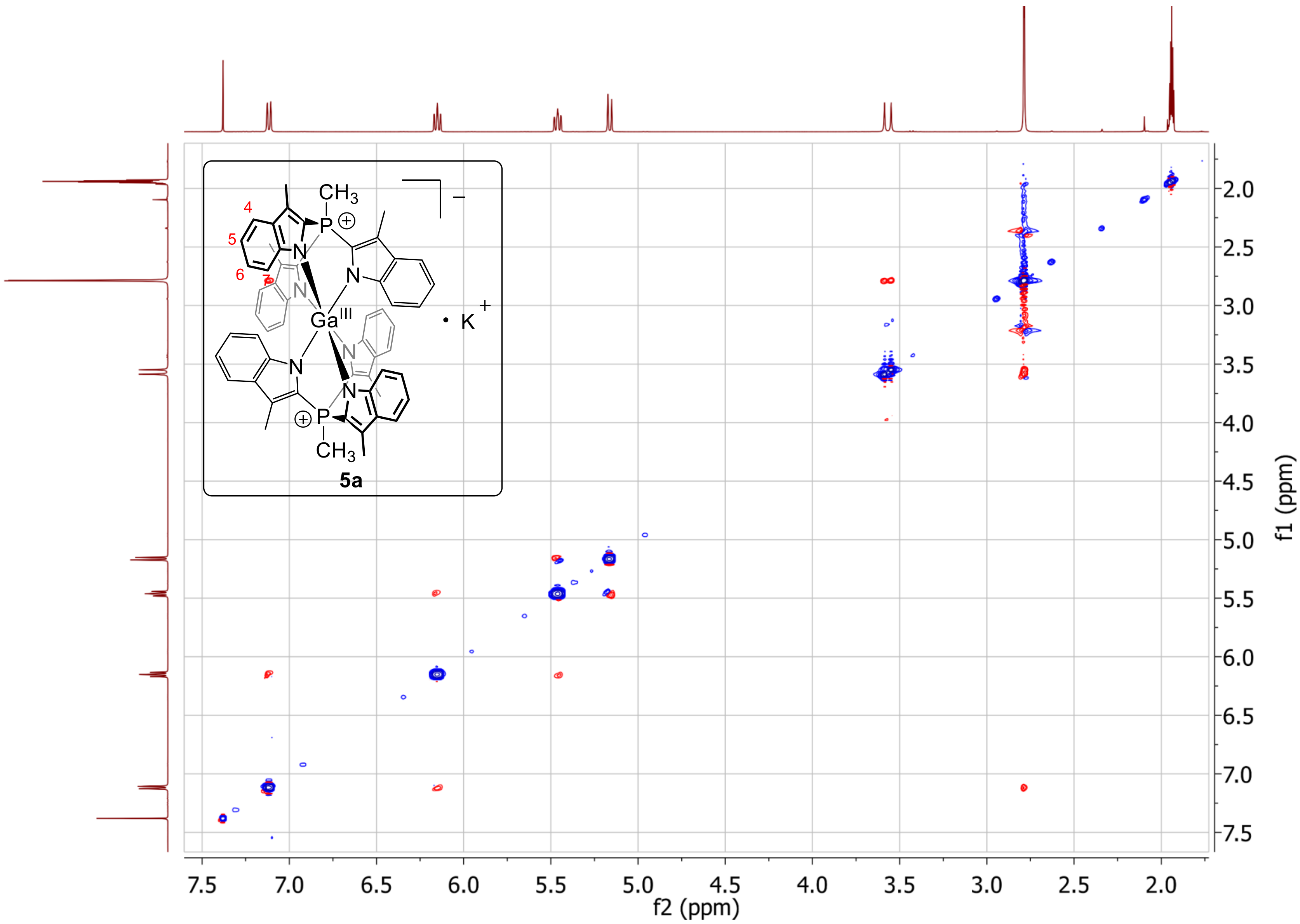

Figure S57. ^{1}H NOESY spectrum of compound **5a** in acetonitrile-d_3.

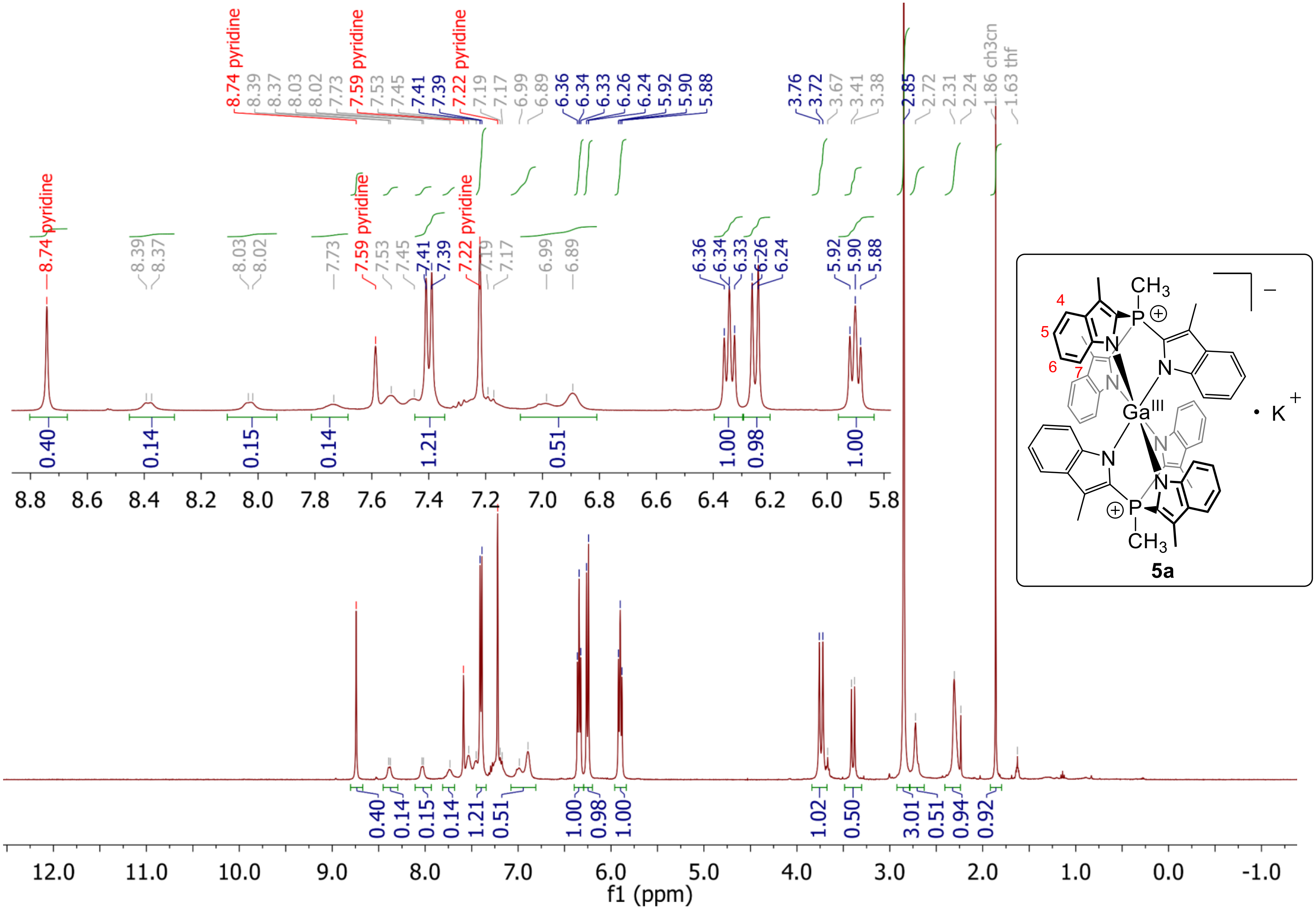

Figure S58. ^{1}H NMR (400 MHz) spectrum of compound **5a** in pyridine-d_5 (see *Section S2.1* for an explanation).

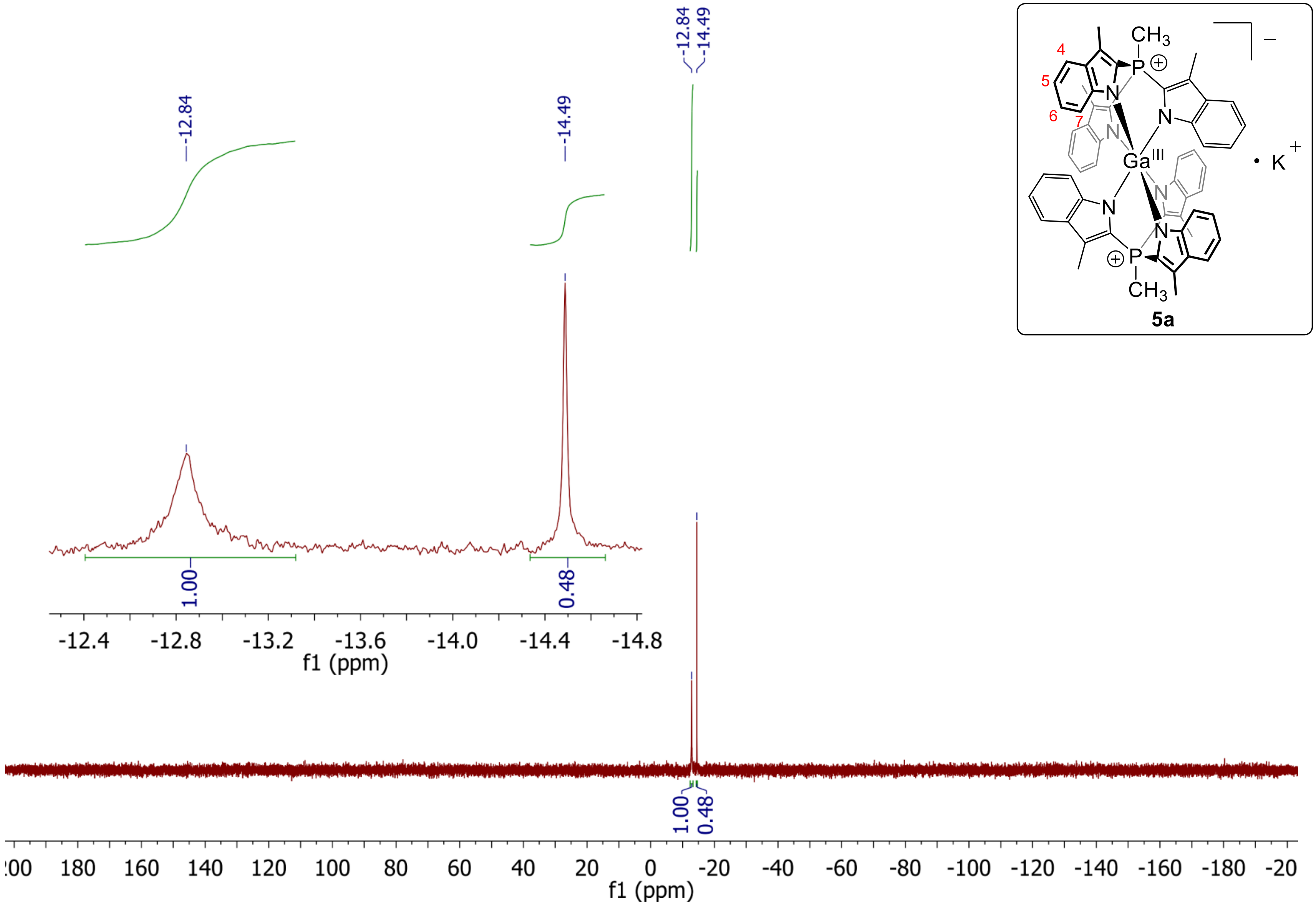

Figure S59. $^{31}P\{^1H\}$ NMR (162 MHz) spectrum of compound **5a** in pyridine-d_5 (see *Section S2.1* for an explanation).

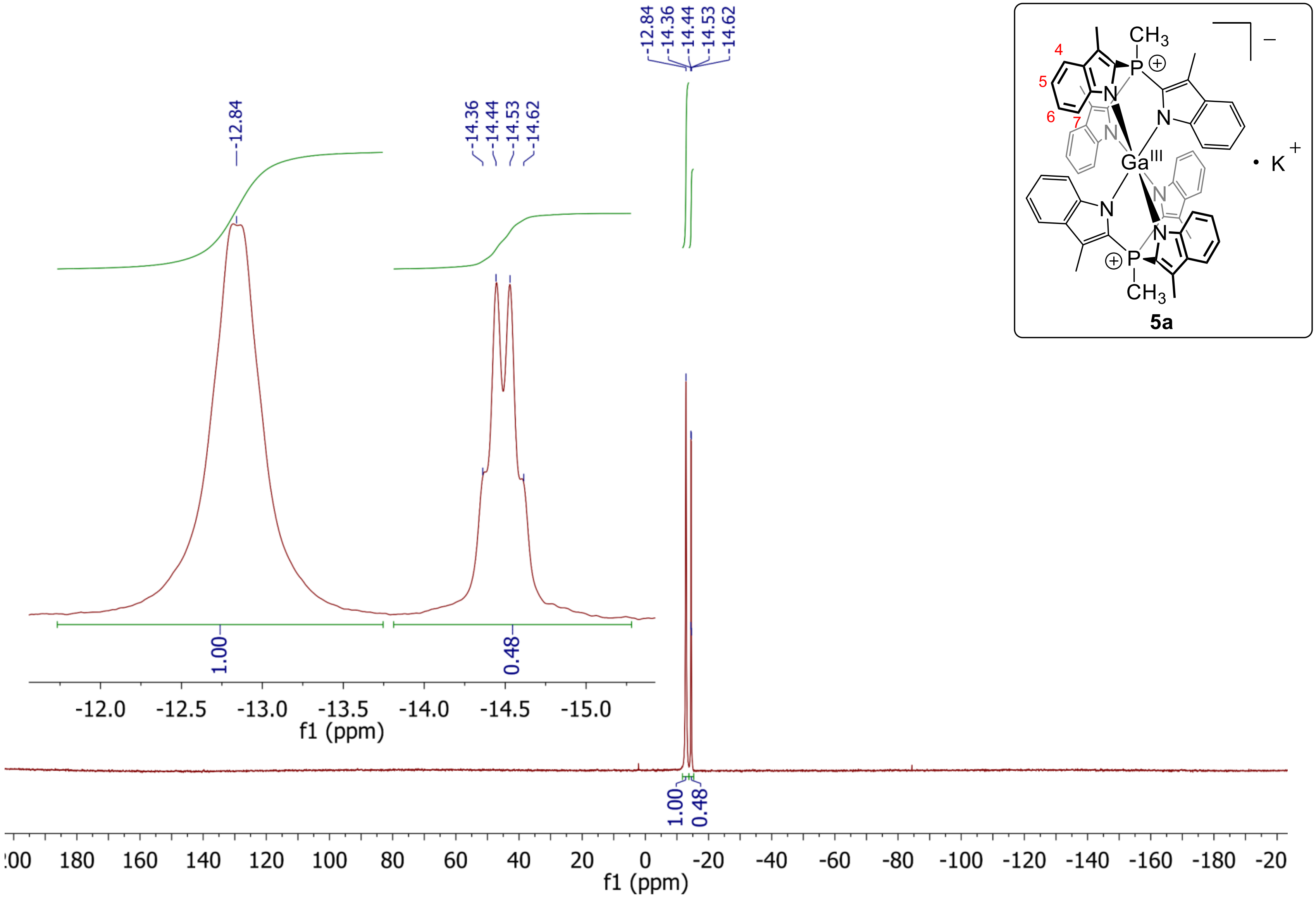

Figure S60. ^{31}P NMR (162 MHz) spectrum of compound **5a** in pyridine-d_5 (see *Section S2.1* for an explanation).

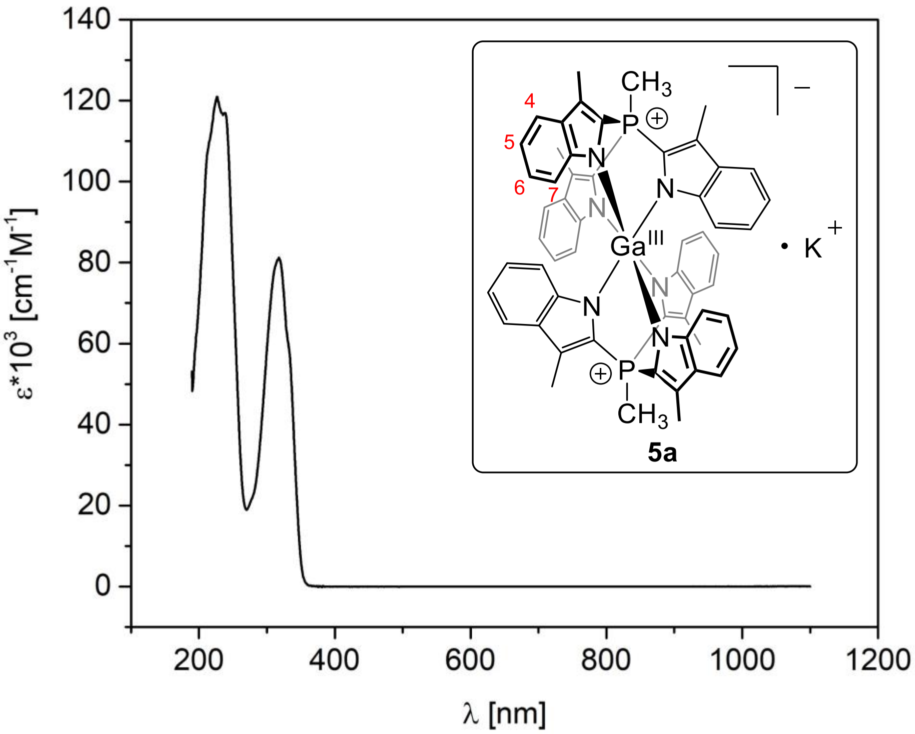

Figure S61. UV-Vis spectrum of compound **5a** in acetonitrile.

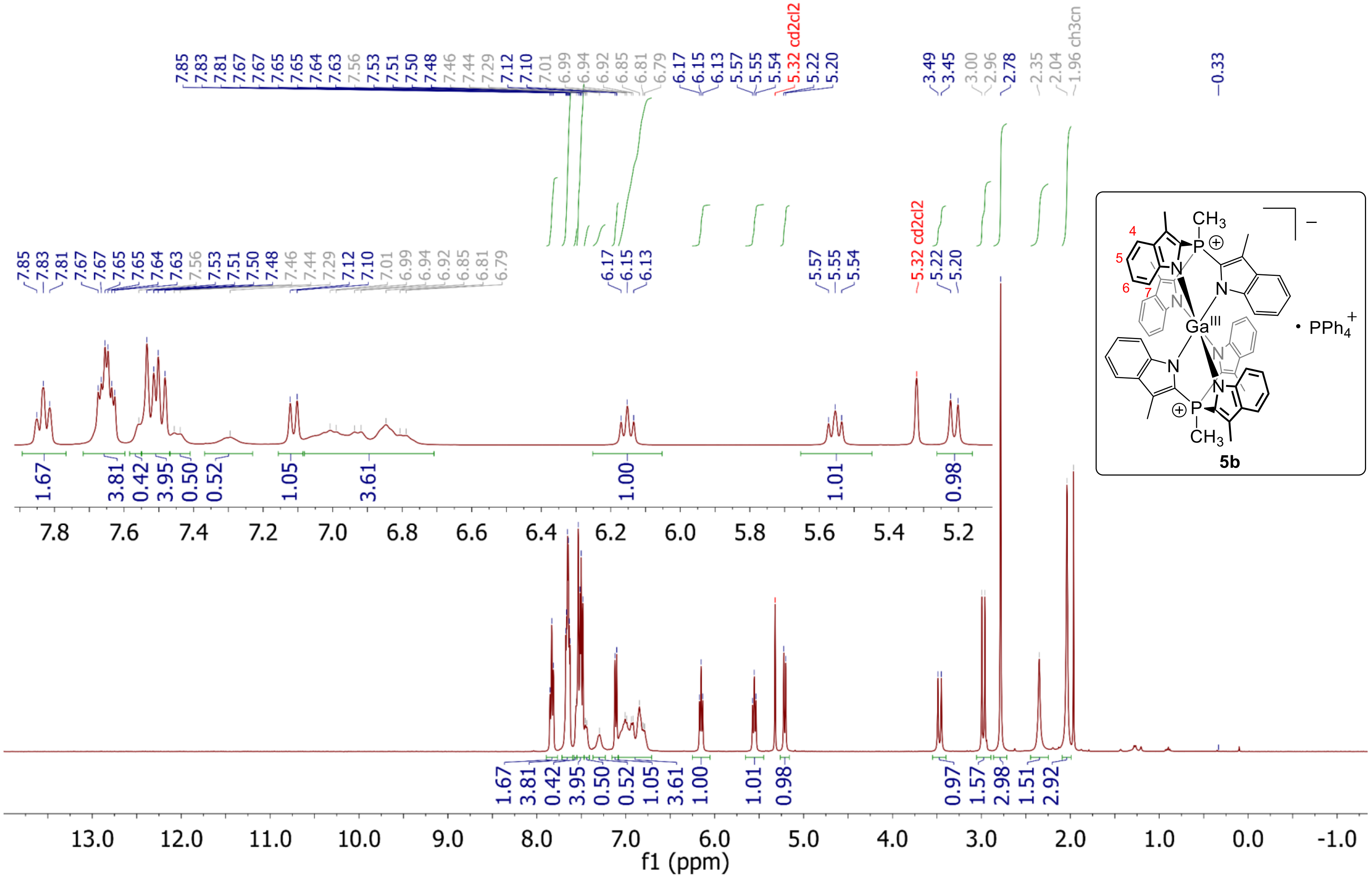

Figure S62. ^{1}H NMR (400 MHz) spectrum of compound **5b** in dichloromethane-d_2 (see *Section S2.1* for an explanation).

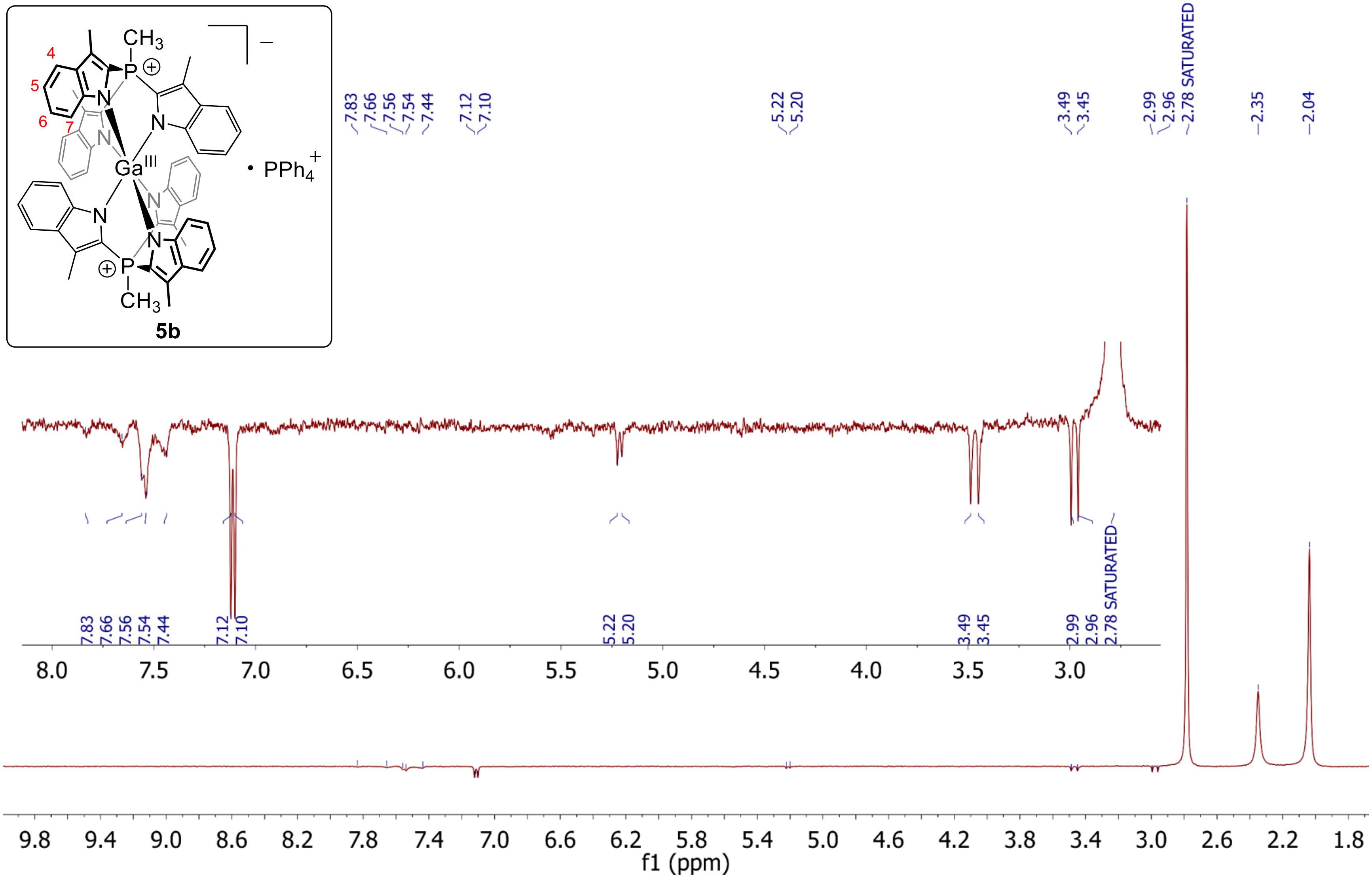

Figure S63. ^{1}H NOE (400 MHz) spectrum of compound **5b** in dichloromethane-d_2 (see *Section S2.1* for an explanation). The signal at 2.78 ppm is being saturated.

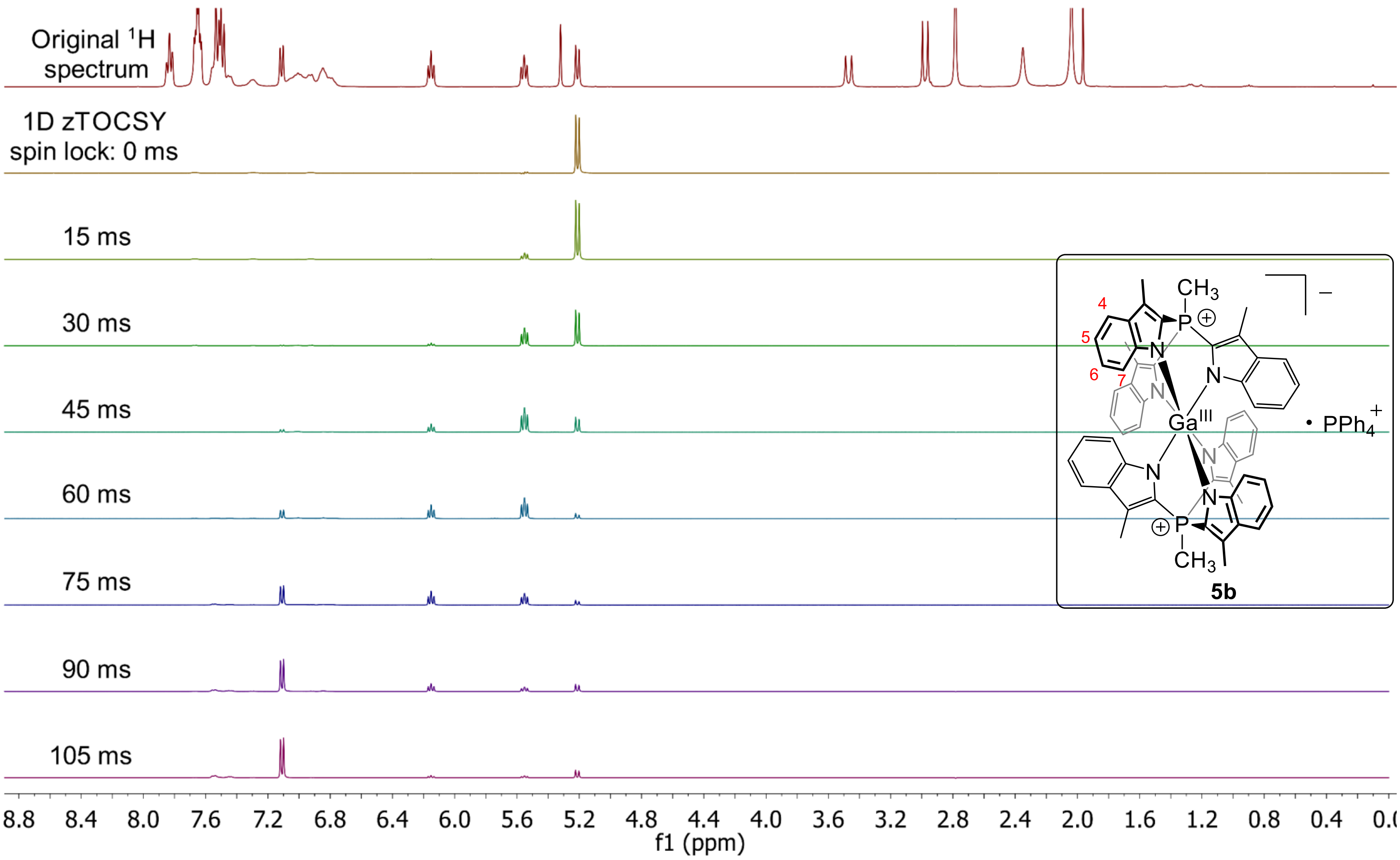

Figure S64. ¹H 1D zTOCSY (400 MHz) spectrum of compound **5b** in dichloromethane-d_2 (see *Section S2.1* for an explanation). The signal at 5.21 ppm selectively excited.

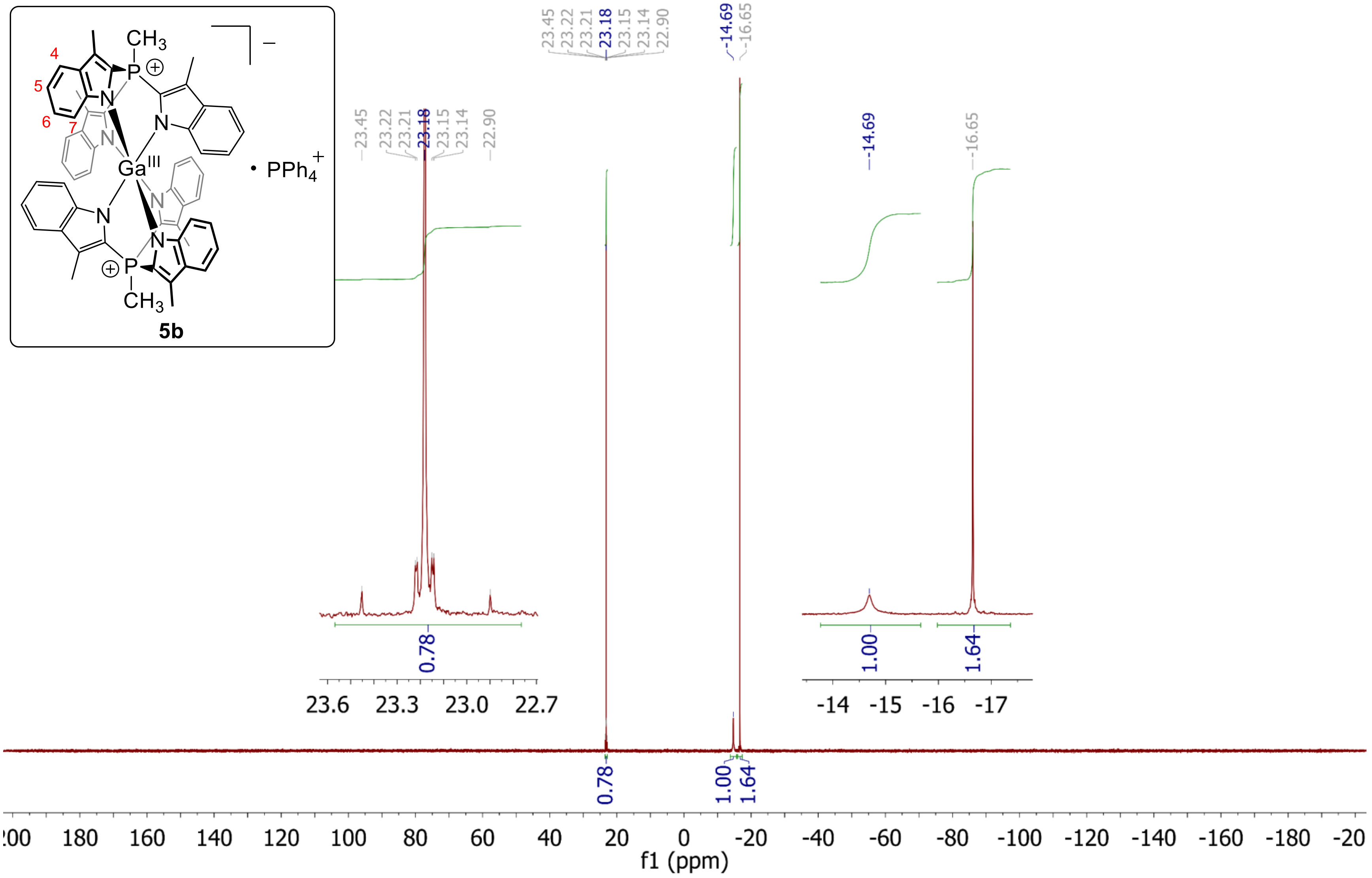

Figure S65. $^{31}P\{^{1}H\}$ NMR (162 MHz) spectrum of compound **5b** in dichloromethane-d_2 (see *Section S2.1* for an explanation).

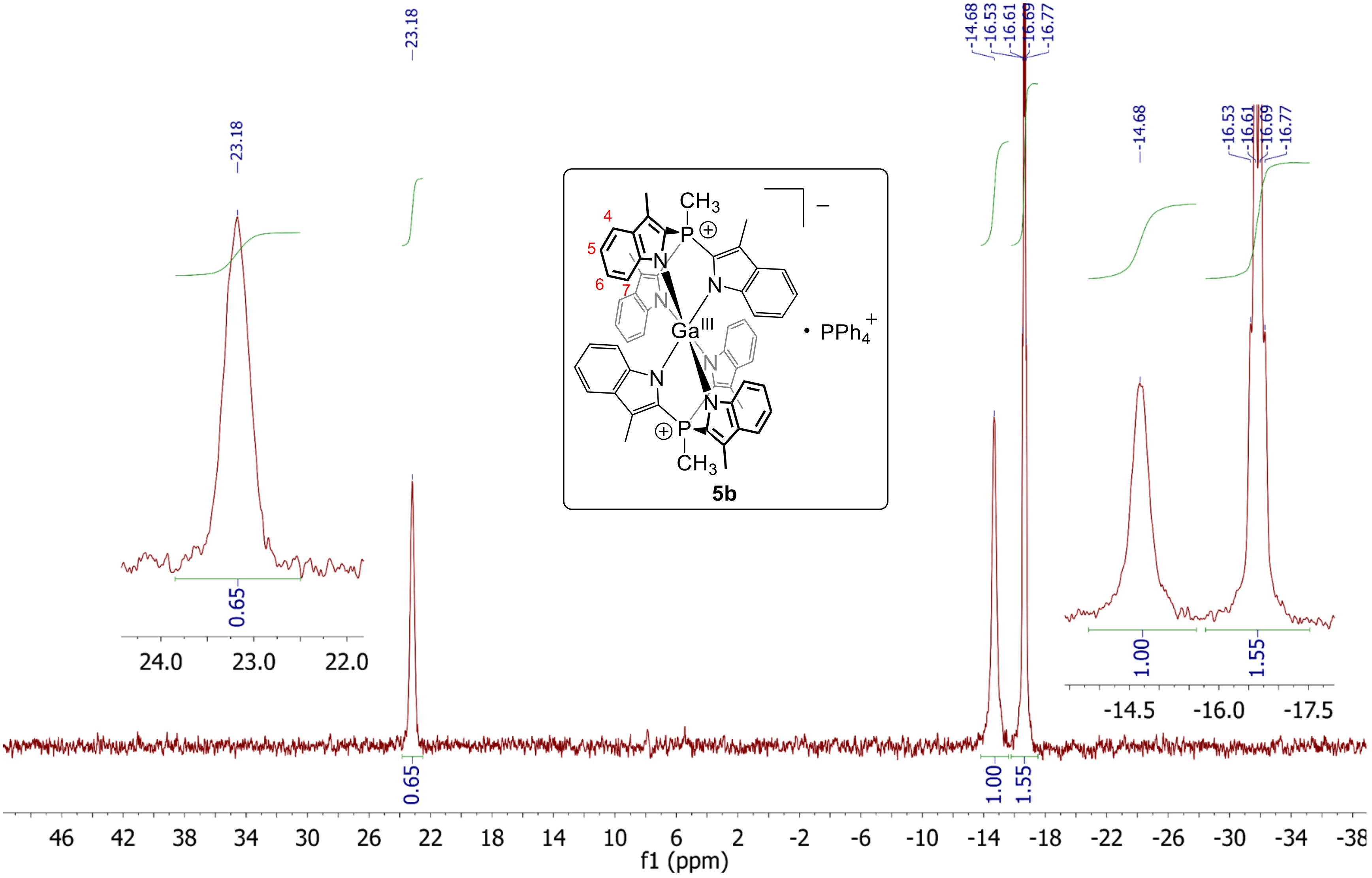

Figure S66. ^{31}P NMR (162 MHz) spectrum of compound **5b** in dichloromethane-d_2 (see *Section S2.1* for an explanation).

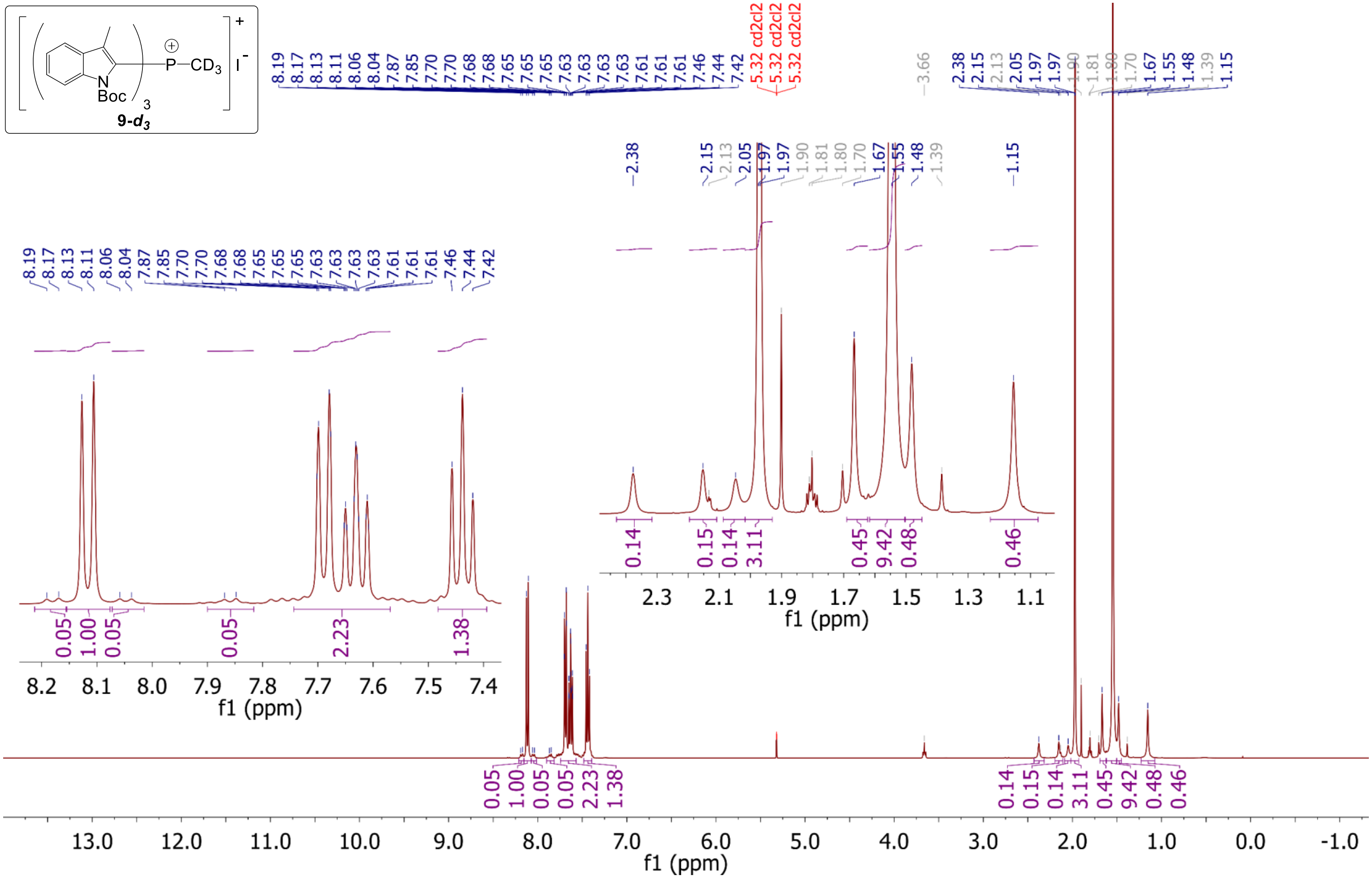

Figure S67. ^{1}H NMR (400 MHz) spectrum of compound **9-*d*$_3$** in dichloromethane-d_2.

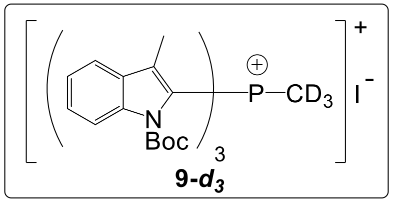

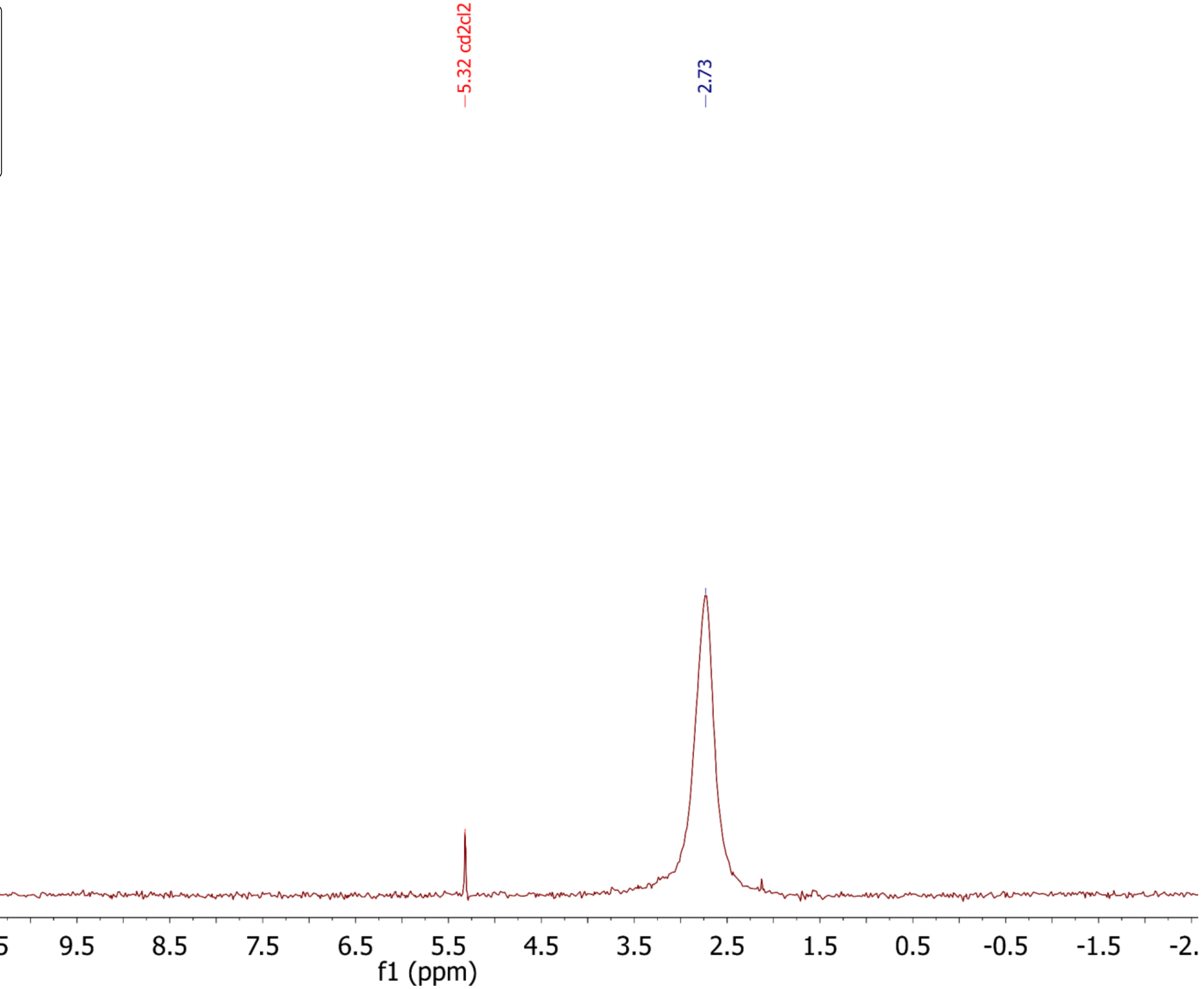

Figure S68. ^{2}H NMR (61 MHz) spectrum of compound **9-*d*$_3$** in dichloromethane-*h*$_2$.

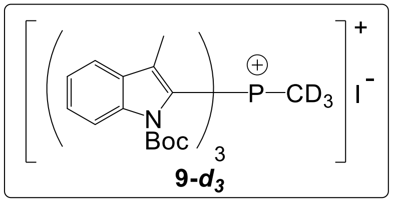

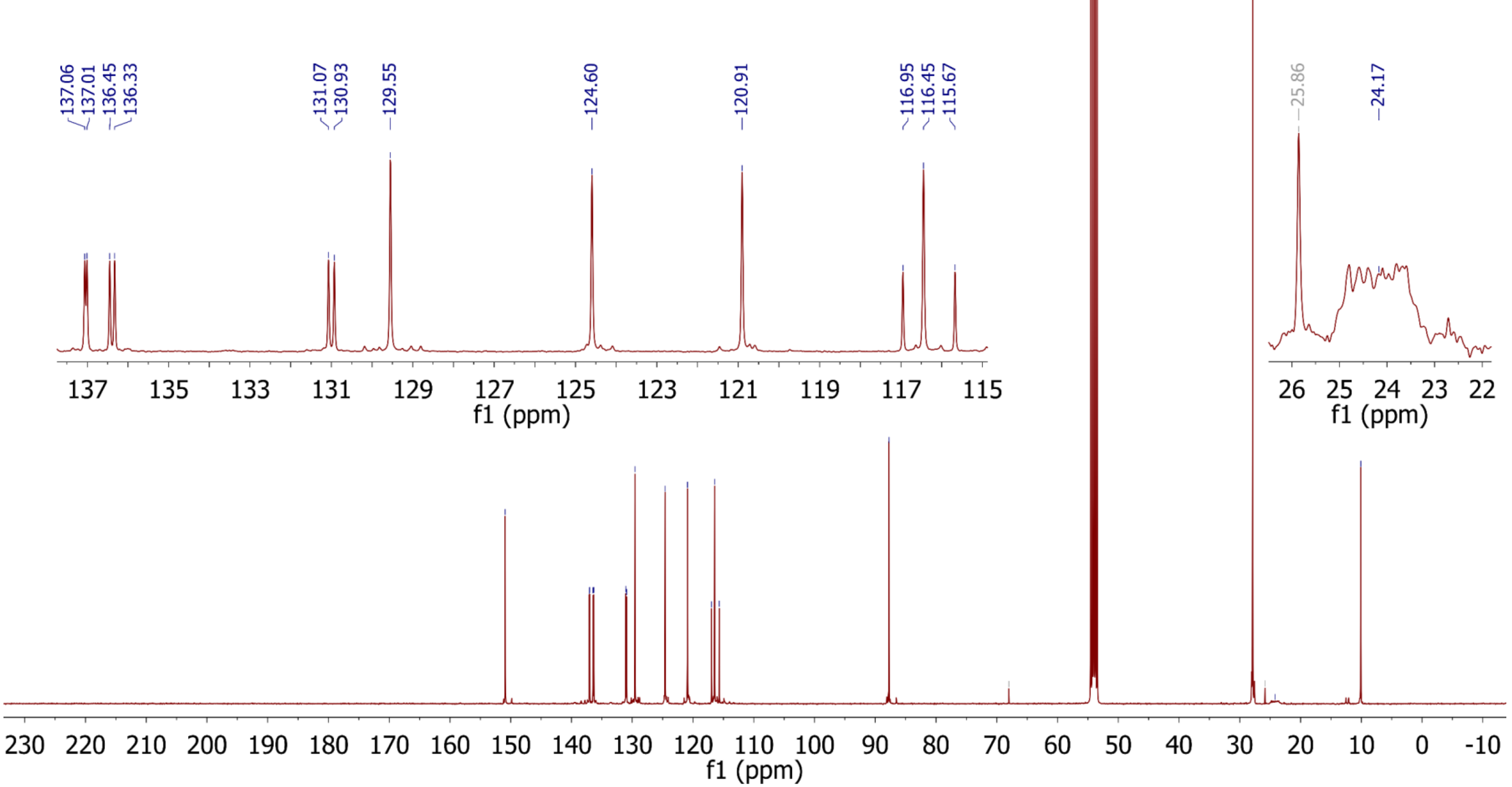

Figure S69. ^{13}C NMR (101 MHz) spectrum of compound **9-*d*$_3$** in dichloromethane-*d*$_2$.

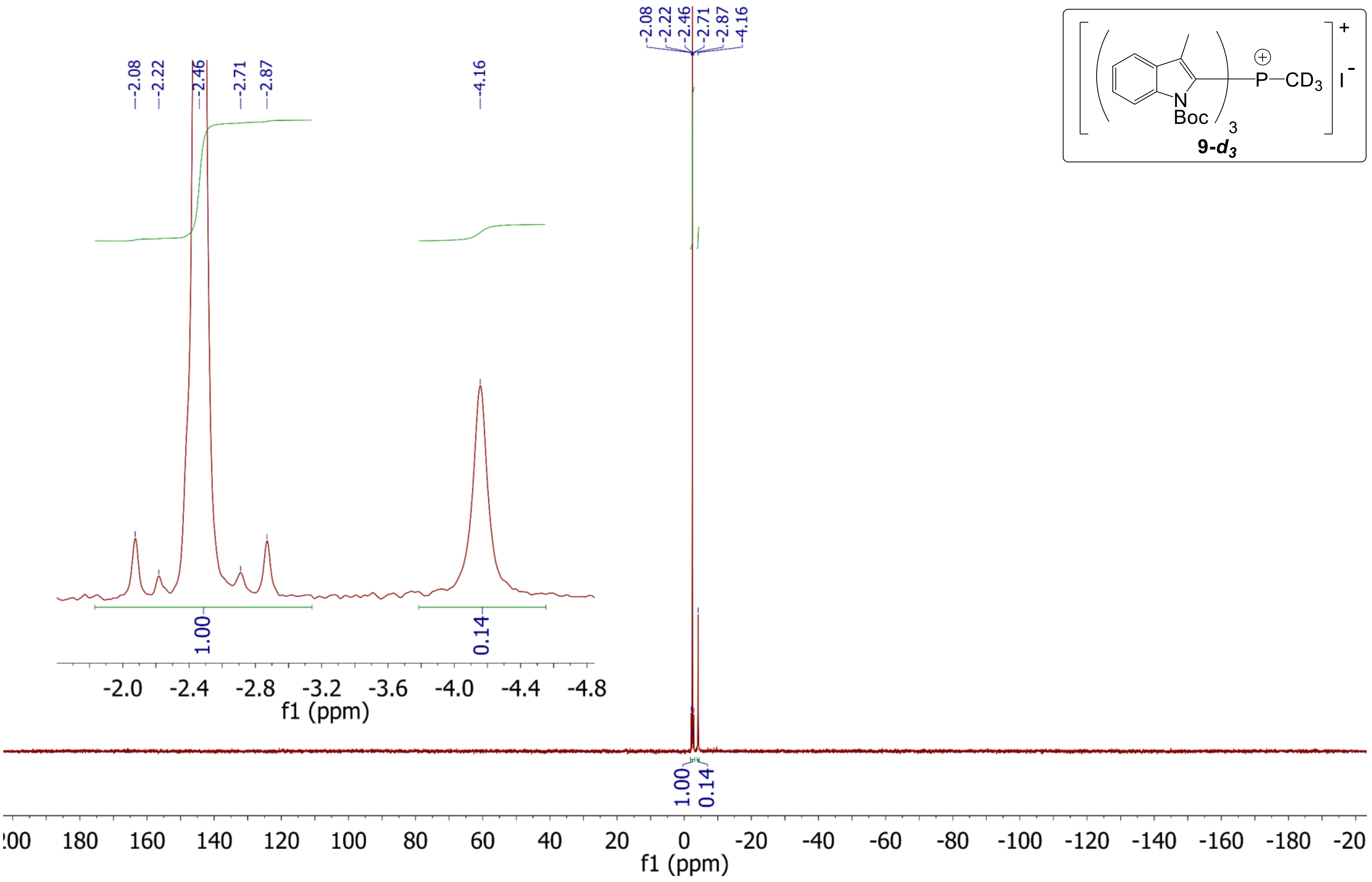

Figure S70. $^{31}P\{^1H\}$ NMR (162 MHz) spectrum of compound **9-*d*₃** in dichloromethane-d_2.

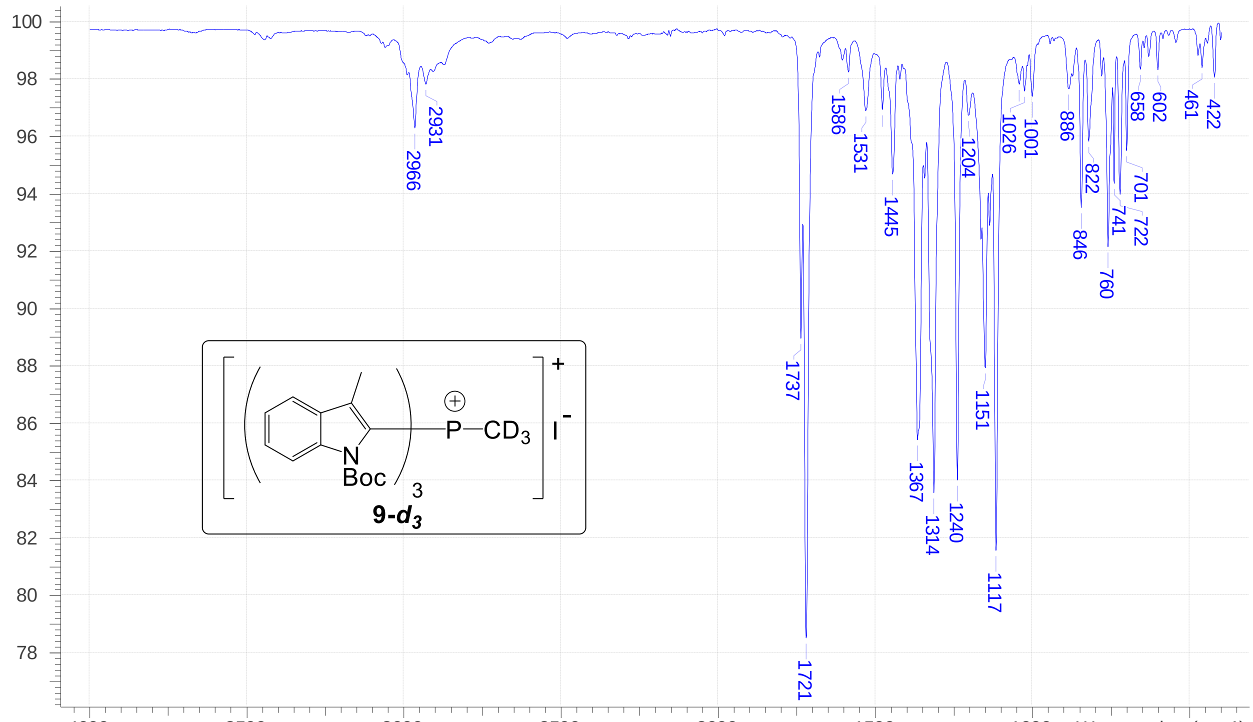

Figure S71. ATR-FTIR (neat) spectrum of compound **9-*d*$_3$**

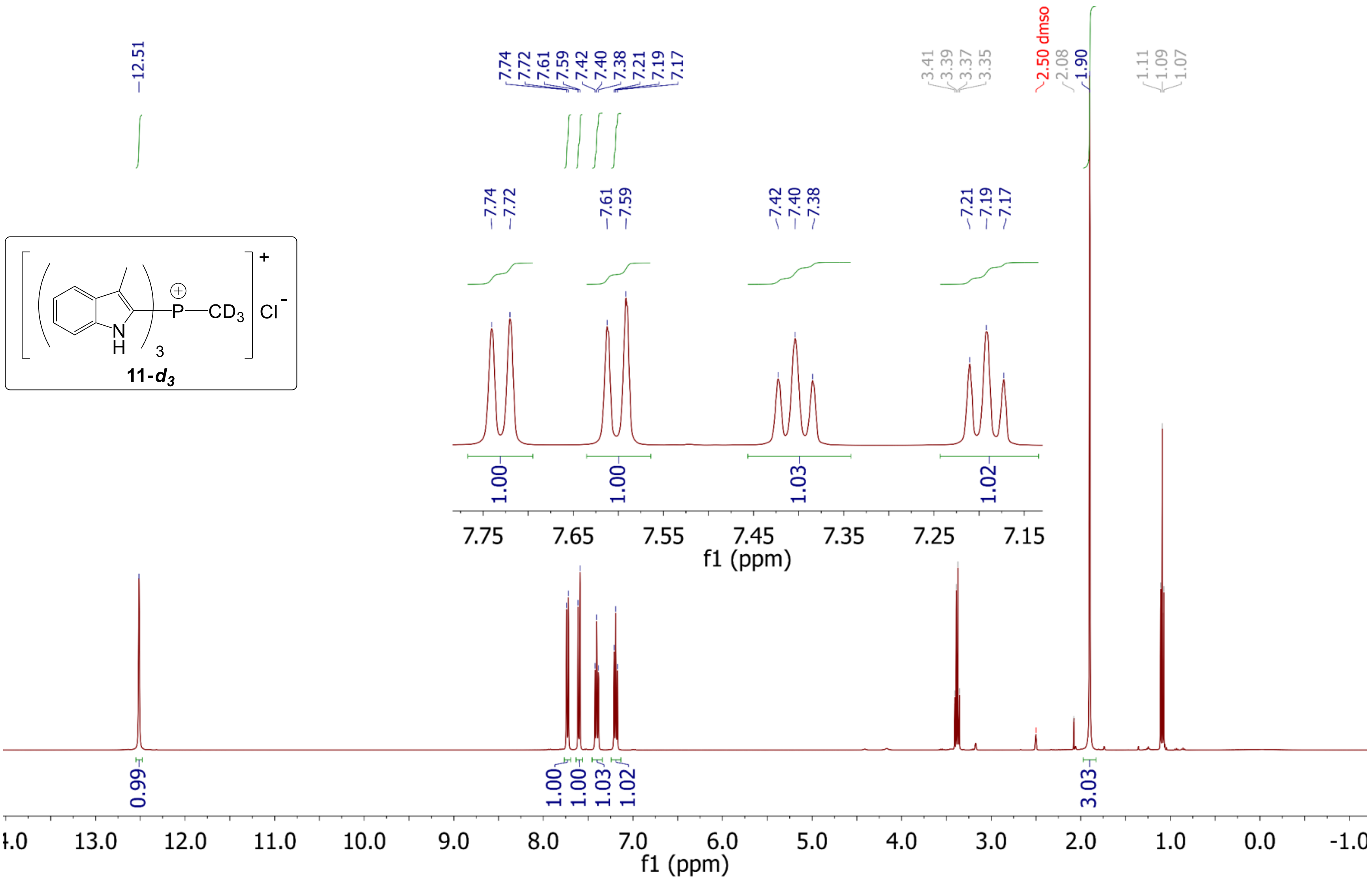

Figure S72. ^{1}H NMR (400 MHz) spectrum of compound **11-d_3** in DMSO-d_6.

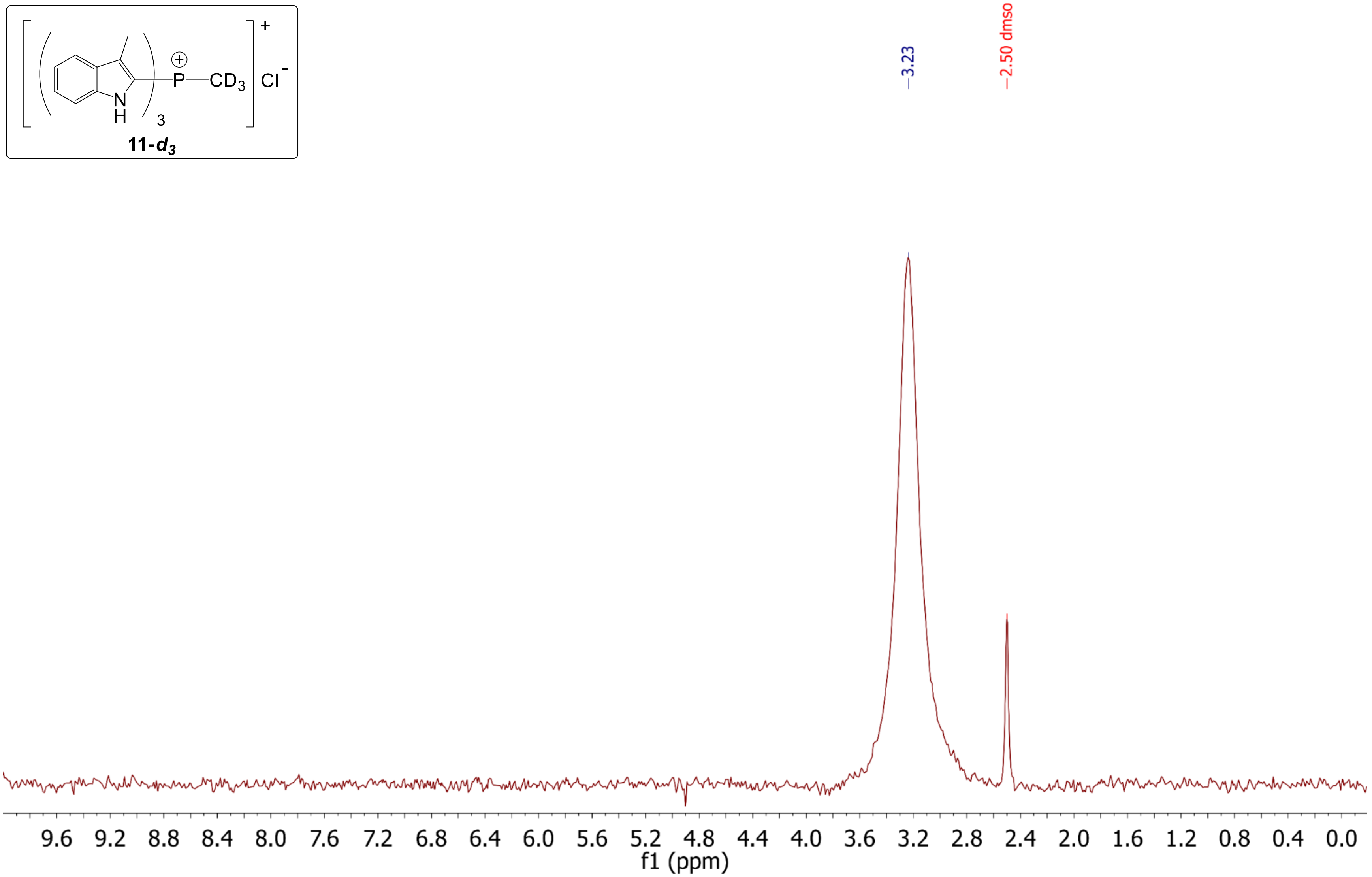

Figure S73. ^{2}H NMR (61 MHz) spectrum of compound **11-*d*$_3$** in DMSO-*h*$_6$.

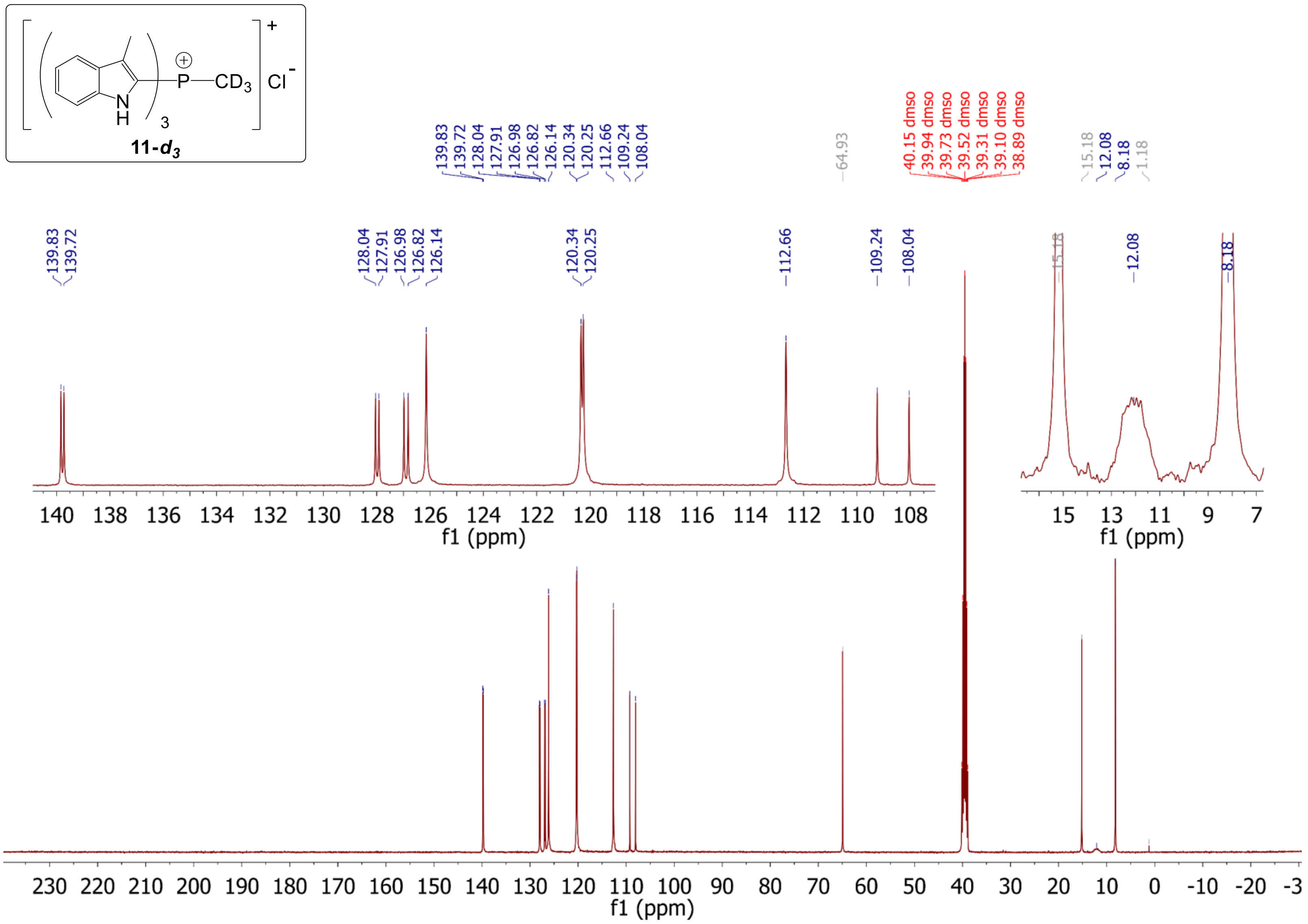

Figure S74. ^{13}C NMR (101 MHz) spectrum of compound **11-*d*$_3$** in DMSO-*d*$_6$.

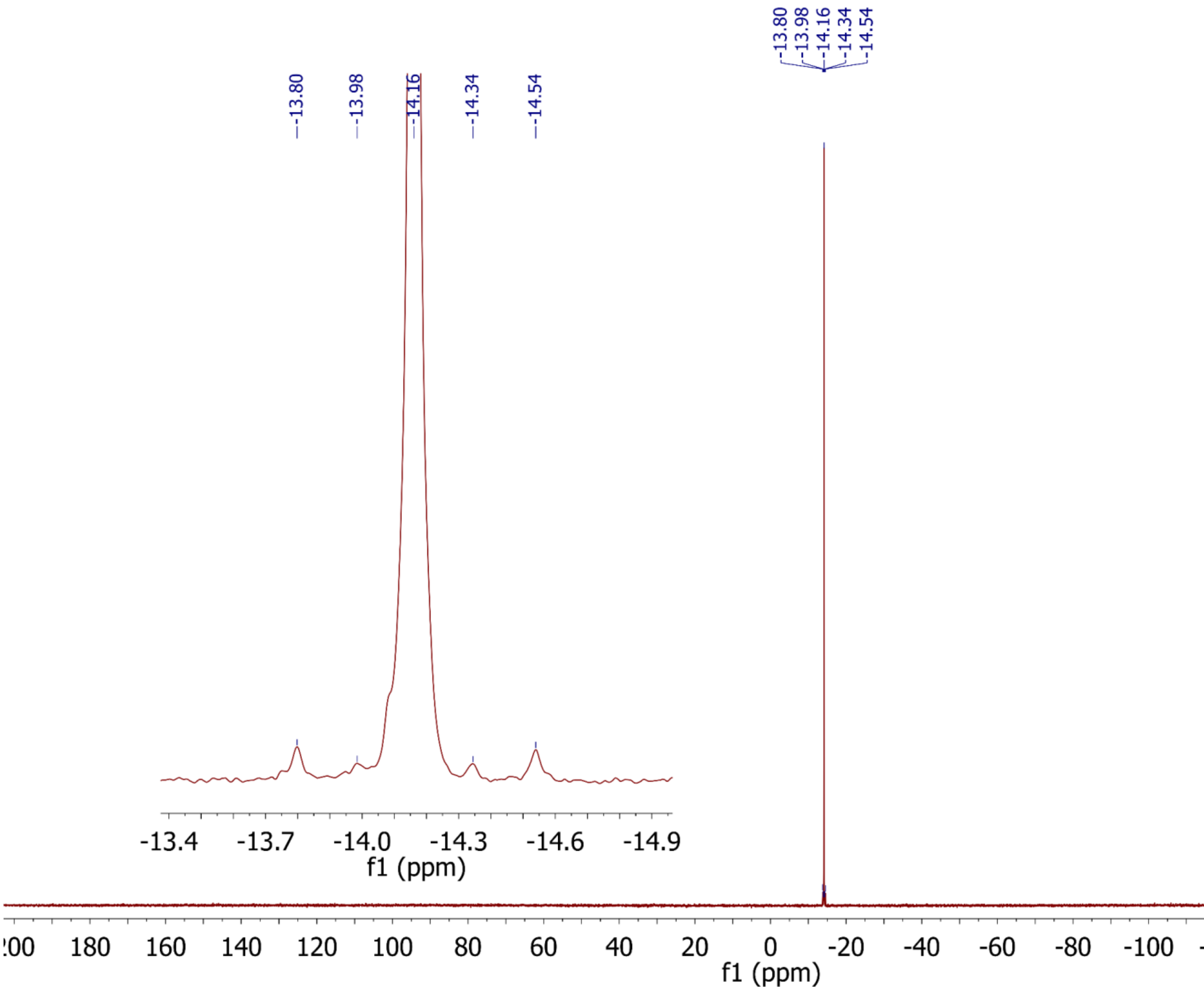

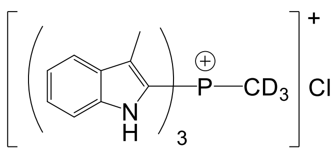

11-*d*$_3$

Figure S75. $^{31}P\{^{1}H\}$ NMR (162 MHz) spectrum of compound **11-*d*$_3$** in DMSO-d_6.

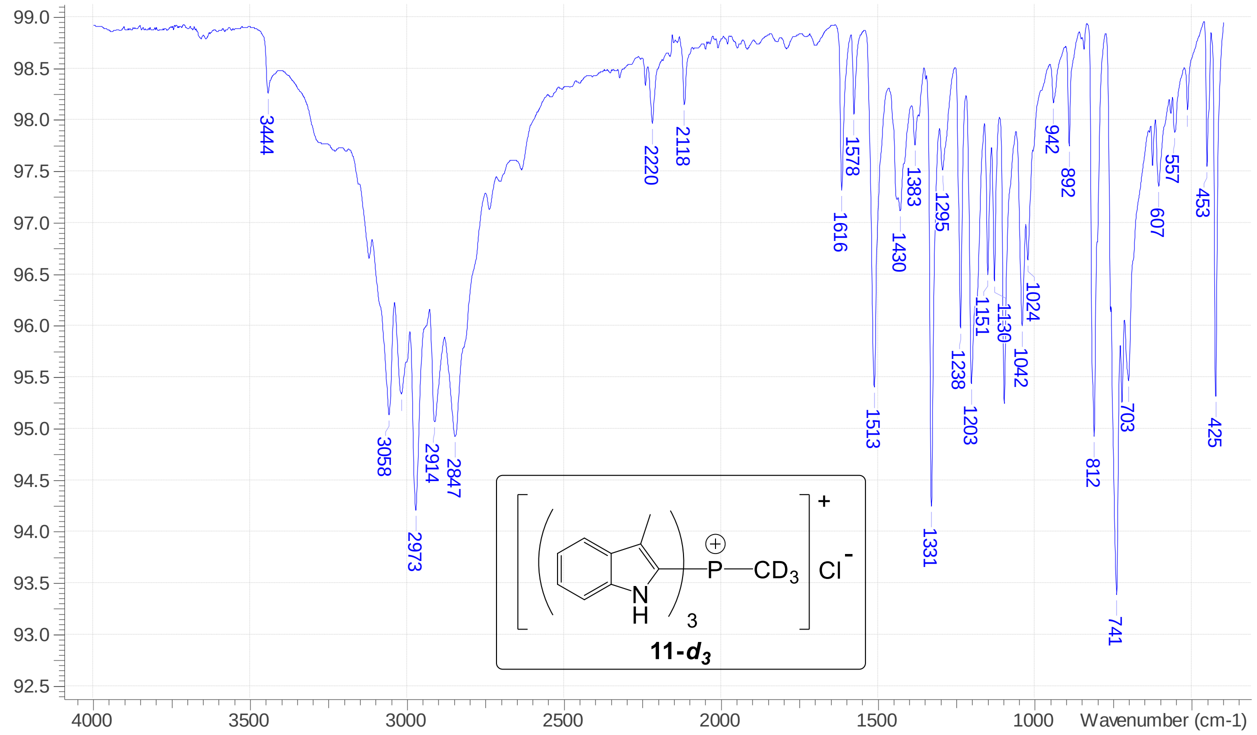

Figure S76. ATR-FTIR (neat) spectrum of compound **11-*d*$_3$**

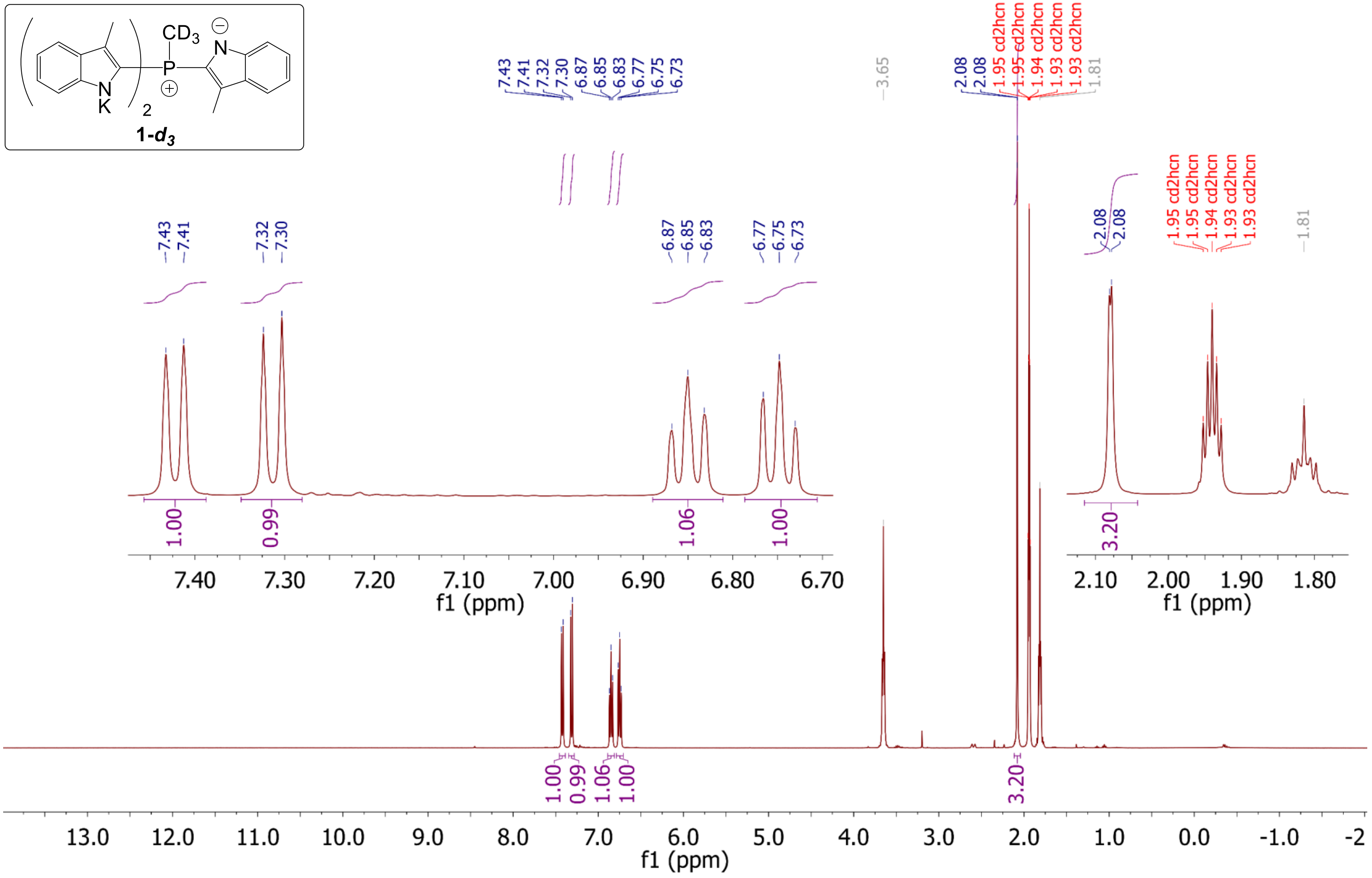

Figure S77. ^{1}H NMR (400 MHz) spectrum of compound **1-*d*$_3$** in acetonitrile-*d*$_3$.

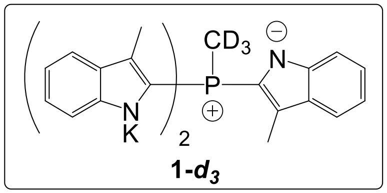

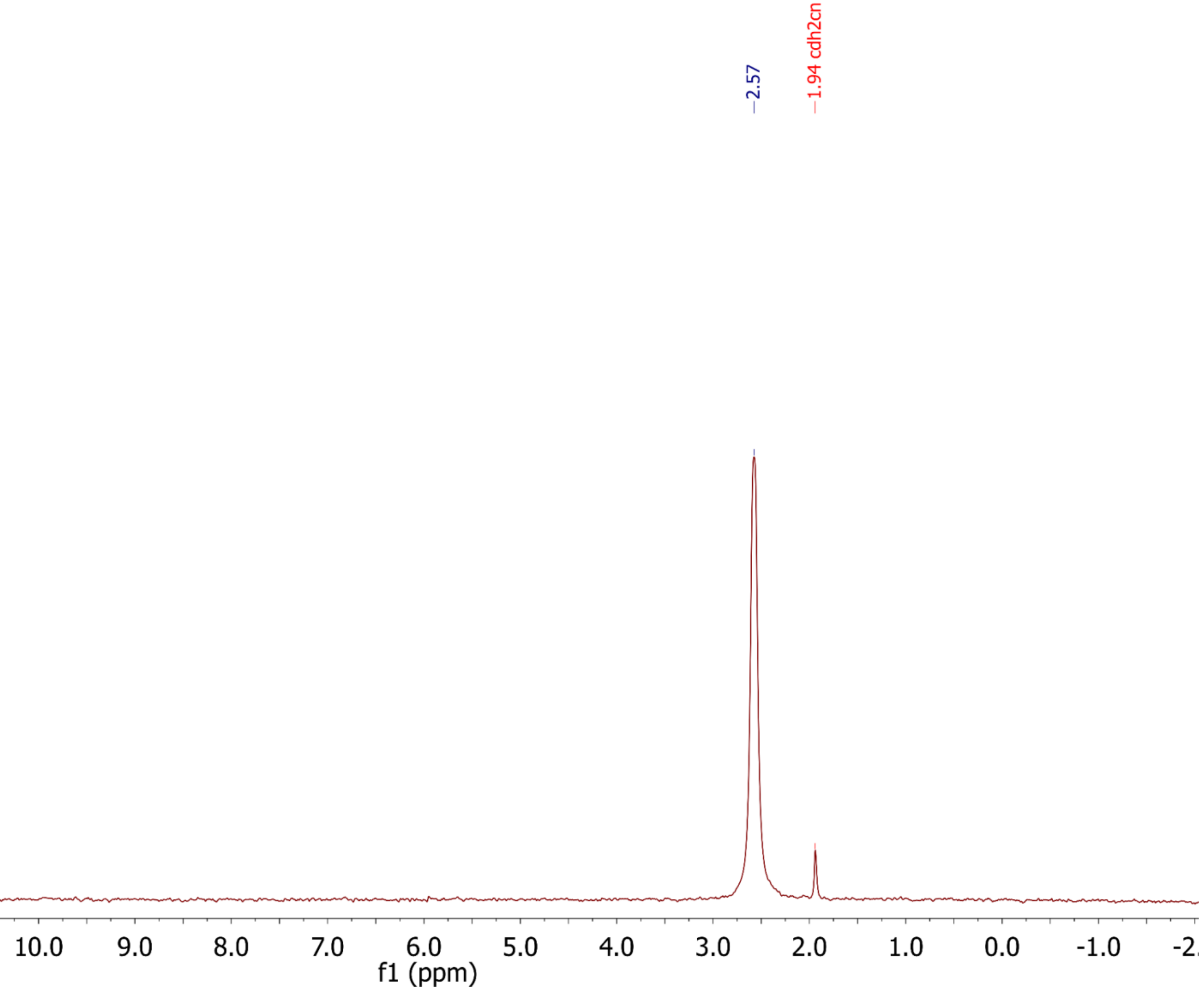

Figure S78. ^{2}H NMR (61 MHz) spectrum of compound **1-*d*$_3$** in acetonitrile-*h*$_3$.

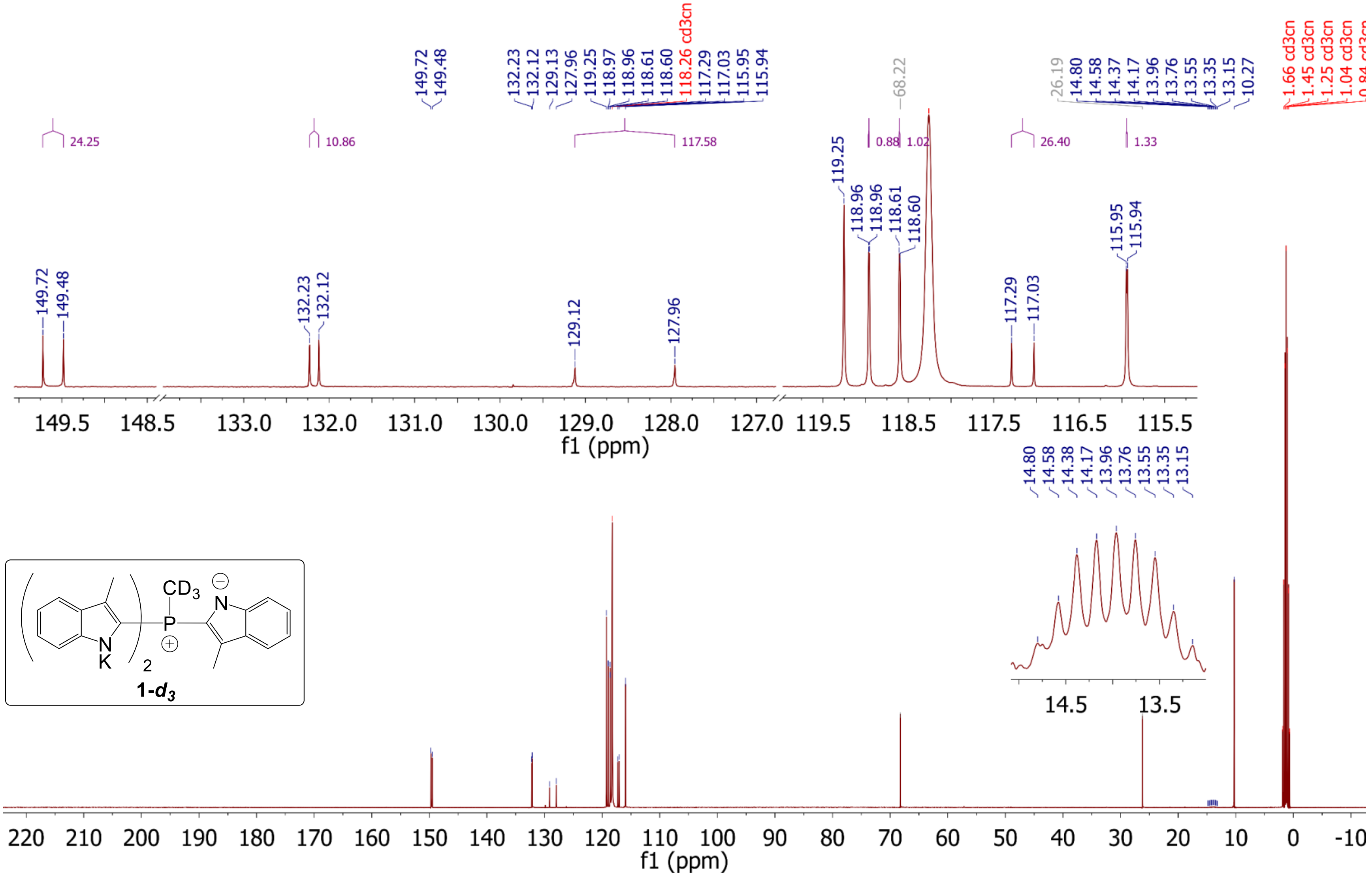

Figure S79. ^{13}C NMR (400 MHz) spectrum of compound **1-*d*$_3$** in acetonitrile-*d*$_3$.

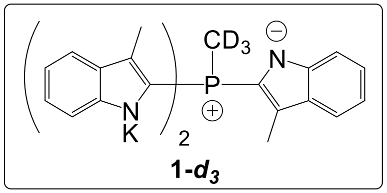

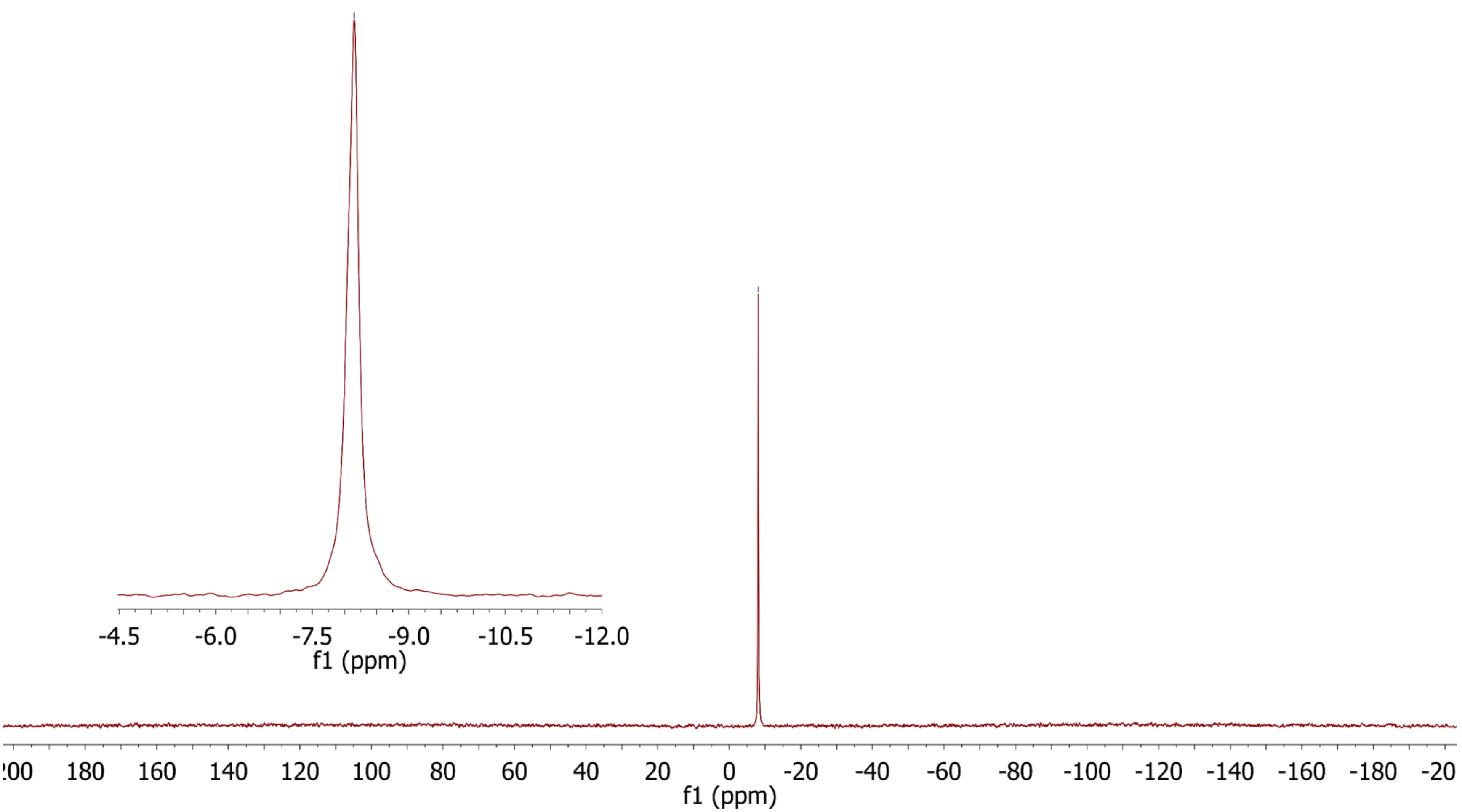

Figure S80. $^{31}P\{^{1}H\}$ NMR (162 MHz) spectrum of compound **1-*d*$_3$** in acetonitrile-*d*$_3$.

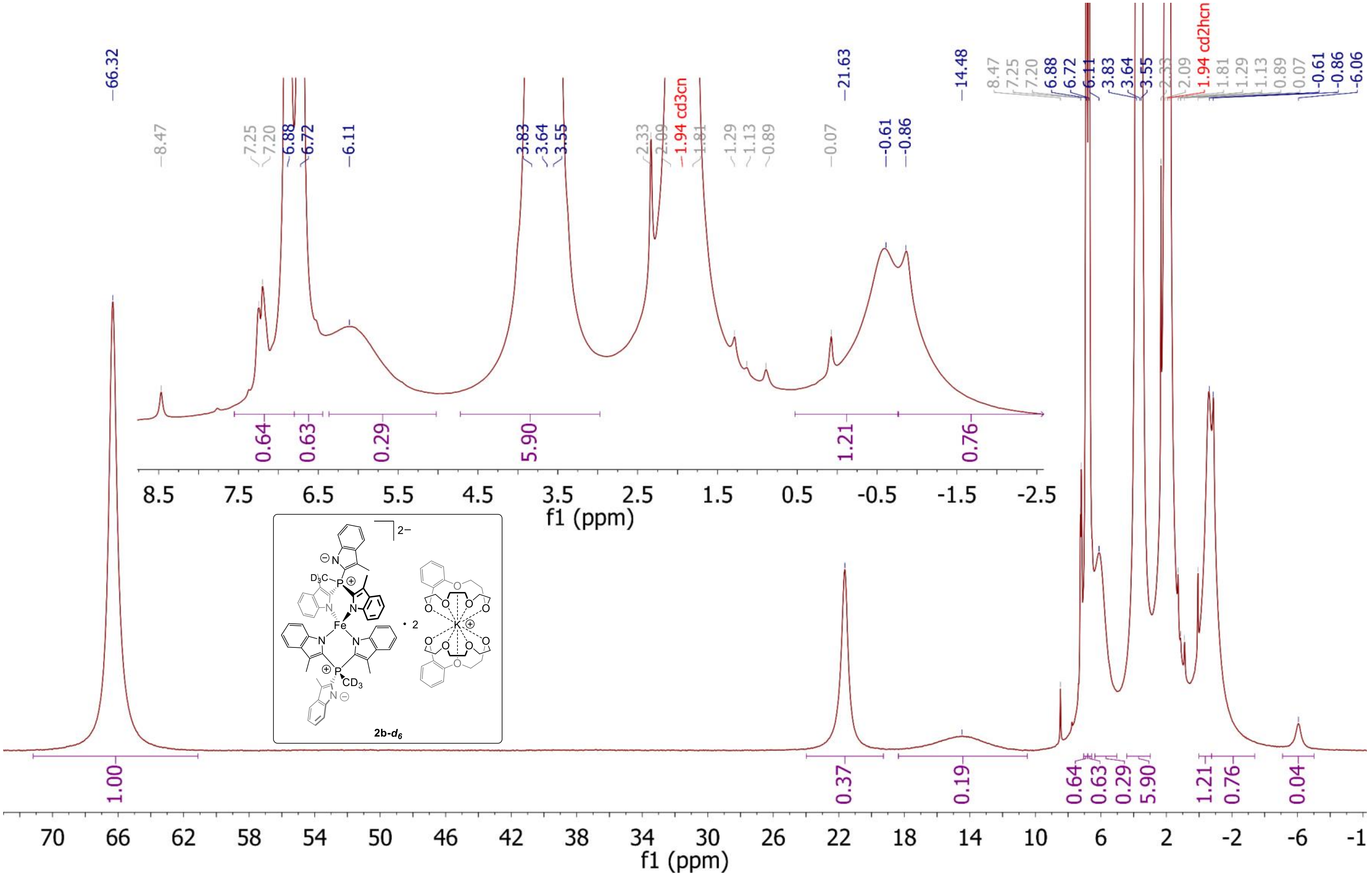

Figure S81. ^{1}H NMR (400 MHz) spectrum of compound **2b-*d*$_6$** in acetonitrile-*d*$_3$.

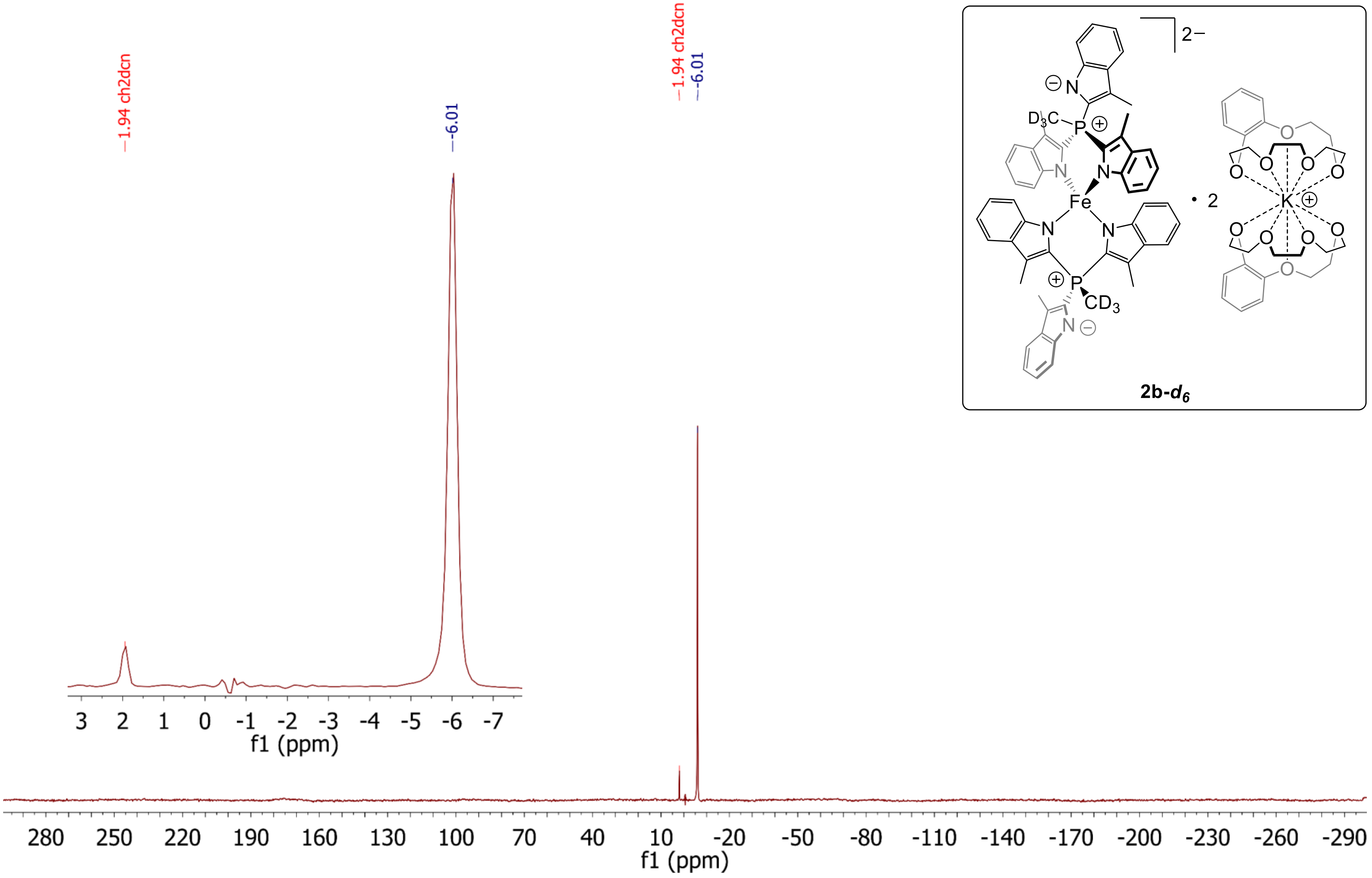

2b-*d*₆

Figure S82. ^{2}H NMR (61 MHz) spectrum of compound **2b-*d*$_6$** in acetonitrile-*h*$_3$.

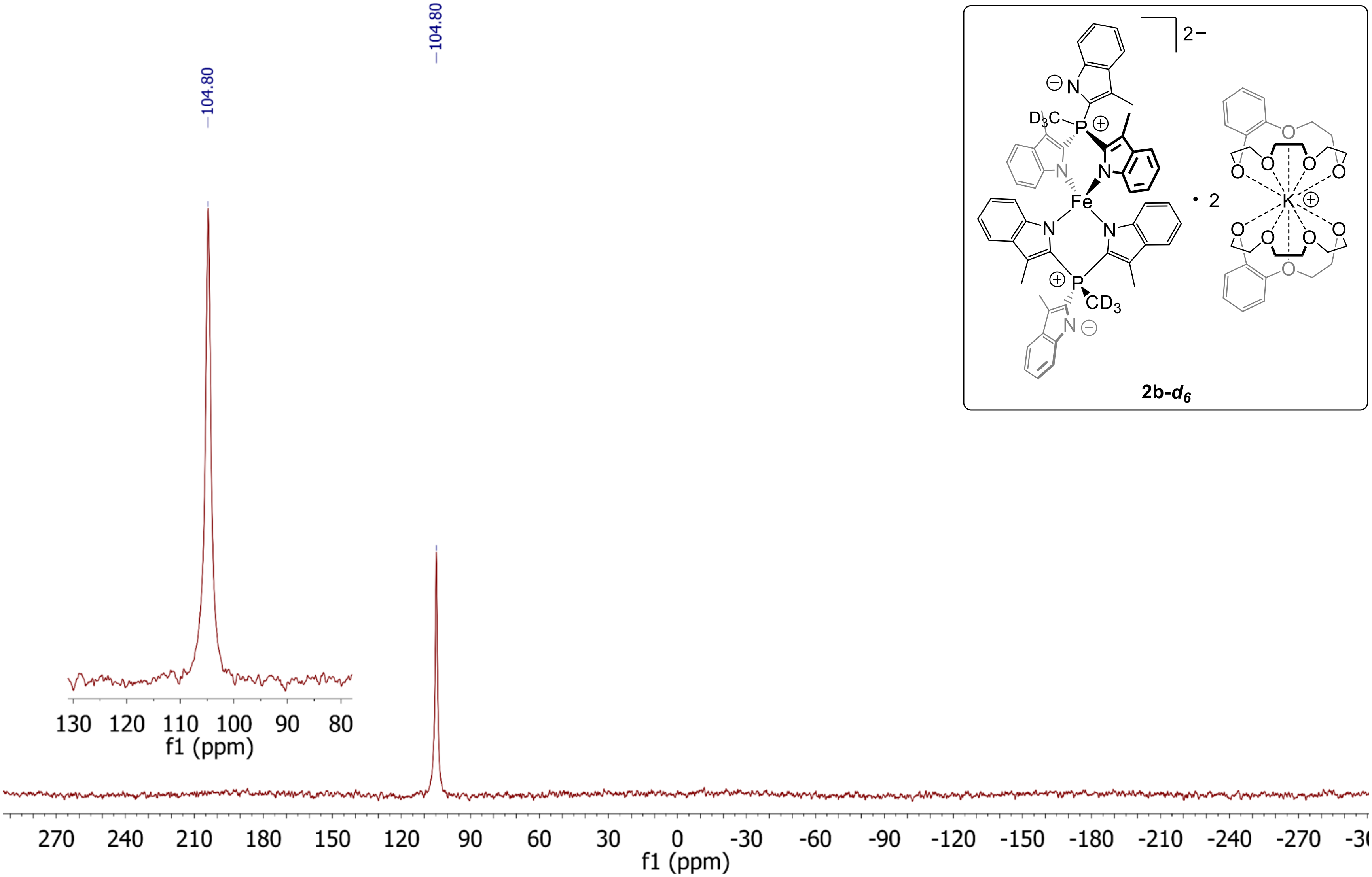

Figure S83. ^{31}P NMR (162 MHz) spectrum of compound **2b-*d*$_6$** in acetonitrile-*d*$_3$.

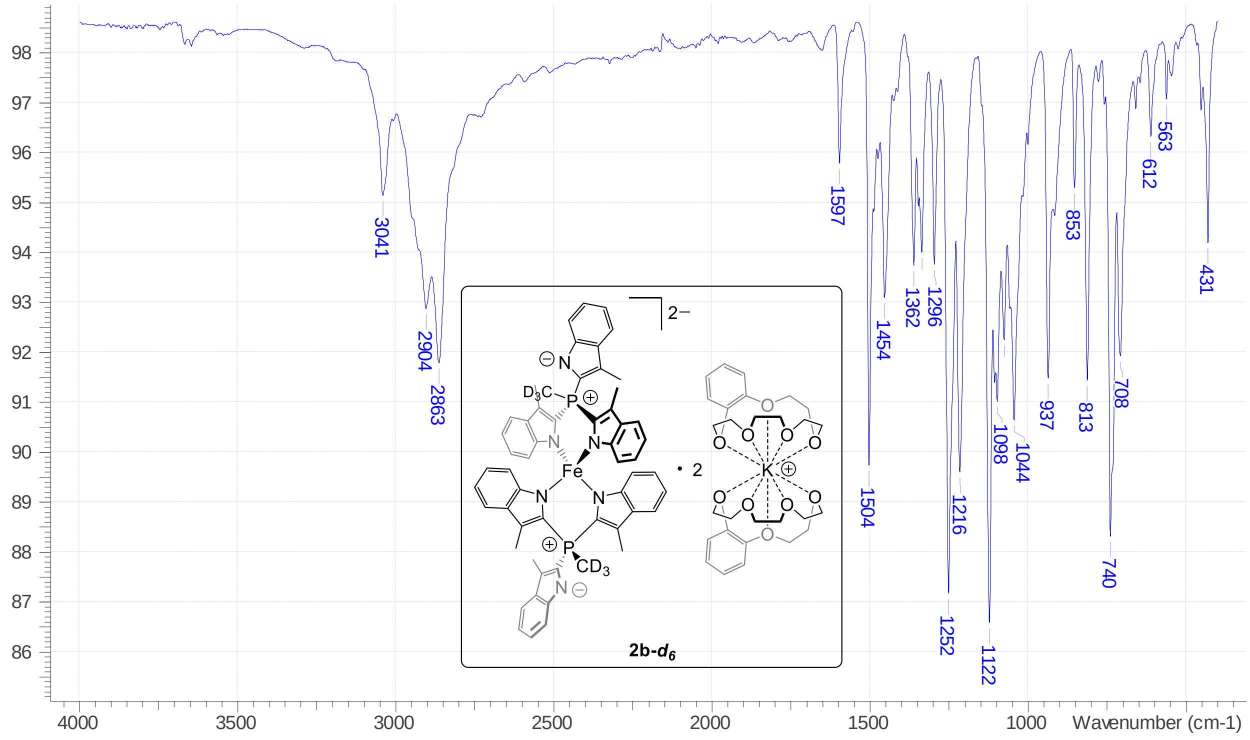

Figure S84. ATR-FTIR (neat) spectrum of compound **2b-*d*$_6$**.

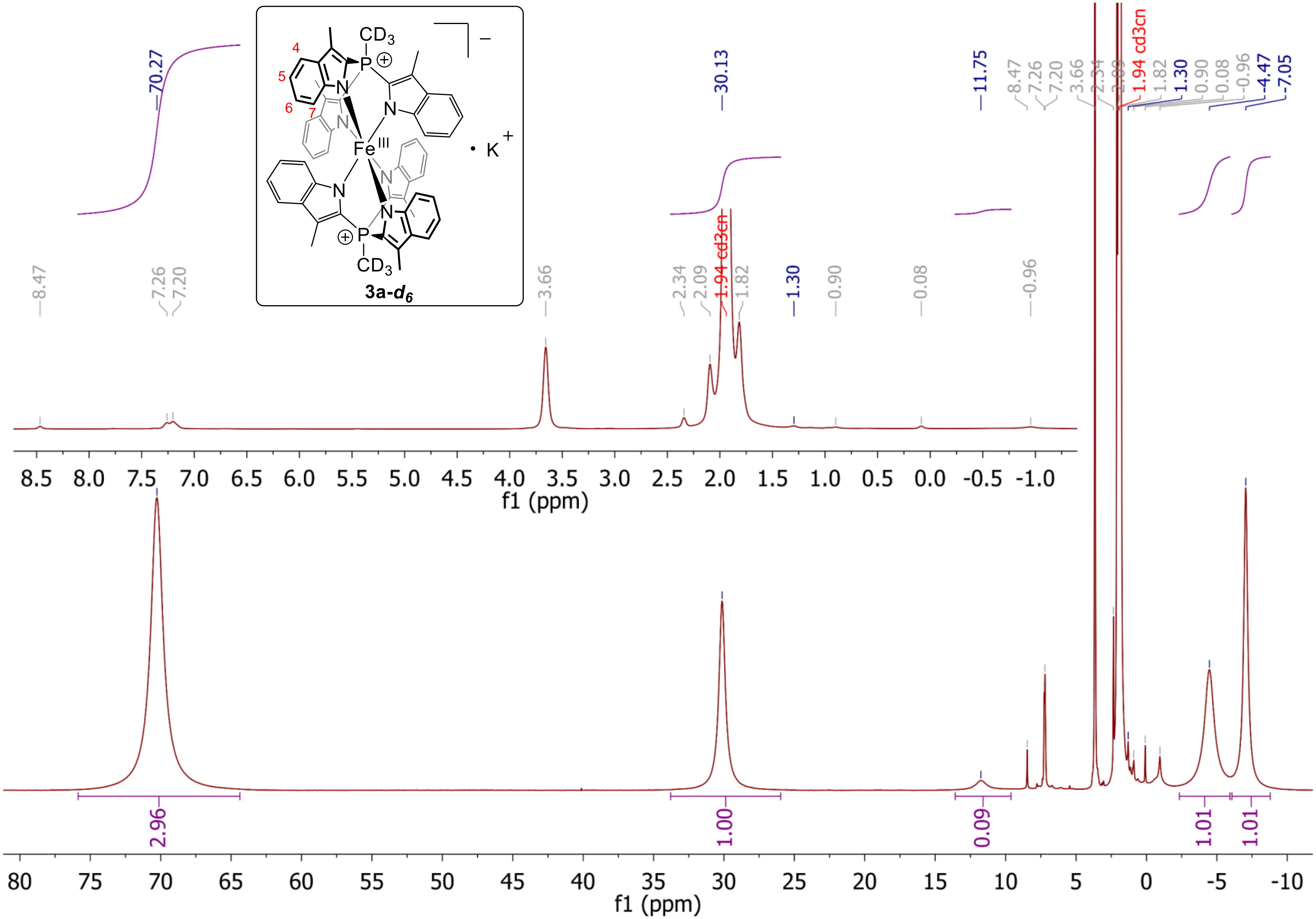

Figure S85. ^{1}H NMR (400 MHz) spectrum of compound **3a-*d*₆** in acetonitrile-d_3.

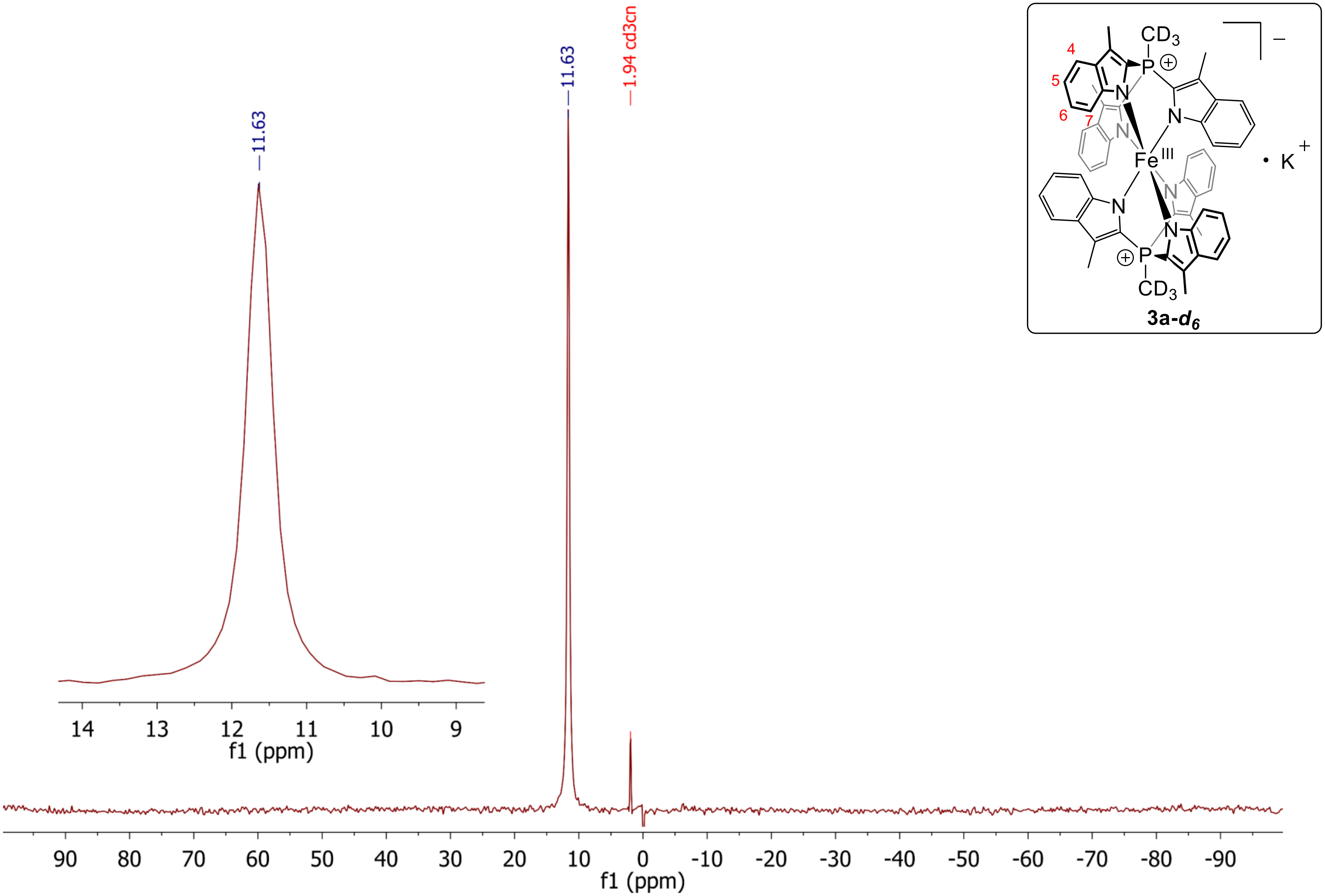

Figure S86. ^{2}H NMR (61 MHz) spectrum of compound **3a-*d*$_6$** in acetonitrile-*h*$_3$.

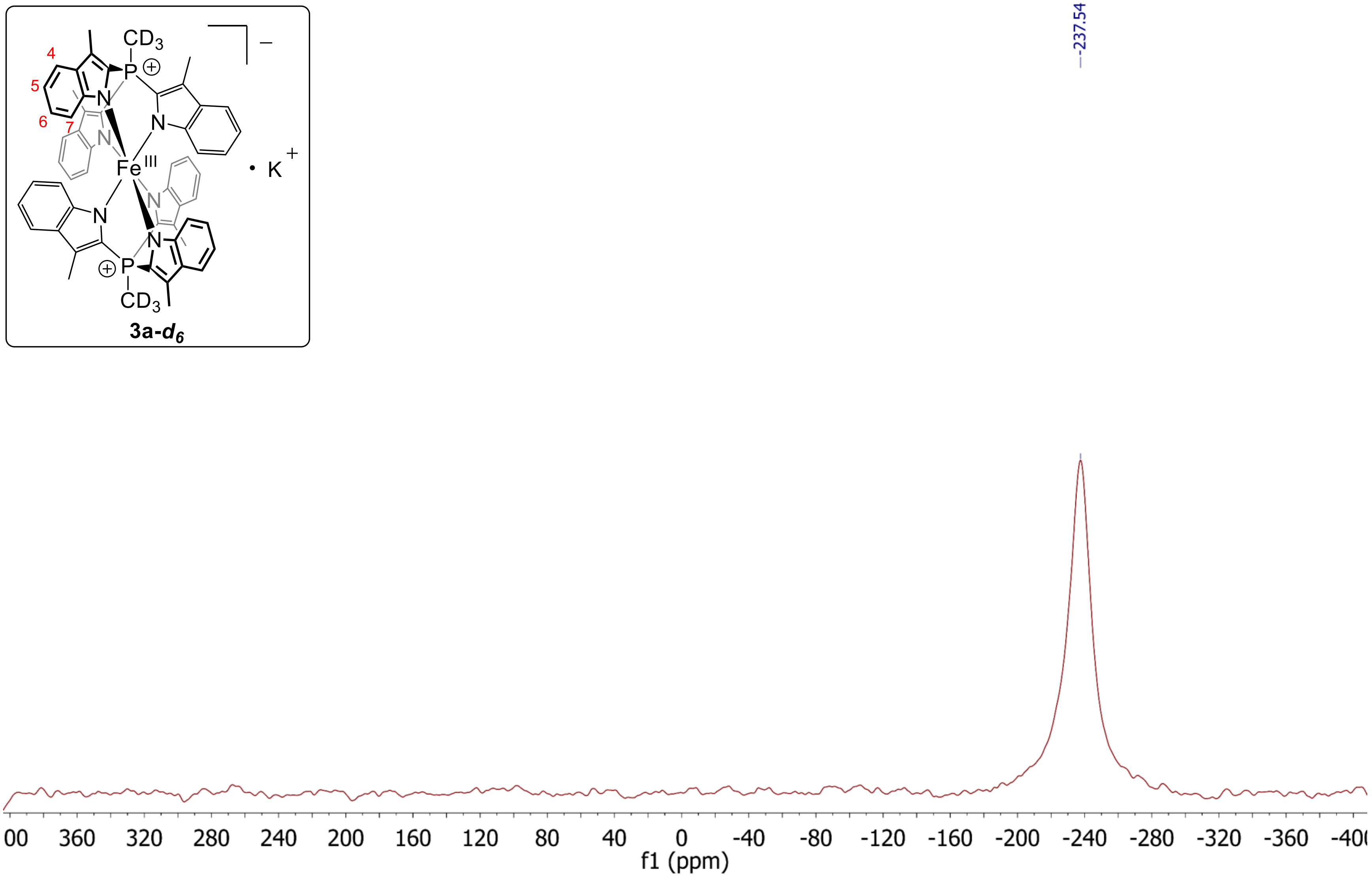

Figure S87. ^{31}P NMR (162 MHz) spectrum of compound **3a-*d*$_6$** in acetonitrile-*d*$_3$.

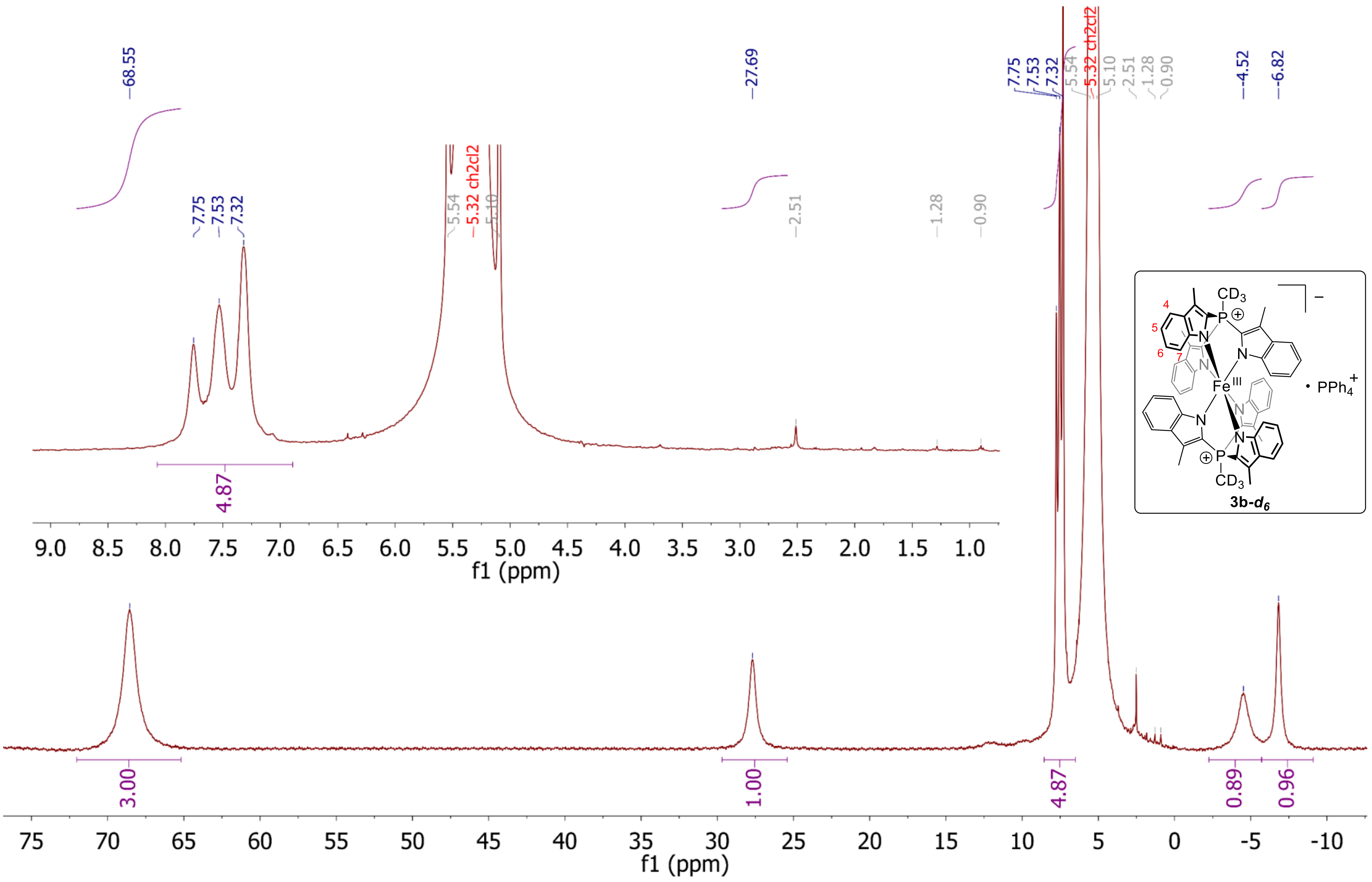

Figure S88. ^{1}H NMR (400 MHz) spectrum of compound **3b-*d*$_6$** in dichloromethane-*d*$_2$.

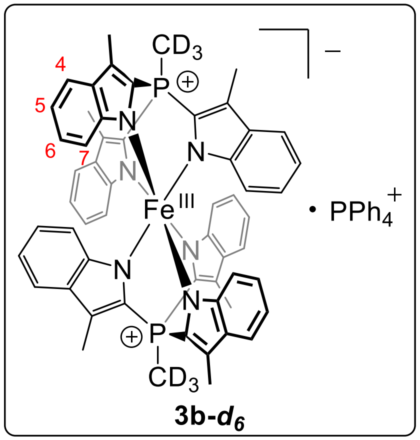

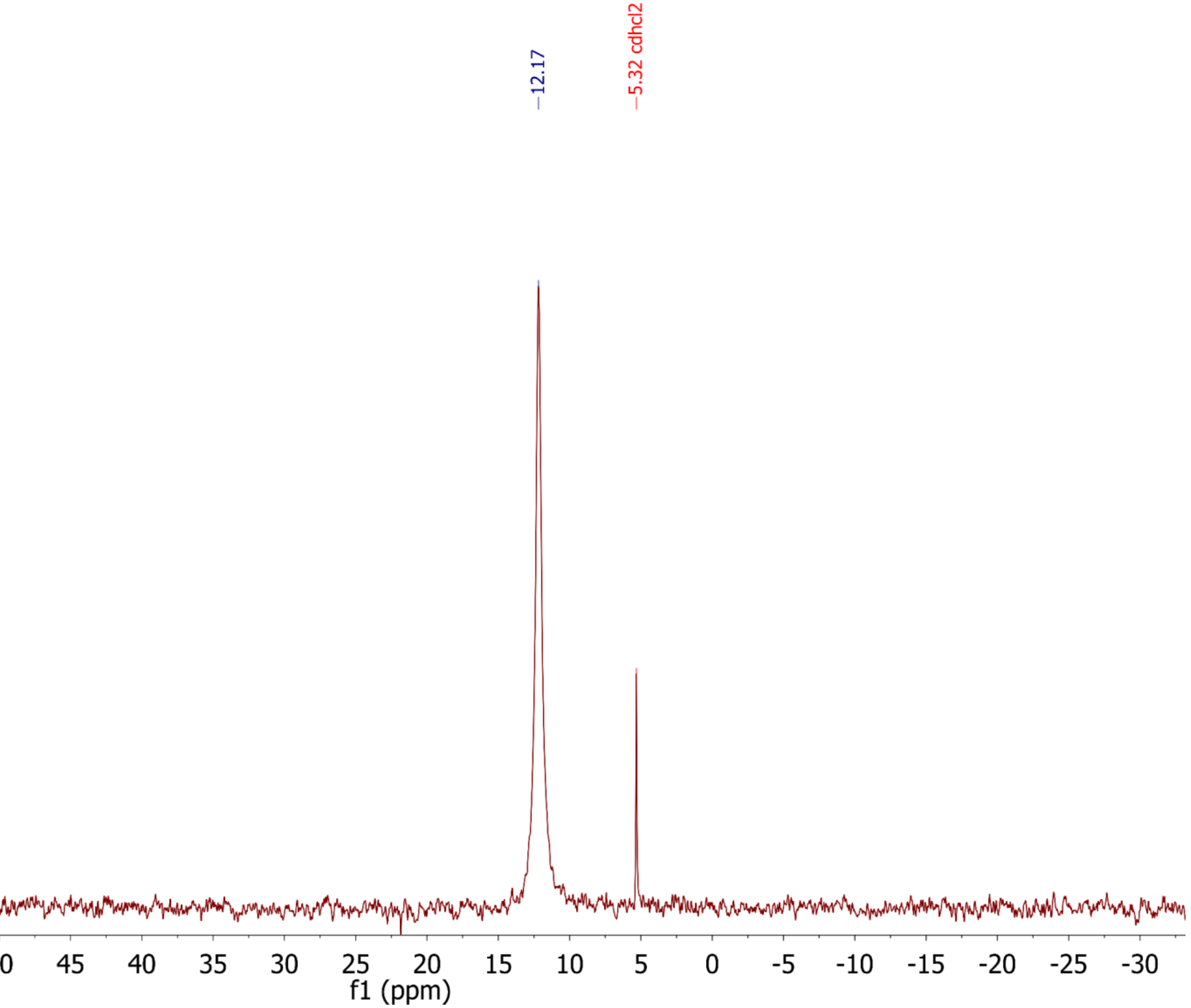

Figure S89. ²H NMR (61 MHz) spectrum of compound **3b-*d*$_6$** in dichloromethane-*h*$_2$.

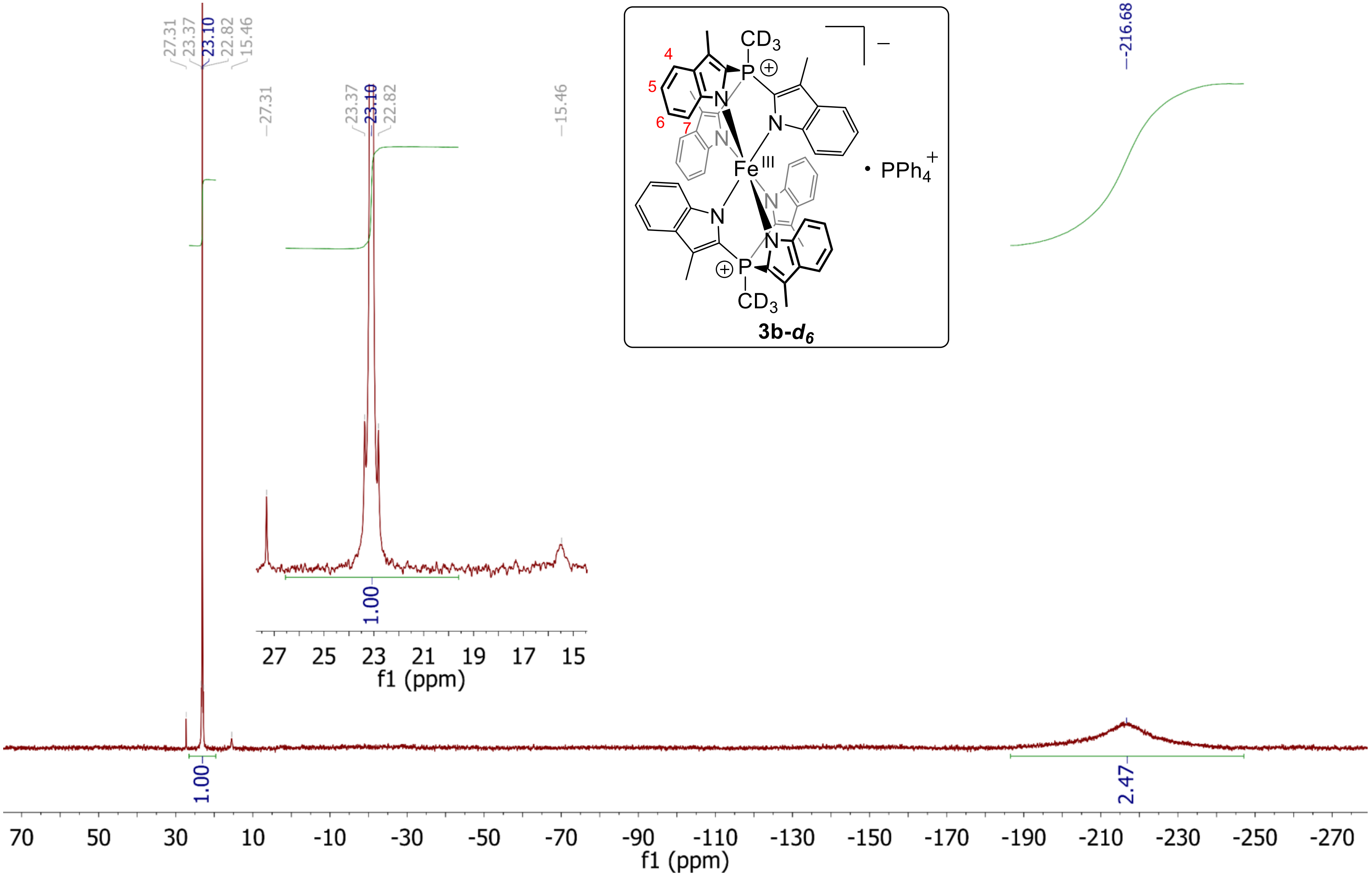

Figure S90. ^{31}P NMR (162 MHz) spectrum of compound **3b-*d*₆** in dichloromethane-*d*₂.

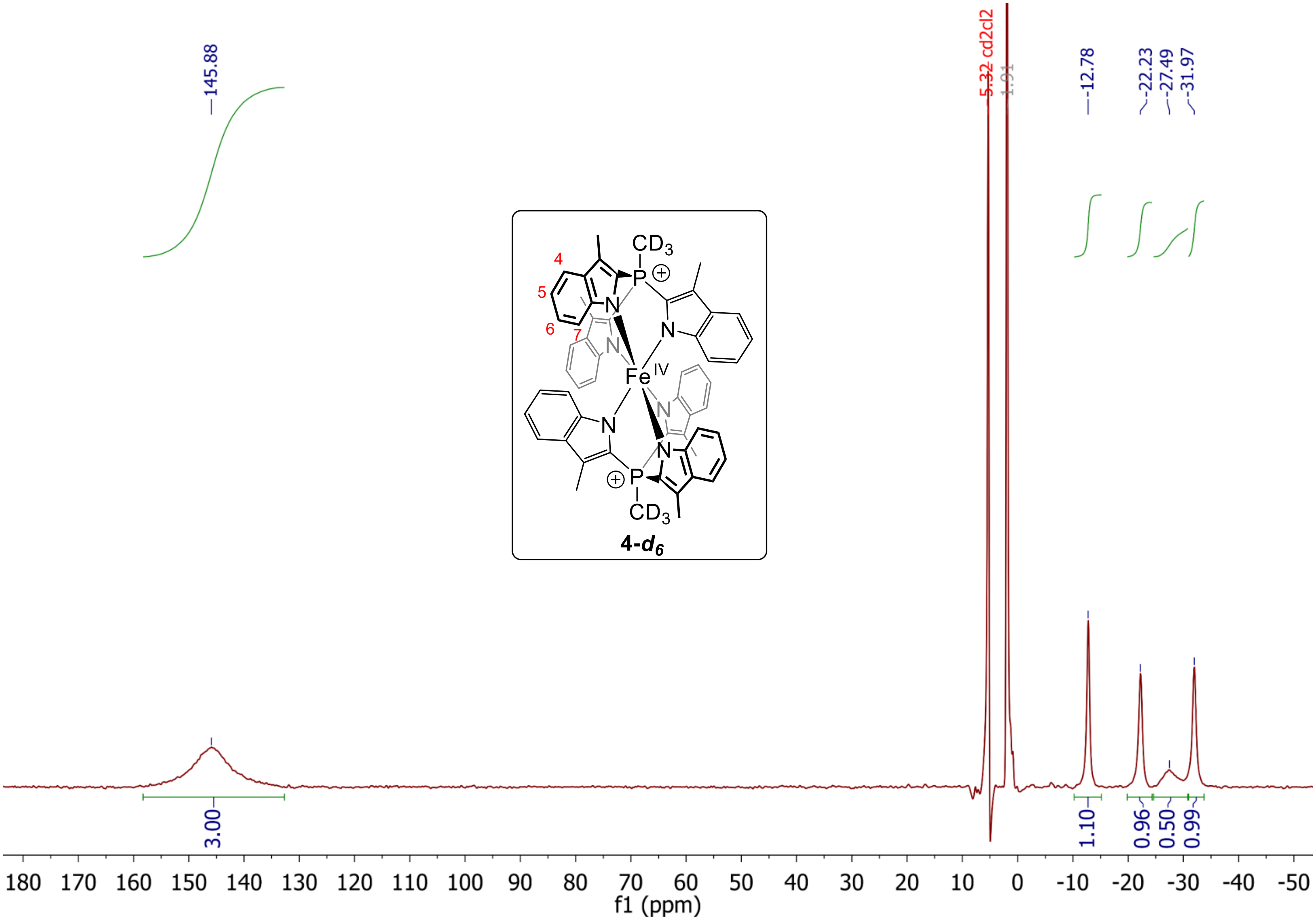

Figure S91. ^{1}H NMR (400 MHz) spectrum of compound **4-*d*$_6$** in dichloromethane-*d*$_2$.

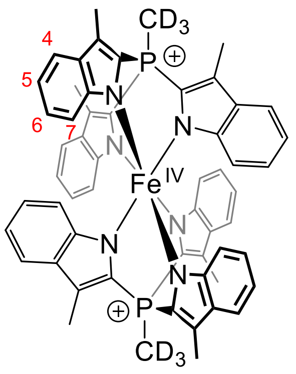

4-*d*$_6$

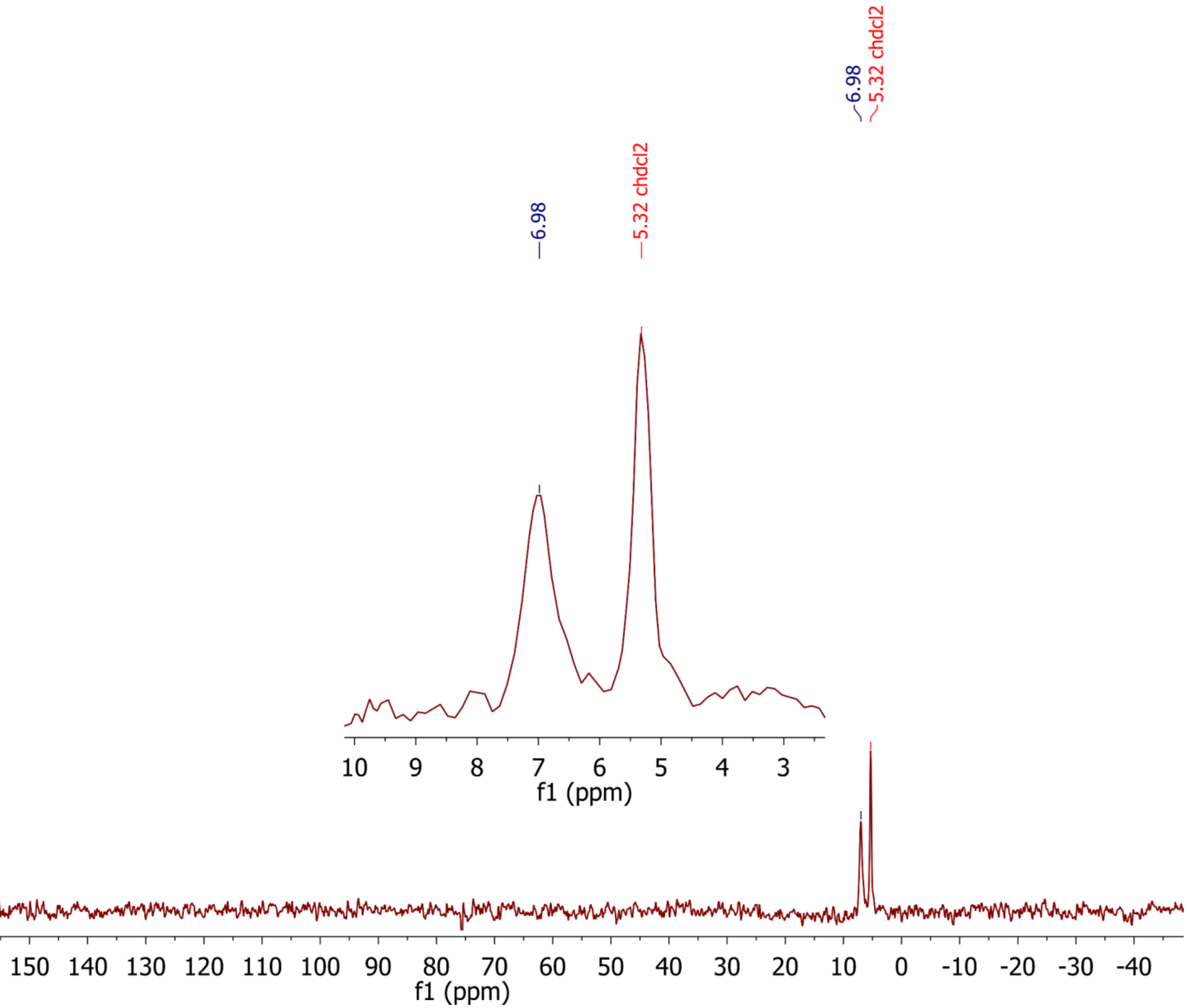

Figure S92. ^{2}H NMR (61 MHz) spectrum of compound **4-*d*$_6$** in dichloromethane-*h*$_2$.

S16 Computational coordinates

S16.1 [(TSMP)$_2$Fe^{III}]$^-$ (3)

Level of theory and spin state: B3LYP-D3BJ/def2-TZVP; S=1/2

Final single point energy: -4439.38431329 Hartree

C	-0.728229000	-2.445266000	-1.690316000
C	-1.892838000	-3.109417000	-2.187772000
C	-1.761877000	-4.266382000	-2.972779000
C	-0.499182000	-4.742702000	-3.253139000
C	0.644220000	-4.080173000	-2.760666000
C	0.548497000	-2.943206000	-1.987357000
C	-2.485636000	-1.322799000	-0.994447000
C	-3.023833000	-2.371321000	-1.730475000
H	-2.638410000	-4.778566000	-3.350953000
H	-0.378926000	-5.633616000	-3.856286000
H	1.624215000	-4.475314000	-2.995418000
H	1.431195000	-2.450430000	-1.617885000
P	-3.177239000	0.013022000	-0.080808000
C	-2.507251000	1.488656000	-0.757564000
C	-3.052837000	2.642257000	-1.315087000
C	-0.756410000	2.688810000	-1.305864000
C	-1.924644000	3.430020000	-1.678191000
C	0.521268000	3.222077000	-1.538919000
C	-1.792204000	4.693329000	-2.280546000
C	0.615890000	4.461958000	-2.130694000
H	1.405234000	2.673307000	-1.261531000
C	-0.530166000	5.198524000	-2.501949000
H	-2.668915000	5.260863000	-2.569021000
H	1.595571000	4.883264000	-2.316464000
H	-0.410112000	6.169607000	-2.965344000
C	-2.545409000	-0.125941000	1.555811000
C	-3.130664000	-0.232224000	2.811613000
C	-0.836839000	-0.229356000	2.935049000
C	-2.031729000	-0.295832000	3.717905000
C	0.418267000	-0.273821000	3.558672000
C	-1.952025000	-0.405851000	5.115628000
C	0.463494000	-0.381814000	4.932012000
H	1.323388000	-0.226668000	2.978168000
C	-0.709898000	-0.447961000	5.711555000
H	-2.851577000	-0.453983000	5.717418000
H	1.426432000	-0.417200000	5.425088000
H	-0.629121000	-0.532126000	6.787831000
N	-1.165697000	-0.123937000	1.609961000
N	-1.130197000	1.501326000	-0.743979000
N	-1.105519000	-1.350835000	-0.958811000
C	-4.578175000	-0.264687000	3.189086000
H	-5.145792000	-1.000292000	2.616079000
H	-4.683564000	-0.532378000	4.240079000
H	-5.068944000	0.704363000	3.056823000
C	-4.455782000	-2.711452000	-1.999764000

H	-4.518500000	-3.471089000	-2.778597000
H	-4.964676000	-3.115852000	-1.119303000
H	-5.033687000	-1.853162000	-2.346862000
C	-4.487685000	3.011838000	-1.523569000
H	-4.963793000	2.425594000	-2.315867000
H	-5.089406000	2.890148000	-0.620523000
H	-4.564493000	4.059037000	-1.815432000
C	-4.973879000	0.003520000	-0.117768000
H	-5.350116000	0.795361000	0.523547000
H	-5.312536000	0.170081000	-1.136300000
H	-5.339135000	-0.956346000	0.235570000
Fe	0.000000000	0.000000000	0.000000000
N	1.165697000	0.123937000	-1.609961000
C	0.836839000	0.229356000	-2.935049000
C	2.545409000	0.125941000	-1.555811000
C	2.031729000	0.295832000	-3.717905000
C	-0.418267000	0.273821000	-3.558672000
C	3.130664000	0.232224000	-2.811613000
P	3.177239000	-0.013022000	0.080808000
C	1.952025000	0.405851000	-5.115628000
C	-0.463494000	0.381814000	-4.932012000
H	-1.323388000	0.226668000	-2.978168000
C	4.578175000	0.264687000	-3.189086000
C	2.507251000	-1.488656000	0.757564000
C	2.485636000	1.322799000	0.994447000
C	4.973879000	-0.003520000	0.117768000
C	0.709898000	0.447961000	-5.711555000
H	2.851577000	0.453983000	-5.717418000
H	-1.426432000	0.417200000	-5.425088000
H	5.068944000	-0.704363000	-3.056823000
H	4.683564000	0.532378000	-4.240079000
H	5.145792000	1.000292000	-2.616079000
C	3.052837000	-2.642257000	1.315087000
N	1.130197000	-1.501326000	0.743979000
C	3.023833000	2.371321000	1.730475000
N	1.105519000	1.350835000	0.958811000
H	5.312536000	-0.170081000	1.136300000
H	5.350116000	-0.795361000	-0.523547000
H	5.339135000	0.956346000	-0.235570000
H	0.629121000	0.532126000	-6.787831000
C	1.924644000	-3.430020000	1.678191000
C	4.487685000	-3.011838000	1.523569000
C	0.756410000	-2.688810000	1.305864000
C	1.892838000	3.109417000	2.187772000
C	4.455782000	2.711452000	1.999764000
C	0.728229000	2.445266000	1.690316000
C	1.792204000	-4.693329000	2.280546000
H	4.564493000	-4.059037000	1.815432000
H	5.089406000	-2.890148000	0.620523000
H	4.963793000	-2.425594000	2.315867000
C	-0.521268000	-3.222077000	1.538919000
C	1.761877000	4.266382000	2.972779000

H	5.033687000	1.853162000	2.346862000
H	4.964676000	3.115852000	1.119303000
H	4.518500000	3.471089000	2.778597000
C	-0.548497000	2.943206000	1.987357000
C	0.530166000	-5.198524000	2.501949000
H	2.668915000	-5.260863000	2.569021000
C	-0.615890000	-4.461958000	2.130694000
H	-1.405234000	-2.673307000	1.261531000
C	0.499182000	4.742702000	3.253139000
H	2.638410000	4.778566000	3.350953000
C	-0.644220000	4.080173000	2.760666000
H	-1.431195000	2.450430000	1.617885000
H	0.410112000	-6.169607000	2.965344000
H	-1.595571000	-4.883264000	2.316464000
H	0.378926000	5.633616000	3.856286000
H	-1.624215000	4.475314000	2.995418000

Level of theory and spin state: B3LYP-D3BJ/def2-TZVP; S=5/2
Final single point energy: -4439.37884404 Hartree

C	-0.928734000	-2.507261000	-1.756586000
C	-2.117613000	-3.148115000	-2.223985000
C	-2.021069000	-4.301976000	-3.018876000
C	-0.770083000	-4.792472000	-3.331076000
C	0.396788000	-4.151523000	-2.863625000
C	0.335507000	-3.017504000	-2.081791000
C	-2.636315000	-1.355691000	-0.996706000
C	-3.219429000	-2.391444000	-1.723448000
H	-2.911291000	-4.800804000	-3.383035000
H	-0.677287000	-5.680872000	-3.942803000
H	1.363637000	-4.561108000	-3.126105000
H	1.228996000	-2.530177000	-1.726119000
P	-3.299836000	0.002264000	-0.077135000
C	-2.639046000	1.489762000	-0.770332000
C	-3.224373000	2.624130000	-1.328619000
C	-0.934242000	2.755190000	-1.329066000
C	-2.124402000	3.456181000	-1.695915000
C	0.328754000	3.315699000	-1.563937000
C	-2.030497000	4.721356000	-2.298570000
C	0.387446000	4.558829000	-2.157379000
H	1.223419000	2.782476000	-1.285295000
C	-0.780755000	5.260266000	-2.523758000
H	-2.921748000	5.267897000	-2.582985000
H	1.353276000	5.008813000	-2.347429000
H	-0.689957000	6.234315000	-2.987505000
C	-2.695137000	-0.129963000	1.580141000
C	-3.323520000	-0.233022000	2.819297000
C	-1.038094000	-0.249277000	3.015283000
C	-2.254958000	-0.311937000	3.762189000
C	0.203735000	-0.302421000	3.662985000
C	-2.209007000	-0.428981000	5.161045000
C	0.215278000	-0.417472000	5.036889000

H	1.118710000	-0.254053000	3.094799000
C	-0.979610000	-0.480794000	5.784652000
H	-3.121051000	-0.478644000	5.743834000
H	1.164133000	-0.460407000	5.555795000
H	-0.925751000	-0.571202000	6.862149000
N	-1.318327000	-0.138543000	1.683010000
N	-1.259926000	1.554884000	-0.764359000
N	-1.256905000	-1.413045000	-1.007758000
C	-4.781612000	-0.263492000	3.155630000
H	-5.294864000	-1.129074000	2.728368000
H	-4.912511000	-0.319442000	4.235788000
H	-5.311372000	0.630461000	2.817620000
C	-4.664166000	-2.697631000	-1.966514000
H	-4.756713000	-3.612704000	-2.550555000
H	-5.223693000	-2.853908000	-1.041071000
H	-5.173682000	-1.909837000	-2.527815000
C	-4.669582000	2.959492000	-1.526094000
H	-5.192135000	2.226184000	-2.145163000
H	-5.216486000	3.040462000	-0.582911000
H	-4.762178000	3.921481000	-2.029092000
C	-5.097022000	0.004913000	-0.119323000
H	-5.468865000	0.857913000	0.440171000
H	-5.432874000	0.071544000	-1.149679000
H	-5.469784000	-0.912456000	0.325980000
Fe	0.000000000	0.000000000	0.000000000
N	1.318327000	0.138543000	-1.683010000
C	1.038094000	0.249277000	-3.015283000
C	2.695137000	0.129963000	-1.580141000
C	2.254958000	0.311937000	-3.762189000
C	-0.203735000	0.302421000	-3.662985000
C	3.323520000	0.233022000	-2.819297000
P	3.299836000	-0.002264000	0.077135000
C	2.209007000	0.428981000	-5.161045000
C	-0.215278000	0.417472000	-5.036889000
H	-1.118710000	0.254053000	-3.094799000
C	4.781612000	0.263492000	-3.155630000
C	2.639046000	-1.489762000	0.770332000
C	2.636315000	1.355691000	0.996706000
C	5.097022000	-0.004913000	0.119323000
C	0.979610000	0.480794000	-5.784652000
H	3.121051000	0.478644000	-5.743834000
H	-1.164133000	0.460407000	-5.555795000
H	5.311372000	-0.630461000	-2.817620000
H	4.912511000	0.319442000	-4.235788000
H	5.294864000	1.129074000	-2.728368000
C	3.224373000	-2.624130000	1.328619000
N	1.259926000	-1.554884000	0.764359000
C	3.219429000	2.391444000	1.723448000
N	1.256905000	1.413045000	1.007758000
H	5.432874000	-0.071544000	1.149679000
H	5.468865000	-0.857913000	-0.440171000
H	5.469784000	0.912456000	-0.325980000

H	0.925751000	0.571202000	-6.862149000
C	2.124402000	-3.456181000	1.695915000
C	4.669582000	-2.959492000	1.526094000
C	0.934242000	-2.755190000	1.329066000
C	2.117613000	3.148115000	2.223985000
C	4.664166000	2.697631000	1.966514000
C	0.928734000	2.507261000	1.756586000
C	2.030497000	-4.721356000	2.298570000
H	4.762178000	-3.921481000	2.029092000
H	5.216486000	-3.040462000	0.582911000
H	5.192135000	-2.226184000	2.145163000
C	-0.328754000	-3.315699000	1.563937000
C	2.021069000	4.301976000	3.018876000
H	5.173682000	1.909837000	2.527815000
H	5.223693000	2.853908000	1.041071000
H	4.756713000	3.612704000	2.550555000
C	-0.335507000	3.017504000	2.081791000
C	0.780755000	-5.260266000	2.523758000
H	2.921748000	-5.267897000	2.582985000
C	-0.387446000	-4.558829000	2.157379000
H	-1.223419000	-2.782476000	1.285295000
C	0.770083000	4.792472000	3.331076000
H	2.911291000	4.800804000	3.383035000
C	-0.396788000	4.151523000	2.863625000
H	-1.228996000	2.530177000	1.726119000
H	0.689957000	-6.234315000	2.987505000
H	-1.353276000	-5.008813000	2.347429000
H	0.677287000	5.680872000	3.942803000
H	-1.363637000	4.561108000	3.126105000

S16.2 [(TSMP)$_2$Fe^{IV}]^0 (4)

Level of theory and spin state: BP86-D3BJ/def2-TZVP; S=0
Final single point energy: -4439.505935898992 Hartree

C	-0.649504000	-2.423221000	-1.665780000
C	-1.799378000	-3.100186000	-2.185571000
C	-1.643769000	-4.245294000	-2.982009000
C	-0.355630000	-4.702315000	-3.252635000
C	0.768899000	-4.029989000	-2.736044000
C	0.641632000	-2.893727000	-1.939863000
C	-2.453379000	-1.313503000	-0.990862000
C	-2.959608000	-2.378519000	-1.741346000
H	-2.513586000	-4.769991000	-3.381848000
H	-0.214326000	-5.591600000	-3.868048000
H	1.766575000	-4.411417000	-2.956922000
H	1.518395000	-2.398873000	-1.532563000
P	-3.161026000	0.033778000	-0.088373000
C	-2.483275000	1.504106000	-0.762576000
C	-3.004374000	2.680843000	-1.323312000
C	-0.691602000	2.678937000	-1.299123000
C	-1.851966000	3.445064000	-1.677261000

C	0.602058000	3.190205000	-1.519608000
C	-1.692079000	4.713383000	-2.276049000
C	0.722987000	4.438402000	-2.108842000
H	1.484531000	2.621784000	-1.236157000
C	-0.412010000	5.198657000	-2.486408000
H	-2.563803000	5.301062000	-2.571596000
H	1.718888000	4.847108000	-2.287279000
H	-0.270307000	6.176834000	-2.948233000
C	-2.510746000	-0.119439000	1.549253000
C	-3.065915000	-0.218154000	2.828625000
C	-0.755659000	-0.221948000	2.908393000
C	-1.937524000	-0.273387000	3.715893000
C	0.514921000	-0.269526000	3.496989000
C	-1.833543000	-0.361569000	5.112967000
C	0.590469000	-0.351958000	4.885749000
H	1.415635000	-0.257032000	2.890458000
C	-0.565537000	-0.398296000	5.688955000
H	-2.727427000	-0.397362000	5.738628000
H	1.571769000	-0.389986000	5.360343000
H	-0.464562000	-0.467329000	6.772724000
N	-1.120328000	-0.121615000	1.575441000
N	-1.095718000	1.490036000	-0.742384000
N	-1.063140000	-1.323376000	-0.931889000
C	-4.499667000	-0.234333000	3.248980000
H	-5.142005000	-0.773595000	2.540787000
H	-4.605711000	-0.727785000	4.224680000
H	-4.906983000	0.784870000	3.359290000
C	-4.374433000	-2.757778000	-2.033925000
H	-4.443365000	-3.250298000	-3.014195000
H	-4.767746000	-3.471320000	-1.290311000
H	-5.049532000	-1.893551000	-2.055778000
C	-4.429253000	3.080228000	-1.549448000
H	-4.845653000	2.650578000	-2.476444000
H	-5.089099000	2.784643000	-0.721655000
H	-4.506322000	4.172273000	-1.643153000
C	-4.960884000	0.020783000	-0.116881000
H	-5.335247000	0.803941000	0.551656000
H	-5.304240000	0.221336000	-1.137889000
H	-5.329537000	-0.956015000	0.214360000
Fe	0.000001000	-0.000075000	-0.000077000
N	1.120338000	0.120003000	-1.575698000
C	0.755682000	0.215370000	-2.909024000
C	2.510763000	0.118052000	-1.549468000
C	1.937580000	0.267697000	-3.716420000
C	-0.514894000	0.257535000	-3.498051000
C	3.065942000	0.215969000	-2.828891000
P	3.161033000	-0.033916000	0.088290000
C	1.833674000	0.352668000	-5.113696000
C	-0.590351000	0.336846000	-4.887000000
H	-1.415717000	0.243627000	-2.891716000
C	4.499710000	0.233014000	-3.249145000
C	2.483238000	-1.503687000	0.763680000

C	2.453399000	1.314079000	0.989737000
C	4.960891000	-0.020938000	0.116796000
C	0.565703000	0.384933000	-5.690028000
H	2.727598000	0.389096000	-5.739263000
H	-1.571643000	0.371075000	-5.361898000
H	4.908140000	-0.785930000	-3.357705000
H	4.605307000	0.724941000	-4.225664000
H	5.141397000	0.774154000	-2.541803000
C	3.004279000	-2.680118000	1.325127000
N	1.095684000	-1.489431000	0.743783000
C	2.959595000	2.379587000	1.739573000
N	1.063181000	1.324057000	0.930497000
H	5.304242000	-0.221101000	1.137882000
H	5.335243000	-0.804360000	-0.551438000
H	5.329558000	0.955727000	-0.214823000
H	0.464784000	0.451332000	-6.773967000
C	1.851806000	-3.442548000	1.682724000
C	4.429173000	-3.080070000	1.550155000
C	0.691461000	-2.675971000	1.305449000
C	1.799449000	3.103305000	2.180670000
C	4.374383000	2.758366000	2.032960000
C	0.649679000	2.426821000	1.660037000
C	1.691828000	-4.709267000	2.284865000
H	4.505623000	-4.171997000	1.645757000
H	5.088160000	-2.786448000	0.720986000
H	4.847048000	-2.649006000	2.475828000
C	-0.602295000	-3.184647000	1.531288000
C	1.643823000	4.250359000	2.974304000
H	5.048861000	1.893716000	2.056999000
H	4.768955000	3.470342000	1.288514000
H	4.442654000	3.252536000	3.012447000
C	-0.641297000	2.900513000	1.929300000
C	0.411679000	-5.192345000	2.499750000
H	2.563549000	-5.297281000	2.579754000
C	-0.723315000	-4.431267000	2.123834000
H	-1.484765000	-2.615589000	1.249097000
C	0.355789000	4.710009000	3.240954000
H	2.513557000	4.774724000	3.374756000
C	-0.768600000	4.038670000	2.722757000
H	-1.517868000	2.406426000	1.520642000
H	0.269907000	-6.169226000	2.964287000
H	-1.719281000	-4.838124000	2.306085000
H	0.214481000	5.600885000	3.854058000
H	-1.766162000	4.422340000	2.940242000

Level of theory and spin state: BP86-D3BJ/def2-TZVP; S=1
Final single point energy: -4439.527914116323 Hartree

C	-0.657872000	-2.430764000	-1.675842000
C	-1.811809000	-3.113886000	-2.184783000
C	-1.657847000	-4.268234000	-2.973589000
C	-0.374491000	-4.725327000	-3.246681000

C	0.755052000	-4.044432000	-2.743011000
C	0.633398000	-2.901738000	-1.961287000
C	-2.461515000	-1.332269000	-0.986595000
C	-2.969766000	-2.395493000	-1.738242000
H	-2.530432000	-4.796931000	-3.362146000
H	-0.234372000	-5.618938000	-3.856219000
H	1.751592000	-4.424100000	-2.972828000
H	1.514738000	-2.390890000	-1.583842000
P	-3.161543000	0.004146000	-0.072442000
C	-2.462380000	1.475088000	-0.748865000
C	-2.974069000	2.647912000	-1.311722000
C	-0.661053000	2.655606000	-1.291475000
C	-1.817378000	3.416181000	-1.669398000
C	0.629592000	3.162090000	-1.511534000
C	-1.665059000	4.679390000	-2.269402000
C	0.749081000	4.412549000	-2.106062000
H	1.511931000	2.595567000	-1.227351000
C	-0.382320000	5.168181000	-2.483469000
H	-2.538409000	5.264414000	-2.564296000
H	1.745087000	4.819822000	-2.285370000
H	-0.244027000	6.145433000	-2.947756000
C	-2.517023000	-0.133141000	1.563148000
C	-3.072945000	-0.233516000	2.841809000
C	-0.761222000	-0.216800000	2.920676000
C	-1.946023000	-0.284394000	3.726992000
C	0.509831000	-0.255676000	3.515366000
C	-1.841744000	-0.386384000	5.126141000
C	0.581778000	-0.356548000	4.899565000
H	1.413625000	-0.207363000	2.914521000
C	-0.577996000	-0.421341000	5.702175000
H	-2.737162000	-0.434289000	5.748931000
H	1.562046000	-0.386506000	5.377290000
H	-0.476896000	-0.499585000	6.785395000
N	-1.126515000	-0.120984000	1.591777000
N	-1.071708000	1.462463000	-0.729097000
N	-1.070937000	-1.338020000	-0.938096000
C	-4.508579000	-0.257105000	3.260465000
H	-5.147858000	-0.799357000	2.551379000
H	-4.612274000	-0.755730000	4.233928000
H	-4.923103000	0.758480000	3.375555000
C	-4.386762000	-2.774680000	-2.027406000
H	-4.458488000	-3.260310000	-3.010968000
H	-4.779372000	-3.492390000	-1.287585000
H	-5.062470000	-1.910525000	-2.044156000
C	-4.394030000	3.055633000	-1.544505000
H	-4.786028000	2.664613000	-2.498396000
H	-5.067527000	2.719796000	-0.745082000
H	-4.471913000	4.150460000	-1.592767000
C	-4.960786000	0.012848000	-0.109166000
H	-5.331199000	0.796102000	0.561300000
H	-5.300918000	0.212418000	-1.131257000
H	-5.335159000	-0.962092000	0.221569000

Fe	0.000001000	-0.000046000	0.000006000
N	1.126528000	0.119820000	-1.591832000
C	0.761269000	0.211862000	-2.920998000
C	2.517032000	0.132270000	-1.563175000
C	1.946060000	0.280329000	-3.727250000
C	-0.509765000	0.246419000	-3.515993000
C	3.072956000	0.232200000	-2.841874000
P	3.161543000	-0.004250000	0.072489000
C	1.841790000	0.379925000	-5.126572000
C	-0.581703000	0.344955000	-4.900361000
H	-1.413546000	0.196820000	-2.915228000
C	4.508597000	0.256674000	-3.260453000
C	2.462352000	-1.474857000	0.749615000
C	2.461521000	1.332619000	0.985994000
C	4.960786000	-0.012961000	0.109233000
C	0.578061000	0.411312000	-5.702859000
H	2.737206000	0.428499000	-5.749314000
H	-1.561952000	0.371860000	-5.378304000
H	4.924110000	-0.758650000	-3.374273000
H	4.611885000	0.754214000	-4.234515000
H	5.147297000	0.800404000	-2.551981000
C	2.974003000	-2.647492000	1.312910000
N	1.071679000	-1.462078000	0.730108000
C	2.969750000	2.396112000	1.737282000
N	1.070957000	1.338553000	0.937183000
H	5.300906000	-0.212687000	1.131298000
H	5.331206000	-0.796112000	-0.561349000
H	5.335165000	0.962029000	-0.221347000
H	0.476971000	0.487586000	-6.786220000
C	1.817266000	-3.414488000	1.673169000
C	4.393979000	-3.055616000	1.544893000
C	0.660951000	-2.653518000	1.296019000
C	1.811863000	3.116192000	2.181301000
C	4.386720000	2.774761000	2.027275000
C	0.657979000	2.433592000	1.671549000
C	1.664884000	-4.676599000	2.275468000
H	4.471376000	-4.150421000	1.594446000
H	5.066905000	-2.721100000	0.744440000
H	4.787039000	-2.663599000	2.497937000
C	-0.629766000	-3.158035000	1.520122000
C	1.657930000	4.272025000	2.967934000
H	5.061863000	1.910203000	2.045735000
H	4.780463000	3.491281000	1.286903000
H	4.457855000	3.261593000	3.010287000
C	-0.633184000	2.907210000	1.953066000
C	0.382086000	-5.163759000	2.492874000
H	2.538233000	-5.261920000	2.569773000
C	-0.749317000	-4.407423000	2.116882000
H	-1.512107000	-2.590920000	1.237118000
C	0.374665000	4.731303000	3.237773000
H	2.530481000	4.800324000	3.357111000
C	-0.754808000	4.051352000	2.732672000

H	-1.514466000	2.397153000	1.574413000
H	0.243743000	-6.140108000	2.959043000
H	-1.745372000	-4.813301000	2.299056000
H	0.234574000	5.626136000	3.845523000
H	-1.751270000	4.432899000	2.959701000

Level of theory and spin state: TPSSh-D3BJ/6-31G(d,p); S=1
Final single point energy: -4438.373643985770 Hartree

C	-0.658888000	-2.404439000	-1.691946000
C	-1.808637000	-3.100159000	-2.182815000
C	-1.651053000	-4.244457000	-2.988234000
C	-0.367882000	-4.673095000	-3.295764000
C	0.760957000	-3.980259000	-2.804274000
C	0.636761000	-2.852499000	-2.003360000
C	-2.460305000	-1.335036000	-0.976273000
C	-2.968849000	-2.402905000	-1.712601000
H	-2.518168000	-4.780486000	-3.364858000
H	-0.224735000	-5.550457000	-3.919425000
H	1.753346000	-4.338580000	-3.062462000
H	1.509294000	-2.331563000	-1.633050000
P	-3.161578000	0.006804000	-0.069041000
C	-2.449037000	1.473583000	-0.750684000
C	-2.952335000	2.620018000	-1.363304000
C	-0.644121000	2.637196000	-1.293409000
C	-1.789960000	3.379126000	-1.719516000
C	0.654338000	3.127922000	-1.515390000
C	-1.624315000	4.614856000	-2.374371000
C	0.786326000	4.344886000	-2.170239000
H	1.523385000	2.569803000	-1.193928000
C	-0.338226000	5.085411000	-2.597461000
H	-2.486663000	5.186594000	-2.706727000
H	1.781027000	4.736934000	-2.361947000
H	-0.189284000	6.032244000	-3.108126000
C	-2.515693000	-0.137389000	1.571207000
C	-3.054424000	-0.266604000	2.848864000
C	-0.745879000	-0.248766000	2.902233000
C	-1.916214000	-0.333716000	3.717068000
C	0.533870000	-0.298839000	3.481700000
C	-1.800743000	-0.464451000	5.113573000
C	0.618722000	-0.428228000	4.861792000
H	1.425499000	-0.238880000	2.872387000
C	-0.532650000	-0.510214000	5.675612000
H	-2.688045000	-0.527531000	5.737761000
H	1.599050000	-0.469382000	5.327555000
H	-0.419168000	-0.610608000	6.751013000
N	-1.122281000	-0.129394000	1.575870000
N	-1.059965000	1.459900000	-0.700507000
N	-1.070210000	-1.314635000	-0.945860000
C	-4.483732000	-0.318616000	3.295577000
H	-5.172864000	-0.543945000	2.478920000
H	-4.615769000	-1.094942000	4.056706000

H	-4.799063000	0.630026000	3.748856000
C	-4.396245000	-2.789719000	-1.969877000
H	-4.443741000	-3.558094000	-2.745471000
H	-4.881369000	-3.203700000	-1.076639000
H	-5.002998000	-1.946905000	-2.317715000
C	-4.375565000	3.003406000	-1.645038000
H	-4.820876000	2.389311000	-2.438013000
H	-5.015842000	2.919293000	-0.760416000
H	-4.425054000	4.041863000	-1.982597000
C	-4.967245000	0.024190000	-0.151109000
H	-5.352497000	0.830918000	0.473687000
H	-5.270551000	0.189307000	-1.185948000
H	-5.360309000	-0.932565000	0.194712000
Fe	0.000010000	0.000164000	-0.000064000
N	1.122304000	0.129704000	-1.575981000
C	0.745904000	0.248568000	-2.902391000
C	2.515713000	0.137837000	-1.571301000
C	1.916239000	0.333827000	-3.717194000
C	-0.533842000	0.297863000	-3.481933000
C	3.054442000	0.267207000	-2.848944000
P	3.161592000	-0.006408000	0.068950000
C	1.800772000	0.464273000	-5.113727000
C	-0.618688000	0.426972000	-4.862051000
H	-1.425471000	0.237561000	-2.872653000
C	4.483747000	0.319565000	-3.295625000
C	2.449072000	-1.473228000	0.750549000
C	2.460286000	1.335375000	0.976235000
C	4.967257000	-0.023755000	0.151048000
C	0.532684000	0.509383000	-5.675829000
H	2.688073000	0.527599000	-5.737892000
H	-1.599013000	0.467586000	-5.327868000
H	4.799320000	-0.629016000	-3.748862000
H	4.615610000	1.095895000	-4.056781000
H	5.172804000	0.545094000	-2.478961000
C	2.952375000	-2.619631000	1.363221000
N	1.060000000	-1.459593000	0.700309000
C	2.968794000	2.403189000	1.712667000
N	1.070195000	1.314924000	0.945824000
H	5.270546000	-0.188855000	1.185895000
H	5.352537000	-0.830482000	-0.473730000
H	5.360310000	0.933005000	-0.194774000
H	0.419204000	0.609532000	-6.751252000
C	1.790013000	-3.378890000	1.719163000
C	4.375595000	-3.002924000	1.645135000
C	0.644173000	-2.637058000	1.292891000
C	1.808556000	3.100136000	2.183272000
C	4.396176000	2.790088000	1.969890000
C	0.658831000	2.404349000	1.692442000
C	1.624383000	-4.614710000	2.373852000
H	4.425124000	-4.041424000	1.982556000
H	5.016010000	-2.918627000	0.760630000
H	4.820718000	-2.388900000	2.438271000

C	-0.654268000	-3.128032000	1.514430000
C	1.650932000	4.244148000	2.989089000
H	5.003026000	1.947271000	2.317552000
H	4.881199000	3.204249000	1.076680000
H	4.443666000	3.558346000	2.745600000
C	-0.636834000	2.851971000	2.004416000
C	0.338306000	-5.085474000	2.596576000
H	2.486731000	-5.186371000	2.706339000
C	-0.786246000	-4.345088000	2.169114000
H	-1.523314000	-2.570024000	1.192780000
C	0.367749000	4.672403000	3.297096000
H	2.518028000	4.780226000	3.365688000
C	-0.761067000	3.979442000	2.805727000
H	-1.509353000	2.330934000	1.634212000
H	0.189373000	-6.032386000	3.107098000
H	-1.780938000	-4.737312000	2.360511000
H	0.224572000	5.549526000	3.921087000
H	-1.753467000	4.337442000	3.064319000

Level of theory and spin state: B3LYP-D3BJ/6-31G(d,p); S=1
Final single point energy: -4436.391110743421

C	-0.663256000	-2.433439000	-1.668077000
C	-1.814657000	-3.122814000	-2.162311000
C	-1.665263000	-4.287257000	-2.937205000
C	-0.387446000	-4.747435000	-3.212359000
C	0.744297000	-4.059384000	-2.722762000
C	0.627070000	-2.909735000	-1.955840000
C	-2.462492000	-1.335279000	-0.983872000
C	-2.971021000	-2.401391000	-1.716341000
H	-2.537224000	-4.817729000	-3.310796000
H	-0.250929000	-5.645100000	-3.808421000
H	1.735550000	-4.440533000	-2.951848000
H	1.504365000	-2.397685000	-1.589300000
P	-3.181690000	-0.000262000	-0.077967000
C	-2.470712000	1.470406000	-0.752294000
C	-2.980672000	2.638326000	-1.306113000
C	-0.672452000	2.651063000	-1.288306000
C	-1.825209000	3.409858000	-1.661562000
C	0.616663000	3.164923000	-1.508526000
C	-1.679840000	4.676398000	-2.255535000
C	0.730682000	4.416977000	-2.093995000
H	1.495737000	2.602710000	-1.230737000
C	-0.402909000	5.172179000	-2.466368000
H	-2.553861000	5.253666000	-2.545683000
H	1.721226000	4.826933000	-2.270056000
H	-0.268634000	6.148011000	-2.923927000
C	-2.527514000	-0.133844000	1.557620000
C	-3.082959000	-0.235727000	2.826955000
C	-0.776267000	-0.219293000	2.913656000
C	-1.958422000	-0.288133000	3.715238000
C	0.492598000	-0.260938000	3.516020000

C	-1.862316000	-0.392635000	5.114411000
C	0.557728000	-0.365751000	4.897466000
H	1.393011000	-0.212645000	2.921944000
C	-0.605017000	-0.431396000	5.696106000
H	-2.758527000	-0.439899000	5.727472000
H	1.531804000	-0.398710000	5.377363000
H	-0.509087000	-0.511476000	6.775052000
N	-1.135931000	-0.121791000	1.581737000
N	-1.078947000	1.454109000	-0.727469000
N	-1.072041000	-1.332084000	-0.938399000
C	-4.523044000	-0.257842000	3.241256000
H	-5.159500000	-0.783529000	2.524081000
H	-4.632170000	-0.771055000	4.201329000
H	-4.932770000	0.752953000	3.370374000
C	-4.396628000	-2.779114000	-1.987199000
H	-4.450449000	-3.465931000	-2.836423000
H	-4.861821000	-3.289282000	-1.133121000
H	-5.020894000	-1.915504000	-2.236536000
C	-4.403581000	3.048338000	-1.536029000
H	-4.775534000	2.719004000	-2.515579000
H	-5.083591000	2.657157000	-0.774755000
H	-4.490796000	4.138929000	-1.512589000
C	-4.989836000	-0.010411000	-0.131079000
H	-5.378658000	0.757373000	0.538659000
H	-5.318949000	0.192046000	-1.151023000
H	-5.354740000	-0.989145000	0.182555000
Fe	0.000012000	-0.000106000	0.000000000
N	1.135962000	0.121929000	-1.581694000
C	0.776282000	0.220655000	-2.913514000
C	2.527542000	0.133866000	-1.557573000
C	1.958431000	0.289315000	-3.715115000
C	-0.492608000	0.263629000	-3.515748000
C	3.082980000	0.236022000	-2.826897000
P	3.181716000	0.000045000	0.077994000
C	1.862293000	0.394703000	-5.114220000
C	-0.557763000	0.369300000	-4.897124000
H	-1.392997000	0.215573000	-2.921627000
C	4.523057000	0.257936000	-3.241234000
C	2.470732000	-1.470736000	0.752072000
C	2.462519000	1.334955000	0.984069000
C	4.989862000	0.010175000	0.131086000
C	0.604976000	0.434588000	-5.695801000
H	2.758489000	0.441835000	-5.727312000
H	-1.531848000	0.403185000	-5.376937000
H	4.932527000	-0.752911000	-3.370754000
H	4.632276000	0.771485000	-4.201117000
H	5.159670000	0.783195000	-2.523883000
C	2.980694000	-2.638732000	1.305735000
N	1.078965000	-1.454475000	0.727152000
C	2.971028000	2.400954000	1.716719000
N	1.072065000	1.331748000	0.938598000
H	5.318982000	-0.192255000	1.151034000

H	5.378668000	-0.757633000	-0.538634000
H	5.354772000	0.988897000	-0.182575000
H	0.509026000	0.515384000	-6.774692000
C	1.825228000	-3.410459000	1.660752000
C	4.403598000	-3.048765000	1.535660000
C	0.672475000	-2.651685000	1.287434000
C	1.814638000	3.122109000	2.163067000
C	4.396627000	2.778758000	1.987511000
C	0.663250000	2.432680000	1.668884000
C	1.679864000	-4.677248000	2.254193000
H	4.490852000	-4.139345000	1.511886000
H	5.083664000	-2.657324000	0.774572000
H	4.775449000	-2.719731000	2.515349000
C	-0.616627000	-3.165854000	1.506983000
C	1.665211000	4.286321000	2.938300000
H	5.021032000	1.915143000	2.236483000
H	4.861643000	3.289263000	1.133537000
H	4.450478000	3.465303000	2.836954000
C	-0.627098000	2.908553000	1.957220000
C	0.402941000	-5.173304000	2.464436000
H	2.553883000	-5.254508000	2.544362000
C	-0.730645000	-4.418143000	2.091954000
H	-1.495705000	-2.603666000	1.229137000
C	0.387371000	4.746171000	3.213907000
H	2.537164000	4.816853000	3.311828000
C	-0.744361000	4.057969000	2.724491000
H	-1.504392000	2.396386000	1.590828000
H	0.268667000	-6.149329000	2.921581000
H	-1.721182000	-4.828312000	2.267548000
H	0.250826000	5.643640000	3.810255000
H	-1.735635000	4.438817000	2.953988000

Level of theory and spin state: PBE-D3BJ/6-31G(d,p); S=1
Final single point energy: -4434.372984896904 Hartree

C	-0.664134000	-2.436403000	-1.687564000
C	-1.823437000	-3.121508000	-2.193113000
C	-1.672726000	-4.280222000	-2.984794000
C	-0.387353000	-4.740104000	-3.265293000
C	0.748336000	-4.058370000	-2.764529000
C	0.630241000	-2.911628000	-1.979184000
C	-2.462576000	-1.341760000	-0.989447000
C	-2.981420000	-2.404858000	-1.739489000
H	-2.550829000	-4.809100000	-3.369772000
H	-0.251367000	-5.636655000	-3.877099000
H	1.746728000	-4.439498000	-2.998811000
H	1.513876000	-2.397695000	-1.601460000
P	-3.173068000	-0.000021000	-0.074708000
C	-2.470178000	1.477923000	-0.755142000
C	-2.993552000	2.647710000	-1.320512000
C	-0.675678000	2.666286000	-1.291182000

C	-1.838084000	3.422588000	-1.673430000
C	0.617000000	3.182644000	-1.512074000
C	-1.691842000	4.690248000	-2.276677000
C	0.730507000	4.438022000	-2.109378000
H	1.502751000	2.616541000	-1.224682000
C	-0.408128000	5.189127000	-2.489891000
H	-2.572014000	5.270099000	-2.573425000
H	1.727509000	4.851666000	-2.287768000
H	-0.275629000	6.169661000	-2.956367000
C	-2.522795000	-0.134778000	1.568550000
C	-3.089784000	-0.235198000	2.845385000
C	-0.773165000	-0.228570000	2.928522000
C	-1.963595000	-0.294180000	3.733362000
C	0.500010000	-0.271277000	3.531459000
C	-1.864771000	-0.398926000	5.137444000
C	0.566619000	-0.375604000	4.920836000
H	1.406974000	-0.223246000	2.929218000
C	-0.599889000	-0.439146000	5.721170000
H	-2.766583000	-0.445828000	5.756733000
H	1.548062000	-0.409035000	5.403109000
H	-0.504128000	-0.519866000	6.807869000
N	-1.129884000	-0.128385000	1.591221000
N	-1.077416000	1.465049000	-0.724727000
N	-1.070133000	-1.337424000	-0.943925000
C	-4.534997000	-0.259951000	3.244653000
H	-5.122931000	-0.993034000	2.666856000
H	-4.632130000	-0.537595000	4.305440000
H	-5.024357000	0.724116000	3.125545000
C	-4.410123000	-2.765836000	-2.016831000
H	-4.463506000	-3.519470000	-2.817553000
H	-4.916594000	-3.199586000	-1.135024000
H	-5.009346000	-1.901962000	-2.350054000
C	-4.424047000	3.038089000	-1.544088000
H	-4.897943000	2.462762000	-2.360496000
H	-5.046037000	2.905626000	-0.642768000
H	-4.489280000	4.100786000	-1.823809000
C	-4.985836000	0.000138000	-0.117346000
H	-5.364369000	0.791075000	0.544158000
H	-5.323818000	0.186502000	-1.145717000
H	-5.358002000	-0.975629000	0.222952000
Fe	0.000000000	0.000000000	0.000000000
N	1.129883000	0.128392000	-1.591220000
C	0.773164000	0.228602000	-2.928520000
C	2.522795000	0.134785000	-1.568550000
C	1.963594000	0.294209000	-3.733360000
C	-0.500010000	0.271334000	-3.531455000
C	3.089784000	0.235211000	-2.845385000
P	3.173068000	0.000022000	0.074707000
C	1.864770000	0.398972000	-5.137441000
C	-0.566620000	0.375678000	-4.920831000
H	-1.406974000	0.223309000	-2.929213000
C	4.534997000	0.259959000	-3.244654000

C	2.470178000	-1.477925000	0.755136000
C	2.462575000	1.341756000	0.989452000
C	4.985836000	-0.000136000	0.117346000
C	0.599888000	0.439213000	-5.721166000
H	2.766583000	0.445872000	-5.756730000
H	-1.548062000	0.409127000	-5.403102000
H	5.024352000	-0.724111000	-3.125555000
H	4.632130000	0.537613000	-4.305438000
H	5.122934000	0.993035000	-2.666850000
C	2.993553000	-2.647713000	1.320501000
N	1.077416000	-1.465052000	0.724720000
C	2.981419000	2.404852000	1.739498000
N	1.070132000	1.337420000	0.943930000
H	5.323817000	-0.186502000	1.145716000
H	5.364369000	-0.791072000	-0.544159000
H	5.358002000	0.975630000	-0.222951000
H	0.504127000	0.519947000	-6.807864000
C	1.838086000	-3.422600000	1.673403000
C	4.424048000	-3.038090000	1.544081000
C	0.675679000	-2.666300000	1.291153000
C	1.823436000	3.121492000	2.193138000
C	4.410123000	2.765832000	2.016838000
C	0.664133000	2.436385000	1.687591000
C	1.691844000	-4.690268000	2.276635000
H	4.489283000	-4.100789000	1.823795000
H	5.046041000	-2.905619000	0.642765000
H	4.897939000	-2.462768000	2.360495000
C	-0.616998000	-3.182671000	1.512020000
C	1.672724000	4.280195000	2.984834000
H	5.009349000	1.901955000	2.350049000
H	4.916588000	3.199592000	1.135033000
H	4.463507000	3.519458000	2.817567000
C	-0.630243000	2.911595000	1.979234000
C	0.408131000	-5.189156000	2.489829000
H	2.572016000	-5.270116000	2.573385000
C	-0.730504000	-4.438055000	2.109308000
H	-1.502750000	-2.616571000	1.224622000
C	0.387351000	4.740063000	3.265352000
H	2.550828000	4.809074000	3.369810000
C	-0.748338000	4.058326000	2.764594000
H	-1.513877000	2.397658000	1.601516000
H	0.275632000	-6.169696000	2.956292000
H	-1.727506000	-4.851709000	2.287681000
H	0.251366000	5.636606000	3.877171000
H	-1.746731000	4.439444000	2.998893000

Level of theory and spin state: PBE-D3BJ/def2-TZVP; S=1
Final single point energy: -4435.429766100296 Hartree

C	-0.677457000	-2.441804000	-1.689440000
C	-1.838810000	-3.117912000	-2.189838000
C	-1.697935000	-4.273590000	-2.979045000

C	-0.421423000	-4.740472000	-3.261347000
C	0.715855000	-4.068290000	-2.765699000
C	0.607475000	-2.924982000	-1.983779000
C	-2.463553000	-1.336568000	-0.987660000
C	-2.987232000	-2.395256000	-1.733813000
H	-2.576492000	-4.796440000	-3.361624000
H	-0.292632000	-5.635200000	-3.871326000
H	1.707897000	-4.455277000	-3.001848000
H	1.495763000	-2.421632000	-1.612250000
P	-3.165564000	-0.000009000	-0.074509000
C	-2.469894000	1.473340000	-0.751465000
C	-2.998206000	2.637622000	-1.314569000
C	-0.687819000	2.670361000	-1.294765000
C	-1.852303000	3.417630000	-1.671641000
C	0.595397000	3.193193000	-1.521039000
C	-1.716028000	4.681648000	-2.273382000
C	0.699131000	4.443938000	-2.117006000
H	1.486023000	2.638099000	-1.239771000
C	-0.441147000	5.185755000	-2.491722000
H	-2.596780000	5.256296000	-2.566036000
H	1.689819000	4.862161000	-2.299485000
H	-0.315954000	6.163831000	-2.957435000
C	-2.523542000	-0.135402000	1.563174000
C	-3.095111000	-0.235947000	2.833962000
C	-0.786578000	-0.226471000	2.933745000
C	-1.978697000	-0.293207000	3.728039000
C	0.476875000	-0.266665000	3.544727000
C	-1.889647000	-0.397440000	5.127894000
C	0.533882000	-0.370042000	4.929044000
H	1.388128000	-0.218031000	2.955057000
C	-0.633869000	-0.435050000	5.718384000
H	-2.791784000	-0.445861000	5.740586000
H	1.508769000	-0.401667000	5.417168000
H	-0.545248000	-0.515240000	6.802317000
N	-1.135523000	-0.128093000	1.600387000
N	-1.081536000	1.472798000	-0.729046000
N	-1.075493000	-1.345161000	-0.949094000
C	-4.536738000	-0.263254000	3.230597000
H	-5.123114000	-0.987462000	2.647358000
H	-4.633308000	-0.549180000	4.285718000
H	-5.020897000	0.720564000	3.120720000
C	-4.412440000	-2.753127000	-2.010966000
H	-4.465587000	-3.499405000	-2.813956000
H	-4.913025000	-3.192628000	-1.132975000
H	-5.010676000	-1.890097000	-2.335356000
C	-4.425240000	3.026650000	-1.534981000
H	-4.893789000	2.458064000	-2.354494000
H	-5.045635000	2.886653000	-0.638388000
H	-4.490201000	4.087987000	-1.806531000
C	-4.964593000	0.000421000	-0.116899000
H	-5.342366000	0.794369000	0.535814000
H	-5.302051000	0.177343000	-1.143532000

H	-5.335920000	-0.969364000	0.230613000
Fe	0.000000000	0.000007000	-0.000003000
N	1.135522000	0.128504000	-1.600364000
C	0.786571000	0.228243000	-2.933619000
C	2.523541000	0.135744000	-1.563155000
C	1.978692000	0.294740000	-3.727931000
C	-0.476888000	0.269952000	-3.544488000
C	3.095111000	0.236493000	-2.833925000
P	3.165564000	0.000032000	0.074500000
C	1.889638000	0.399888000	-5.127718000
C	-0.533898000	0.374222000	-4.928738000
H	-1.388143000	0.221701000	-2.954790000
C	4.536737000	0.263534000	-3.230582000
C	2.469905000	-1.473455000	0.751165000
C	2.463545000	1.336407000	0.987912000
C	4.964593000	-0.000394000	0.116890000
C	0.633855000	0.438753000	-5.718114000
H	2.791776000	0.448129000	-5.740424000
H	-1.508791000	0.406910000	-5.416782000
H	5.020592000	-0.720487000	-3.121183000
H	4.633372000	0.549929000	-4.285569000
H	5.123354000	0.987283000	-2.647014000
C	2.998230000	-2.637819000	1.314088000
N	1.081548000	-1.472956000	0.728676000
C	2.987224000	2.394990000	1.734210000
N	1.075482000	1.344950000	0.949422000
H	5.302052000	-0.177359000	1.143515000
H	5.342369000	-0.794312000	-0.535858000
H	5.335917000	0.969408000	-0.230579000
H	0.545230000	0.519692000	-6.801991000
C	1.852347000	-3.418274000	1.670244000
C	4.425260000	-3.026717000	1.534755000
C	0.687861000	-2.671126000	1.293133000
C	1.838776000	3.117048000	2.191115000
C	4.412438000	2.753039000	2.011100000
C	0.677414000	2.440773000	1.690964000
C	1.716097000	-4.682697000	2.271140000
H	4.490309000	-4.088169000	1.805831000
H	5.045905000	-2.886202000	0.638415000
H	4.893468000	-2.458438000	2.354675000
C	-0.595324000	-3.194646000	1.517991000
C	1.697882000	4.272185000	2.981111000
H	5.010959000	1.889986000	2.334904000
H	4.912663000	3.193061000	1.133164000
H	4.465667000	3.498953000	2.814423000
C	-0.607556000	2.923019000	1.986666000
C	0.441242000	-5.187374000	2.488308000
H	2.596849000	-5.257255000	2.563972000
C	-0.699033000	-4.445783000	2.113136000
H	-1.485947000	-2.639737000	1.236351000
C	0.421338000	4.738299000	3.264536000
H	2.576444000	4.795163000	3.363502000

C	-0.715955000	4.065802000	2.769349000
H	-1.495856000	2.419413000	1.615516000
H	0.316069000	-6.165781000	2.953331000
H	-1.689700000	-4.864491000	2.294619000
H	0.292530000	5.632585000	3.875159000
H	-1.708023000	4.452137000	3.006450000

Level of theory and spin state: BP86-D3BJ/def2-TZVP; S=2
Final single point energy: -4439.474810601670 Hartree

C	-0.705687000	-2.407390000	-1.710607000
C	-1.867711000	-3.013887000	-2.288810000
C	-1.732669000	-4.132546000	-3.129809000
C	-0.458565000	-4.626912000	-3.385238000
C	0.679187000	-4.018238000	-2.814414000
C	0.575046000	-2.911602000	-1.978496000
C	-2.482274000	-1.248568000	-1.038905000
C	-3.007854000	-2.258291000	-1.853228000
H	-2.609821000	-4.603725000	-3.577567000
H	-0.333767000	-5.493701000	-4.035414000
H	1.667264000	-4.425372000	-3.032706000
H	1.458612000	-2.452898000	-1.543321000
P	-3.212289000	0.060161000	-0.092204000
C	-2.618772000	1.587530000	-0.758295000
C	-3.200711000	2.728630000	-1.341716000
C	-0.894520000	2.804781000	-1.382093000
C	-2.081199000	3.527586000	-1.749155000
C	0.381296000	3.332469000	-1.633618000
C	-1.968976000	4.782191000	-2.376350000
C	0.460802000	4.572562000	-2.259750000
H	1.275014000	2.781076000	-1.347157000
C	-0.698240000	5.291298000	-2.627735000
H	-2.857670000	5.348691000	-2.662682000
H	1.441233000	5.001212000	-2.472193000
H	-0.592070000	6.261123000	-3.115734000
C	-2.544281000	-0.118898000	1.536499000
C	-3.116732000	-0.124626000	2.813520000
C	-0.809117000	-0.197358000	2.924805000
C	-2.004180000	-0.183246000	3.716801000
C	0.454193000	-0.263904000	3.533387000
C	-1.917461000	-0.229185000	5.120618000
C	0.508723000	-0.302545000	4.920927000
H	1.362119000	-0.286176000	2.936780000
C	-0.661761000	-0.284909000	5.711409000
H	-2.819245000	-0.217982000	5.736010000
H	1.481822000	-0.349363000	5.411620000
H	-0.574750000	-0.317202000	6.798213000
N	-1.153605000	-0.156384000	1.587930000
N	-1.239188000	1.620064000	-0.776551000
N	-1.097176000	-1.322288000	-0.946965000
C	-4.556783000	-0.053638000	3.214943000
H	-5.186488000	-0.765516000	2.662748000

H	-4.662739000	-0.291629000	4.281439000
H	-4.982419000	0.952868000	3.068849000
C	-4.429469000	-2.551612000	-2.213382000
H	-4.468233000	-3.205089000	-3.094950000
H	-4.966034000	-3.073919000	-1.404085000
H	-4.998976000	-1.644883000	-2.459159000
C	-4.641924000	3.066147000	-1.550453000
H	-5.043317000	2.620792000	-2.476792000
H	-5.278197000	2.731336000	-0.719805000
H	-4.771505000	4.153669000	-1.639993000
C	-5.010471000	-0.031462000	-0.094164000
H	-5.409343000	0.729270000	0.585495000
H	-5.380283000	0.156651000	-1.107743000
H	-5.328620000	-1.025695000	0.237590000
Fe	0.000004000	0.000003000	0.000024000
N	1.153603000	0.156376000	-1.587892000
C	0.809106000	0.197198000	-2.924771000
C	2.544279000	0.118830000	-1.536470000
C	2.004163000	0.182966000	-3.716774000
C	-0.454207000	0.263716000	-3.533350000
C	3.116719000	0.124405000	-2.813495000
P	3.212289000	-0.060164000	0.092240000
C	1.917434000	0.228761000	-5.120595000
C	-0.508747000	0.302209000	-4.920893000
H	-1.362125000	0.286074000	-2.936734000
C	4.556765000	0.053319000	-3.214920000
C	2.618770000	-1.587502000	0.758396000
C	2.482283000	1.248607000	1.038888000
C	5.010472000	0.031451000	0.094188000
C	0.661730000	0.284457000	-5.711382000
H	2.819212000	0.217463000	-5.735994000
H	-1.481848000	0.349003000	-5.411585000
H	4.982375000	-0.953179000	-3.068697000
H	4.662720000	0.291165000	-4.281448000
H	5.186494000	0.765252000	-2.662823000
C	3.200713000	-2.728574000	1.341868000
N	1.239184000	-1.620043000	0.776647000
C	3.007856000	2.258355000	1.853187000
N	1.097183000	1.322313000	0.946962000
H	5.380291000	-0.156620000	1.107772000
H	5.409336000	-0.729314000	-0.585440000
H	5.328623000	1.025667000	-0.237614000
H	0.574712000	0.316636000	-6.798190000
C	2.081202000	-3.527512000	1.749350000
C	4.641926000	-3.066072000	1.550638000
C	0.894522000	-2.804725000	1.382258000
C	1.867722000	3.014047000	2.288626000
C	4.429465000	2.551664000	2.213378000
C	0.705705000	2.407563000	1.710398000
C	1.968982000	-4.782081000	2.376617000
H	4.771513000	-4.153590000	1.640223000
H	5.278208000	-2.731290000	0.719986000

H	5.043303000	-2.620677000	2.476964000
C	-0.381294000	-3.332395000	1.633824000
C	1.732683000	4.132806000	3.129494000
H	4.998942000	1.644939000	2.459234000
H	4.966072000	3.073908000	1.404068000
H	4.468211000	3.205197000	3.094906000
C	-0.575012000	2.911930000	1.978067000
C	0.698247000	-5.291170000	2.628042000
H	2.857677000	-5.348565000	2.662978000
C	-0.460798000	-4.572452000	2.260028000
H	-1.275015000	-2.781018000	1.347340000
C	0.458591000	4.627293000	3.384749000
H	2.609828000	4.603980000	3.577269000
C	-0.679150000	4.018660000	2.813857000
H	-1.458566000	2.453254000	1.542834000
H	0.592078000	-6.260967000	3.116098000
H	-1.441228000	-5.001086000	2.472504000
H	0.333795000	5.494162000	4.034818000
H	-1.667215000	4.425902000	3.032002000

S16.3 Si-tethered analogue of [(TSMP)$_2$Fe^{IV}]^0 (4)

Level of theory and spin state: BP86-D3BJ/def2-TZVP; S=1
Final single point energy: -4335.935498098608 Hartree

C	-0.603766000	-2.452189000	-1.668764000
C	-1.731395000	-3.174364000	-2.188733000
C	-1.535563000	-4.328053000	-2.966093000
C	-0.235025000	-4.757860000	-3.222854000
C	0.866526000	-4.042905000	-2.708569000
C	0.700031000	-2.896108000	-1.935109000
C	-2.458974000	-1.390855000	-0.998506000
C	-2.905467000	-2.479702000	-1.748950000
H	-2.392782000	-4.879399000	-3.363313000
H	-0.064378000	-5.653398000	-3.825879000
H	1.879451000	-4.392944000	-2.919006000
H	1.563102000	-2.360801000	-1.549668000
Si	-3.254064000	0.004633000	-0.074275000
C	-2.459899000	1.516813000	-0.793123000
C	-2.906491000	2.703205000	-1.377146000
C	-0.604571000	2.659473000	-1.312347000
C	-1.732332000	3.451205000	-1.718260000
C	0.699414000	3.133845000	-1.518379000
C	-1.536307000	4.704002000	-2.323059000
C	0.866056000	4.379243000	-2.120257000
H	1.562421000	2.546918000	-1.217048000
C	-0.235563000	5.163097000	-2.521583000
H	-2.393459000	5.308806000	-2.633048000
H	1.879123000	4.753423000	-2.283104000
H	-0.064819000	6.135501000	-2.990655000
C	-2.517347000	-0.114736000	1.621682000
C	-3.009320000	-0.210366000	2.924336000

C	-0.706133000	-0.204455000	2.936414000
C	-1.864006000	-0.268705000	3.784169000
C	0.579434000	-0.241025000	3.496559000
C	-1.715968000	-0.368911000	5.177637000
C	0.698409000	-0.340533000	4.881117000
H	1.464664000	-0.193059000	2.868805000
C	-0.433022000	-0.404460000	5.720672000
H	-2.596167000	-0.418213000	5.825257000
H	1.696990000	-0.369298000	5.322514000
H	-0.299518000	-0.482255000	6.802741000
N	-1.122059000	-0.110882000	1.619317000
N	-1.065669000	1.481903000	-0.749239000
N	-1.064865000	-1.366334000	-0.944420000
C	-4.432655000	-0.249169000	3.382103000
H	-5.131626000	-0.195417000	2.538005000
H	-4.655381000	-1.176300000	3.940144000
H	-4.665412000	0.590516000	4.061375000
C	-4.311345000	-2.882354000	-2.062580000
H	-4.500507000	-2.886994000	-3.151146000
H	-4.532603000	-3.902338000	-1.700413000
H	-5.040320000	-2.202878000	-1.603465000
C	-4.312829000	3.150582000	-1.620206000
H	-4.506543000	3.320871000	-2.694523000
H	-5.041105000	2.410248000	-1.266006000
H	-4.530881000	4.104033000	-1.106413000
C	-5.139129000	0.006491000	-0.118856000
H	-5.540684000	0.855700000	0.452085000
H	-5.501582000	0.084766000	-1.153725000
H	-5.538130000	-0.920685000	0.316560000
Fe	-0.000006000	-0.000081000	0.000186000
N	1.122066000	0.110735000	-1.618928000
C	0.706151000	0.204502000	-2.936008000
C	2.517357000	0.114537000	-1.621275000
C	1.864034000	0.268629000	-3.783762000
C	-0.579415000	0.241372000	-3.496138000
C	3.009343000	0.210080000	-2.923930000
Si	3.254050000	-0.004805000	0.074701000
C	1.716005000	0.368945000	-5.177222000
C	-0.698379000	0.340983000	-4.880692000
H	-1.464651000	0.193546000	-2.868382000
C	4.432684000	0.248732000	-3.381690000
C	2.459874000	-1.516962000	0.793573000
C	2.458953000	1.390708000	0.998883000
C	5.139115000	-0.006665000	0.119321000
C	0.433060000	0.404741000	-5.720249000
H	2.596210000	0.418145000	-5.824843000
H	-1.696957000	0.369951000	-5.322083000
H	4.665365000	-0.590994000	-4.060941000
H	4.655505000	1.175827000	-3.939753000
H	5.131643000	0.194933000	-2.537585000
C	2.906458000	-2.703282000	1.377751000
N	1.065649000	-1.482099000	0.749569000

C	2.905442000	2.479526000	1.749368000
N	1.064847000	1.366222000	0.944732000
H	5.501548000	-0.084918000	1.154198000
H	5.540681000	-0.855885000	-0.451597000
H	5.538124000	0.920501000	-0.316107000
H	0.299563000	0.482620000	-6.802313000
C	1.732305000	-3.451495000	1.718414000
C	4.312786000	-3.150511000	1.621133000
C	0.604557000	-2.659902000	1.312198000
C	1.731387000	3.174594000	2.188550000
C	4.311312000	2.881993000	2.063274000
C	0.603766000	2.452609000	1.668297000
C	1.536279000	-4.704411000	2.322966000
H	4.531044000	-4.103957000	1.107423000
H	5.041067000	-2.410119000	1.267063000
H	4.506282000	-3.320745000	2.695500000
C	-0.699417000	-3.134623000	1.517497000
C	1.535567000	4.328585000	2.965464000
H	5.040278000	2.202191000	1.604631000
H	4.532899000	3.901794000	1.700793000
H	4.500147000	2.886999000	3.151896000
C	-0.700004000	2.897198000	1.933658000
C	0.235544000	-5.163788000	2.520890000
H	2.393423000	-5.309100000	2.633200000
C	-0.866062000	-4.380133000	2.119138000
H	-1.562412000	-2.547862000	1.215815000
C	0.235051000	4.758933000	3.221428000
H	2.392779000	4.879784000	3.362903000
C	-0.866486000	4.044282000	2.706693000
H	-1.563066000	2.362150000	1.547837000
H	0.064798000	-6.136286000	2.989765000
H	-1.879121000	-4.754553000	2.281478000
H	0.064415000	5.654720000	3.824086000
H	-1.879393000	4.394783000	2.916449000

S16.4 3-methylindole

Level of theory: TPSSh-D3BJ/6-31G(d,p)
Final single point energy: -403.178894910368 Hartree

C	-5.093719000	-0.375368000	0.114487000
C	-4.052357000	-1.116828000	-0.514605000
C	-2.835799000	-0.533416000	-0.887559000
C	-2.664774000	0.820364000	-0.612590000
C	-3.676762000	1.573053000	0.019694000
C	-4.886442000	0.989876000	0.382442000
C	-5.787445000	-2.504989000	-0.132148000
H	-2.054953000	-1.112220000	-1.372430000
H	-1.734286000	1.306285000	-0.890564000
H	-3.502964000	2.625240000	0.224921000
H	-5.659927000	1.579030000	0.866886000

H	-6.316118000	-3.447656000	-0.146897000
C	-7.517572000	-0.945107000	0.957596000
H	-8.042447000	-0.177923000	0.376190000
H	-7.405579000	-0.554775000	1.976621000
H	-8.162526000	-1.827261000	1.004917000
C	-6.192383000	-1.281045000	0.343576000
N	-4.504535000	-2.413367000	-0.648734000
H	-3.987157000	-3.173165000	-1.060144000

S17 Literature references

(1) Fulmer, G. R.; Miller, A. J. M.; Sherden, N. H.; Gottlieb, H. E.; Nudelman, A.; Stoltz, B. M.; Bercaw, J. E.; Goldberg, K. I. NMR Chemical Shifts of Trace Impurities: Common Laboratory Solvents, Organics, and Gases in Deuterated Solvents Relevant to the Organometallic Chemist. *Organometallics* **2010**, *29* (9), pp 2176–2179. DOI: 10.1021/om100106e.

(2) MestReNova v.9.0.1-13254. Mestrelab Research S.L. 2014.

(3) Kupče, E.; Freeman, R. Fast Multidimensional NMR by Polarization Sharing. *Magn. Reson. Chem.* **2007**, *45* (1), pp 2–4. DOI: 10.1002/mrc.1931.

(4) VnmrJ 4.2, Revision A. Agilent Technologies: Santa Clara, USA 2014.

(5) Tretiakov, S.; Damen, J. A. M.; Lutz, M.; Moret, M.-E. A Dianionic C_3-Symmetric Scorpionate: Synthesis and Coordination Chemistry. *Dalt. Trans.* **2020**, *49* (39), pp 13549–13556. DOI: 10.1039/D0DT02601H.

(6) Evans, D. F. 400. The Determination of the Paramagnetic Susceptibility of Substances in Solution by Nuclear Magnetic Resonance. *J. Chem. Soc.* **1959**, pp 2003–2005. DOI: 10.1039/jr9590002003.

(7) Piguet, C. Paramagnetic Susceptibility by NMR: The "Solvent Correction" Removed for Large Paramagnetic Molecules. *J. Chem. Educ.* **1997**, *74* (7), p 815. DOI: 10.1021/ed074p815.

(8) Sur, S. K. Measurement of Magnetic Susceptibility and Magnetic Moment of Paramagnetic Molecules in Solution by High-Field Fourier Transform NMR Spectroscopy. *J. Magn. Reson.* **1989**, *82* (1), pp 169–173. DOI: 10.1016/0022-2364(89)90178-9.

(9) Yatsunyk, L. A.; Walker, F. A. Structural, NMR, and EPR Studies of S = 1/2 and S = 3/2 Fe(III) Bis(4-Cyanopyridine) Complexes of Dodecasubstituted Porphyrins. *Inorg. Chem.* **2004**, *43* (2), pp 757–777. DOI: 10.1021/ic035010q.

(10) Schreurs, A. M. M.; Xian, X.; Kroon-Batenburg, L. M. J. EVAL15 : A Diffraction Data Integration Method Based on Ab Initio Predicted Profiles. *J. Appl. Crystallogr.* **2010**, *43* (1), pp 70–82. DOI: 10.1107/S0021889809043234.

(11) Herbst-Irmer, R.; Sheldrick, G. M. Refinement of Twinned Structures with SHELXL97. *Acta Crystallogr. Sect. B Struct. Sci.* **1998**, *54* (4), pp 443–449. DOI: 10.1107/S0108768197018454.

(12) Sheldrick, G. M. SADABS. Universität Göttingen, Germany 2014.

(13) Sheldrick, G. M. TWINABS. Universität Göttingen, Germany 2014.

(14) Sheldrick, G. M. SHELXT – Integrated Space-Group and Crystal-Structure Determination. *Acta Crystallogr. Sect. A Found. Adv.* **2015**, *71* (1), pp 3–8. DOI:

10.1107/S2053273314026370.

(15) Sheldrick, G. M. Crystal Structure Refinement with SHELXL. *Acta Crystallogr. Sect. C Struct. Chem.* **2015**, *71* (1), pp 3–8. DOI: 10.1107/S2053229614024218.

(16) Spek, A. L. Structure Validation in Chemical Crystallography. *Acta Crystallogr. Sect. D Biol. Crystallogr.* **2009**, *65* (2), pp 148–155. DOI: 10.1107/S090744490804362X.

(17) Spek, A. L. PLATON SQUEEZE: A Tool for the Calculation of the Disordered Solvent Contribution to the Calculated Structure Factors. *Acta Crystallogr. Sect. C Struct. Chem.* **2015**, *71* (1), pp 9–18. DOI: 10.1107/S2053229614024929.

(18) Neese, F. The ORCA Program System. *WIREs Comput. Mol. Sci.* **2012**, *2* (1), pp 73–78. DOI: 10.1002/wcms.81.

(19) Neese, F. Software Update: The ORCA Program System, Version 4.0. *WIREs Comput. Mol. Sci.* **2018**, *8* (1). DOI: 10.1002/wcms.1327.

(20) Römelt, M.; Ye, S.; Neese, F. Calibration of Modern Density Functional Theory Methods for the Prediction of ^{57}Fe Mössbauer Isomer Shifts: Meta-GGA and Double-Hybrid Functionals. *Inorg. Chem.* **2009**, *48* (3), pp 784–785. DOI: 10.1021/ic801535v.

(21) Savéant, J.-M. *Elements of Molecular and Biomolecular Electrochemistry: An Electrochemical Approach to Electron Transfer Chemistry*; John Wiley & Sons: Hoboken, NJ, 2006.

(22) Bain, G. A.; Berry, J. F. Diamagnetic Corrections and Pascal's Constants. *J. Chem. Educ.* **2008**, *85* (4), p 532. DOI: 10.1021/ed085p532.

(23) Lee, S.-J.; Titus, C. J.; Alonso Mori, R.; Baker, M. L.; Bennett, D. A.; Cho, H.-M.; Doriese, W. B.; Fowler, J. W.; Gaffney, K. J.; Gallo, A.; Gard, J. D.; Hilton, G. C.; Jang, H.; Joe, Y. Il; Kenney, C. J.; Knight, J.; Kroll, T.; Lee, J.-S.; Li, D.; Lu, D.; Marks, R.; Minitti, M. P.; Morgan, K. M.; Ogasawara, H.; O'Neil, G. C.; Reintsema, C. D.; Schmidt, D. R.; Sokaras, D.; Ullom, J. N.; Weng, T.-C.; Williams, C.; Young, B. A.; Swetz, D. S.; Irwin, K. D.; Nordlund, D. Soft X-Ray Spectroscopy with Transition-Edge Sensors at Stanford Synchrotron Radiation Lightsource Beamline 10-1. *Rev. Sci. Instrum.* **2019**, *90* (11), p 113101. DOI: 10.1063/1.5119155.

(24) Stavitski, E.; de Groot, F. M. F. The CTM4XAS Program for EELS and XAS Spectral Shape Analysis of Transition Metal L Edges. *Micron* **2010**, *41* (7), pp 687–694. DOI: 10.1016/j.micron.2010.06.005.

(25) Nehrkorn, J.; Martins, B. M.; Holldack, K.; Stoll, S.; Dobbek, H.; Bittl, R.; Schnegg, A. Zero-Field Splittings in MetHb and MetMb with Aquo and Fluoro Ligands: A FD-FT THz-EPR Study. *Mol. Phys.* **2013**, *111* (18–19), pp 2696–2707. DOI: 10.1080/00268976.2013.809806.

(26) Nehrkorn, J.; Holldack, K.; Bittl, R.; Schnegg, A. Recent Progress in Synchrotron-Based Frequency-Domain Fourier-Transform THz-EPR. *J. Magn. Reson.* **2017**, *280*, pp 10–19. DOI: 10.1016/j.jmr.2017.04.001.

(27) Schnegg, A.; Behrends, J.; Lips, K.; Bittl, R.; Holldack, K. Frequency Domain Fourier Transform THz-EPR on Single Molecule Magnets Using Coherent Synchrotron Radiation. *Phys. Chem. Chem. Phys.* **2009**, *11* (31), pp 6820–6825. DOI: 10.1039/b905745e.

(28) Nehrkorn, J.; Schnegg, A.; Holldack, K.; Stoll, S. General Magnetic Transition Dipole Moments for Electron Paramagnetic Resonance. *Phys. Rev. Lett.* **2015**, *114* (1), p 010801. DOI: 10.1103/PhysRevLett.114.010801.

(29) Nehrkorn, J.; Telser, J.; Holldack, K.; Stoll, S.; Schnegg, A. Simulating Frequency-Domain Electron Paramagnetic Resonance: Bridging the Gap between Experiment and Magnetic Parameters for High-Spin Transition-Metal Ion Complexes. *J. Phys. Chem. B* **2015**, *119* (43), pp 13816–13824. DOI: 10.1021/acs.jpcb.5b04156.

(30) Stoll, S.; Schweiger, A. EasySpin, a Comprehensive Software Package for Spectral Simulation and Analysis in EPR. *J. Magn. Reson.* **2006**, *178* (1), pp 42–55. DOI: 10.1016/j.jmr.2005.08.013.

(31) Nehrkorn, J.; Veber, S. L.; Zhukas, L. A.; Novikov, V. V.; Nelyubina, Y. V.; Voloshin, Y. Z.; Holldack, K.; Stoll, S.; Schnegg, A. Determination of Large Zero-Field Splitting in High-Spin Co(I) Clathrochelates. *Inorg. Chem.* **2018**, *57* (24), pp 15330–15340. DOI: 10.1021/acs.inorgchem.8b02670.

(32) Köhler, F. H. Paramagnetic Complexes in Solution: The NMR Approach. In *Encyclopedia of Magnetic Resonance*; John Wiley & Sons, Ltd: Chichester, UK, 2011. DOI: 10.1002/9780470034590.emrstm1229.

(33) Köhler, F. H. Probing Spin Densities by Use of NMR Spectroscopy. In *Magnetism: Molecules to Materials*; Wiley-VCH Verlag GmbH & Co. KGaA: Weinheim, Germany, 2003; Vol. 1–5, pp 379–430. DOI: 10.1002/9783527620548.ch12.

(34) Bertini, I.; Luchinat, C.; Parigi, G. Magnetic Susceptibility in Paramagnetic NMR. *Prog. Nucl. Magn. Reson. Spectrosc.* **2002**, *40* (3), pp 249–273. DOI: 10.1016/S0079-6565(02)00002-X.

(35) Pell, A. J.; Pintacuda, G.; Grey, C. P. Paramagnetic NMR in Solution and the Solid State. *Prog. Nucl. Magn. Reson. Spectrosc.* **2019**, *111*, pp 1–271. DOI: 10.1016/j.pnmrs.2018.05.001.

(36) Drago, R. S. *Physical Methods for Chemists*, 2nd ed.; Saunders College Publishing: Philadelphia, 1992.

(37) Bertini, I.; Luchinat, C.; Giacomo, P. *Solution NMR of Paramagnetic Molecules, Volume 2 of Current Methods in Inorganic Chemistry*, 1st ed.; Elsevier Science: Amsterdam, 2001.

(38) Bertini, I.; Luchinat, C. *NMR of Paramagnetic Molecules in Biological Systems*; Benjamin/Cummings Pub. Co.: Menlo Park, California, 1986.

(39) Bren, K. L. NMR Analysis of Spin Densities. In *Spin States in Biochemistry and Inorganic*

Chemistry; John Wiley & Sons, Ltd: Oxford, UK, 2015; pp 409–434. DOI: 10.1002/9781118898277.ch16.

(40) Bleaney, B. Nuclear Magnetic Resonance Shifts in Solution Due to Lanthanide Ions. *J. Magn. Reson.* **1972**, *8* (1), pp 91–100. DOI: 10.1016/0022-2364(72)90027-3.

(41) Bertini, I.; Luchinat, C. NMR of Paramagnetic Substances. *Coord Chem Rev.* **1996**, *150*, pp 29–75.

(42) La Mar, G. N. *NMR of Paramagnetic Molecules - Principles and Applications*; La Mar, G. N., Dew. Horrocks, W., Holm, R. H., Eds.; Academic Press, INC., New York and London, 1973.

(43) McGarvey, B. R. Theory of the NMR Paramagnetic Shift of Pseudotetrahedral Complexes of Nickel(II) and Cobalt(II). *Inorg. Chem.* **1995**, *34* (24), pp 6000–6007. DOI: 10.1021/ic00128a010.

(44) Damjanović, M.; Samuel, P. P.; Roesky, H. W.; Enders, M. NMR Analysis of an Fe(I)-Carbene Complex with Strong Magnetic Anisotropy. *Dalt. Trans.* **2017**, *46* (16), pp 5159–5169. DOI: 10.1039/C7DT00408G.

(45) Mao, J.; Zhang, Y.; Oldfield, E. Nuclear Magnetic Resonance Shifts in Paramagnetic Metalloporphyrins and Metalloproteins. *J. Am. Chem. Soc.* **2002**, *124* (46), pp 13911–13920. DOI: 10.1021/ja020297w.

(46) Knorr, R.; Hauer, H.; Weiss, A.; Polzer, H.; Ruf, F.; Löw, P.; Dvortsák, P.; Böhrer, P. Unpaired Spin Densities from NMR Shifts and Magnetic Anisotropies of Pseudotetrahedral Cobalt(II) and Nickel(II) Vinamidine Bis(Chelates). *Inorg. Chem.* **2007**, *46* (20), pp 8379–8390. DOI: 10.1021/ic700656r.

(47) Fernández, P.; Pritzkow, H.; Carbó, J. J.; Hofmann, P.; Enders, M. ^{1}H NMR Investigation of Paramagnetic Chromium(III) Olefin Polymerization Catalysts: Experimental Results, Shift Assignment and Prediction by Quantum Chemical Calculations. *Organometallics* **2007**, *26* (18), pp 4402–4412. DOI: 10.1021/om070173y.

(48) Roquette, P.; Maronna, A.; Reinmuth, M.; Kaifer, E.; Enders, M.; Himmel, H.-J. Combining NMR of Dynamic and Paramagnetic Molecules: Fluxional High-Spin Nickel(II) Complexes Bearing Bisguanidine Ligands. *Inorg. Chem.* **2011**, *50* (5), pp 1942–1955. DOI: 10.1021/ic102420x.

(49) Weber, B.; Walker, F. A. Solution NMR Studies of Iron(II) Spin-Crossover Complexes. *Inorg. Chem.* **2007**, *46* (16), pp 6794–6803. DOI: 10.1021/ic062349e.

(50) Breikss, A. I.; Nicholson, T.; Jones, A. G.; Davison, A. Synthesis and Characterization of Technetium(III) and Technetium(II) Complexes with Mixed Phosphine, Chloride, and Nitrogen-Donor Ligands. X-Ray Crystal Structure of (2,2'-Bipyridine)Trichloro(Triphenylphosphine)Technetium. *Inorg. Chem.* **1990**, *29* (4), pp 640–645. DOI: 10.1021/ic00329a017.

(51) *NMR of Paramagnetic Molecules*; Berliner, L. J., Reuben, J., Eds.; Biological Magnetic Resonance; Springer US: Boston, MA, 1993; Vol. 12. DOI: 10.1007/978-1-4615-2886-9.

(52) Bertini, I.; Turano, P.; Vila, A. J. Nuclear Magnetic Resonance of Paramagnetic Metalloproteins. *Chem. Rev.* **1993**, *93* (8), pp 2833–2932. DOI: 10.1021/cr00024a009.

(53) DE, S.; Tewary, S.; Garnier, D.; Li, Y.; Gontard, G.; Lisnard, L.; Flambard, A.; Breher, F.; Boillot, M.-L.; Rajaraman, G.; Lescouezec, R. Solution and Solid-State Study of the Spin-Crossover $[Fe^{II}(R\text{-}Bik)_3](BF_4)_2$ Complexes (R = Me, Et, Vinyl). *Eur. J. Inorg. Chem.* **2017**. DOI: 10.1002/ejic.201701013.

(54) Martin, R. L. Natural Transition Orbitals. *J. Chem. Phys.* **2003**, *118* (11), pp 4775–4777. DOI: 10.1063/1.1558471.

(55) Petzold, H.; Djomgoue, P.; Hörner, G.; Lochenie, C.; Weber, B.; Rüffer, T. Bis-Meridional Fe^{2+} Spincrossover Complexes of Phenyl and Pyridyl Substituted 2-(Pyridin-2-Yl)-1,10-Phenanthrolines. *Dalt. Trans.* **2018**, *47* (2), pp 491–506. DOI: 10.1039/C7DT02320K.
